# Genome-wide and evolutionary analysis of the class III peroxidase gene family in wheat and *Aegilops tauschii* reveals that some members are involved in stress responses

**DOI:** 10.1186/s12864-019-6006-5

**Published:** 2019-08-22

**Authors:** Jun Yan, Peisen Su, Wen Li, Guilian Xiao, Yan Zhao, Xin Ma, Hongwei Wang, Eviatar Nevo, Lingrang Kong

**Affiliations:** 1 College of Information Science and Engineering, Shandong Agricultural University, Tai’an, Shandong 271018 People’s Republic of China; 2 State Key Laboratory of Crop Biology, Shandong Key Laboratory of Crop Biology, College of Agronomy, Shandong Agricultural University, Tai’an, Shandong 271018 People’s Republic of China; 3 Institute of Evolution, University of Haifa, 199 Aba Khoushy Ave., Mount Carmel, 3498838 Haifa, Israel

**Keywords:** Wheat class III peroxidase gene family, Conserved exon-intron structures, Collinearity events, Expression pattern

## Abstract

**Background:**

The class III peroxidase (PRX) gene family is a plant-specific member of the PRX superfamily that is closely related to various physiological processes, such as cell wall loosening, lignification, and abiotic and biotic stress responses. However, its classification, evolutionary history and gene expression patterns are unclear in wheat and *Aegilops tauschii*.

**Results:**

Here, we identified 374, 159 and 169 PRXs in *Triticum aestivum*, *Triticum urartu* and *Ae. tauschii*, respectively*.* Together with PRXs detected from eight other plants, they were classified into 18 subfamilies. Among subfamilies V to XVIII, a conserved exon-intron structure within the “001” exon phases was detected in the PRX domain. Based on the analysis, we proposed a phylogenetic model to infer the evolutionary history of the exon-intron structures of PRX subfamilies. A comparative genomics analysis showed that subfamily VII could be the ancient subfamily that originated from green algae (*Chlamydomonas reinhardtii).* Further integrated analysis of chromosome locations and collinearity events of PRX genes suggested that both whole genome duplication (WGD) and tandem duplication (TD) events contributed to the expansion of *T. aestivum* PRXs (*Tae*PRXs) during wheat evolution. To validate functions of these genes in the regulation of various physiological processes, the expression patterns of PRXs in different tissues and under various stresses were studied using public microarray datasets. The results suggested that there were distinct expression patterns among different tissues and PRXs could be involved in biotic and abiotic responses in wheat. qRT-PCR was performed on samples exposed to drought, phytohormone treatments and *Fusarium graminearum* infection to validate the microarray predictions. The predicted subcellular localizations of some *Tae*PRXs were consistent with the confocal microscopy results. We predicted that some *Tae*PRXs had hormone-responsive *cis*-elements in their promoter regions and validated these predicted *cis*-acting elements by sequencing promoters.

**Conclusion:**

In this study, identification, classification, evolution, and expression patterns of PRXs in wheat and relative plants were performed. Our results will provide information for further studies on the evolution and molecular mechanisms of wheat PRXs.

**Electronic supplementary material:**

The online version of this article (10.1186/s12864-019-6006-5) contains supplementary material, which is available to authorized users.

## Background

Peroxidases (PRXs) are enzymes that catalyse the oxidation of many substrates by reducing hydrogen peroxide to water. They can be classified into two major groups: heme PRXs and non-heme PRXs. Heme PRXs can be further classified into two families: animal PRXs and non-animal PRXs. The non-animal PRXs contain three classes of PRXs, namely, class I, II and III PRXs [1]. Class I PRXs, such as microbial cytochrome c PRX, bacterial catalase-PRX and ascorbate PRX, are intracellular enzymes in plants, bacteria and yeast [2]. Class II PRXs are secreted oxidoreductive enzymes originating from fungi [3]. Class III PRXs are plant-specific secreted enzymes originating from plants [1].

Class III PRXs are involved in a broad range of physiological processes, such as cellular growth and cell wall loosening, lignification and suberisation, abiotic and biotic stress responses, fruit growth and ripening, and plant senescence [4]. By mediating the production of ROS (reactive oxygen species), a member of the cotton (*Gossypium hirsutum*) class III PRXs, *GhPOX1*, may play an important role during fibre cell elongation [5]. *Arabidopsis thaliana* class III PRX *AtPRX17* is the direct target of the transcription factor AGL15 and regulates lignified tissue formation [6]. *A. thaliana* class III PRX *AtPRX71* strengthens cell walls and restricts cell expansion in response to cell wall damage and during normal growth [7]. Four *A. thaliana* PRXs, *AtPrx 4*, *52*, *49* and *72*, were predicted to be involved in lignification [8]. Using a knock-out mutant of *AtPrx4*, *AtPrx4* was proven to be involved in cell wall lignification [9]. *AtPrx52* was also proven to be involved in the synthesis of interfascicular fibres during the lignification process, and the suppression of *AtPrx52* affected fibre lignification in *Arabidopsis* [10]. *AtPrx72* is involved in lignin biosynthesis [11]. In proanthocyanidin-deficient *A. thaliana* seeds, class III PRXs are significantly activated relative to wild-type seeds, resulting in lower levels of H_2_O_2_ [12]. Overexpression of the class III PRXs *AtPrx22*, *AtPrx39*, and *AtPrx69* increased cold tolerance in BRI1-GFP (green fluorescent protein) *A. thaliana* plants [13]. A putative *Coffea arabica* class III PRX was induced in response to root-knot nematode infection [14]. *Solanum lycopersicum* class III PRX *LePrx17* was induced by JA (jasmonic acid) and pathogen infection, and *LePrx09* was induced by ethephon, SA (salicylic acid), JA, pathogen infection, wounding and H_2_O_2_ stress [15]. Under arsenic (As) stress conditions, overexpression of the rice (*Oryza sativa*) class III PRX *OsPRX38* in *A. thaliana* increased PRX, SOD (superoxide dismutase) and GST (glutathione-S-transferase) activity and enhanced lignification, resulting in reduced As accumulation [16]. Overexpression of *A. thaliana* class III PRX *AtPrx64* in tobacco increased root growth and reduced the accumulation of aluminium (Al) and ROS in the roots, thereby improving tolerance to Al stress [17].

Only a few articles have reported the genome-wide identification of plant class III PRXs. In 2002, Tognolli et al. identified 73 class III PRXs in *A. thaliana* and analysed gene structures (intron/exon), gene duplication events, and expression patterns in different tissues (roots, stems, leaves and flowers) [18]. In 2004, Passardi et al. identified 138 rice class III PRXs and classified them into eight subfamilies (I-VIII) [19]. Despite the lack of complete plant genomes, they also studied the origin and expansion of class III PRXs by using EST (Expressed Sequence Tag) sequences and found 11–14 putative PRX sequences in *Physcomitrella patens* (moss) [19]. In 2014, Ren et al. identified 93 *Populus trichocarpa* class III PRXs and investigated the *Pt*PRX expression patterns in five tissues (roots, shoots, leaves, buds, and phloem) and under abiotic stresses (H_2_O_2_, SA, salt, and drought) [20]. They found two large tandem-arrayed clusters of *Pt*PRXs and identified seven positively selected sites in the four vacuole *Pt*PRXs (*PtPRX2*, *3*, *4*, and *7*). In 2015, Wang et al. identified 119 maize (*Zea mays*) class III PRXs and divided them into 18 groups [21]. They identified 16 related segmental duplication events and 12 tandem duplication events, calculated the *Ka* (non-synonymous substitution) */Ks* (synonymous substitution) values and found that most *Zm*PRXs underwent purifying selection. Expression pattern analysis of *Zm*PRXs was also performed using a public microarray dataset and qRT-PCR (quantitative real-time PCR) under H_2_O_2_, SA, NaCl and PEG (polyethylene glycol) stress treatments. In 2016, Cao et al. identified 94 pear (*Pyrus bretschneideri*) class III PRXs and performed analyses of duplication events, conserved motifs, *Ka/Ks* values and expression patterns by qRT-PCR [22].

Some articles studied the phylogeny of class III PRXs. In 2006, a class III plant PRX database, PeroxiBase, was published [23]. In 2015, a phylogenetic reconstruction of the non-animal PRX superfamily (class I-III PRXs, and others) was performed to trace their molecular evolution, and two additional class I members were identified [24]. In 2015, *Ka/Ks* values of 62 *A. thaliana* class III PRXs were calculated to examine their evolutionary divergence, and the nucleotide and amino acid substitutions of the duplicated genes *AtPrx53*-*AtPrx54* and *AtPrx36*-*AtPrx72* (*Ka/Ks* > 2) were identified as positive selection targets [25].

In this study, we performed genome-wide identification, evolution analysis and expression pattern analysis of class III PRXs in wheat and *Aegilops tauschii.* We identified PRXs in *Triticum aestivum*, *Triticum urartu*, *Ae. tauschii,* and eight other plant species and classified them into 18 subfamilies. We found that PRX subfamily VII first appeared in green algae (*Chlamydomonas reinhardtii)*, and then VII and I appeared in moss (*P. patens*). A conserved exon-intron structure within the “001” exon phases in the PRX domain was shared in subfamilies V-XVIII. An evolutionary model of PRX exon-intron structures was proposed. Chromosome locations and collinearity events of PRXs were identified in *T. aestivum* and related genomes to study the expansion and evolution of wheat PRXs. The expression patterns of tissues, abiotic stress responses and biotic stress responses were analysed using public microarray datasets. To validate the microarray predictions, qRT-PCR was performed on samples under drought conditions, samples with four phytohormone treatments and samples with *Fusarium graminearum* (*Fg*) infection. The confocal microscopy results validated the predicted subcellular localizations of some *Tae*PRXs. Sequencing promoters of some *Tae*PRXs validated the predicted hormone-responsive *cis*-elements. Our work will help researchers study the evolution and molecular mechanism of wheat PRXs.

## Results

### Genome-wide identification and classification of class III PRXs in wheat, *Ae. tauschii* and other plants

We searched the *T. aestivum*, *T. urartu,* and *Ae. tauschii* proteomes by using HMMER 3.1 and Pfam 30.0 in batch mode with the PRX domain (see Methods). The results showed that 374, 159 and 169 typical class III PRXs were identified in *T. aestivum*, *T. urartu* and *Ae. tauschii*, respectively. We also identified PRXs in eight other plant proteomes, including *Brachypodium distachyon*, *Z. mays*, *O. sativa*, *A. thaliana*, *Vitis vinifera, Selaginella moellendorffii*, *P. patens* and *C. reinhardtii* (Table 1, Additional file 13: Table S1). Atypical PRXs of these eleven plants, with less than 50% PRX domain alignment, were excluded in the following analysis (Additional file 14: Table S2).

**Table 1:** The numbers of class III peroxidase gene families in 11 plants

Species	Number
*Chlamydomonas reinhardtii*	6
*Physcomitrella patens*	60
*Selaginella moellendorffii*	167
*Vitis vinifera*	85
*Arabidopsis thaliana*	81
*Zea mays*	156
*Oryza sativa*	125
*Brachypodium distachyon*	149
*Aegilops tauschii*	169
*Triticum urartu*	159
*Triticum aestivum*	374

**Fig. 1:**
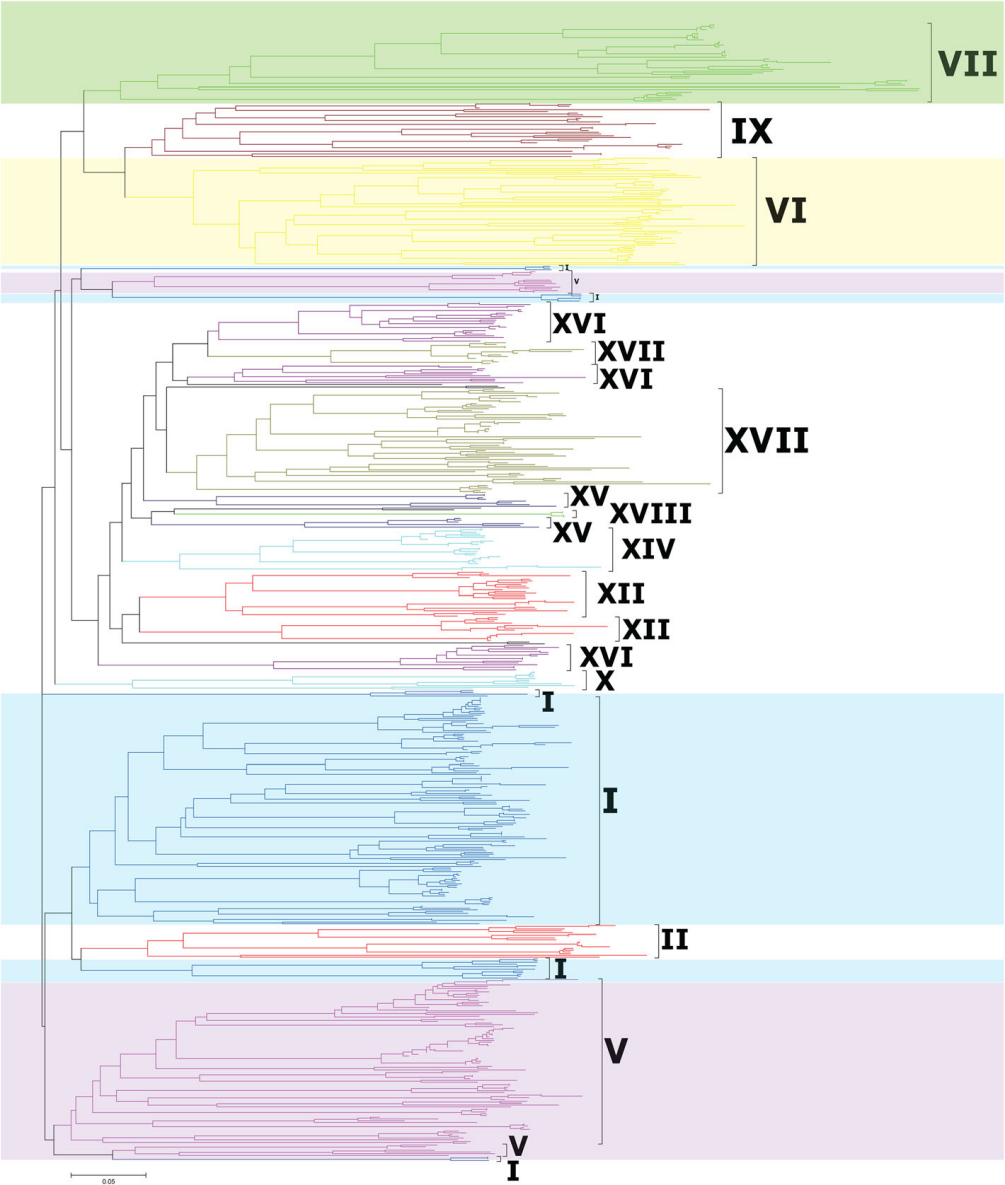
Classification and phylogenetic relationships of the class III peroxidases in wheat and *Ae. tauschii*. The Neighbour-Joining tree was constructed by the amino acid sequences of the PRX domain using MEGA-CC 7.0 with the p-distance model. Major groups are labelled with different colours. Detailed information is provided in Additional file 1: Figure S1a

The classification of PRXs was based on two methods. First, the classification of PRXs in eleven plants was performed against the HMM (The hidden Markov model) based on the maize PRX alignments of Wang [21] (Additional file 13: Table S1). The results showed that PRXs were classified into I-XVIII subfamilies in *T. aestivum*, *T. urartu* and *Ae. tauschii*. Second, to verify the HMM classification, we constructed a neighbour-joining (NJ) phylogenetic tree of PRXs based on *T. aestivum*, *T. urartu* and *Ae. tauschii* truncated PRX domain sequences with the p-distance model and 1000 bootstraps (Fig. 1, Additional file 1: Figure S1a). A large NJ tree of PRXs was also constructed based on the truncated PRX domain sequences of these eleven plants (Additional file 1: Figure S1b). Interestingly, almost all PRXs belonging to the same clade indicated the same subfamily classification as that identified by HMMER. Large numbers of members were found in subfamilies I, V, VI and VII (Fig. 1). Some species-specific PRX clusters were found in *V. vinifera*, *S. moellendorffii* and *P. patens*. According to HMM and NJ classification, all six *C. reinhardtii* PRXs belong to subfamily VII, suggesting that PRX subfamily VII might be the ancient subfamily.

### Class III PRX evolution and conserved exon- intron structures

**Fig. 2:**
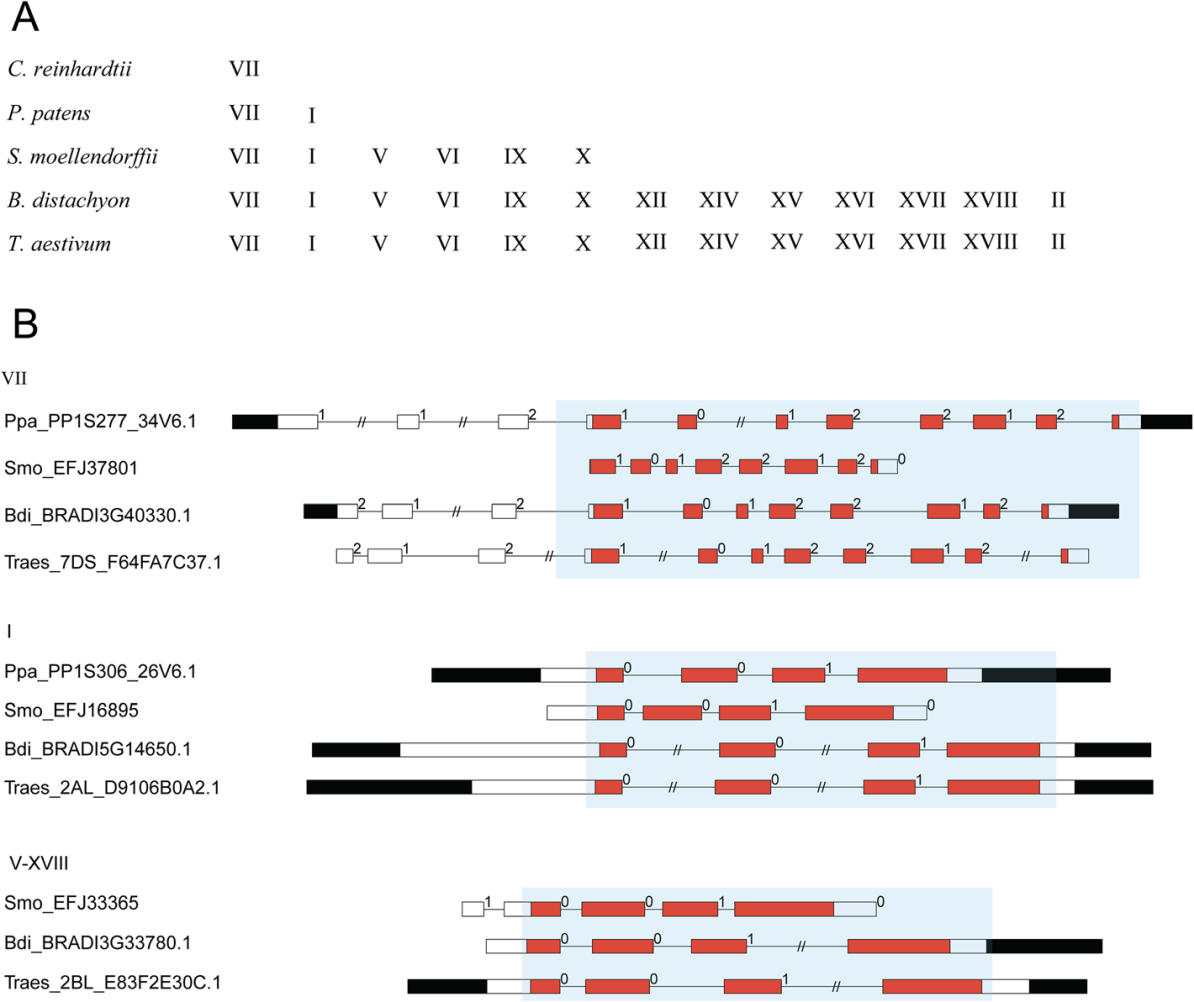
Conserved exon-intron structures of PRXs. **a** The evolutionary history of the PRX subfamilies. **b** The diagram indicates that conserved exon-intron structures with conserved exon phases were present in the PRX domain. Filled boxes: red represents the PRX domain; black boxes: untranslated regions (UTRs); white boxes: other exon regions; lines: introns; numbers 0, 1, and 2: exon phases. The lengths of the boxes and lines are scaled based on the lengths of the genes. The long introns are shortened by “//”

We summarized the evolutionary process of plant class III PRXs by investigating the subfamily distributions in *T. aestivum*, *B. distachyon*, *S. moellendorffii*, *P. patens* and *C. reinhardtii* (Fig. 2a). The results indicated that subfamily VII first appeared in green algae (*C. reinhardtii).* Then, subfamilies VII and I were found in primitive plant moss (*P. patens*). The PRXs of fern (*S. moellendorffii*) expanded into six subfamilies, namely, VII, I, V, VI, IX and X. The PRXs of monocots (*T. aestivum* and *B. distachyon*) expanded into thirteen subfamilies, namely, VII, I, V, VI, IX, X, XII, XIV-XVIII and II.

To obtain further insights into PRX evolution, we diagrammed the exon-intron structures within the PRX domain in the eleven investigated plants (Additional file 2: Figure S2). The results showed that some conserved exon-intron structures were present in the same subfamilies of PRXs across the investigated plants, especially in the PRX domain. We summarized these conserved exon-intron structures in *T. aestivum*, *B. distachyon*, *S. moellendorffii*, and *P. patens* (Additional file 3: Figure S3)*.* For instance, the PRX subfamily I sequences of *T. aestivum* (Traes_1BS_8A9C9C25B.1), *B. distachyon* (BRADI2G37000.1), *S. moellendorffii* (EFJ22715), and *P. patens* (PP1S46_76V6.1) shared the same conserved exon-intron structure within the “01” exon phases in the PRX domain. Based on the above analysis, we noticed that most subfamilies V-XVIII shared the same conserved exon-intron structure within the “001” exon phases in the PRX domain (Fig. 2b, Additional file 3: Figure S3). These “001” exon phases could also be found in subfamily I but not in VII. Subfamily VII contained three types of conserved exon-intron structures, which were within the “1012–212”, “2100–1020” and “0101–0000” exon phases in the PRX domain (Additional file 3: Figure S3).

### Chromosome locations and duplication events of *T. aestivum* class III PRXs

We mapped *T. aestivum* class III PRXs to chromosome positions (Additional file 4: Figure S4, Additional file 15: Table S3). Two hundred and sixty-nine of 374 *T. aestivum* PRXs could be mapped on the chromosomes; the others were in scaffolds. These 269 *T. aestivum* PRXs were unevenly distributed among 21 chromosomes. Chromosomes 1A, 1B, 1D, 2A, 2B, 2D and 7D contained more PRXs than other *T. aestivum* chromosomes.

**Fig. 3:**
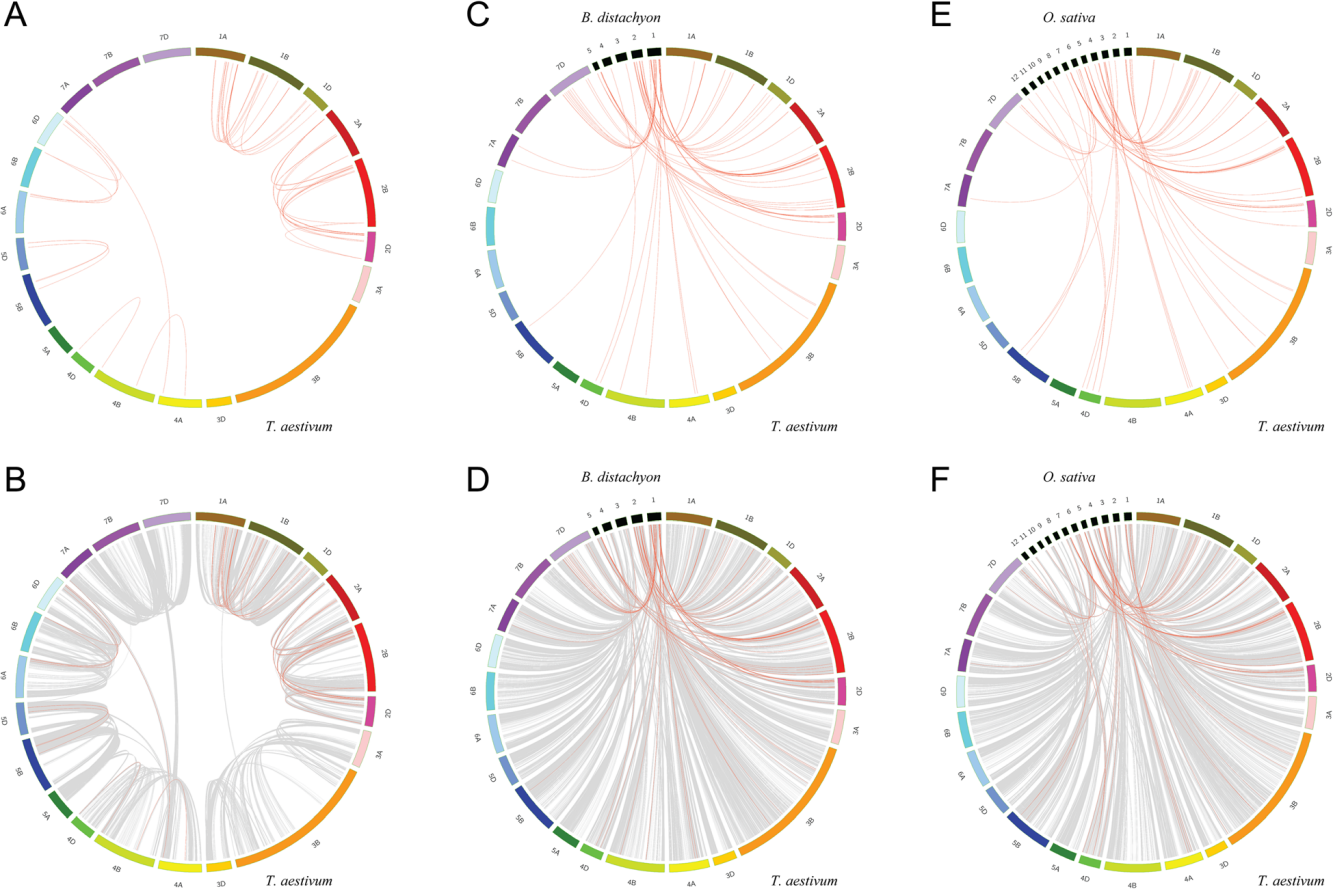
Synteny analysis of PRX genes. This graph displays syntenic maps of *T. aestivum* associated with two *Graminaceae* (*B. distachyon* and *O. sativa*). Red curves represent syntenic gene pairs between the PRXs, and grey curves represent other genes. **a** Synteny of PRXs in *T. aestivum*. **b** Synteny of PRXs and other genes in *T. aestivum*. **c** Synteny of PRXs in *T. aestivum* and *B. distachyon*. **d** Synteny of PRXs and other genes in *T. aestivum* and *B. distachyon*. **e** Synteny of PRXs in *T. aestivum* and *O. sativa*. **f** Synteny of PRXs and other genes in *T. aestivum* and *O. sativa*

The allohexaploid bread wheat (*T. aestivum*) genome consists of three subgenomes (A, B, and D), which are involved in three rounds of polyploidization [26]. To detect the relationship between PRX expansion and *T. aestivum* polyploidization, we identified 46 collinearity events by using MCscanX (Additional file 16: Table S4). The chromosome locations of related PRXs were distributed among chromosomes 1A, 1B, 1D, 2A, 2B, 2D, 4A, 4B, 4D, 5B, 5D, 6A, 6B, 6D and 7A (Fig. 3a). Our previous research demonstrated a peak *Ks* value of 0.03–0.45 for collinearity events of *T. aestivum* PKs (protein kinase) [27]. In this study, we also found that most *Ks* values of *T. aestivum* PRXs were less than 0.45, hinting that these *T. aestivum* PRX collinearity events could be attributed to *T. aestivum* polyploidization. Furthermore, we detected the PRX collinearity events among all synteny blocks in the *T. aestivum* genome (Fig. 3b).

To further infer the phylogenetic mechanism of the *T. aestivum* class III PRX gene family, comparative syntenic maps of *T. aestivum* associated with two *Graminaceae* (*B. distachyon* and *O. sativa*) were constructed (Fig. 3c-f, Additional file 16: Table S4). The results showed that 73 syntenic PRX gene pairs were detected between *T. aestivum* and *B. distachyon* (Fig. 3c). Similarly, 63 syntenic PRX gene pairs were detected between *T. aestivum* and *O. sativa* (Fig. 3e)*.* We noticed that some syntenic PRX gene pairs shared the same *T. aestivum* PRX member associated with *B. distachyon* and *O. sativa,* suggesting that they might originate from a common ancestor before the *Graminaceae* split. For instance, the *Bdi-Tae* gene pair (BRADI1G20010.1 and Traes_2AS_5DF52D5D1.1) and *Osa-Tae* gene pair (Traes_2AS_5DF52D5D1.1 and OS07T0638600–00) shared the same *T. aestivum* PRX member (Traes_2AS_5DF52D5D1.1). We also calculated the *Ka/Ks* values of syntenic PRX gene pairs between the *T. aestivum* A subgenome and *T. urartu* (86 gene pairs) and the *T. aestivum* D subgenome and *Ae. tauschii* (104) (Additional file 17: Table S5).

**Fig. 4:**
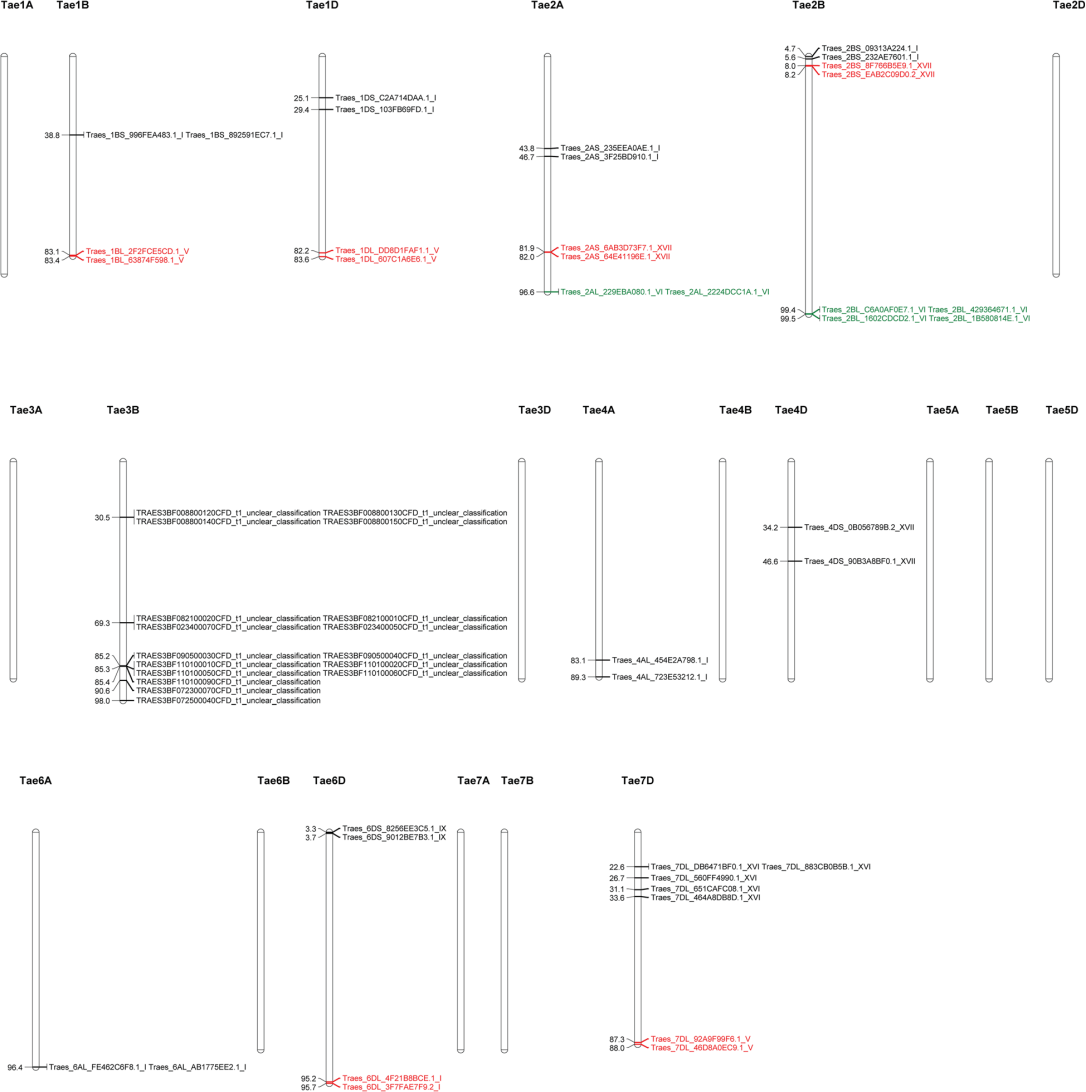
Chromosomal locations of the tandemly arrayed *T. aestivum* PRX genes. The 56 tandemly arrayed *T. aestivum* PRX genes were grouped into 18 clusters distributed unevenly among the 10 chromosomes. Gene IDs and subfamilies are labelled on the right of each chromosome, and the chromosomal location of each cluster is on the left of each chromosome. Genes in the same cluster are highlighted in the same colour

We identified 56 tandem *T. aestivum* PRX genes on 10 chromosomes, including 1B, 1D, 2A, 2B, 3B, 4A, 4D, 6A, 6D and 7D (Fig. 4, Additional file 18: Table S6). The genes formed 18 clusters on these 10 chromosomes. The number of members in each cluster ranged from 2 to 5, whereas the largest cluster was subfamily XVI, located on chromosome 7D.

### Expression patterns of *T. aestivum* class III PRXs in different tissues

We performed a microarray-based expression pattern analysis of *T. aestivum* class III PRXs using 11 public datasets from the Affymetrix microarray GPL3802 platform. The results showed that 170 of 374 *T. aestivum* PRXs have probes in GPL3802. Based on the quality control of the NUSE (Normalized unscaled standard errors) and RLE (Relative log expression) diagrams (Additional file 5: Figure S5), we excluded 4 CEL files in the following analysis (Additional file 19: Table S7). The expression patterns of the 170 *T. aestivum* PRXs were investigated in different tissues, including three tissues (coleoptile, root and embryo) in the germinating seed stage, three tissues (root, crown and leaf) in the seedling stage, immature inflorescence tissue, three tissues (floral bracts, pistil and anthers) before anthesis, caryopsis tissue at 3–5 DAP (day after planting), embryonic tissue at 22 DAP, and endosperm tissue at 22 DAP (Additional file 6: Figure S6, Additional file 20: Table S8). The results showed that some *T. aestivum* PRXs, such as Traes_2BL_2B45081D4.1 (VI), Traes_2AL_2224DCC1A.1 (VI) and Traes_4DL_8CE055F15.1 (VII), showed high expression levels in all investigated tissues. Similarly, some *T. aestivum* PRXs, such as Traes_4AL_0C8DFDE2B.1 (VI) and Traes_2AS_AB001AAB7.1 (XVII), exhibited low expression levels in all investigated tissues. We also noticed that some *T. aestivum* PRXs exhibited different expression levels in different tissues. For example, Traes_7DL_651CAFC08.1 (XVI) exhibited high expression level in the root of the seedling stage but relatively low expression levels in the other 11 tissues. Approximately 13 *T. aestivum* PRXs, such as Traes_7DL_6233C6F03.1 (I) and Traes_1AL_91E56EC8C.1 (V), exhibited high expression levels in two tissues, the root in the germinating seed stage and the root in the seedling stage, suggesting that they might participate in the development of the *T. aestivum* root.

### Subcellular localization of *T. aestivum* class III PRXs

**Fig. 5:**
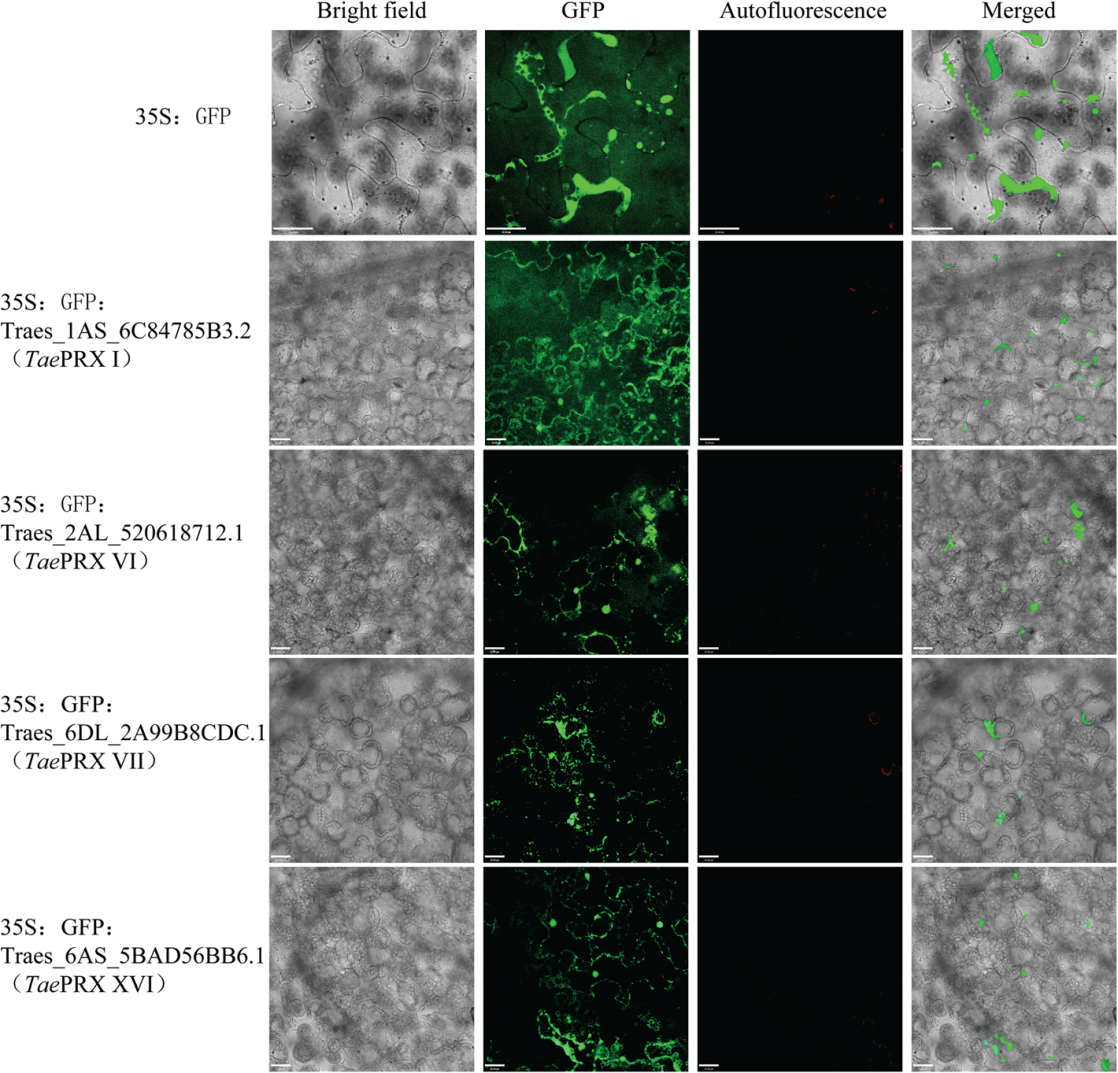
The subcellular localization of *Tae*PRX proteins in tobacco leaves. Localization of GFP signals from *Tae*PRX proteins fused with GFP. Bright field, epifluorescence, chloroplast autofluorescence and merged images of tobacco leaves transfected with constructs expressing different fusion proteins. Bars = 32 μm

We selected four *T. aestivum* class III PRXs from different subfamilies to investigate their subcellular localization. The prediction websites WoLF PSORT and TargetP were used to predict their subcellular localization (Table 2). To test the predicted subcellular localization of *Tae*PRXs, N-terminal GFP-fused *Tae*PRX proteins were expressed in tobacco leaves (Fig. 5). Interestingly, most of the predicted subcellular localizations were consistent with the confocal microscopy results (Table 2). For instance, Traes_6DL_2A99B8CDC.1 (VII) was predicted to be expressed in the chloroplast by WoLF PSORT and TargetP. Indeed, Traes_6DL_2A99B8CDC.1 (VII) was expressed in the chloroplast according to confocal microscopy.

**Table 2:** Predicted and experimental subcellular localization of *Tae*PRXs

Gene ID	Subfamily	Subcellular localization (WoLF PSORT)	Subcellular localization (TargetP)	Subcellular localization (confocal microscopy)
Traes_1AS_6C84785B3.2	I	extr: 11, chlo: 1, mito: 1	Secretory pathway	cell membrane
Traes_2AL_520618712.1	VI	extr: 10, vacu: 4	Secretory pathway	vacuole
Traes_6DL_2A99B8CDC.1	VII	chlo: 13	Chloroplast	thylakoid, chloroplast, cell membrane
Traes_6AS_5BAD56BB6.1	XVI	chlo: 7, extr: 3, vacu: 2, nucl: 1	Secretory pathway	vacuole, cell membrane, nucleus, cytoplasm

### Expression patterns of *T. aestivum* class III PRXs under abiotic stress

The expression patterns of 170 *T. aestivum* class III PRXs were determined under various abiotic stress treatments (Additional file 7: Figure S7, Additional file 8: Figure S8, Additional file 21: Table S9). To search for the differentially expressed genes under abiotic stress, *T. aestivum* PRXs with |FC| > 1.5 (fold change) and *p* < 0.05 were selected (Additional file 22: Table S10). (1) Cold: under cold treatment, most *T. aestivum* PRXs exhibited the same expression trend in the two wheat genotypes (Freeze Resistance “SD16029” and Freeze Susceptible “SD16169”). For instance, Traes_2AL_2224DCC1A.1 (VI) and Traes_2BL_2B45081D4.1 (VI) showed upregulation in “SD16029” and “SD16169” under cold stress, hinting that these *T. aestivum* PRXs might regulate the cold stress response. Similarly, more PRXs, such as Traes_1DS_3D2F70A22.1 (I) and Traes_1AS_6C84785B3.2 (I), exhibited downregulation in “SD16029” and “SD16169” under cold stress. (2) Heat: under heat stress, most *T. aestivum* PRXs exhibited slight upregulation or downregulation. However, we also noticed that two PRXs, Traes_4DL_A6041E3DC.1 (V) and Traes_2AS_2B95E681C.1 (VII), exhibited dramatic upregulation under heat stress. (3) Drought: some PRXs exhibited slight upregulation or downregulation in both wheat genotypes (drought-susceptible “WL711” and drought-tolerant “C306”) under drought stress. A few PRXs also showed reverse expression trends in “WL711” and “C306”. For example, under drought stress, Traes_1DS_578ADDF80.1 (XII) exhibited upregulation in “WL711” but downregulation in “C306”. (4) Nutrient deficiency: under the five nutrient-deficient stress treatments, most PRXs showed slight upregulation or downregulation. However, we also noticed that a few PRXs, such as Traes_2AS_3161D54F8.1 (XVII), Traes_7DL_D99ED7064.1 (XII) and Traes_7AL_80967149B.1 (XII), exhibited dramatic downregulation with only the no-phosphate fertilization treatment. Two PRXs, Traes_7DL_602B9D252.1 (XVI) and Traes_3AL_78711D4EB.1 (XII), showed dramatic upregulation with the no-sulphate fertilization treatment. (5) Seven phytohormones: most PRXs showed slight upregulation or downregulation under the seven phytohormones. For instance, Traes_3B_8732922B8.1 (VI) showed slight upregulation under all seven phytohormones (SA 0.19, MeJA (methyljasmonic acid) 0.08, GA (gibberellic acid) 0.33, ABA (abscisic acid) 0.48, CK (trans-zeatin,one kind of cytokinins) 0.18, ET (ethylene) 0.55, and IAA (indole-3-acetic acid) 0.47). However, we also noticed that some *T. aestivum* PRXs, such as Traes_2AS_3161D54F8.1 (XVII), Traes_2DS_708F03DA3.1 (XVII) and Traes_2BS_B6EBC0962.1 (XVII), exhibited dramatic upregulation with only MeJA treatment. Similarly, some PRXs, such as Traes_2AL_2224DCC1A.1 (VI), Traes_5BL_3ED1B0234.2 (XVII) and Traes_1DL_607C1A6E6.1 (V), exhibited dramatic downregulation with ABA treatment.

**Fig. 6:**
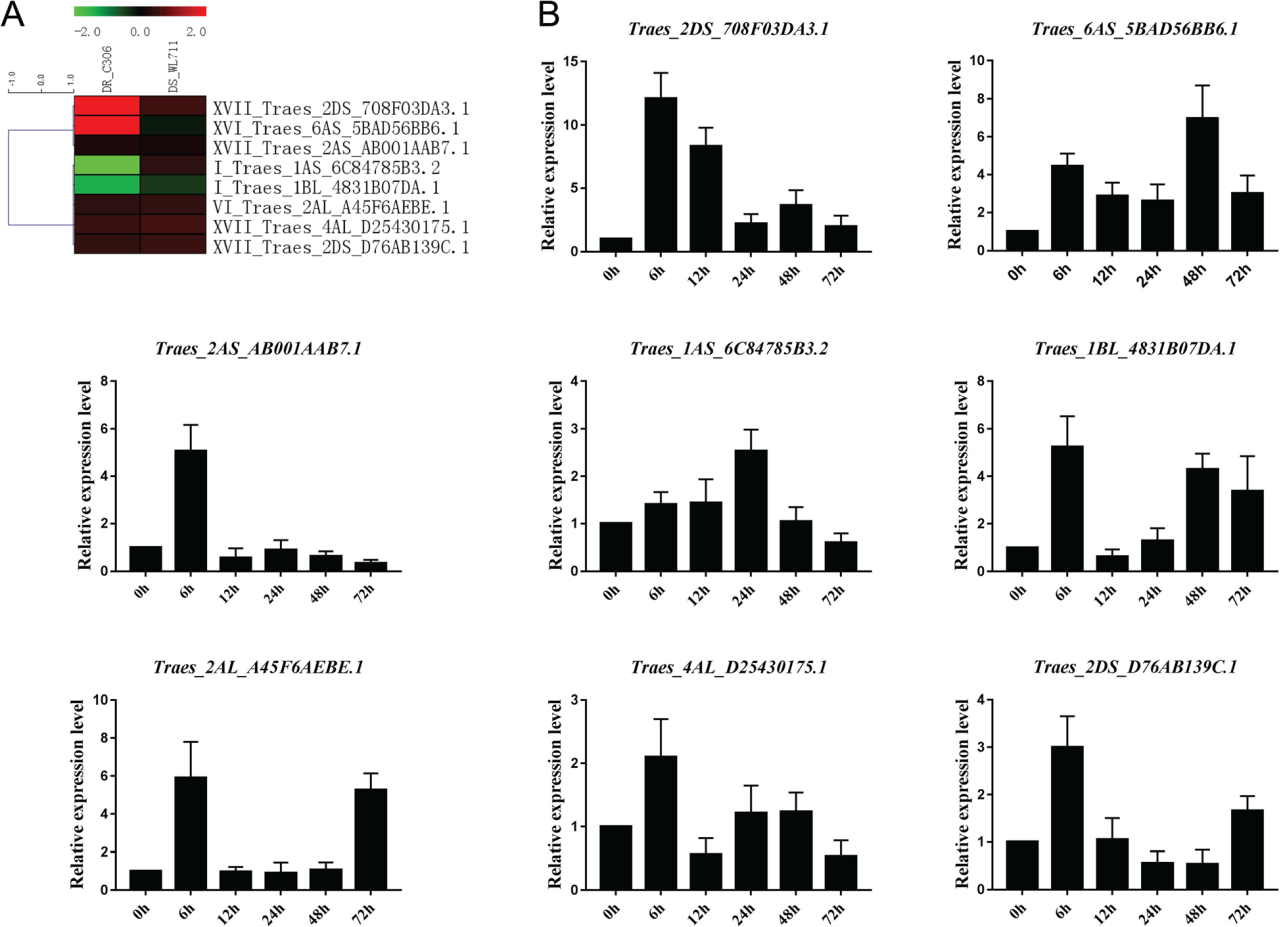
Heatmap of eight selected *T. aestivum* PRXs and their qRT-PCR results under drought conditions. **a** Heatmap of the microarray. **b** qRT-PCR under PEG-600 treatment

To further confirm the expression pattern analysis of the GEO (Gene Expression Omnibus) microarray, we selected eight *T. aestivum* PRXs to examine their expression in the cultivar “Sumai-3” under PEG (drought) treatment using qRT-PCR (Fig. 6). Some of the eight PRXs exhibited similar microarray and qRT-PCR results. (1) Traes_2DS_708F03DA3.1 (XVII): the log2 values of “WL711” (drought-susceptible) and “C306” (drought-tolerant) under drought conditions were 0.55 and 2.87, while our qRT-PCR results showed upregulation at 6–72 h (h) after PEG treatment. (2) Traes_6AS_5BAD56BB6.1 (XVI): the microarray prediction of “C306” was consistent with our qRT-PCR results. The log2 value of “C306” was 2.05, while our qRT-PCR results exhibited upregulation at 6–72 h. (3) Traes_2AS_AB001AAB7.1 (XVII): the log2 values of “WL711” and “C306” were 0.18 and 0.22, respectively, while our qRT-PCR results showed upregulation at 6 h. (4) Traes_2AL_A45F6AEBE.1 (VI): the log2 values of “WL711” and “C306” were 0.42 and 0.38, respectively, while our qRT-PCR results showed upregulation at 6 and 72 h. (5) Traes_4AL_D25430175.1 (XVII): the log2 values of “WL711” and “C306” were 0.59 and 0.51, respectively, while our qRT-PCR results showed upregulation at 6 h. (6) Traes_2DS_D76AB139C.1 (XVII): the log2 values of “WL711” and “C306” were 0.52 and 0.44, respectively, while our qRT-PCR results showed upregulation at 6 h.

**Fig. 7:**
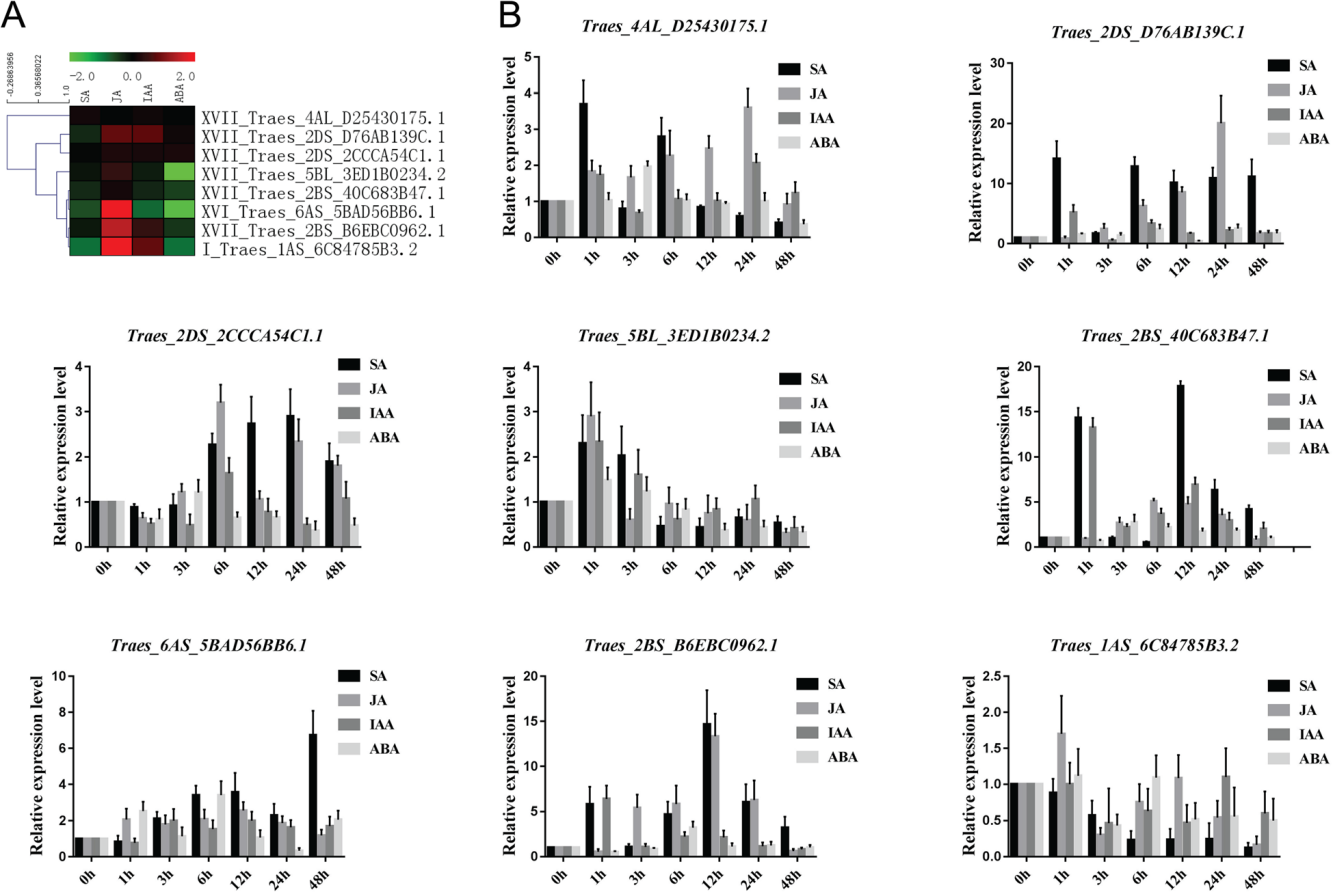
Heatmap of eight selected *T. aestivum* PRXs and their qRT-PCR results under four phytohormone treatments. **a** Heatmap of the microarray. **b** qRT-PCR under SA, JA, IAA and ABA treatments

To confirm the phytohormone microarray predictions, we also selected eight PRXs and examined their expression under four phytohormone (SA, JA, IAA and ABA) treatments using qRT-PCR (Fig. 7). Some of the eight PRXs exhibited similar microarray and qRT-PCR results. (1) Traes_4AL_D25430175.1 (XVII): the log2 values under SA, JA, IAA and ABA treatments were 0.14, 0.02, 0.11 and 0.02, respectively, while our qRT-PCR results remained at the same expression level at 6 and 12 h (compared with 0 h) under IAA and ABA treatments. Under JA treatment, this *Tae*PRX exhibited upregulation at 1–48 h and reached peak expression at 24 h. (2) Traes_2DS_D76AB139C.1 (XVII): the log2 values under SA, JA, IAA and ABA treatments were − 0.30, 0.80, 0.76 and 0.11, respectively, while our qRT-PCR results showed dramatic upregulation at 1 and 6 h under IAA treatment. Under ABA treatment, this *Tae*PRX exhibited slight upregulation at 1, 3, 6, 24 and 48 h. With JA treatment, this *Tae*PRX exhibited upregulation at 6–24 h and reached an approximately 20-fold peak in expression at 24 h. (3) Traes_2DS_2CCCA54C1.1 (XVII): the log2 values under SA, JA, IAA and ABA treatments were − 0.03, 0.22, 0.19 and 0.20, respectively, while our qRT-PCR results showed upregulation at 3–48 h under JA treatment. (4) Traes_5BL_3ED1B0234.2 (XVII): the log2 values under SA, JA, IAA and ABA treatments were − 0.12, 0.44, − 0.19 and − 2.57, respectively, while our qRT-PCR results showed downregulation at 6 h under SA and IAA treatments. Under ABA treatment, this *Tae*PRX exhibited downregulation at 12–48 h. Under JA treatment, this *Tae*PRX exhibited upregulation at 1 h. (5) Traes_2BS_40C683B47.1 (XVII): the log2 values under SA, JA, IAA and ABA treatments were − 0.32, 0.15, − 0.28 and − 0.50, respectively, while our qRT-PCR results showed upregulation at 3–24 h under JA treatment. (6) Traes_6AS_5BAD56BB6.1 (XVI): the log2 values under SA, JA, IAA and ABA treatments were − 0.60, 1.97, − 0.81 and − 1.88, respectively, while our qRT-PCR results showed downregulation at 1 h under SA and IAA treatments. Under JA treatment, this *Tae*PRX exhibited upregulation at 1–24 h. (7) Traes_2BS_B6EBC0962.1 (XVII): the log2 values under SA, JA, IAA and ABA treatments were − 0.15, 1.47, 0.46 and − 0.42, respectively, while our qRT-PCR results showed that this *Tae*PRX reached an expression peak at 1 h and still exhibited slight upregulation at 6–12 h under IAA treatment. Under ABA treatment, this *Tae*PRX exhibited slight downregulation at 1–3 h. Under JA treatment, this *Tae*PRX exhibited upregulation at 3–24 h and reached an approximately 12-fold expression peak at 12 h. (8) Traes_1AS_6C84785B3.2 (I): the log2 values under SA, JA, IAA and ABA treatments were − 0.87, 2.72, 0.82 and − 0.88, respectively, while our qRT-PCR results showed that this *Tae*PRX exhibited downregulation and slowly declined at 1–48 h under SA treatment. Under ABA treatment, this *Tae*PRX exhibited downregulation at 3 and 12–48 h. Under JA treatment, this *Tae*PRX exhibited upregulation at 1 h.

To study the mechanism of *Tae*PRX expression induced by phytohormones, we predicted *cis*-acting elements in the upstream promoters of *Tae*PRXs by using PlantCARE42 (Additional file 9: Figure S9 and Additional file 23: Table S11). The results showed that almost all investigated *Tae*PRXs promoters contained the putative *cis*-acting elements responding to MeJA and ABA (Table 3). Some of these predicted results were consistent with the qRT-PCR and microarray results. For instance, the log2 value of Traes_2DS_D76AB139C.1 (XVII) by microarray under ABA treatment was 0.11, and the qRT-PCR results showed that this *Tae*PRX exhibited slight upregulation at 1, 3, 6, 24 and 48 h under ABA treatment (Fig. 7). Indeed, the promoter of this *Tae*PRX contained *cis*-acting elements responding to ABA.

**Table 3:** Predicted and experimental *cis*-acting elements related to stress or hormone response

Gene ID	Subfamily	Predicted *cis*-acting elements related to stress or hormone response	Experimental verification of *cis*-acting elements using sequencing
Traes_2DS_D76AB139C.1	XVII	MeJA, GA, ABA, IAA	MeJA, GA, ABA, IAA
Traes_2DS_2CCCA54C1.1	XVII	MeJA, SA, ABA, Low temperature, Drought inducibility	MeJA, ABA, Low temperature
Traes_5BL_3ED1B0234.2	XVII	MeJA, SA, GA, ABA, Anaerobic induction, Drought inducibility	no sequencing
Traes_2BS_40C683B47.1	XVII	MeJA, ABA, SA, Anaerobic_induction	MeJA, ABA, SA, Anaerobic_induction
Traes_6AS_5BAD56BB6.1	XVI	SA, GA, ABA, Anaerobic induction, Drought inducibility	SA, GA, ABA, Anaerobic induction
Traes_2BS_B6EBC0962.1	XVII	MeJA, IAA, Anaerobic induction, SA, ABA	MeJA, IAA, Anaerobic induction, SA
Traes_1AS_6C84785B3.2	I	MeJA, ABA, Low temperature, Drought inducibility, SA	MeJA, ABA, Low temperature, Drought inducibility, SA

To test these predicted *cis*-acting elements, sequencing validation of these upstream promoter sequences was performed (Additional file 10: Figure S10). The results showed that almost all predicted *cis*-acting elements were present in sequencing promoters. However, we found a *cis*-acting element within SNP (single nucleotide polymorphism) in Traes_6AS_5BAD56BB6.1 (XVI). This *cis*-acting element (GTGCAC) was responsive to ABA. SNP could also be found in other regions of the promoter of Traes_6AS_5BAD56BB6.1 (XVI).

We also compared four *T. aestivum* PRXs and their homologous maize PRXs [21] using qRT-PCR (Additional file 11: Figure S11). The results showed that three *T. aestivum* PRXs had similar expression trends as those of homologous maize PRXs under SA treatment. (1) Traes_4DS_90B3A8BF0.1 (XVII): both Traes_4DS_90B3A8BF0.1 and its homologous maize PRX (*ZmPRX71* GRMZM2G171078_T02) exhibited downregulation at 3 h under SA treatment. The expression of both genes recovered by one-fold at 6 h. (2) Traes_2AS_E509AB03B.1 (XVII): the homologous maize PRX (*ZmPRX26*, GRMZM2G133475_T01) showed a 2.5-fold upregulation at 12 h under SA treatment, while our qRT-PCR results showed that Traes_2AS_E509AB03B.1 exhibited an approximately 1.5-fold upregulation at 12 h under SA treatment. (3) Traes_2AS_6AB3D73F7.1 (XVII): both Traes_2AS_6AB3D73F7.1 and its homologous maize PRX (*ZmPRX75*, GRMZM2G025441_T01) exhibited approximately 2-fold upregulation at 6 h under SA treatment.

### Expression patterns of *T. aestivum* class III PRXs under biotic stress

We studied the expression patterns of 170 *T. aestivum* class III PRXs under biotic stress treatments (Additional file 12: Figure S12, Additional file 24: Table S12). The differentially expressed genes of *T. aestivum* PRXs (|FC| > 1.5 and *p* < 0.05) under biotic stress were also determined (Additional file 25: Table S13). (1) Fusarium head blight (FHB): we noticed that a few *T. aestivum* PRXs, such as Traes_1BS_BA046E212.1 (XII), Traes_2BL_E8A65526C.1 (VI), Traes_7DL_602B9D252.1 (XVI) and Traes_2DS_D76AB139C.1 (XVII), exhibited progressive upregulation at 1–4 days (d) after *Fg* infection, suggesting that they might participate in the pathway responding to *Fg* infection. For instance, the log2 values of Traes_1BS_BA046E212.1 (XII) at 1, 2 and 4 d after *Fg* infection were 0.26, 1.23 and 2.71, respectively. (2) Powdery mildew: most *T. aestivum* PRXs exhibited the same expression trend but different log2 values with the two types of treatments. For example, the log2 values of Traes_2AL_2224DCC1A.1 (VI) under Si(+) (supply of soluble silicon) *Bgt*(+) (infection of *Blumeria graminis* f. sp. *tritici*, *Bgt*) and Si(−) *Bgt*(+) treatments were both upregulated at 1.53 and 0.57, respectively. However, a few *T. aestivum* PRXs, such as Traes_4AL_0C8DFDE2B.1 (VI) and Traes_2AS_647C2FAA9.1 (I), exhibited opposite expression trends in the Si(+) *Bgt*(+) and Si(−) *Bgt*(+) treatments. (3) Blast disease: some *T. aestivum* PRXs, such as Traes_2BL_B36482127.1 (VI), Traes_4AS_9EEABCE1C.1 (VII), Traes_4DL_8CE055F15.1 (VII) and Traes_1BL_4831B07DA.1 (I), were upregulated when infected with the three types of *Magnaporthe* pathogens. Similarly, three *T. aestivum* PRXs, Traes_6DL_2A99B8CDC.1 (VII), Traes_2AL_7EABAC855.1 (VII) and Traes_2AL_0A0101B75.1 (IX), showed downregulation under these three *Magnaporthe* pathogen infections. (4) Fly larval attack: we noticed that a few *T. aestivum* PRXs, such as TRAES3BF008800130CFD_t1 (unclear_classification), Traes_7DL_CE37E2AF1.1 (V), Traes_1DS_A23800206.1 (II) and Traes_1AS_C70D49E2E.4 (VI), exhibited progressive upregulation at 6, 12 and 24 d after fly larval attack. For instance, the log2 values of Traes_1DS_A23800206.1 (II) at 6, 12 and 24 d were 0.84, 0.97 and 1.14, respectively. Similar to FHB, these PRXs might also regulate the pathway of the fly larval attack response. (5) Earthworm: most *T. aestivum* PRXs exhibited the same expression trend but different log2 values under both treatments. For instance, the log2 values of Traes_6DL_2A99B8CDC.1 (VII) were 0.04 and 0.77 in the earthworm(+) *Ggt*(+) (infection by the soil-borne fungus *Gaeumannomyces graminis* var. *tritici*, *Ggt*) and earthworm(+) *Ggt*(−) treatments, respectively. We also noticed that some PRXs, such as Traes_7DS_F64FA7C37.1 (VII) and Traes_2AS_647C2FAA9.1 (I), exhibited opposite expression trends under earthworm(+) *Ggt*(+) and earthworm(+) *Ggt*(−) treatments.

**Fig. 8:**
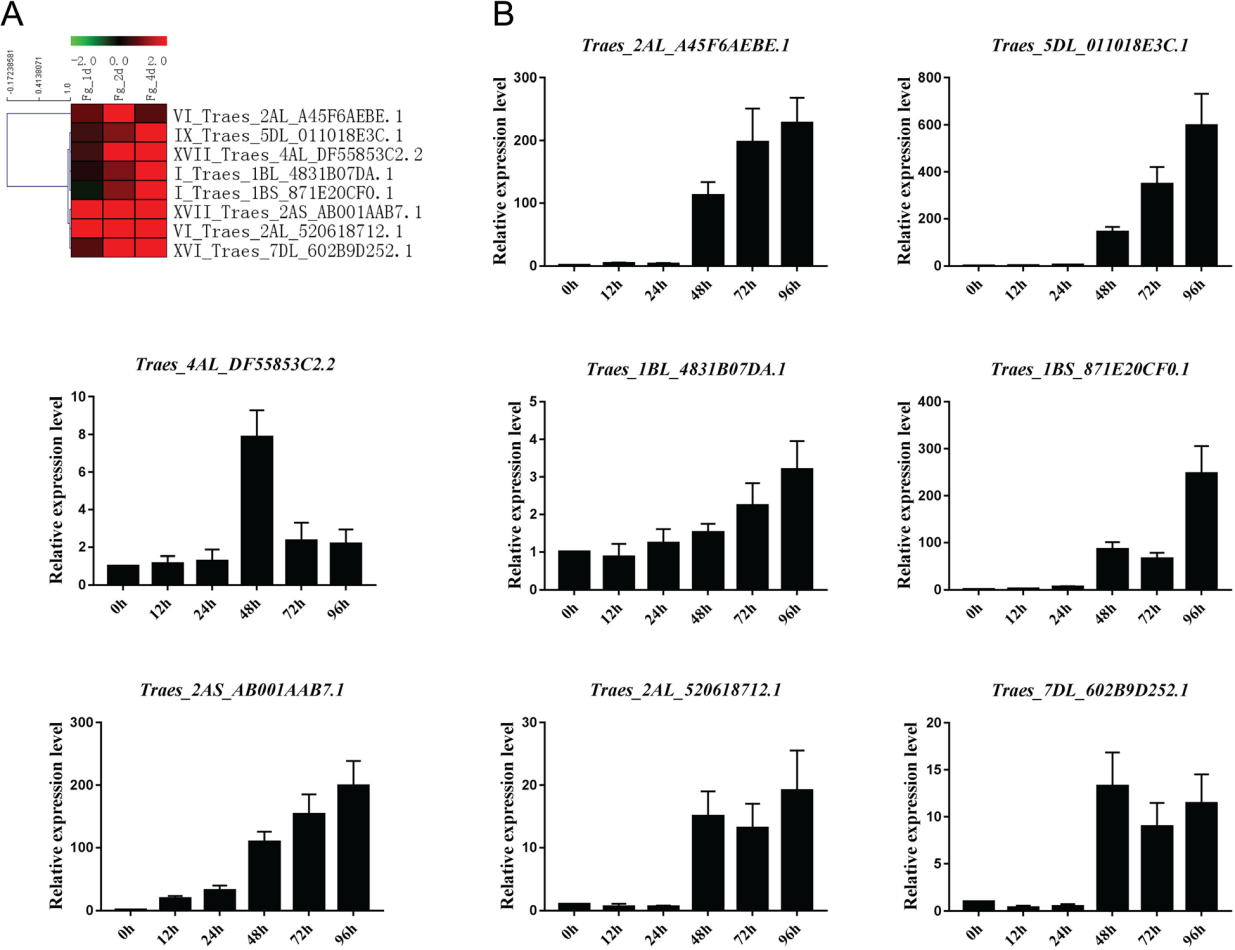
Heatmap of eight selected *T. aestivum* PRXs and their qRT-PCR results with *Fg* infection. **a** Heatmap of the microarray. **b** qRT-PCR with *Fg* infection

To confirm the FHB expression pattern of the GEO microarray, we examined eight *T. aestivum* PRXs using qRT-PCR (Fig. 8). The log2 values of these eight *T. aestivum* PRXs were all upregulated at 1, 2 and 4 d after *Fg* infection. This result was consistent with our qRT-PCR results that all exhibited upregulation at 48–96 h after *Fg* infection. Interestingly, four *T. aestivum* PRXs, Traes_2AL_A45F6AEBE.1 (VI), Traes_5DL_011018E3C.1 (IX), Traes_1BS_871E20CF0.1 (I) and Traes_2AS_AB001AAB7.1 (XVII), showed more than 100-fold upregulation at 48–96 h after *Fg* infection, hinting that they might be important for the signalling pathways of the *Fg* infection response.

## Discussion

### Evolution and duplication events of the class III PRX gene family in wheat and *Ae. tauschii*

In this study, we determined the identification, evolution and expression of the class III PRX gene family in wheat and *Ae. tauschii*. The numbers of PRX gene families in *T. urartu* (159) and *Ae. tauschii* (169) are similar to those in three investigated monocots (*B. distachyon*, 149, *O. sativa*, 125, and *Z. mays*, 156) but higher than that in two eudicots (*V. vinifera*, 85, and *A. thaliana*, 81). This is consistent with a report that maize PRXs (119) are higher in number than *Arabidopsis* (73) and poplar (93) [21]. The number of PRXs in *T. aestivum* (374) is approximately three-fold higher than that in *T. urartu* (159) and *Ae. tauschii* (169). This “three-fold” relationship is consistent with our previous article on the PK gene family in which the number of PKs in *T. aestivum* (3269) is approximately three-fold greater than that in *T. urartu* (1213) and *Ae. tauschii* (1448) [27]. This is because the origin of allohexaploid bread wheat (*T. aestivum*) was involved in three polyploidizations [26]. The examples of the “three-fold” relationship are presented in greater detail in Additional file 1: Figure S1. For instance, five sequences (Traes_7AS_81327ECA0.1, TRIUR3_11069-P1, Traes_7BS_3878BF050.1, EMT10887 and Traes_7DS_F0152FC36.1) formed a clan with a bootstrap value of 100.

To infer the evolutionary history of the PRX gene family, we also identified PRXs in eight other plants, including primitive green algae (6, *C. reinhardtii*), moss (60, *P. patens*) and fern (167, *S moellendorffii*). Our results showed that the PRX subfamily VII first appeared in *C. reinhardtii.* In 2004, Passardi et al. [19] found 11–14 putative PRXs in *P. patens* using the EST database but none in *C. reinhardtii, Thalassiosira pseudonana* or *Phytophthora sojae*. The absence of *C. reinhardtii* PRX in the Passardi report may be because of the incomplete *C. reinhardtii* genome at that time or due to use of the TBLASTN search method. The conserved exon-intron structure with exon phase “001” in the PRX domain could be found in most of the subfamilies V-XVIII, from *S. moellendorffii* to *T. aestivum*, hinting that this exon-intron structure might be important for the function of PRXs. This “001” exon-intron structure could also be found in *P. patens* (PP1S306_26V6.1), but not in *C. reinhardtii*, suggesting that the ancestral sequence of subfamilies V-XVIII might have appeared in moss-resembling ancestors*.* We did not found PRX subfamily II in two investigated eudicots (*V. vinifera* and *A. thaliana*) but found it in all investigated monocots, and its “001” exon-intron structure had changed into a “0” two-exon structure, suggesting that a monocot-specific exon fusion event occurred in the PRX subfamily II after the monocot-eudicot split.

**Fig. 9:**
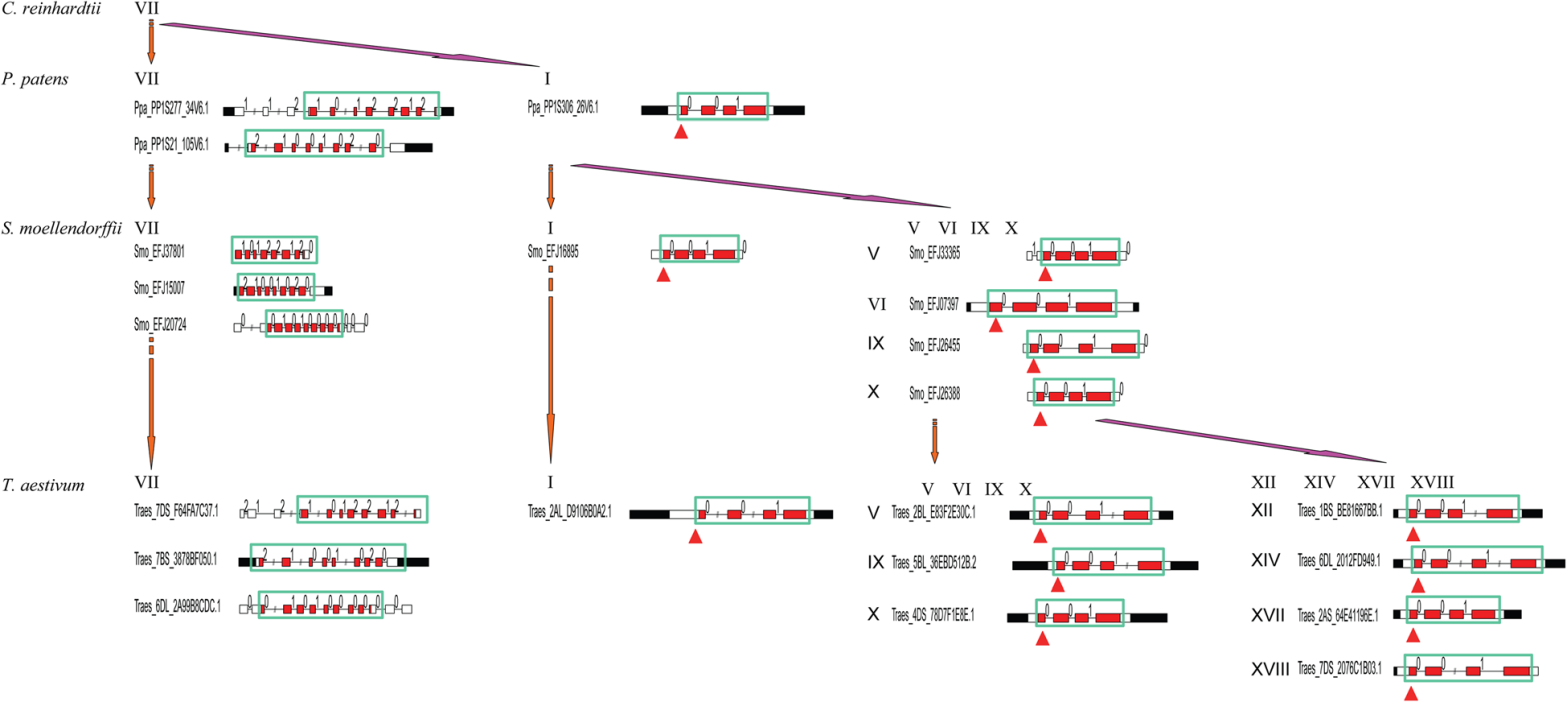
The evolutionary model of the exon-intron structure of PRX genes. This graph displays the evolution of PRX subfamilies in four representative plants (*C. reinhardtii*, *P. patens*, *S. moellendorffii* and *T. aestivum*). We found that a conserved exon-intron structure with exon phase “001” in the PRX domain was present in many PRX subfamilies of the investigated plants. These “001” structures are circled by a green box and red arrow. The descriptions of the domain and exon phases are the same as those in Fig. 2

Based on the analysis of PRXs across *C. reinhardtii* to *T. aestivum,* we proposed a model to infer the evolution of the PRX gene family (Fig. 9). First, the PRX subfamily VII might have appeared in an algae-resembling ancestor, but their exon-intron structures were algae-specific. Indeed, we cannot find the four types of exon-intron structures of the *C. reinhardtii* subfamily VII PRXs in other investigated plants. Second, PRX subfamilies VII and I might appear in a moss-resembling ancestor. Two types of conserved exon-intron structures (“1012–212” and “2100–1020”) appeared in moss PRX subfamily VII, and they were conserved from *P. patens* to *T. aestivum*. The conserved exon-intron structure within exon phase “0101–0000” appeared in *S. moellendorffii* PRX subfamily VII, and it was conserved from *S. moellendorffii* to *T. aestivum*. In contrast to the abundant exon-intron structures of subfamily VII, PRX subfamily I contained four sample conserved exon-intron structures (“001”, “01”, “0” and “00”). Third, except for subfamilies VII and I, the new PRX subfamilies V, VI, IX and X appeared in *S. moellendorffii,* but their exon-intron structures were all “001”. Therefore, we inferred that PRX subfamilies V, VI, IX and X might originate in subfamily I. Interestingly, subfamilies XII, XIV-XVIII and II appeared in eudicots and monocots, but most of their exon-intron structures were still “001”. In summary, most PRX subfamilies contained conserved exon-intron structure “001”, which might originate in subfamily I.

It was reported that both segmental and tandem duplication contributed to the expansion of maize PRXs [21], while segmental duplication mainly contributed to the expansion of Chinese pear PRXs [22]. In this study, we determined 46 *T. aestivum* PRX collinearity events contributed by segmental duplication (Additional file 16: Table S4), and there were 56 tandem *T. aestivum* PRXs (Additional file 18: Table S6), suggesting that both segmental and tandem duplication contributed to the expansion of *T. aestivum* PRXs. The number of orthologous genes (73) between *T. aestivum* and *B. distachyon* is more than that (63) between *T. aestivum* and rice (Additional file 16: Table S4), suggesting that the split between *T. aestivum* and *B. distachyon* progenitors occurred after rice diverged from the common ancestor of *T. aestivum* and *B. distachyon*. Indeed, it was reported that *Triticeae* and *Brachypodieae* are sister clades and that *Oryza* is located on the root of the cluster containing six sister clades (*Diarrheneae*, *Brachypodieae*, *Poeae*, *Aveneae*, *Bromeae* and *Triticeae*) [28].

### Biological functions and expression patterns of the class III PRX gene family in wheat and *Ae. tauschii*

Aside from the catalytic function of reducing hydrogen peroxide to water, class III PRXs are involved in various other biological functions, including lignification, defence, development and germination [29]. Cosio et al. summarized the research methods (microarrays, transgenic plants, proteomics, and so on), expressed organs, and studied mechanisms (low oxygen response, aluminium stress, cold-inducible tolerance, etc.) of almost all 73 published *A. thaliana* PRXs [30]. In our results, we examined the expression patterns of *T. aestivum* PRXs under drought conditions and phytohormone treatments using public microarray datasets and qRT-PCR.

Some papers reported that a few PRXs were involved in root and stresses. Three plasma membrane-bound class III PRXs, namely, pmPOX1, pmPOX2b and pmPOX3, were purified in maize root and then identified by ESI-MS/MS and MALDI-TOF MS [31]. The protein levels of four maize root PRXs, pmPOX1, pmPOX2a, pmPOX2b, and pmPOX3, changed in response to various stresses, including H_2_O_2_, SA, wounding, MeJA, *Fg* infection, *Fusarium culmorum* infection, chitosan and cantharidin [32]. In our study, we also analysed the *T. aestivum* PRX expression patterns in root tissue (Additional file 6: Figure S6, Additional file 20: Table S8). We noticed that some *T. aestivum* PRXs, such as Traes_7DL_6233C6F03.1 (I), Traes_1AL_38F9A30EA.1 (I) and Traes_1AL_91E56EC8C.1 (V), exhibited relatively higher expression levels in two root tissues (root in the germinating seed stage and root in the seedling stage) than in other tissues, suggesting that they might play roles in root development or metabolic processes.

Some published articles about the abiotic stress responses of PRXs are consistent with our microarray and qRT-PCR results. (1) Drought: the transcript levels of selected *T. aestivum* PRXs were determined under PEG6000 in two wheat cultivars (“Plainsman V”, drought-tolerant; and “Cappelle Desprez”, drought sensitive) [33]. The results showed that the *Ta*Prx04 transcript was enhanced in the root apex of “Plainsman V” but decreased in “Cappelle Desprez”. In our drought microarray results, we also found similar *T. aestivum* PRXs (Traes_1DS_578ADDF80.1, Traes_2AS_3161D54F8.1, etc.) with reverse expression trends in two wheat cultivars (drought-susceptible “WL711” and drought-tolerant “C306”). The results showed that *Ta*Prx01, *Ta*Prx03, *Ta*Prx19, *Ta*Prx68, *Ta*Prx107 and *Ta*Prx109-C decreased in both cultivars. In our drought microarray results, we also found similar *T. aestivum* PRXs (Traes_2AS_647C2FAA9.1, Traes_2BL_E8A65526C.1, etc.) with downregulation in both “WL711” and “C306”. (2) Hormones: we compared the four published maize PRXs [21] to our homologous *T. aestivum* PRXs by using qRT-PCR under SA treatment (Additional file 11: Figure S11). The results showed that these four homologous gene pairs exhibited similar expression trends, but their peaks of upregulation did not occur at the same time after SA treatment.

Pathogen attack in plants leads to three defence mechanisms (cell wall lignification, production of antimicrobial metabolites, and production of ROS and RNS (reactive nitrogen species), which involve PRXs [34]. In this study, we also checked expression patterns under *Fg* infection using public microarray datasets and qRT-PCR. All eight investigated *T. aestivum* PRXs were upregulated in qRT-PCR and microarray results, suggesting that they might participate in the response to *Fg* infection. Additionally, our qRT-PCR results showed that four *T. aestivum* PRXs (Traes_2AL_A45F6AEBE.1, Traes_5DL_011018E3C.1, Traes_1BS_871E20CF0.1 and Traes_2AS_AB001AAB7.1) were strongly upregulated (more than 100-fold) after *Fg* infection, suggesting that they play a central role in the FHB stress regulatory network. How do plant PRXs function in pathogen defence? A novel *Marsdenia megalantha* PRX was purified and shown to inhibit the phytopathogenic fungi *Fusarium oxysporum* and *Fusarium solani* through a cell membrane permeabilization mechanism [35].

In 2018, Lüthje et al. summarized the physiological functions (such as senescence, floral organ development, lignification of vessels, drought, cold and pathogen) of membrane-bound class III PRXs in *Arabidopsis*, barrel medic, rice and maize [36]. In this article, we selected eleven maize PRXs involved in drought or pathogen stress response and compared them with our predicted microarray results in *T. aestivum* PRXs by BLAST. Among them, only six maize PRXs had homologous probes in our microarray. However, five maize PRXs had the same physiological function as the homologous *T. aestivum* PRX (Additional file 21: Table S9). (1) Drought: *T. aestivum* PRXs (Traes_2BS_990895438.1 and Traes_6AS_ADF96853B.1), which are homologous to *ZmaPrx70* and *ZmaPrx122* (A5H452 and A0A1D6H652), showed slight upregulation in “C306” (drought-tolerant) under drought conditions. (2) Pathogen: *T. aestivum* PRXs (Traes_2BS_430425C78.1 and Traes_2AL_C4CDAA081.1), which are homologous to *ZmaPrx56* and *ZmaPrx85* (A0A1D6IMZ0 and A0A1D6E530), showed progressive upregulation at 1–4 d after *Fg* infection. (3) Drought and pathogen: *T. aestivum* PRX (Traes_2DS_D76AB139C.1), which is homologous to *ZmaPrx114* (C0PPB6), showed upregulation under drought and *Fg* stresses. Our qRT-PCR results also showed that Traes_2DS_D76AB139C.1 exhibited upregulation in “Sumai-3” at 6 h after PEG treatment (Fig. 6).

## Conclusions

In this study, we performed genome-wide identification and classification of class III PRXs in wheat, *Ae. tauschii* and eight other representative plants. To infer PRX evolution, the exon-intron structures of PRXs were diagrammed in these eleven plants. Some conserved exon-intron structures with conserved exon phases in the PRX domain were found across species from *P. patens* to *T. aestivum*. Based on our analysis, an evolutionary model of exon-intron structures of PRX genes was proposed in which subfamily VII could be the ancient subfamily, and most subfamily V-XVIII PRXs contained the conserved exon-intron structure “001”, which might origin in subfamily I. WGD, TD and syntenic analysis were performed with *T. aestivum, B. distachyon* and *O. sativa* PRXs. The results showed that both WGD and TD contributed to the expansion of *T. aestivum* PRXs. Global expression pattern analysis using public microarray datasets revealed that some PRXs could be involved in biotic and abiotic responses in wheat. qRT-PCR of selected *T. aestivum* PRXs under PEG, phytohormone and *Fg* treatments validated the microarray predictions. The confocal microscopy results indicating the subcellular localization of *Tae*PRXs from different subfamilies were consistent with the website predictions. Sequencing promoters validated the predicted hormone responsive *cis*-elements. Our results will provide clues for researchers regarding the evolution and biological functions of PRXs.

## Methods

### Identification and classification of class III PRXs in wheat, *Ae. tauschii* and other plants

The genomes and proteomes of *T. aestivum* were downloaded from Ensembl Plants, release-31 (http://plants.ensembl.org/). The genomes and proteomes of ten other plants, including *T. urartu, Ae. tauschii*, *B. distachyon*, *Z. mays*, *O. sativa*, *A. thaliana*, *V. vinifera, S. moellendorffii*, *P. patens* and *C. reinhardtii*, were downloaded from Ensembl Plants, release-33. To identify the PRXs, the proteomes of all eleven plants were scanned by our local server HMMER3.1 (Pfam profile PF00141.21, peroxidase.hmm, PRX domain) and the website Pfam 30.0 (http://pfam.xfam.org/) in batch mode with an E value of 0.01. Atypical PRXs with a PRX domain covering less than 50% alignment were excluded in the following analysis. The PRX alignment of *T. aestivum, T. urartu* and *Ae. tauschii* truncated sequences in the PRX domain was performed by ClustalW v2.0 [37]. The NJ phylogenetic tree was constructed by MEGA-CC 7.0 [38] with a p-distance model and 1000 bootstrap repetitions in our local server. Similarly, the large NJ phylogenetic tree of these eleven plants was also constructed by ClustalW v2.0 and MEGA-CC 7.0. The classification of PRX subfamilies was performed by HMMER3.1, and models were generated based on the maize PRX alignments [21].

### Domain and exon-intron structure diagram of PRXs

The domain and exon-intron structures of PRXs in these eleven plants were generated by our Perl and R scripts based on the corresponding GFF file information from Ensembl Plants (http://plants.ensembl.org/). The domain information was batched from Pfam 30.0 (http://pfam.xfam.org/).

### Chromosome locations, duplication events and synthetic analysis of wheat PRXs

Based on the extracted information in GFF files from Ensembl Plants (http://plants.ensembl.org/), the chromosome locations of *T. aestivum* PRXs were diagrammed using Mapchart v2.3 (https://www.wur.nl/en/show/mapchart.htm). BLASTP was performed against PRXs of *T. aestivum*, *B. distachyon* and *O. sativa* with an E value of e-100. Based on the GFF and BLAST results, tandem duplication and segmental duplication were searched using MCScanX [39]. The *Ka* and *Ks* values were calculated by “add_ka_and_ks_to_collinearity.pl” in MCScanX. To search for the synthetic relations between the *T. aestivum* A subgenome and *T. urartu*, between the *T. aestivum* D subgenome and *Ae. tauschii*, BLAST was performed with an E value of e-100. Then, the *Ka* and *Ks* values were calculated by KaKs_Calculator2.0 [40] with the γ-YN method.

### Microarray expression data analysis

Public wheat microarray expression data was downloaded from the GEO database of NCBI. Microarray datasets of tissues and stress treatments were selected from the Affymetrix Wheat Genome Array platform GPL3802. (1) Tissues: GSE12508, thirteen tissues at defined developmental stages for wild-type wheat (cultivar Chinese Spring) [41]. (2) Abiotic stresses: cold: GSE14697, two wheat lines, freeze resistant and freeze susceptible, were compared with and without 4 °C treatment [42]; heat: GSE60351, flag leaves of the wheat cultivar “TAM 107” were sampled after 1 h of heat stress (40 °C) treatment; drought: GSE87325, leaf tissues of two wheat genotypes (drought-susceptible variety “WL711” and drought-tolerant variety “C306”) were collected for Affymetrix microarrays under drought stress; nutrient deficiency: GSE61679, the root tissues of wheat cultivar “Hereward” under five nutrient-deficient conditions; phytohormones: GSE103430, wheat spike tissues exposed to seven phytohormones, including IAA, GA (GA3), ABA, ET, CK (trans-zeatin), SA and MeJA. (3) biotic stresses: Fusarium head blight: GSE36283, wheat spikelets from the very susceptible spring wheat cultivar “Roblin” at 1, 2 and 4 d after *Fg* infection [43]; powdery mildew: GSE12936, wheat cultivar “AC Drummond” under soluble silicon (Si) and pathogen stress (*B. graminis* f.sp. *tritici*, *Bgt*) [44]; blast fungus: GSE31760, wheat cultivar “Renan” infected by three *Magnaporthe* pathogen isolates (non-adapted BR29, adapted BR32 and BR37) [45]; Hessian fly larvae: GSE34445, two wheat lines, “Molly” (containing R gene H13, resistance) and “Newton” (susceptible), were collected at 6, 12 and 24 h after Hessian fly egg hatching [46]; earthworms: GSE47479, wheat was inoculated with the soil-borne fungus *G. graminis* var. *tritici* (*Ggt*) and earthworms. Quality control and normalization of raw data were performed by RMAexpress v1.2.0 (http://www.rmaexpress.bmbolstad.com/). The mean expression levels of tissues were calculated using our Perl script. The expression levels (log2 value of fold change, treatment vs. control) of *T. aestivum* PRXs under stress treatments were calculated by R software and the R package limma. Heat maps of *T. aestivum* PRX expression levels were generated by Mev4.9 [47].

### Plant material and stress treatments

Wheat (*T. aestivum* L.) cultivar “Sumai-3” seeds were germinated on damp filter paper at room temperature for approximately 24 h. The mesocotyls grew to approximately 2–3 mm in length, and then the seedlings were transferred into pots for growth in a greenhouse at 20–25 °C with a photoperiod of 16 h/8 h. The seedlings with two leaves were used in all experiments unless stated otherwise. Then, the seedlings were subjected to drought stress (20% (m/V) PEG-6000) for 0, 6, 12, 24, 48 and 72 h. The seedlings were also treated with four phytohormones, 1.5 mM SA, 100 μM MeJA, 100 μM IAA and 100 μM ABA, for 0, 1, 3, 6, 12, 24 and 48 h. The spikelets of “Sumai-3” in the flowering stage were inoculated with *Fg* for 0, 12, 24, 48, 72 and 96 h, respectively. Leaves (drought and phytohormones) and spikelets (*Fg*) were separately collected and immediately frozen in liquid nitrogen and then stored at − 80 °C for qRT-PCR. At least 30 samples of each experimental replicate were analysed with different treatments.

### RNA extraction and qRT-PCR

Total RNA was isolated using the TRIzol kit (TransGen Biotech Co., Ltd., Beijing, China). The first-strand cDNA was synthesized with oligo-dT primers using TransScript First-Strand cDNA Synthesis Supermix (TransGen Biotech Co., Ltd., Beijing, China). qRT-PCR was performed in a 20-μl reaction volume using a Roche LightCycler® 480 (Roche Diagnostics GmbH, Mannheim, Germany) for three biological replicates. Wheat β*-Actin* was used as an internal reference. Relative mRNA levels were calculated using the 2 ^−ΔΔCT^ method. The qRT-PCR primers for PEG, four phytohormones and FHB are supplied in Additional file 26: Table S14.

### Subcellular localization of *Tae*PRXs

The coding region of *Tae*PRXs was amplified and fused to the N-terminus of eGFP, which was driven by the CaMV 35S promoter to generate pBIN35S-*Tae*PRX-eGFP. At the same time, we used a 35S:eGFP fusion construct as a control. The constructs were used for subcellular localization analysis. The positive clones were transformed into *Agrobacterium* EHA105. The resulting *Agrobacterium* culture was resuspended in an infiltration medium [10 mM 4-morpholineethanesulfonic acid hydrate (MES) (pH 5.6), 10 mM MgCl_2_, and 200 mM acetosyringone) and then injected into four-week-old tobacco (*Nicotiana benthamiana*) leaves at an OD600 of 0.6. Transformed tobacco leaves were imaged using a confocal microscope (PERKINEIMER UITRAVIEW VOX Confocal Microscope). The *Tae*PRXs cloning experiment was performed in leaves of the wheat cultivar “Sumai-3”. Subcellular localization of the primers for *Tae*PRXs are supplied in Additional file 27: Table S15. Predicted subcellular localization of *Tae*PRXs was performed in WoLF PSORT (https://www.genscript.com/wolf-psort.html?src=leftbar) and TargetP (http://www.cbs.dtu.dk/services/TargetP/).

### Predicted *cis*-acting elements and sequencing validation

For promoter analysis, 2 kb upstream region from the translation start codon ATG of each *Tae*PRX was truncated by our Perl scripts, and then predicted *cis*-acting elements in the (+) strand and (−) strand of each promoter of *Tae*PRXs were found using the PlantCARE42 database (http://bioinformatics.psb.ugent.be/webtools/plantcare/html/). To validate these predicted *cis*-acting elements, total DNA was isolated, and then PCR was performed to amply 2-kb upstream promoter sequences. Gel extraction and sequencing validation were performed. The *Tae*PRX promoter cloning experiment was performed in leaves of the wheat cultivar “Sumai-3”. Primers used to clone the promoters related to predicted *cis*-elements are supplied in Additional file 28: Table S16.

## Additional files

Additional file 1: **Figure S1.** Expanded phylogenetic classification of class III peroxidases using the neighbour-joining method. (a) *T. aestivum*, *T. urartu* and *Ae. tauschii*; (b) All investigated plants. (PDF 376 kb)

Additional file 2: **Figure S2.** Exon−intron and domain diagrams of class III peroxidases in *T. aestivum, T. urartu, Ae. tauschii*, *B. distachyon*, *Z. mays*, *O. sativa*, *A. thaliana*, *V. vinifera, S. moellendorffii*, *P. patens* and *C. reinhardtii*. The descriptions of the domain and exon phases are the same as those in Fig. 2. The lengths of the boxes and lines are scaled based on the lengths of the genes. (PDF 487 kb)

Additional file 3: **Figure S3.** Conserved exon−intron and domain diagrams of class III peroxidases in *T. aestivum, B. distachyon*, *S. moellendorffii* and *P. patens.* The descriptions of the domain and exon phases are the same as those in Fig. 2. The lengths of the boxes and lines are scaled based on the lengths of the genes. (PDF 30 kb)

Additional file 4: **Figure S4.** Chromosome locations of class III peroxidases in *T. aestivum*. (PDF 49 kb)

Additional file 5: **Figure S5.** Quality control of GEO microarray datasets. RLE (Relative log expression) and NUSE (Normalized unscaled standard errors) values of each GEO microarray dataset. (PDF 2638 kb)

Additional file 6: **Figure S6.** Heatmap of the expression patterns of *T. aestivum* class III peroxidase genes in different tissues. The expression patterns of 170 class III peroxidase genes in different tissues: coleoptile, root and embryo of germinating seed; root, crown and leaf of seedling; immature inflorescence; floral bracts, pistil and anthers before anthesis; 3-5 DAP (day after planting) caryopsis; 22 DAP embryos. The heatmap was generated using MeV (Multiple Experiment Viewer) software, version 4.9. Red and green correspond to upregulation and downregulation, respectively. Normalized gene expression values and *p* values are provided in Additional file 20: Table S8. (PDF 1350 kb)

Additional file 7: **Figure S7.** Heatmap of the expression patterns of *T. aestivum* class III peroxidase genes under abiotic stress treatments. The expression patterns of 170 class III peroxidase genes under abiotic stress treatments (cold, heat, drought and nutrient deficiency) are presented. Normalized gene expression values are provided in Additional file 21: Table S9. (PDF 1333 kb)

Additional file 8: **Figure S8.** Heatmap of the expression patterns of *T. aestivum* class III peroxidase genes under seven phytohormone treatments. The expression patterns of 170 class III peroxidase genes under seven phytohormone treatments, including IAA, GA (GA3), ABA, ET, CK (trans-zeatin), SA and MeJA, are presented. Normalized gene expression values are provided in Additional file 21: Table S9. (PDF 1226 kb)

Additional file 9: **Figure S9.** Upstream sequences and predicted *cis*-acting elements related to stress and hormone responses in *Tae*PRXs. Various predicted *cis*-acting elements are circled by different colours. The corresponding information is provided in Additional file 23: Table S11. (PDF 6070 kb)

Additional file 10: **Figure S10.** Sequencing data of the predicted *c* is-elements in upstream. Pair alignments were performed between upstream sequences and sequencing data. Matched *cis*-acting elements between them are circled by red boxes. *Cis*-acting elements with tiny differences are circled by green boxes. (PDF 1069 kb)

Additional file 11: **Figure S11.** qRT-PCR of four *T. aestivum* PRXs under four phytohormone treatments and homologous maize PRXs under SA treatment. (A) qRT-PCR of *T. aestivum* PRXs under SA, JA, IAA and ABA treatments. (B) Homologous maize PRXs under SA treatment. (PDF 912 kb)

Additional file 12: **Figure S12.** Heatmap of the expression patterns of *T. aestivum* class III peroxidase genes under biotic stress treatments. The expression patterns of 170 class III peroxidase genes under biotic stress treatments (Fusarium head blight, powdery mildew, blast fungus, Hessian fly larvae and earthworms) are presented. Normalized gene expression values are provided in Additional file 24: Table S12. (PDF 1418 kb)

Additional file 13: **Table S1.** Subfamily classification of class III peroxidases in the investigated plant genomes. (XLS 168 kb)

Additional file 14: **Table S2.** List of atypical class III peroxidases in the investigated plant genomes. (XLS 87 kb)

Additional file 15: **Table S3.** Chromosome locations of *T. aestivum* class III peroxidases. (XLS 64 kb)

Additional file 16: **Table S4.** Collinearity events and *Ka/Ks* values of class III peroxidases among *T. aestivum*, *B. distachyon* and *O. sativa*. Sheet 1 was *Ka/Ks* values of collinearity events in *T. aestivum* class III peroxidases*;* sheet 2 was *Ka/Ks* values of collinearity events in all *T. aestivum* genes. Similarly, sheets 3-4 showed *T. aestivum* and *B. distachyon*. Sheets 5-6 showed *T. aestivum* and *O. sativa*. (XLS 5739 kb)

Additional file 17: **Table S5.** Collinearity events and *Ka/Ks* values of class III peroxidases in *T. aestivum* subgenomes, *Ae. tauschii* and *T. urartu*. Sheet 1 was *Ka/Ks* values of class III peroxidase collinearity events in the *T. aestivum* A-subgenome and *T. urartu*. Similarly, sheet 2 showed the *T. aestivum* D-subgenome and *Ae. tauschii*. (XLS 58 kb)

Additional file 18: **Table S6.** Chromosome locations of tandemly arrayed *T. aestivum* class III peroxidases. (XLS 34 kb)

Additional file 19: **Table S7.** Public wheat expression data. (XLS 48 kb)

Additional file 20: **Table S8.** Normalized gene expression values of 170 *T. aestivum* class III peroxidase genes in different tissues. (XLS 72 kb)

Additional file 21: **Table S9.** Normalized gene expression values of 170 *T. aestivum* class III peroxidase genes under abiotic stress treatments. (XLS 138 kb)

Additional file 22: **Table S10.** Differentially expressed (*p*<0.01 and |FC|>1.5) *T. aestivum* class III peroxidase genes under abiotic stress treatments. (XLS 47 kb)

Additional file 23: **Table S11.** Putative *cis*-acting elements related to stress and hormone responses in *Tae*PRXs promoters. (XLS 39 kb)

Additional file 24: **Table S12.** Normalized gene expression values of 170 *T. aestivum* class III peroxidase genes under biotic stress treatments. (XLS 111 kb)

Additional file 25: **Table S13.** Differentially expressed (p<0.01 and |FC|>1.5) *T. aestivum* class III peroxidase genes under biotic stress treatments. (XLS 38 kb)

Additional file 26: **Table S14.** Primers used in qRT-PCR analysis. (XLSX 40 kb)

Additional file 27: **Table S15.** Primers of *Tae*PRXs used for subcellular localization. (XLS 20 kb)

Additional file 28: **Table S16.** Primers used to clone the promoters related to predicted *cis*-elements. (XLS 20 kb)

## Data Availability

The genomes, proteomes and GFF files of the investigated plants are available in Ensembl Plants (http://plants.ensembl.org/). The accession numbers of plants are *T. aestivum* (IWGSC1 + popseq.31), *Ae. tauschii* (ASM34733v1), *A. thaliana* (TAIR10), *B. distachyon* (v1.0), *O. sativa* (IRGSP-1.0), *P. patens* (ASM242v1), *S. moellendorffii* (v1.0), *T. urartu* (ASM34745v1), *V. vinifera* (IGGP_12x), and *Z. mays* (AGPv4). Public wheat microarray expression data are available in the GEO database of NCBI. The accession numbers of microarrays are GSE12508, GSE14697, GSE60351, GSE87325, GSE61679, GSE103430, GSE36283, GSE12936, GSE31760, GSE34445 and GSE47479.

## Abbreviations

ABA: Abscisic acid
CK: Trans-zeatin, one kind of cytokinins
DAP: Day after planting
EST: Expressed Sequence Tag
ET: Ethylene
FC: Fold change
FHB: Fusarium head blight
GA: Gibberellic acid
GEO: Gene expression omnibus
GFP: Green fluorescent protein
GST: Glutathione-S-transferase
HMM: The hidden Markov model
IAA: Indole-3-acetic acid
JA: Jasmonic acid
*Ka*: Non-synonymous substitution
*Ks*: Synonymous substitution
MeJA: Methyljasmonic acid
NJ: Neighbour-joining
NUSE: Normalized unscaled standard errors
PEG: Polyethylene glycol
PK: Protein kinase
PRX: Peroxidase
qRT-PCR: Quantitative real-time PCR
RLE: Relative log expression
RNS: Reactive nitrogen species
ROS: Reactive oxygen species
SA: Salicylic acid
SNP: Single nucleotide polymorphism
SOD: Superoxide dismutase
*Tae*PRXs: *T. aestivum* PRXs
TD: Tandem duplication
WGD: Whole genome duplication
