# Supplementary material for: Genome-wide and evolutionary analysis of the class III peroxidase gene family in wheat and Aegilops tauschii reveals that some members are involved in stress responses
Source: BMC Genomics. 2019 Aug 22;20:666. doi: 10.1186/s12864-019-6006-5 (PMC6704529; doi:10.1186/s12864-019-6006-5)

# T.ae class III peroxidase I subfamily exon−intron and prx domain diagram (part 1)

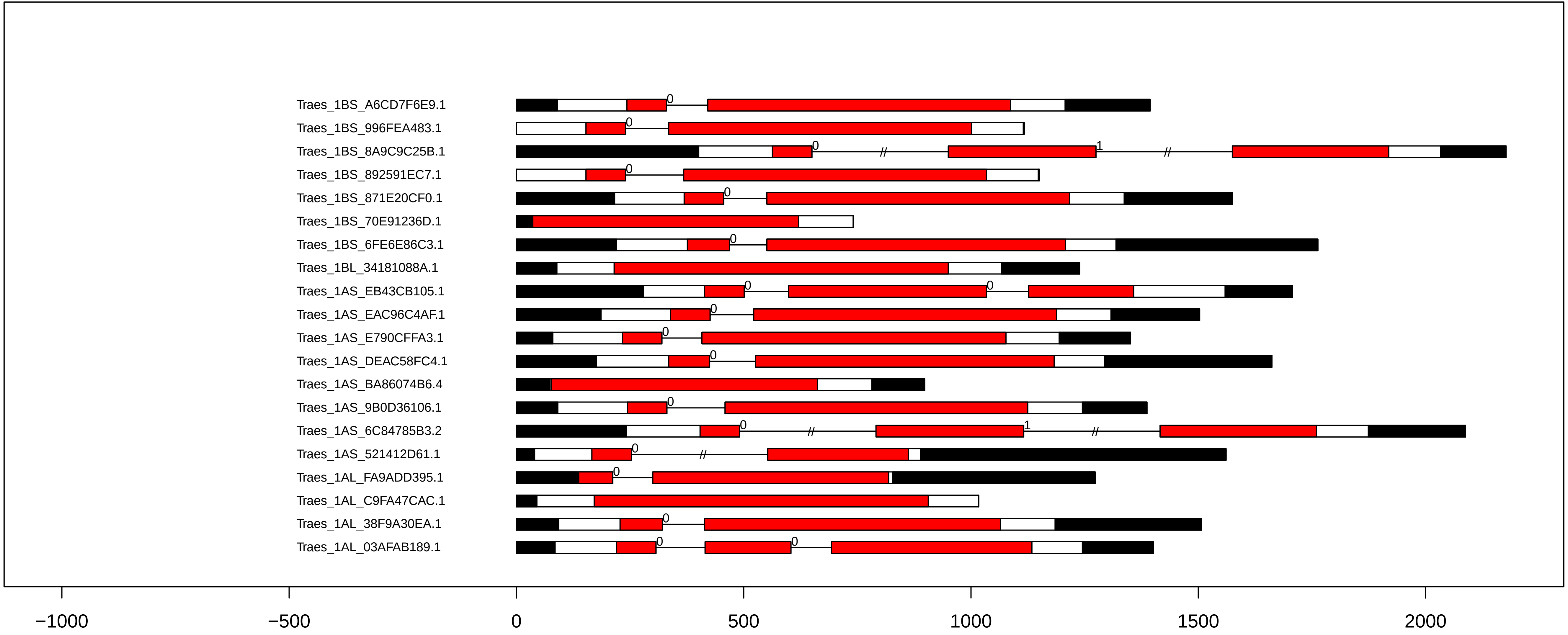

T.ae class III peroxidase I subfamily exon−intron and prx domain diagram (part 2)

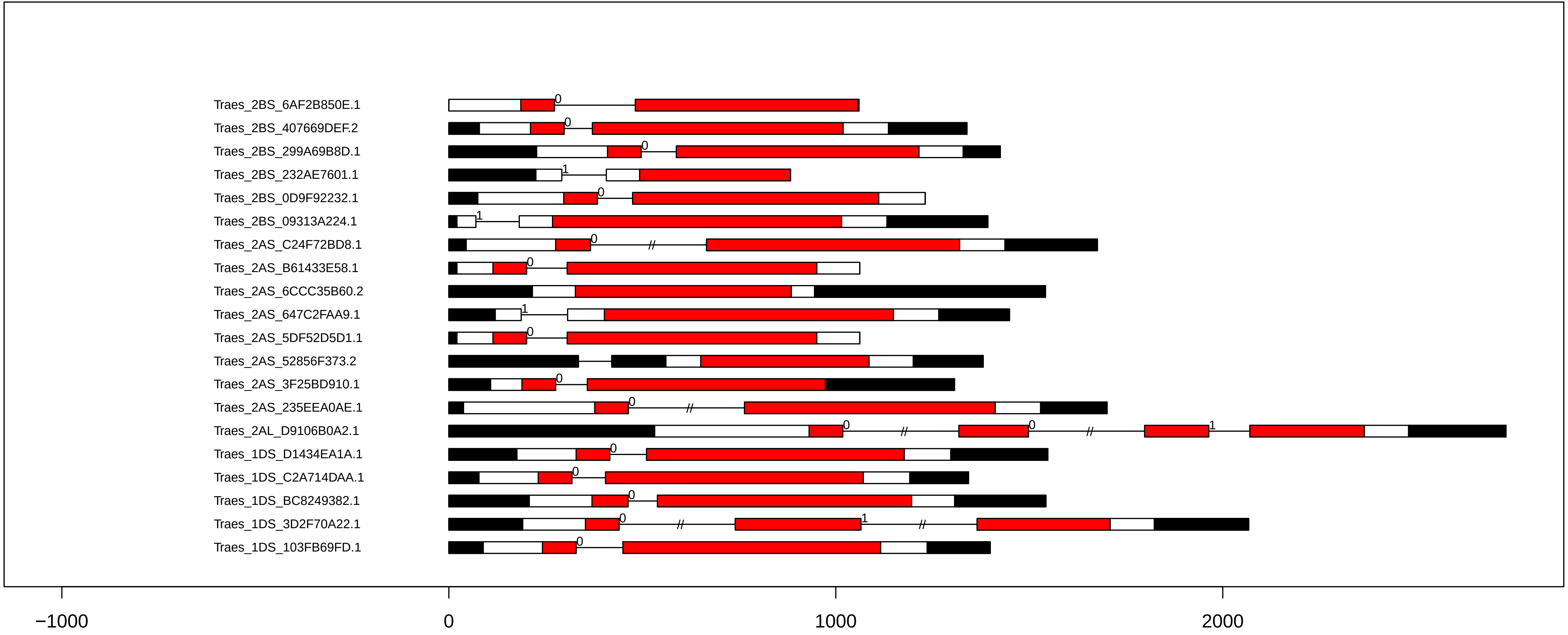

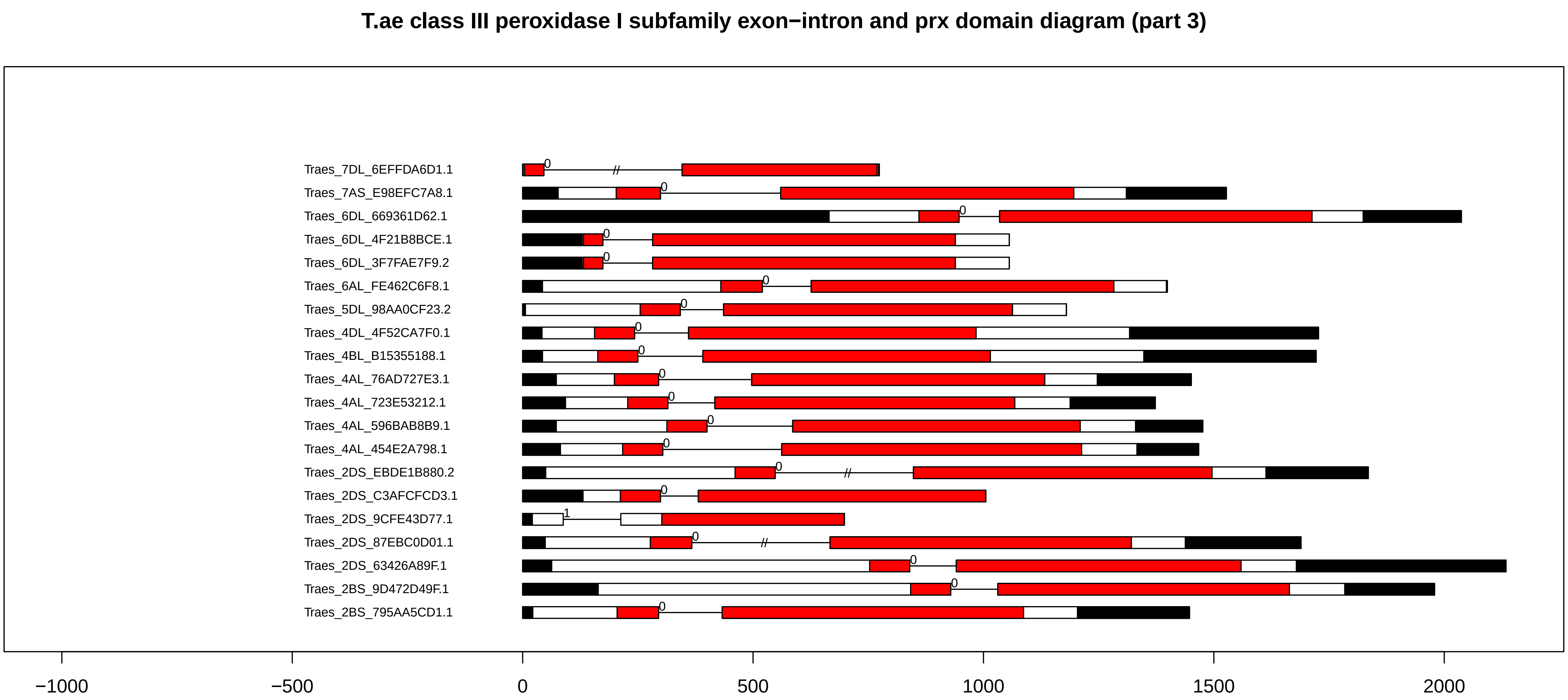
T.ae class III peroxidase I subfamily exon−intron and prx domain diagram (part 3)
Traes_7DL_6EFFDA6D1.1
Traes_7AS_E98EFC7A8.1
Traes_6DL_669361D62.1
Traes_6DL_4F21B8BCE.1
Traes_6DL_3F7FAE7F9.2
Traes_6AL_FE462C6F8.1
Traes_5DL_98AA0CF23.2
Traes_4DL_4F52CA7F0.1
Traes_4BL_B15355188.1
Traes_4AL_76AD727E3.1
Traes_4AL_723E53212.1
Traes_4AL_596BAB8B9.1
Traes_4AL_454E2A798.1
Traes_2DS_EBDE1B880.2
Traes_2DS_C3AFCFCD3.1
Traes_2DS_9CFE43D77.1
Traes_2DS_87EBC0D01.1
Traes_2DS_63426A89F.1
Traes_2BS_9D472D49F.1
Traes_2BS_795AA5CD1.1
−1000
−500
0
500
1000
1500
2000

# T.ae class III peroxidase I subfamily exon−intron and prx domain diagram (part 4)

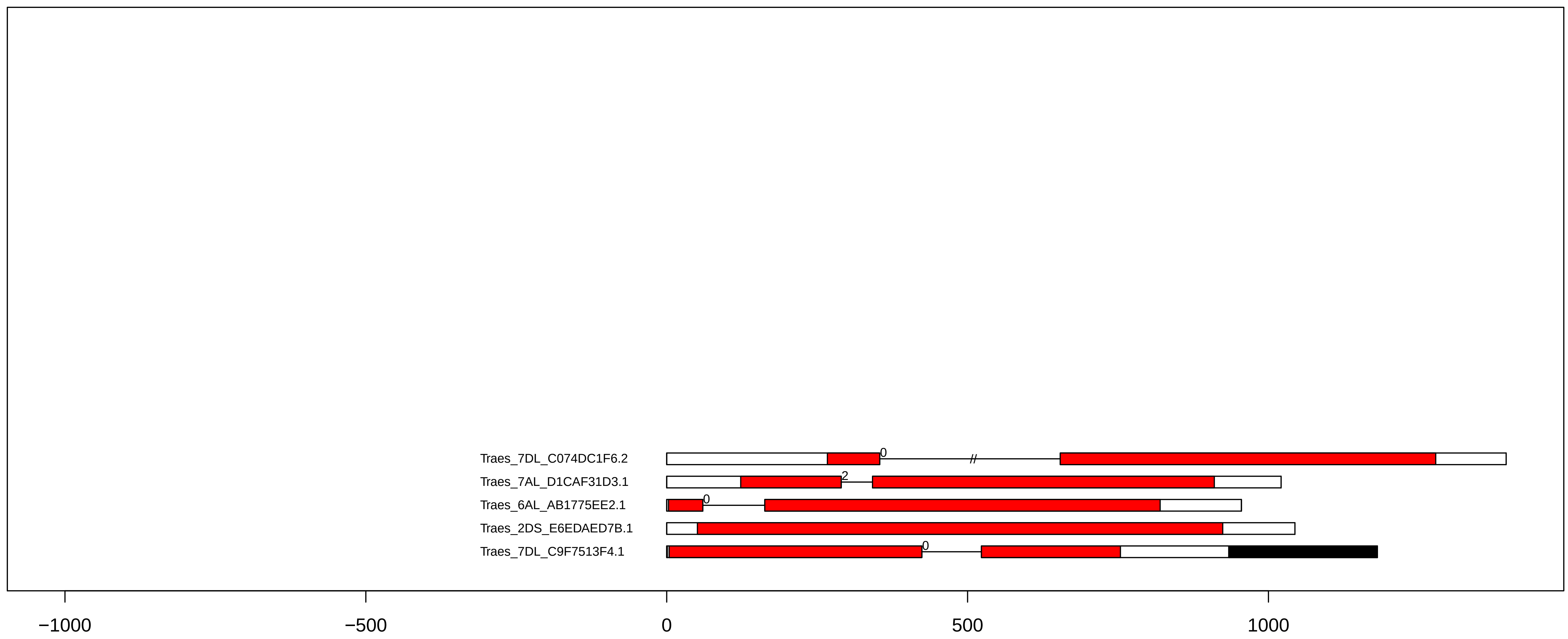

# T.ae class III peroxidase II subfamily exon−intron and prx domain diagram (all)

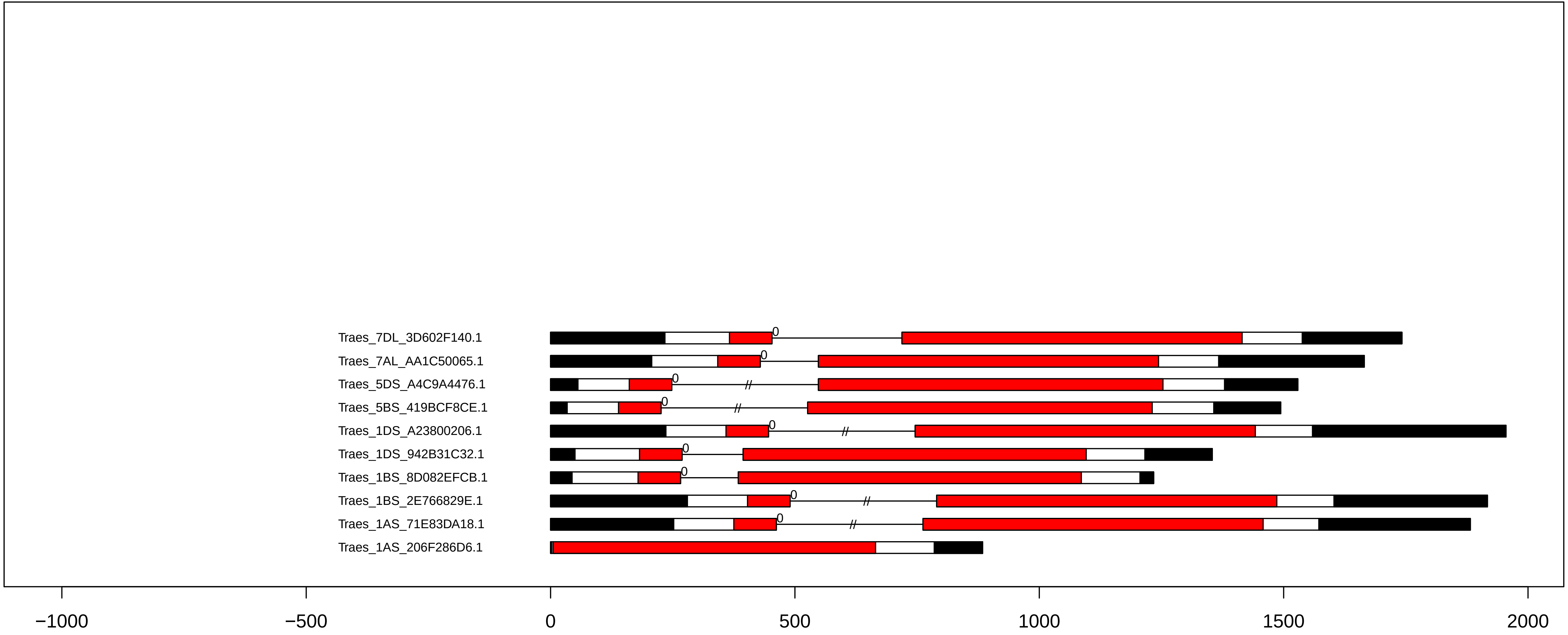

# T.ae class III peroxidase V subfamily exon−intron and prx domain diagram (part 1)

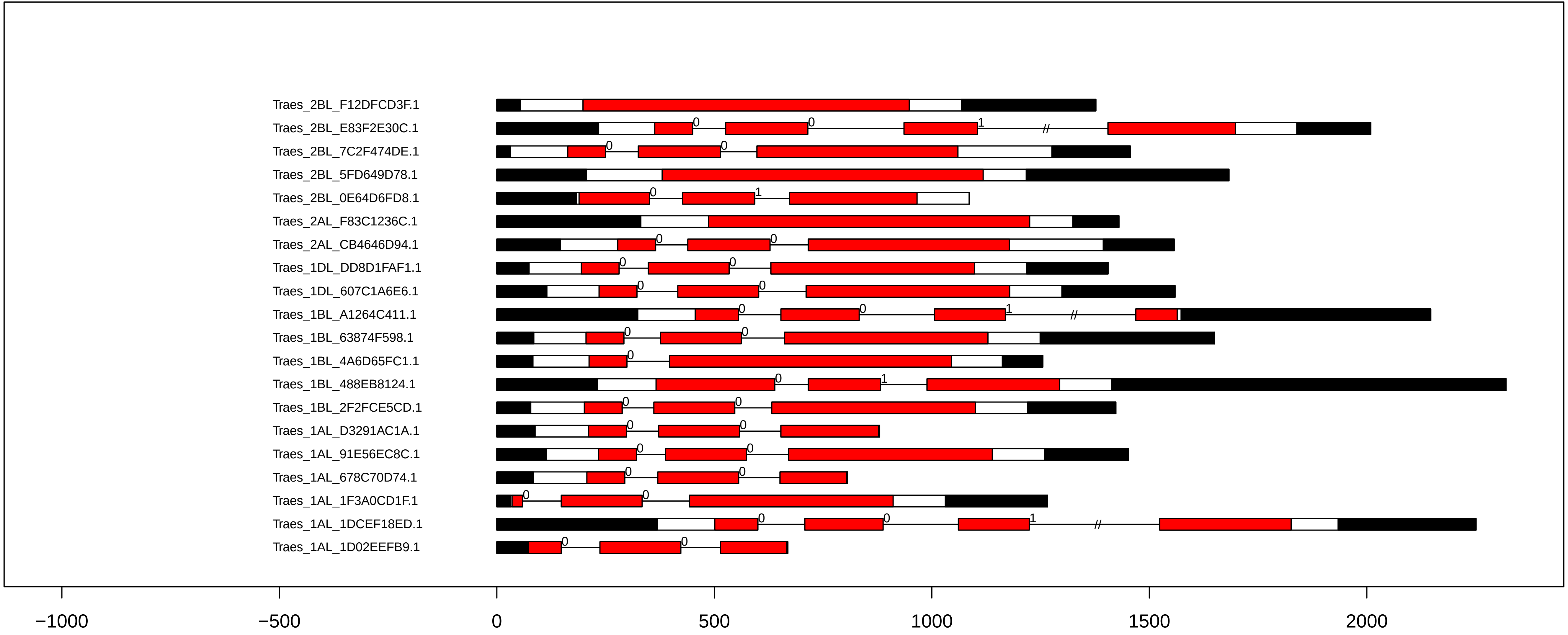

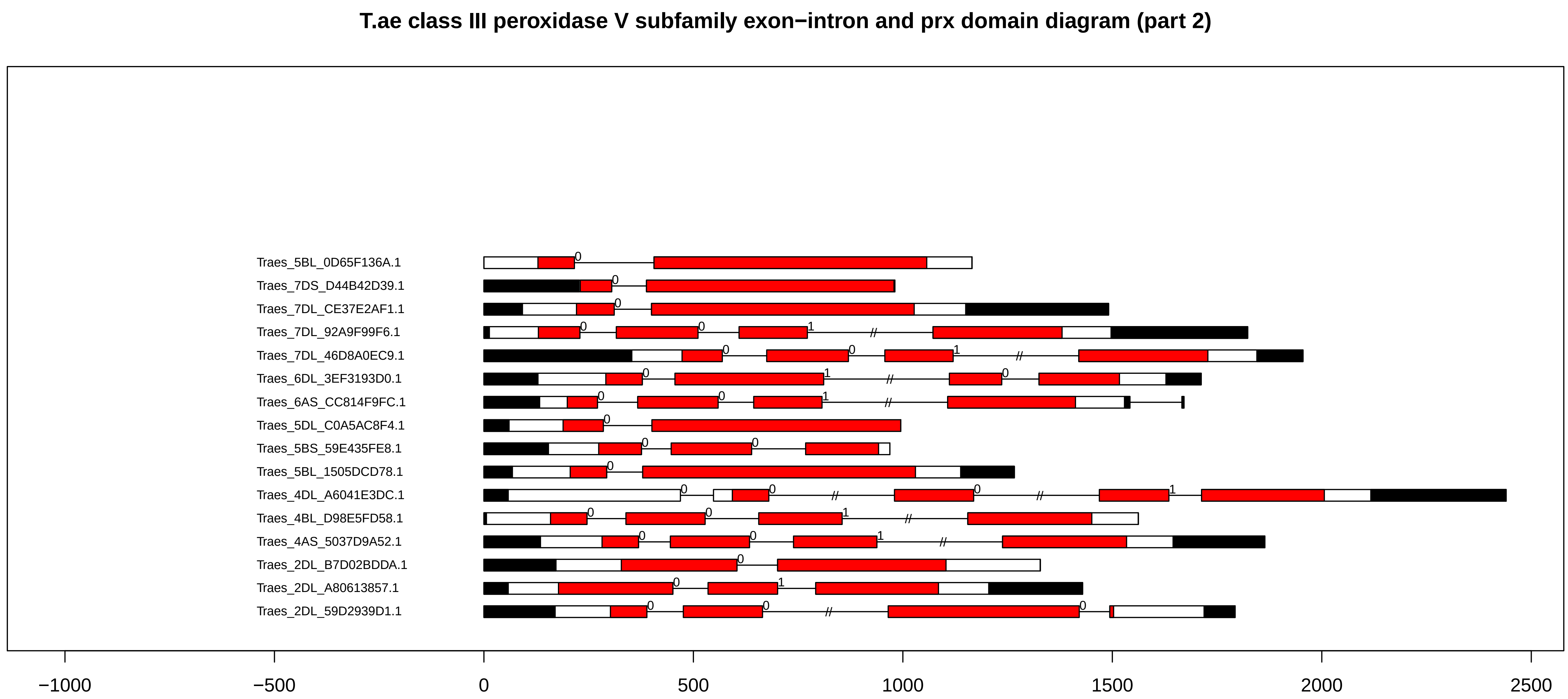

T.ae class III peroxidase V subfamily exon−intron and prx domain diagram (part 2)
Traes_5BL_0D65F136A.1
Traes_7DS_D44B42D39.1
Traes_7DL_CE37E2AF1.1
Traes_7DL_92A9F99F6.1
Traes_7DL_46D8A0EC9.1
Traes_6DL_3EF3193D0.1
Traes_6AS_CC814F9FC.1
Traes_5DL_C0A5AC8F4.1
Traes_5BS_59E435FE8.1
Traes_5BL_1505DCD78.1
Traes_4DL_A6041E3DC.1
Traes_4BL_D98E5FD58.1
Traes_4AS_5037D9A52.1
Traes_2DL_B7D02BDDA.1
Traes_2DL_A80613857.1
Traes_2DL_59D2939D1.1
−1000
−500
0
500
1000
1500
2000
2500

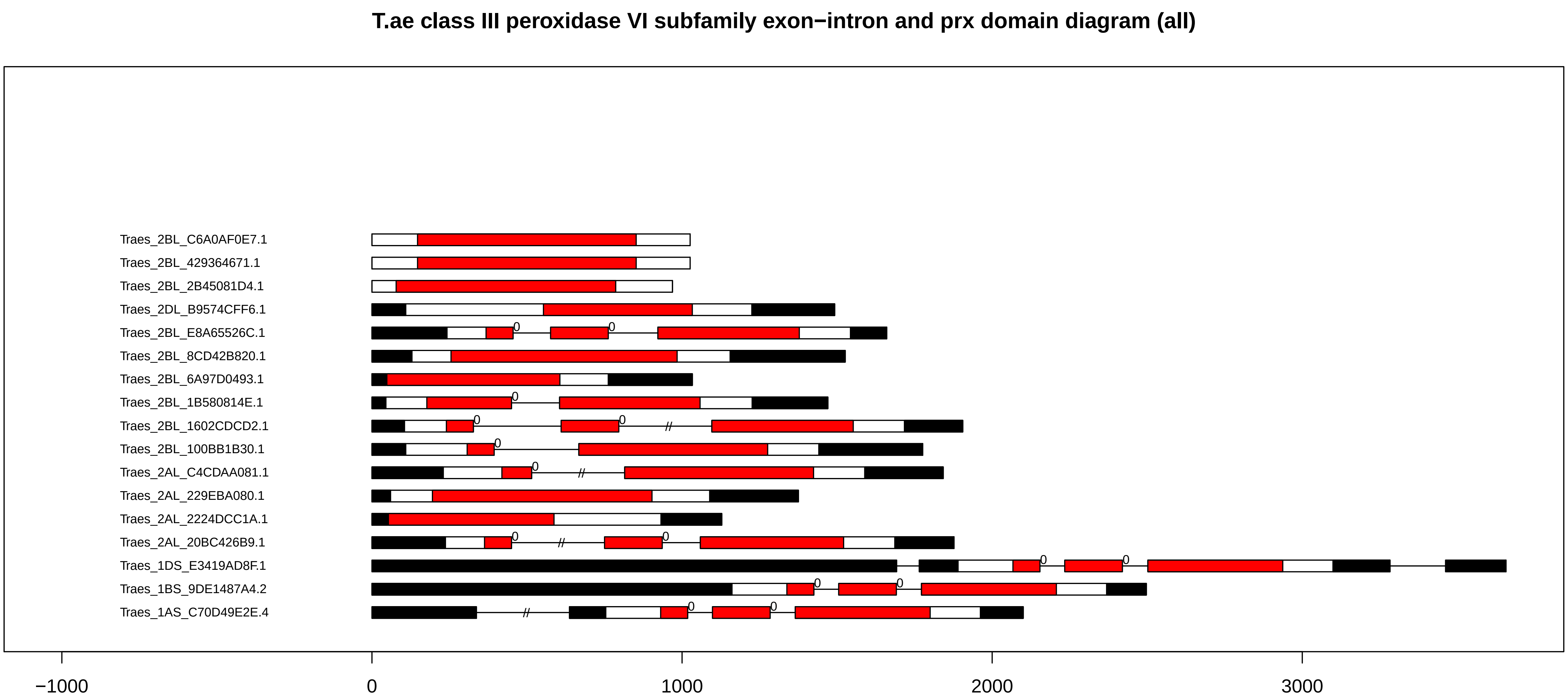

T.ae class III peroxidase VI subfamily exon−intron and prx domain diagram (all)
Traes_2BL_C6A0AF0E7.1
Traes_2BL_429364671.1
Traes_2BL_2B45081D4.1
Traes_2DL_B9574CFF6.1
Traes_2BL_E8A65526C.1
Traes_2BL_8CD42B820.1
Traes_2BL_6A97D0493.1
Traes_2BL_1B580814E.1
Traes_2BL_1602CDCD2.1
Traes_2BL_100BB1B30.1
Traes_2AL_C4CDAA081.1
Traes_2AL_229EBA080.1
Traes_2AL_2224DCC1A.1
Traes_2AL_20BC426B9.1
Traes_1DS_E3419AD8F.1
Traes_1BS_9DE1487A4.2
Traes_1AS_C70D49E2E.4
−1000
0
1000
2000
3000

# T.ae class III peroxidase VII subfamily exon−intron and prx domain diagram (all)

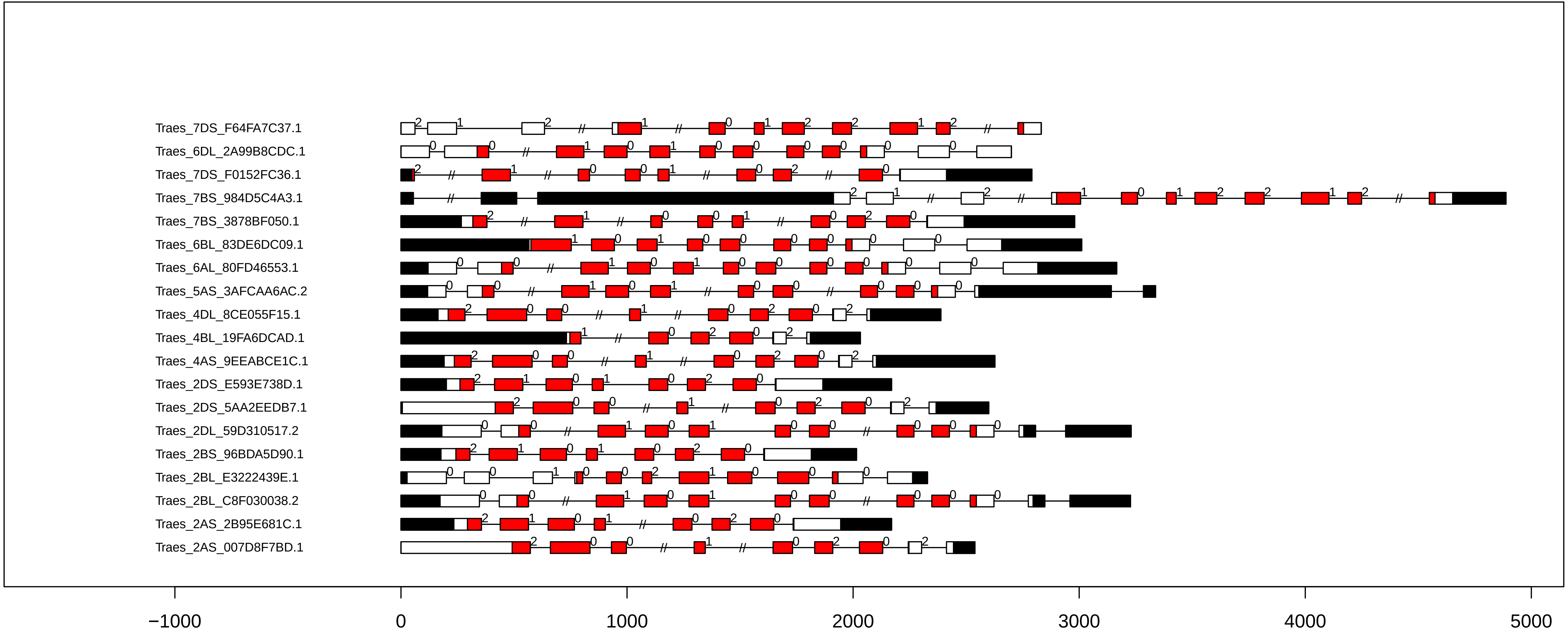

T.ae class III peroxidase IX subfamily exon−intron and prx domain diagram (all)

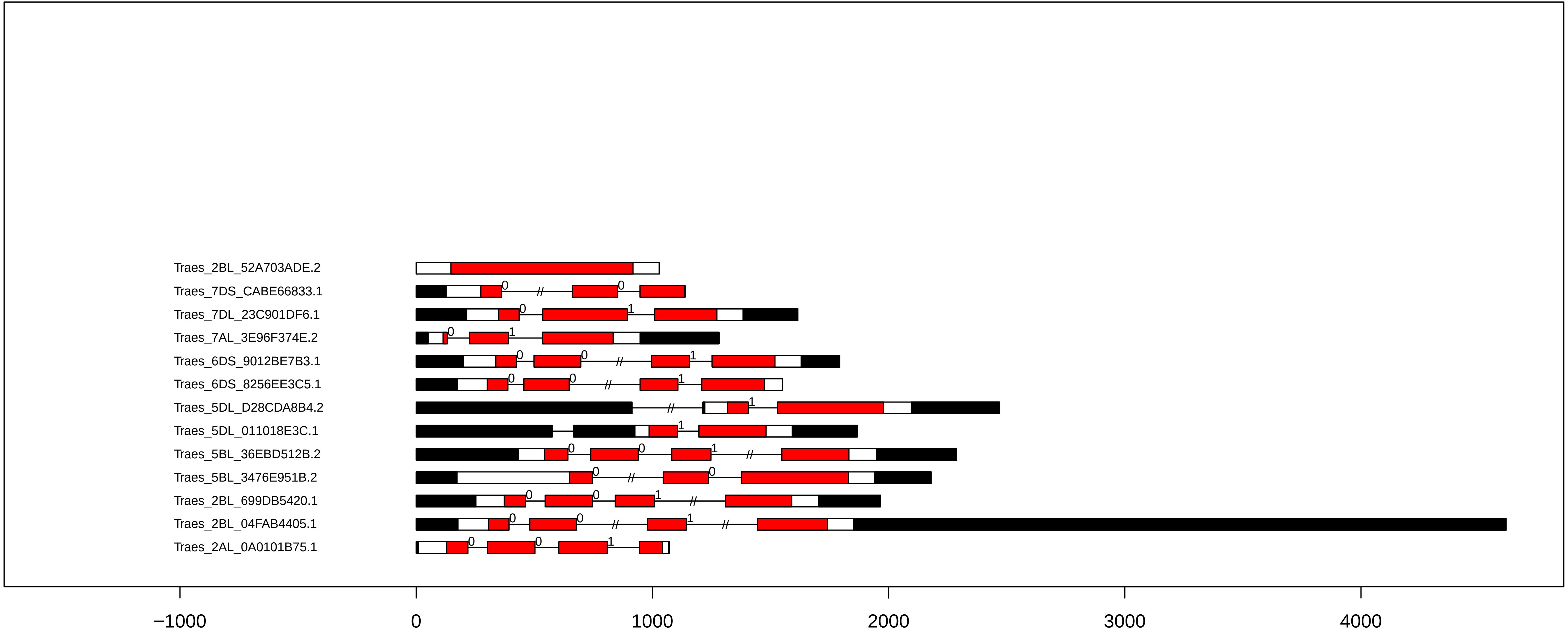

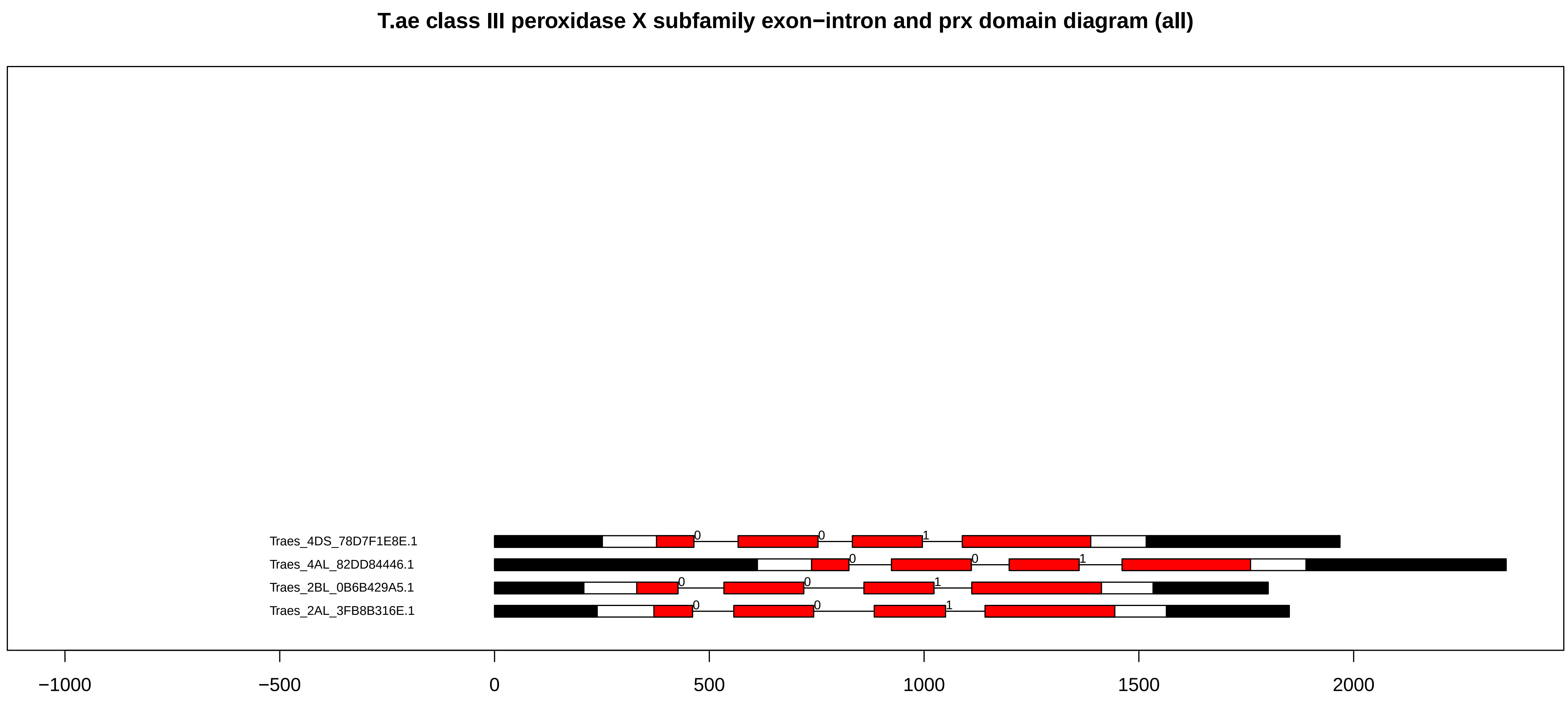

T.ae class III peroxidase X subfamily exon−intron and prx domain diagram (all)
Traes_4DS_78D7F1E8E.1
Traes_4AL_82DD84446.1
Traes_2BL_0B6B429A5.1
Traes_2AL_3FB8B316E.1
−1000
−500
0
500
1000
1500
2000

# T.ae class III peroxidase XII subfamily exon−intron and prx domain diagram (all)

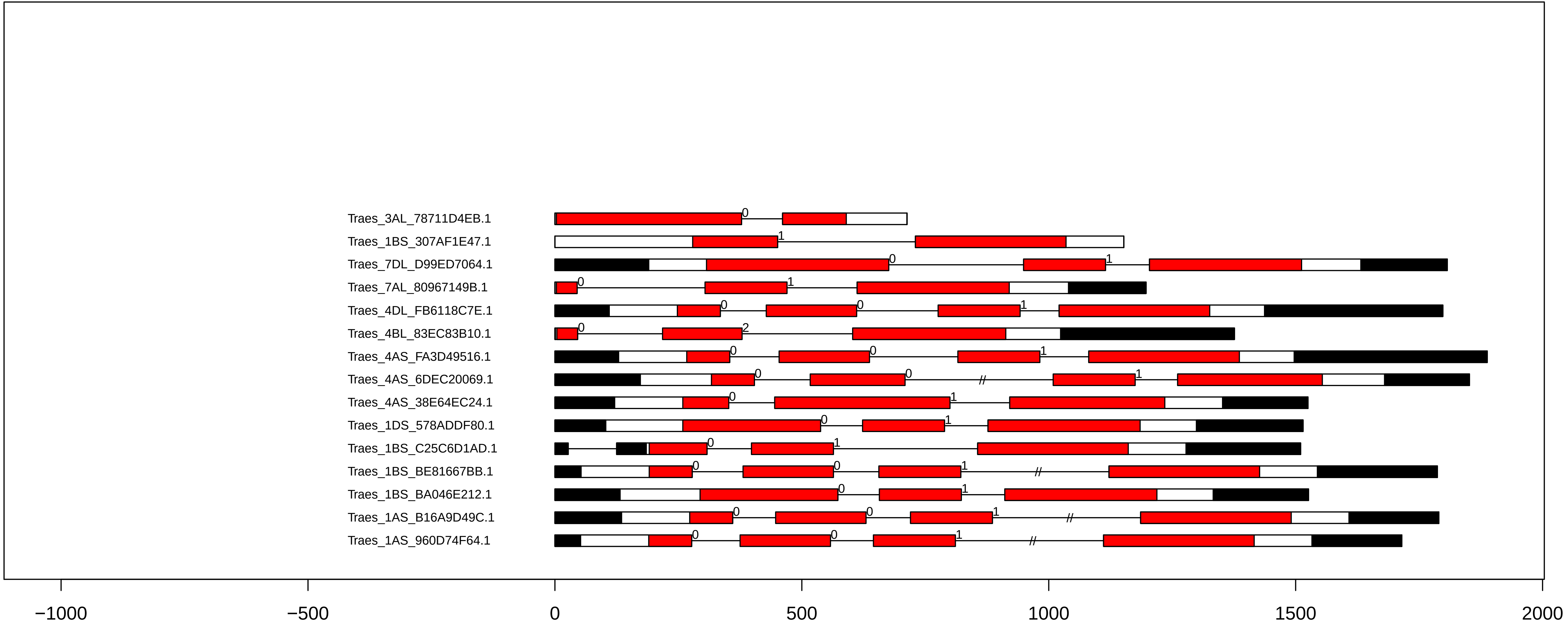

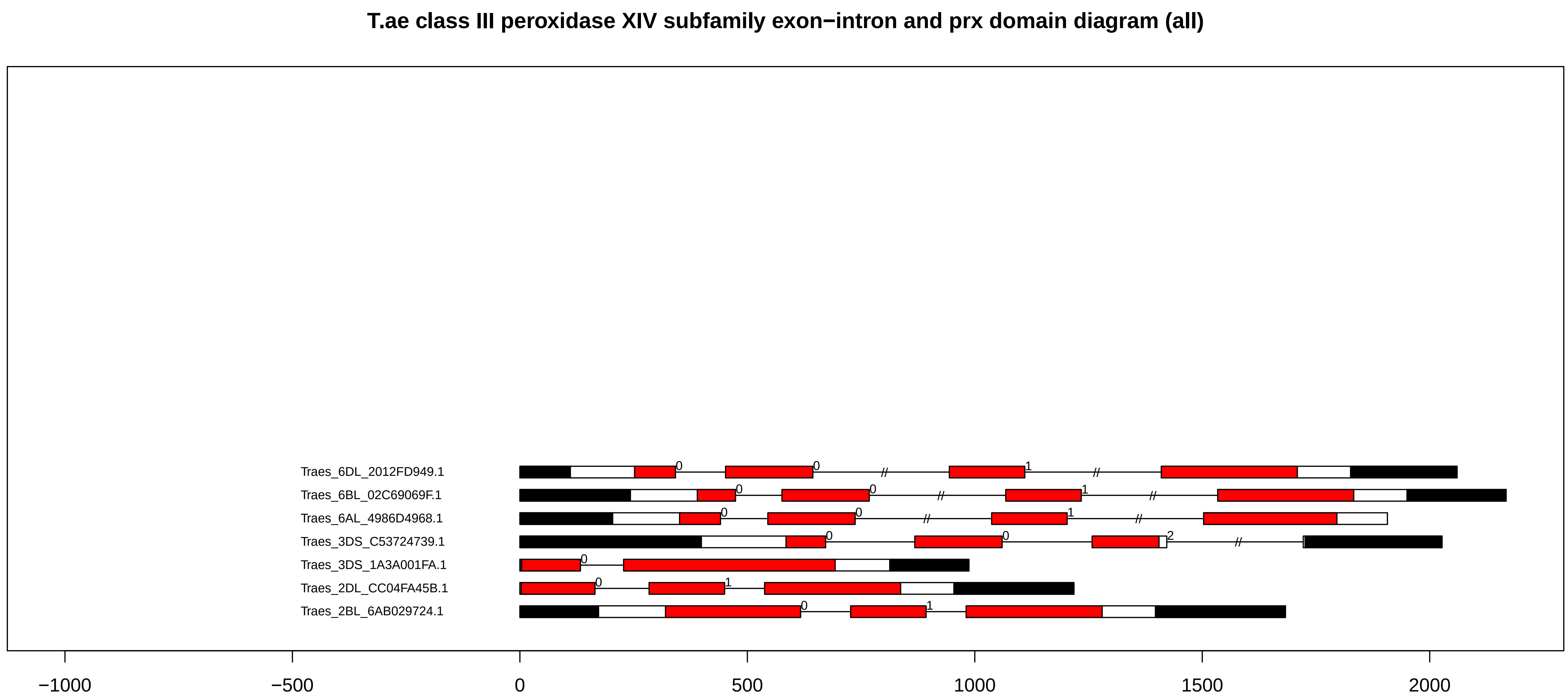
T.ae class III peroxidase XIV subfamily exon−intron and prx domain diagram (all)
Traes_6DL_2012FD949.1
Traes_6BL_02C69069F.1
Traes_6AL_4986D4968.1
Traes_3DS_C53724739.1
Traes_3DS_1A3A001FA.1
Traes_2DL_CC04FA45B.1
Traes_2BL_6AB029724.1
−1000
−500
0
500
1000
1500
2000

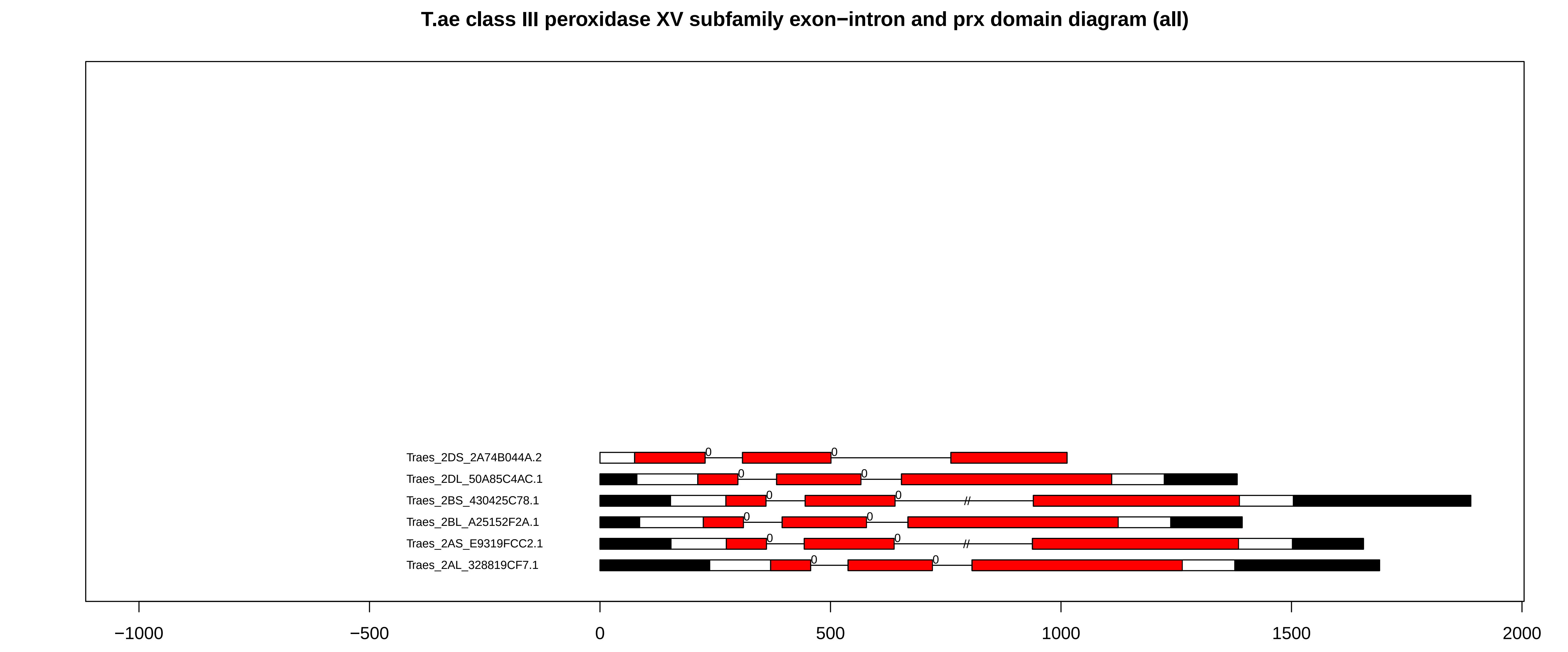

T.ae class III peroxidase XV subfamily exon−intron and prx domain diagram (all)
Traes_2DS_2A74B044A.2
Traes_2DL_50A85C4AC.1
Traes_2BS_430425C78.1
Traes_2BL_A25152F2A.1
Traes_2AS_E9319FCC2.1
Traes_2AL_328819CF7.1
−1000
−500
0
500
1000
1500
2000

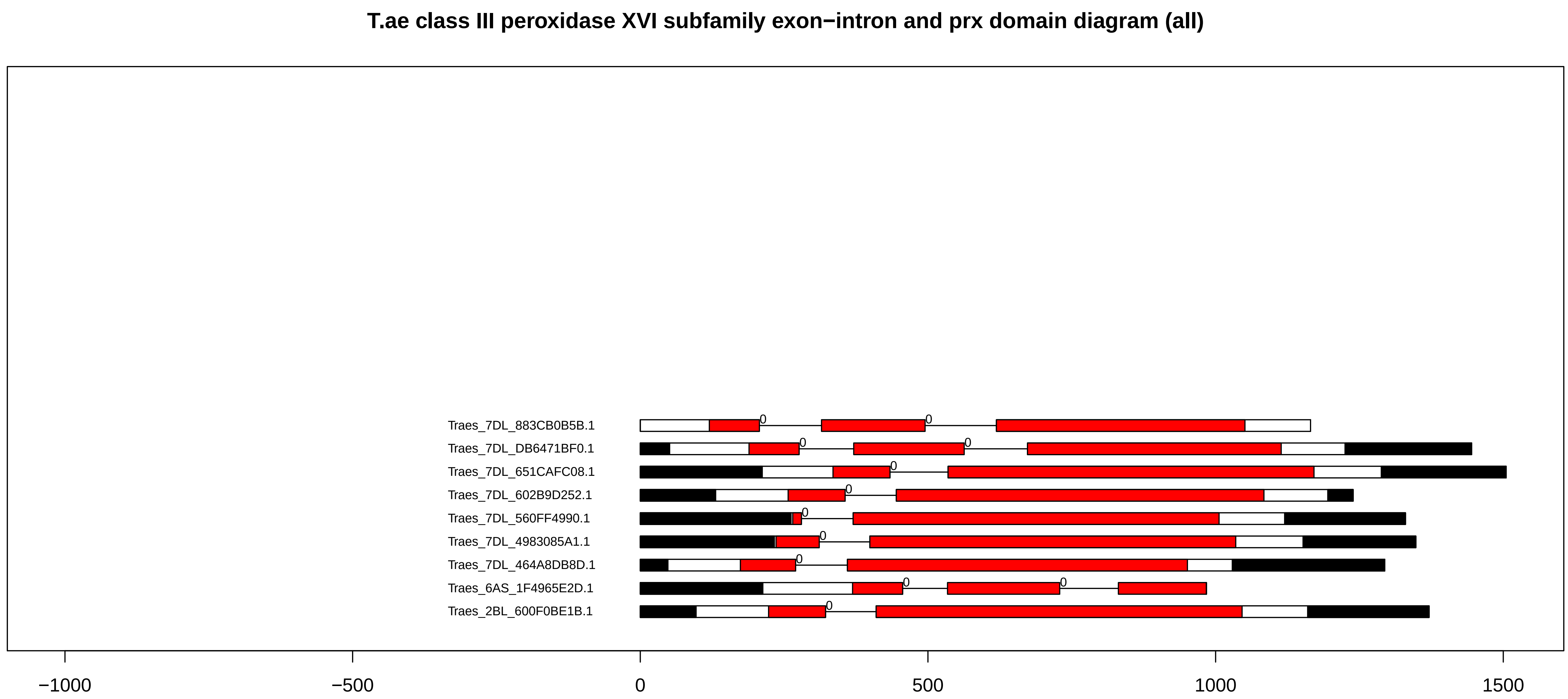
T.ae class III peroxidase XVI subfamily exon−intron and prx domain diagram (all)
Traes_7DL_883CB0B5B.1
Traes_7DL_DB6471BF0.1
Traes_7DL_651CAFC08.1
Traes_7DL_602B9D252.1
Traes_7DL_560FF4990.1
Traes_7DL_4983085A1.1
Traes_7DL_464A8DB8D.1
Traes_6AS_1F4965E2D.1
Traes_2BL_600F0BE1B.1
0
−1000
−500
0
500
1000
1500

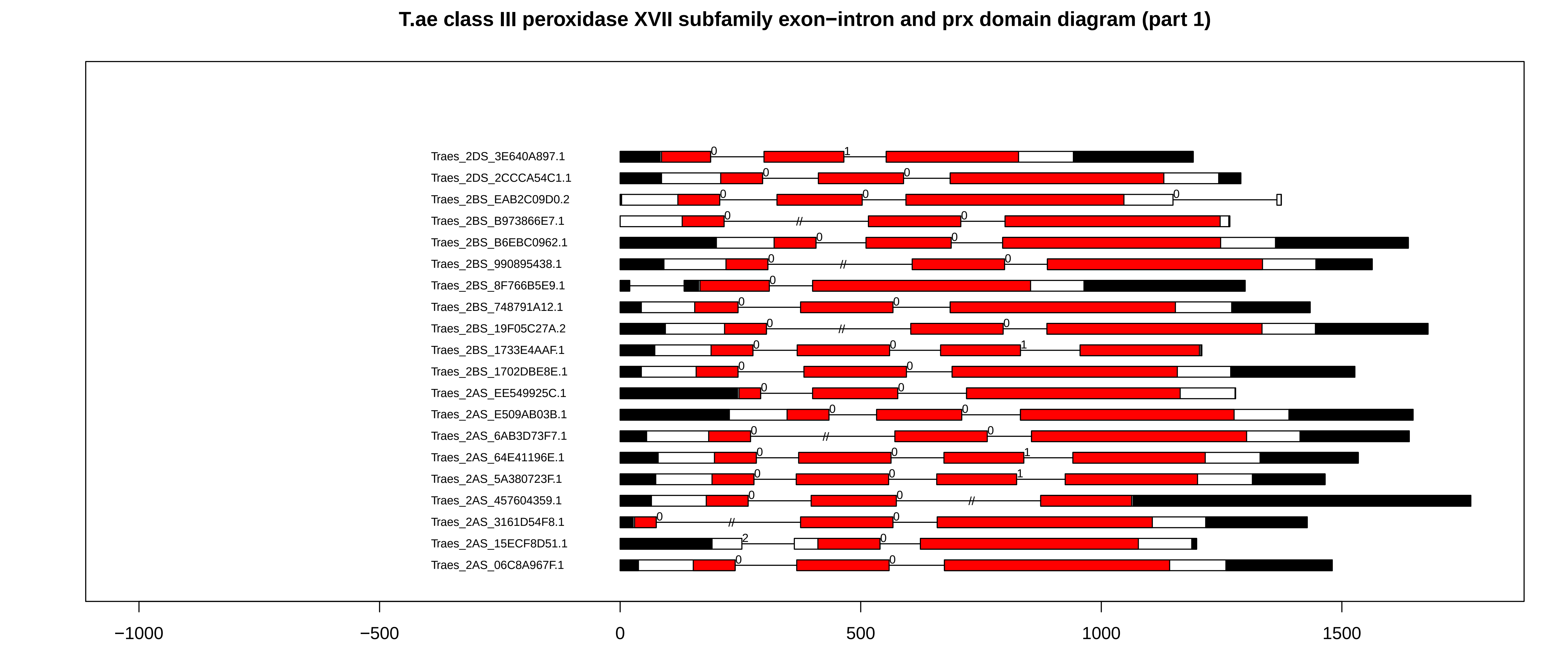

T.ae class III peroxidase XVII subfamily exon−intron and prx domain diagram (part 1)
Traes_2DS_3E640A897.1
Traes_2DS_2CCCA54C1.1
Traes_2BS_EAB2C09D0.2
Traes_2BS_B973866E7.1
Traes_2BS_B6EBC0962.1
Traes_2BS_990895438.1
Traes_2BS_8F766B5E9.1
Traes_2BS_748791A12.1
Traes_2BS_19F05C27A.2
Traes_2BS_1733E4AAF.1
Traes_2BS_1702DBE8E.1
Traes_2AS_EE549925C.1
Traes_2AS_E509AB03B.1
Traes_2AS_6AB3D73F7.1
Traes_2AS_64E41196E.1
Traes_2AS_5A380723F.1
Traes_2AS_457604359.1
Traes_2AS_3161D54F8.1
Traes_2AS_15ECF8D51.1
Traes_2AS_06C8A967F.1
−1000
−500
0
500
1000
1500

## T.ae class III peroxidase XVII subfamily exon−intron and prx domain diagram (part 2)

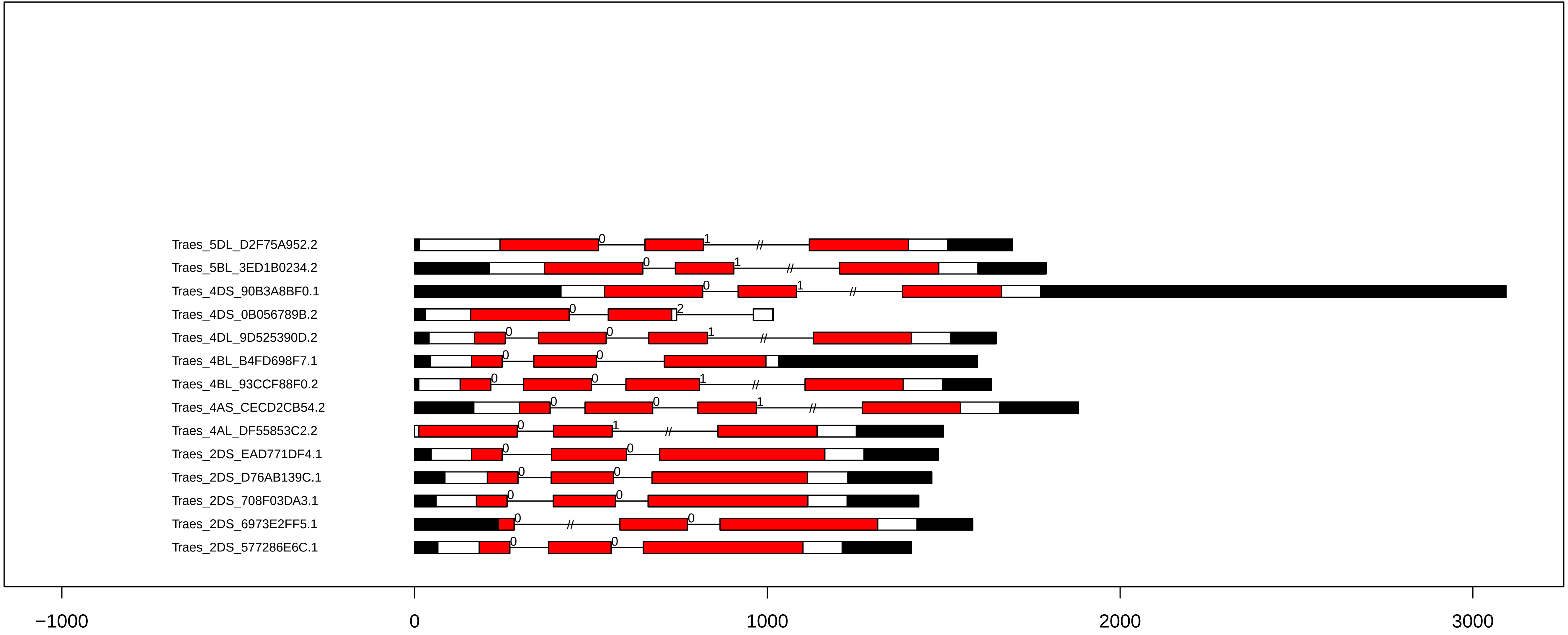

## T.ae class III peroxidase XVIII subfamily exon−intron and prx domain diagram (all)

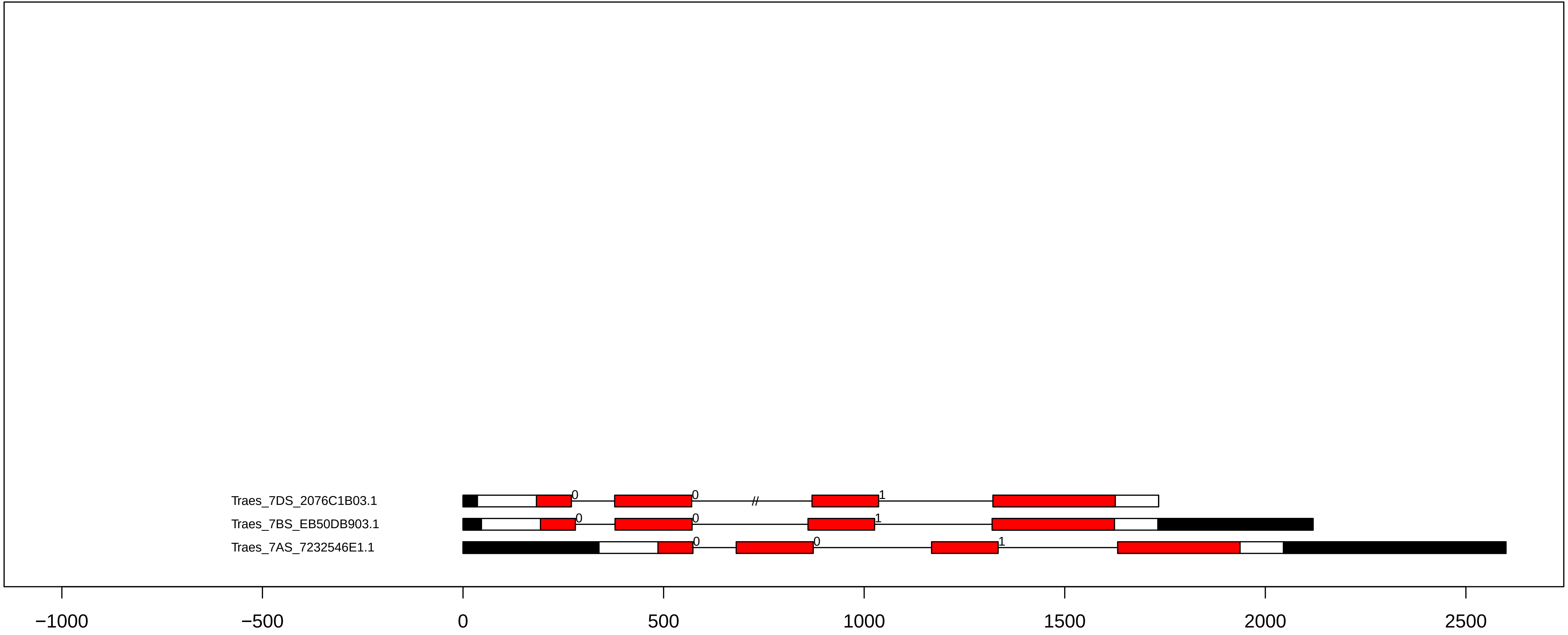

# T.ae class III peroxidase unclear_classification subfamily exon−intron and prx domain diagram (part 1)

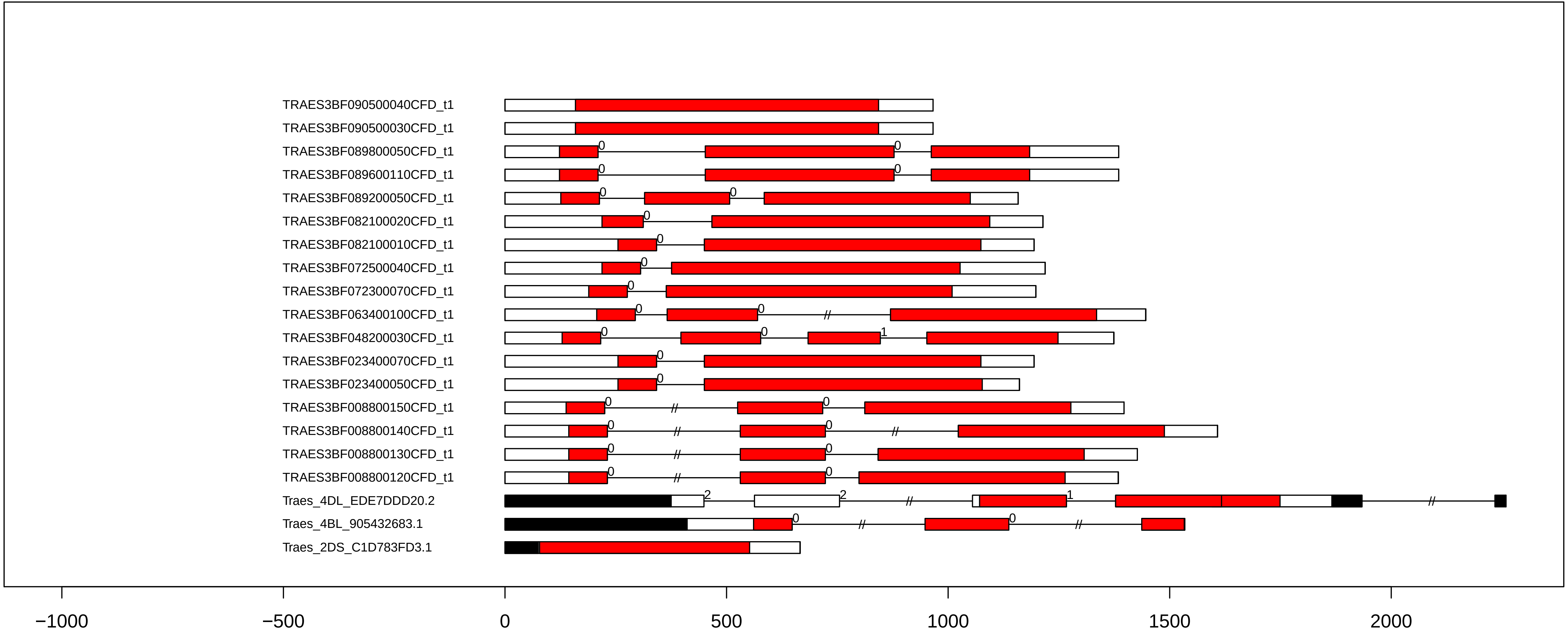

T.ae class III peroxidase unclear_classification subfamily exon−intron and prx domain diagram (part 2)

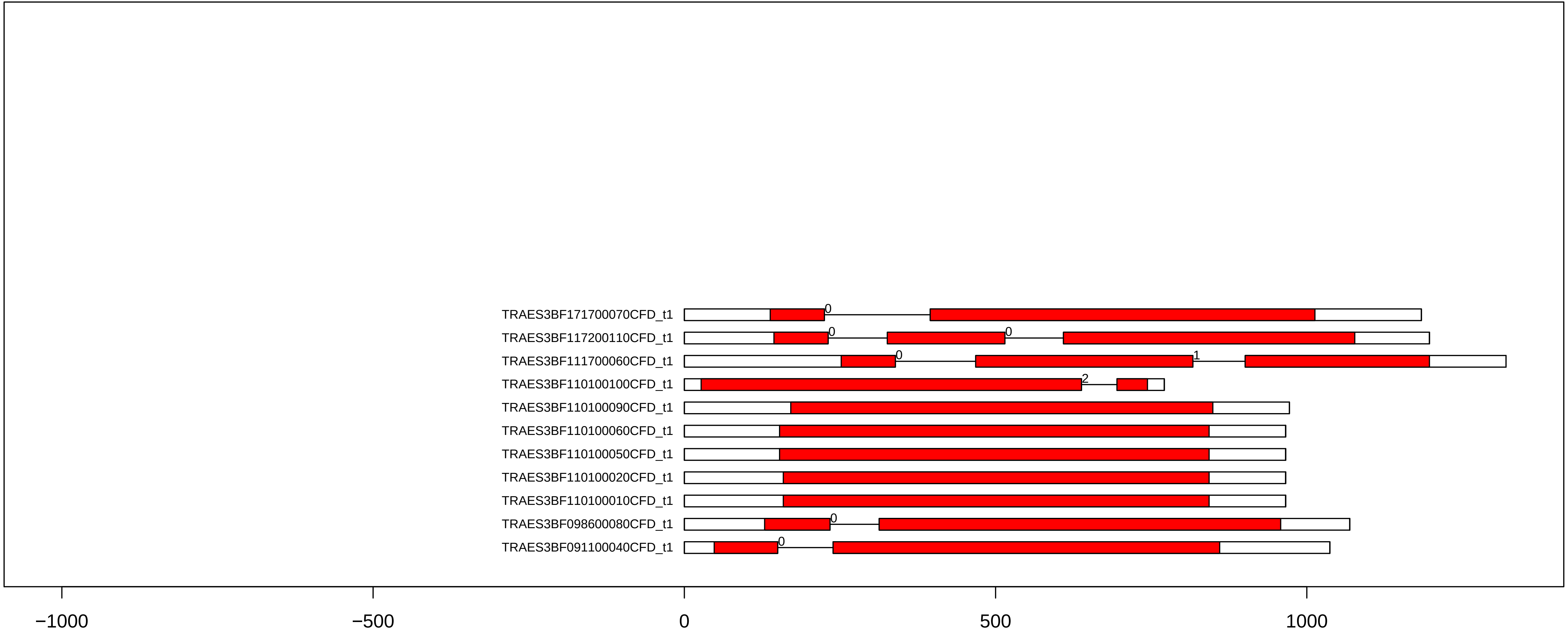

## T.ur class III peroxidase I subfamily exon−intron and prx domain diagram (part 1)

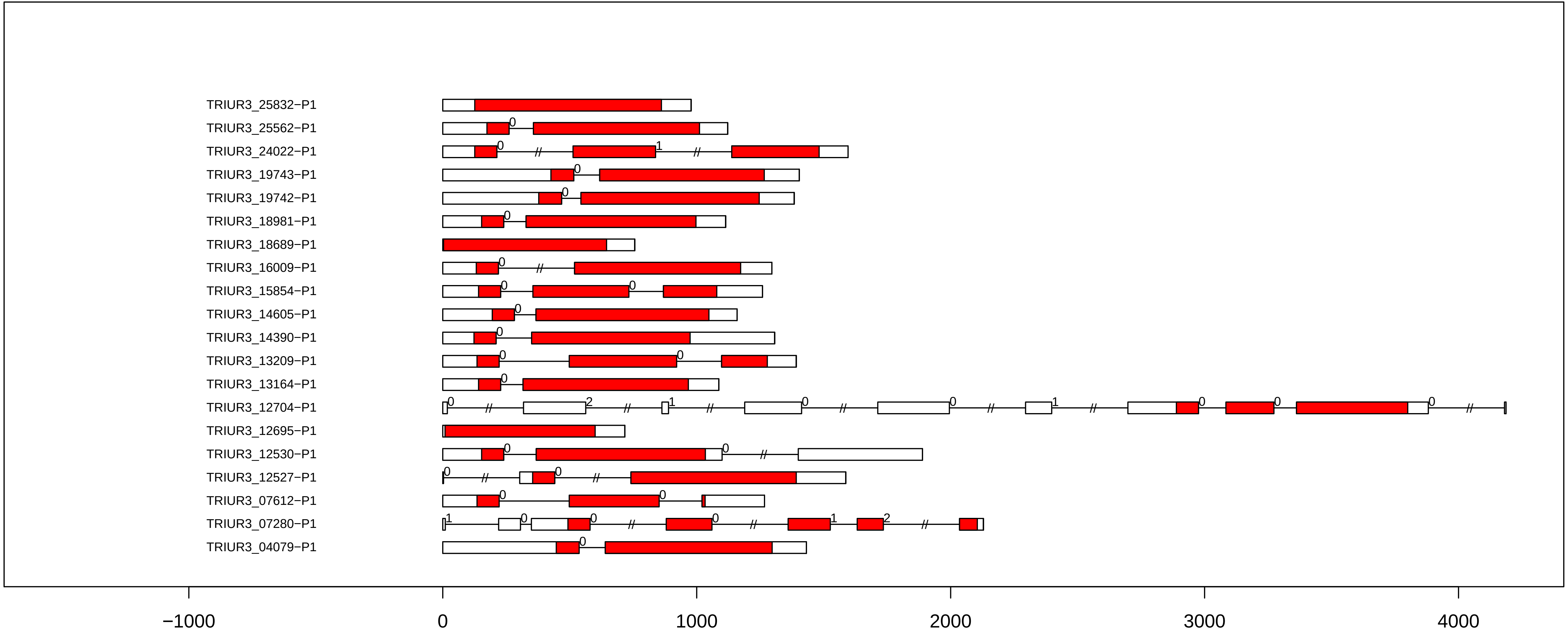

# T.ur class III peroxidase I subfamily exon−intron and prx domain diagram (part 2)

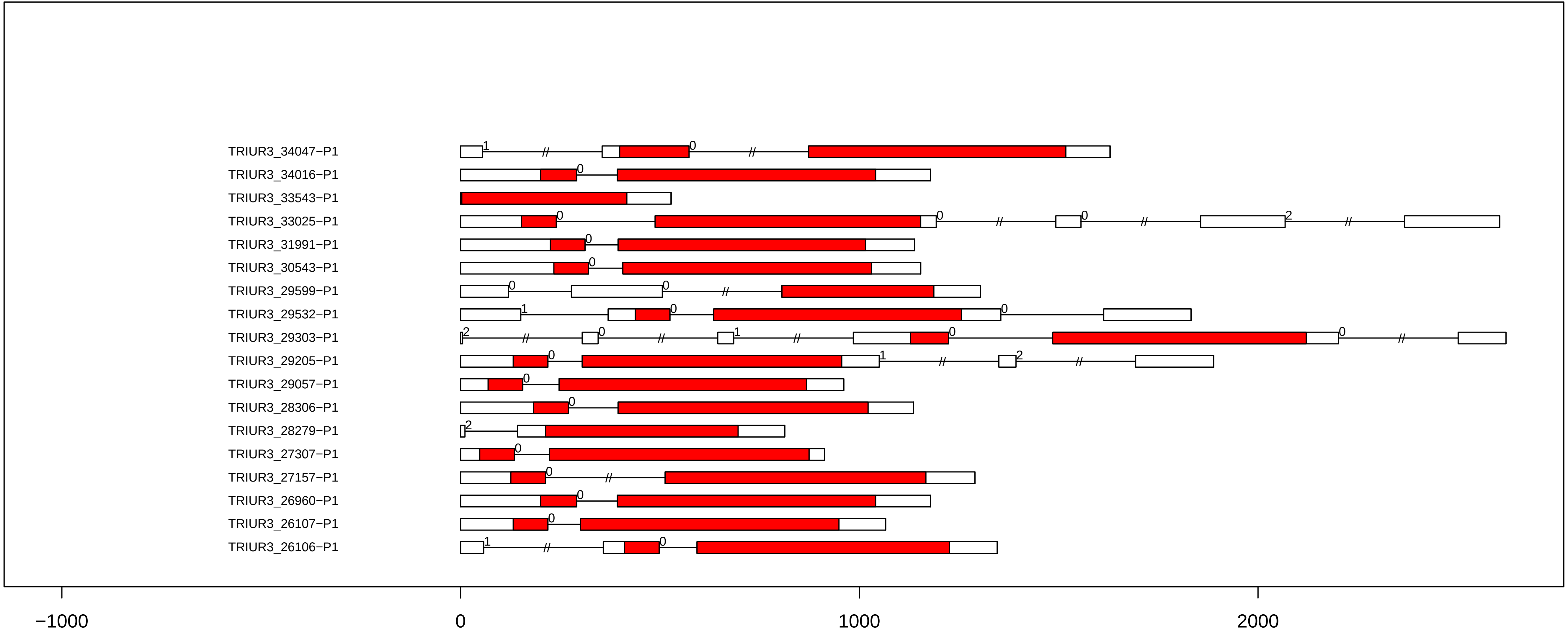

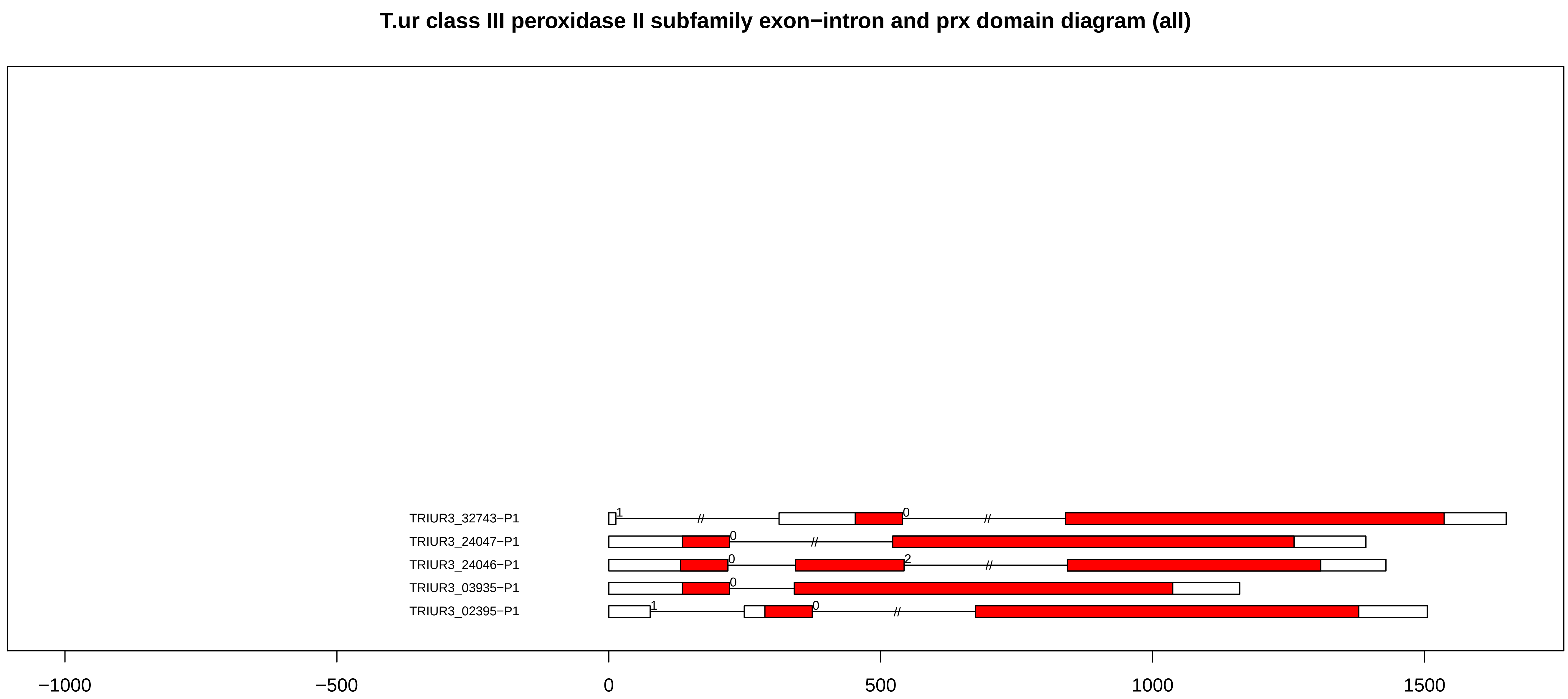
T.ur class III peroxidase II subfamily exon−intron and prx domain diagram (all)
TRIUR3_32743−P1
TRIUR3_24047−P1
TRIUR3_24046−P1
TRIUR3_03935−P1
TRIUR3_02395−P1
1
0
0
0
2
0
1
0
−1000
−500
0
500
1000
1500

T.ur class III peroxidase V subfamily exon−intron and prx domain diagram (part 1)

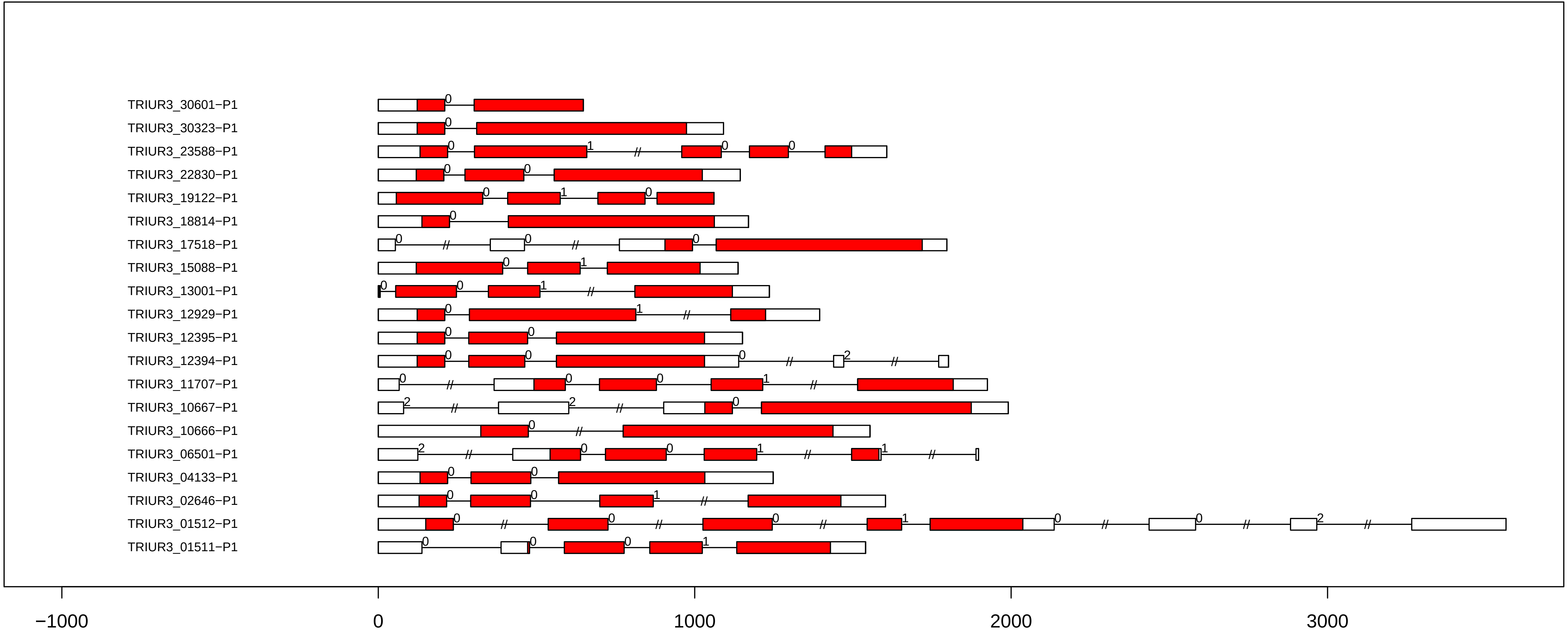

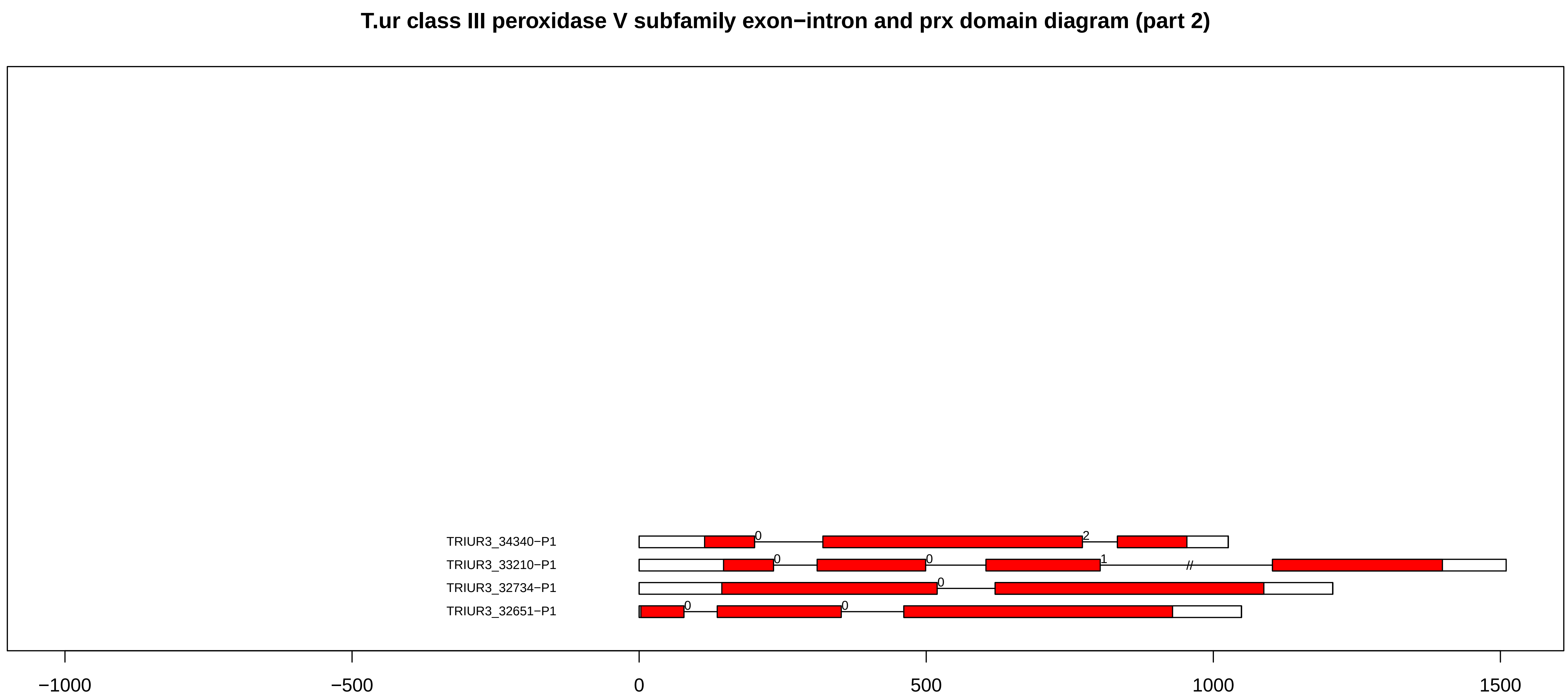

T.ur class III peroxidase V subfamily exon−intron and prx domain diagram (part 2)
TRIUR3_34340−P1
TRIUR3_33210−P1
TRIUR3_32734−P1
TRIUR3_32651−P1
0
2
0
0
1
//
0
0
0
−1000
−500
0
500
1000
1500

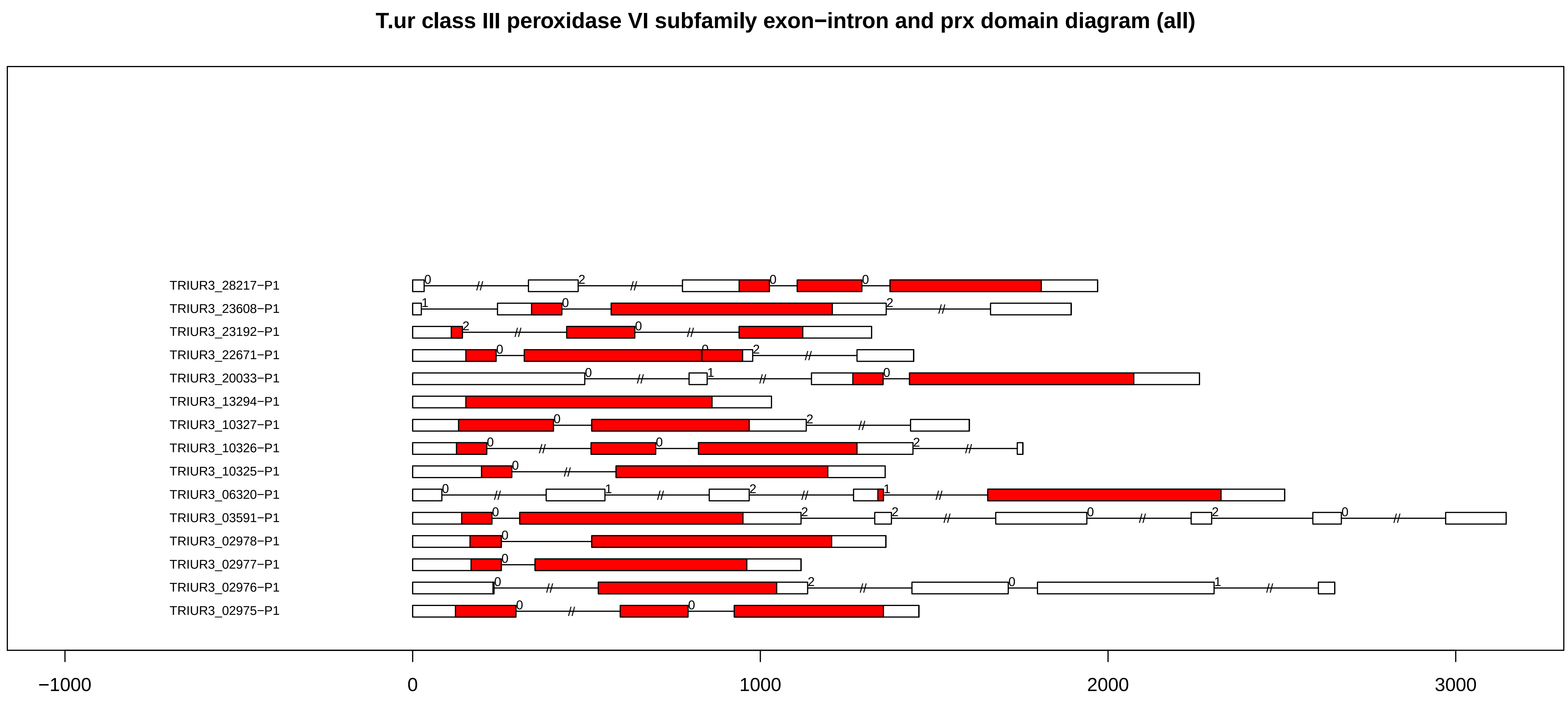
T.ur class III peroxidase VI subfamily exon−intron and prx domain diagram (all)
TRIUR3_28217−P1
TRIUR3_23608−P1
TRIUR3_23192−P1
TRIUR3_22671−P1
TRIUR3_20033−P1
TRIUR3_13294−P1
TRIUR3_10327−P1
TRIUR3_10326−P1
TRIUR3_10325−P1
TRIUR3_06320−P1
TRIUR3_03591−P1
TRIUR3_02978−P1
TRIUR3_02977−P1
TRIUR3_02976−P1
TRIUR3_02975−P1
−1000
0
1000
2000
3000

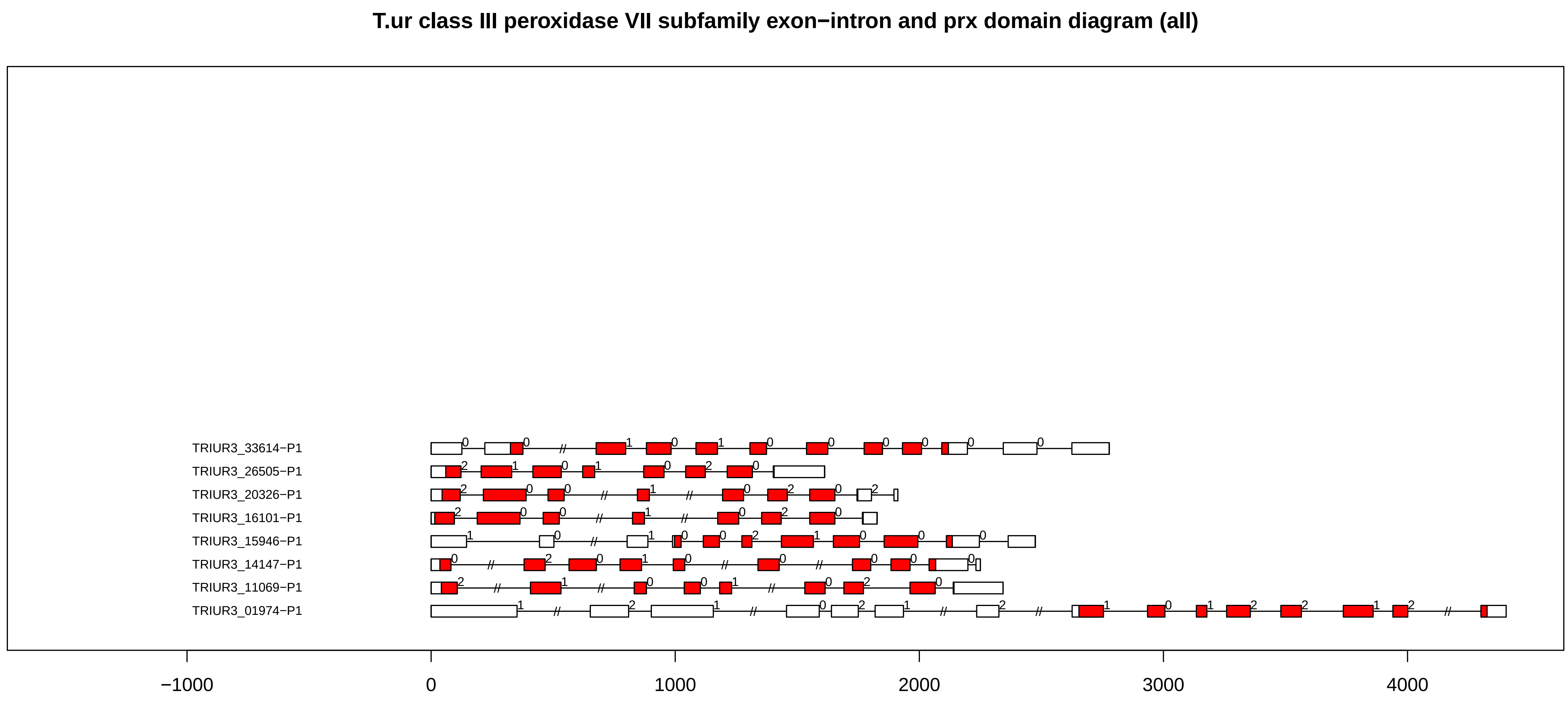
T.ur class III peroxidase VII subfamily exon−intron and prx domain diagram (all)
TRIUR3_33614−P1
TRIUR3_26505−P1
TRIUR3_20326−P1
TRIUR3_16101−P1
TRIUR3_15946−P1
TRIUR3_14147−P1
TRIUR3_11069−P1
TRIUR3_01974−P1
−1000
0
1000
2000
3000
4000

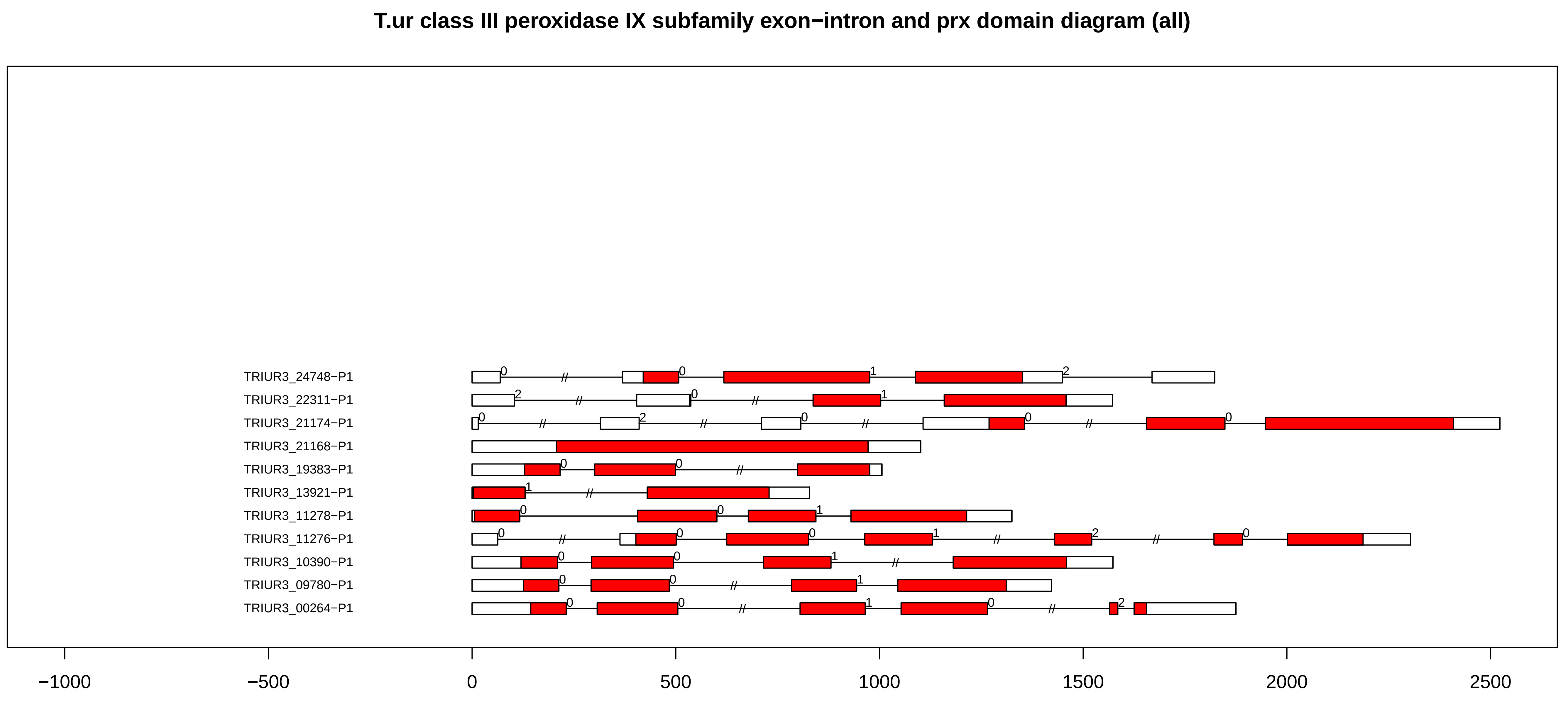
T.ur class III peroxidase IX subfamily exon−intron and prx domain diagram (all)
TRIUR3_24748−P1
TRIUR3_22311−P1
TRIUR3_21174−P1
TRIUR3_21168−P1
TRIUR3_19383−P1
TRIUR3_13921−P1
TRIUR3_11278−P1
TRIUR3_11276−P1
TRIUR3_10390−P1
TRIUR3_09780−P1
TRIUR3_00264−P1
−1000
−500
0
500
1000
1500
2000
2500

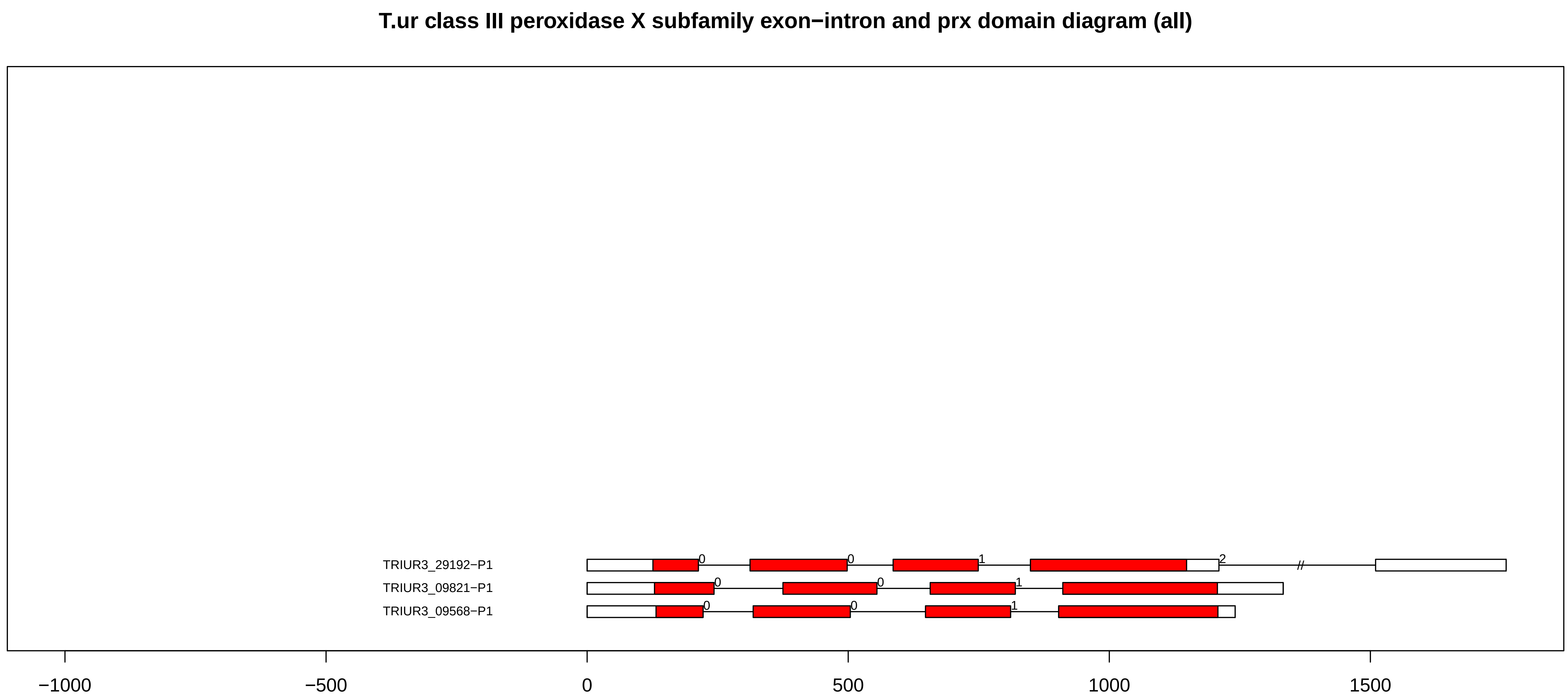
T.ur class III peroxidase X subfamily exon−intron and prx domain diagram (all)
TRIUR3_29192−P1
TRIUR3_09821−P1
TRIUR3_09568−P1
0
0
1
2
//
0
0
1
0
0
1
−1000
−500
0
500
1000
1500

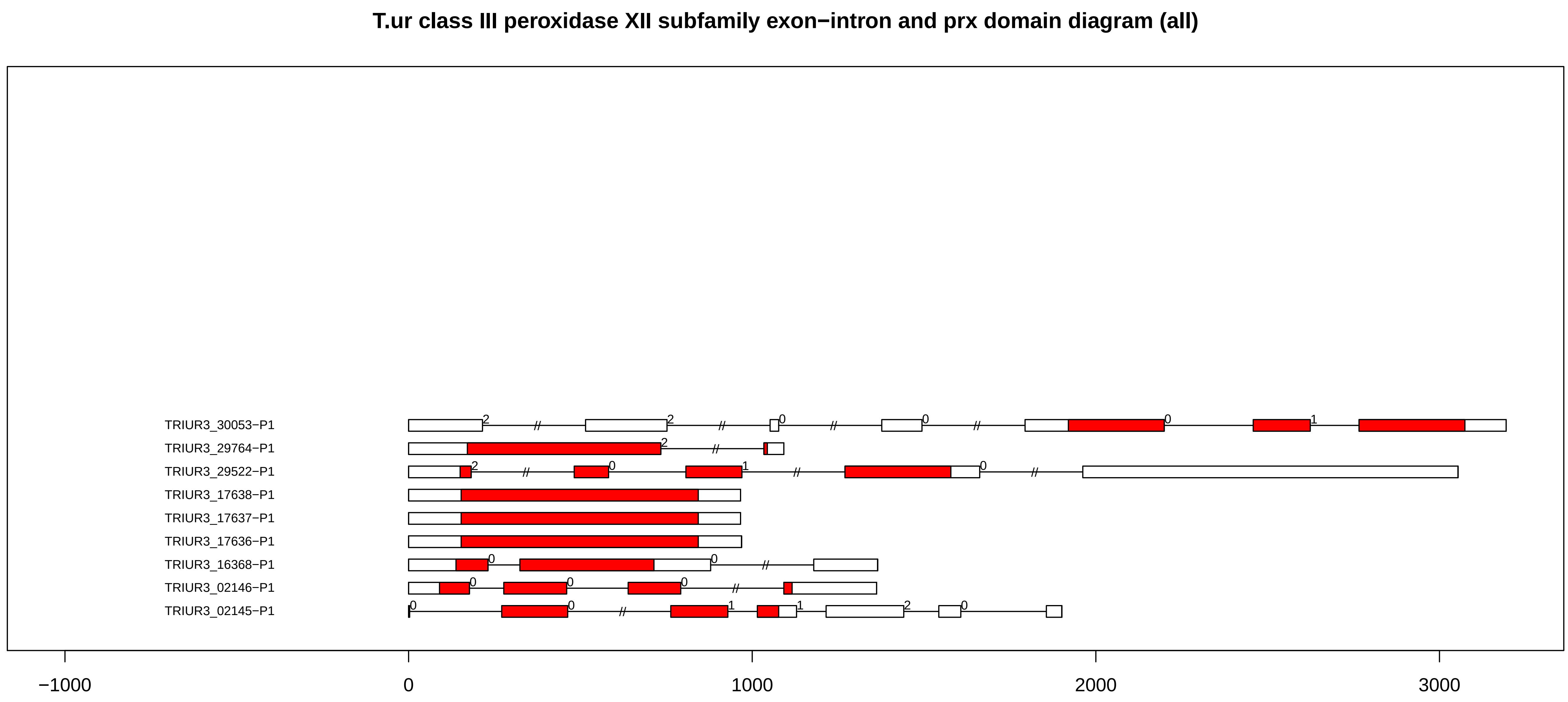

T.ur class III peroxidase XII subfamily exon−intron and prx domain diagram (all)
TRIUR3_30053−P1
TRIUR3_29764−P1
TRIUR3_29522−P1
TRIUR3_17638−P1
TRIUR3_17637−P1
TRIUR3_17636−P1
TRIUR3_16368−P1
TRIUR3_02146−P1
TRIUR3_02145−P1
−1000
0
1000
2000
3000

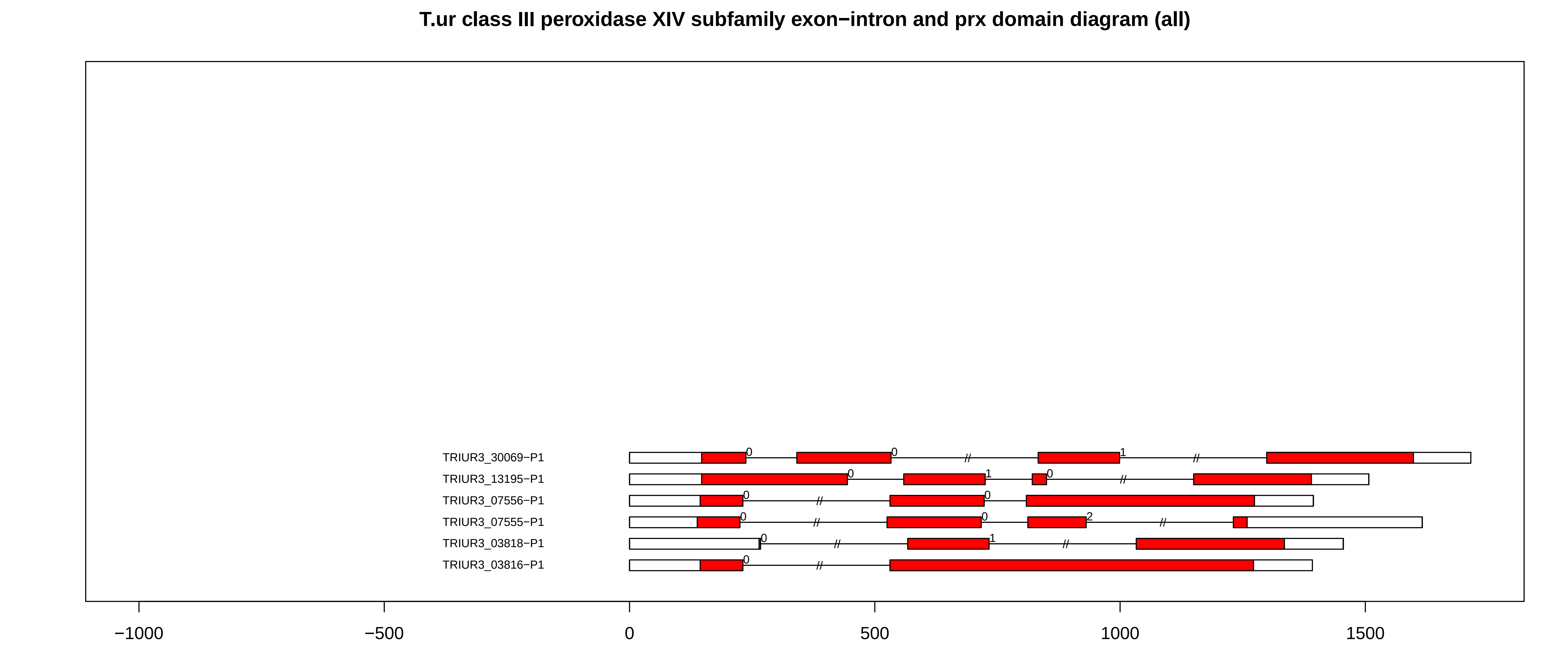

T.ur class III peroxidase XIV subfamily exon−intron and prx domain diagram (all)
TRIUR3_30069−P1
TRIUR3_13195−P1
TRIUR3_07556−P1
TRIUR3_07555−P1
TRIUR3_03818−P1
TRIUR3_03816−P1
−1000
−500
0
500
1000
1500

## T.ur class III peroxidase XV subfamily exon−intron and prx domain diagram (all)

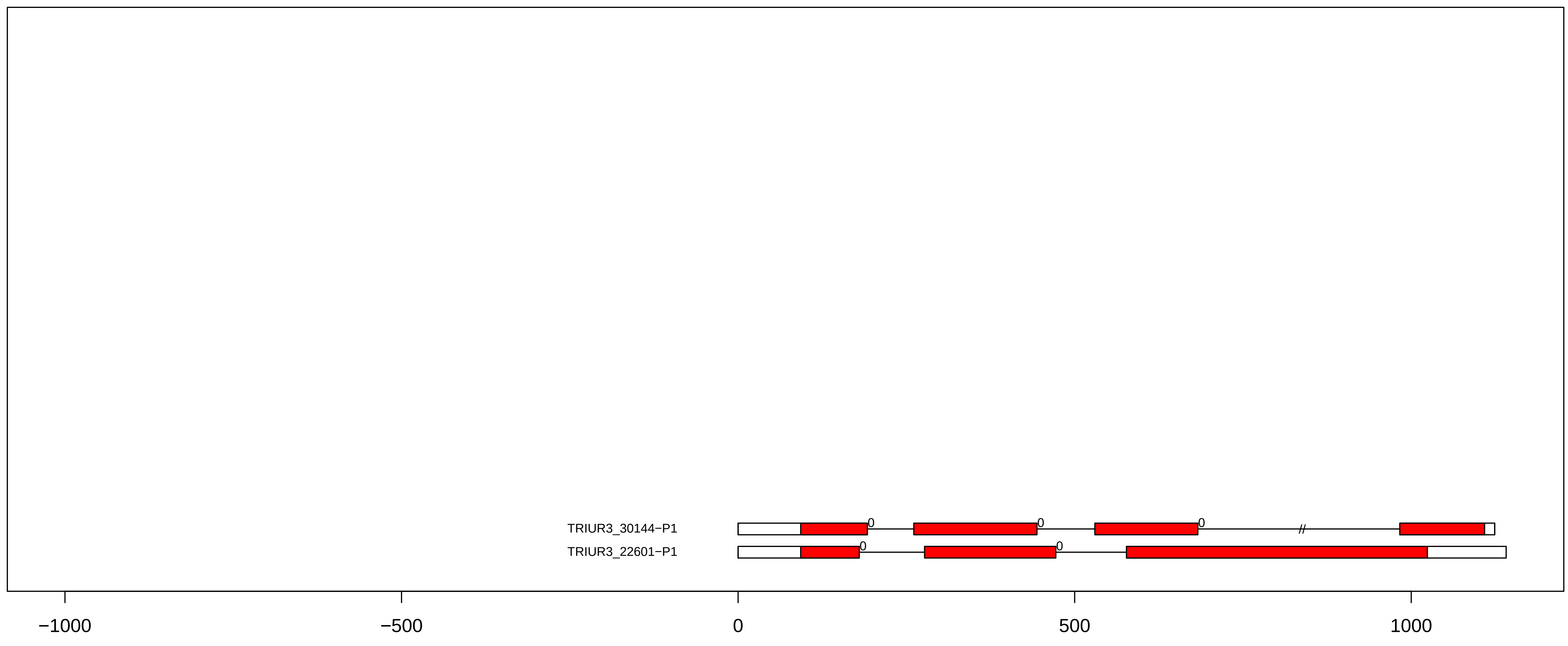

## T.ur class III peroxidase XVI subfamily exon−intron and prx domain diagram (all)

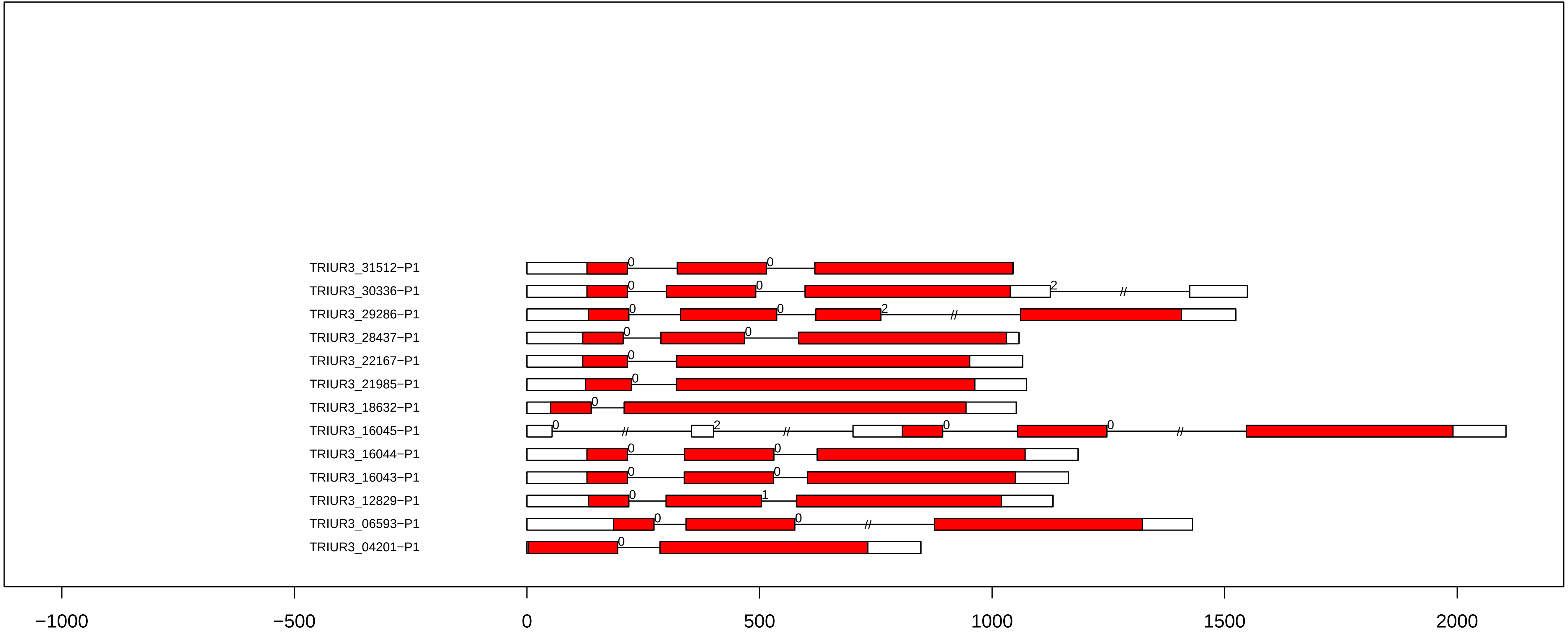

## T.ur class III peroxidase XVII subfamily exon−intron and prx domain diagram (all)

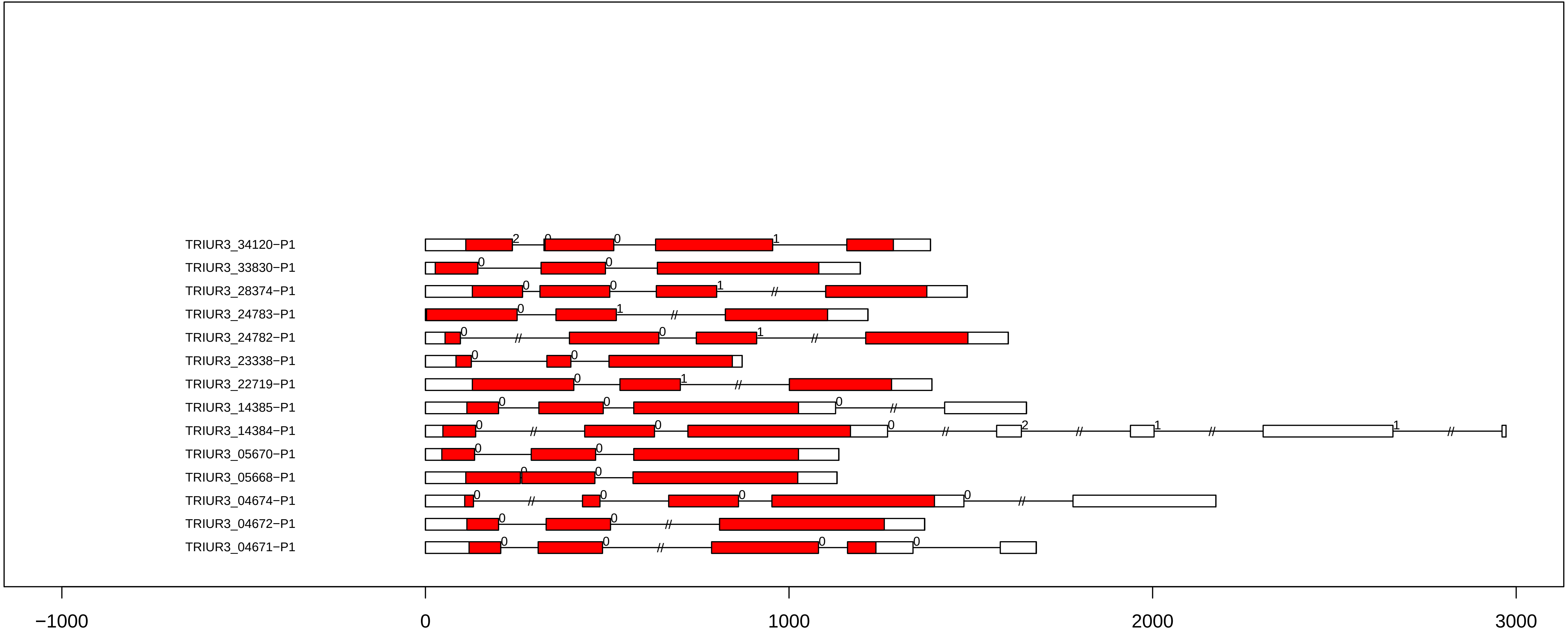

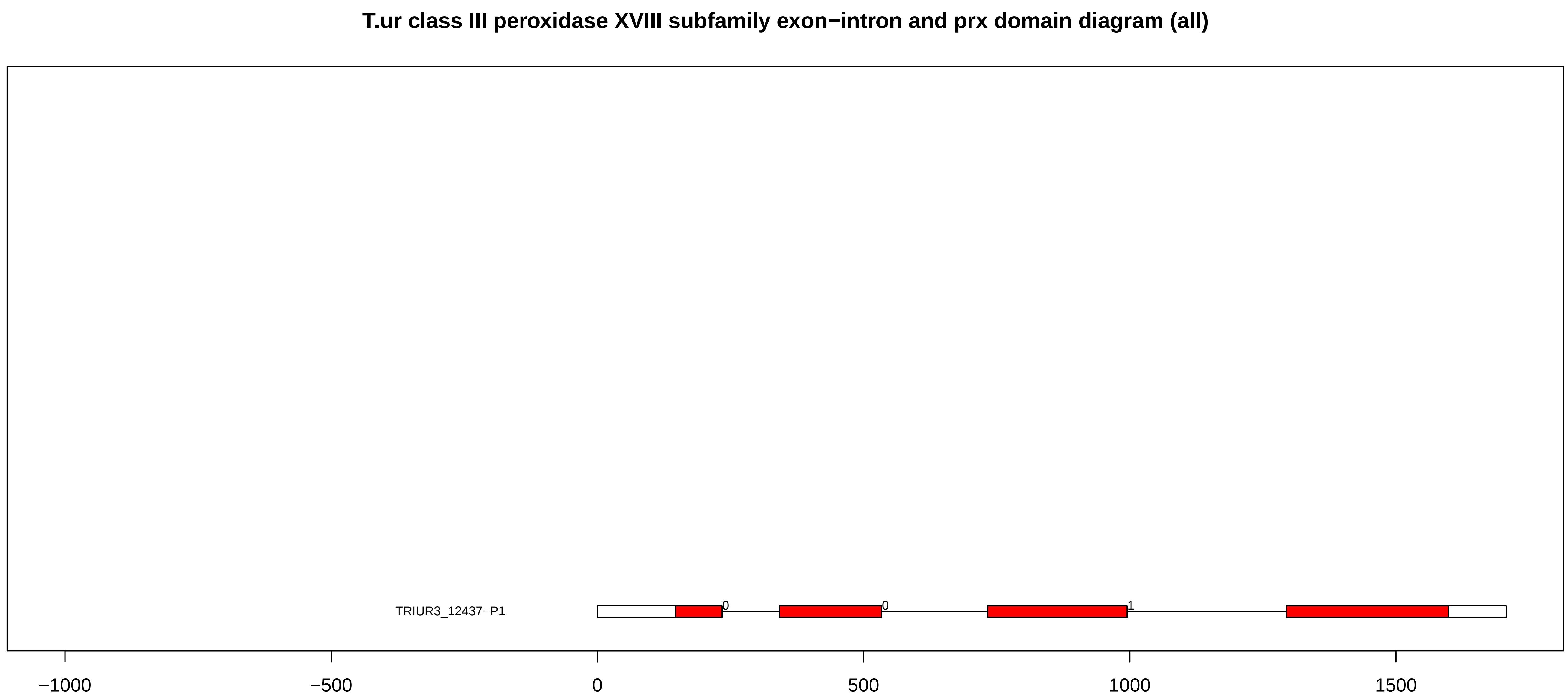
T.ur class III peroxidase XVIII subfamily exon−intron and prx domain diagram (all)
TRIUR3_12437−P1
0
0
1
−1000
−500
0
500
1000
1500

## T.ur class III peroxidase unclear_classification subfamily exon−intron and prx domain diagram (all)

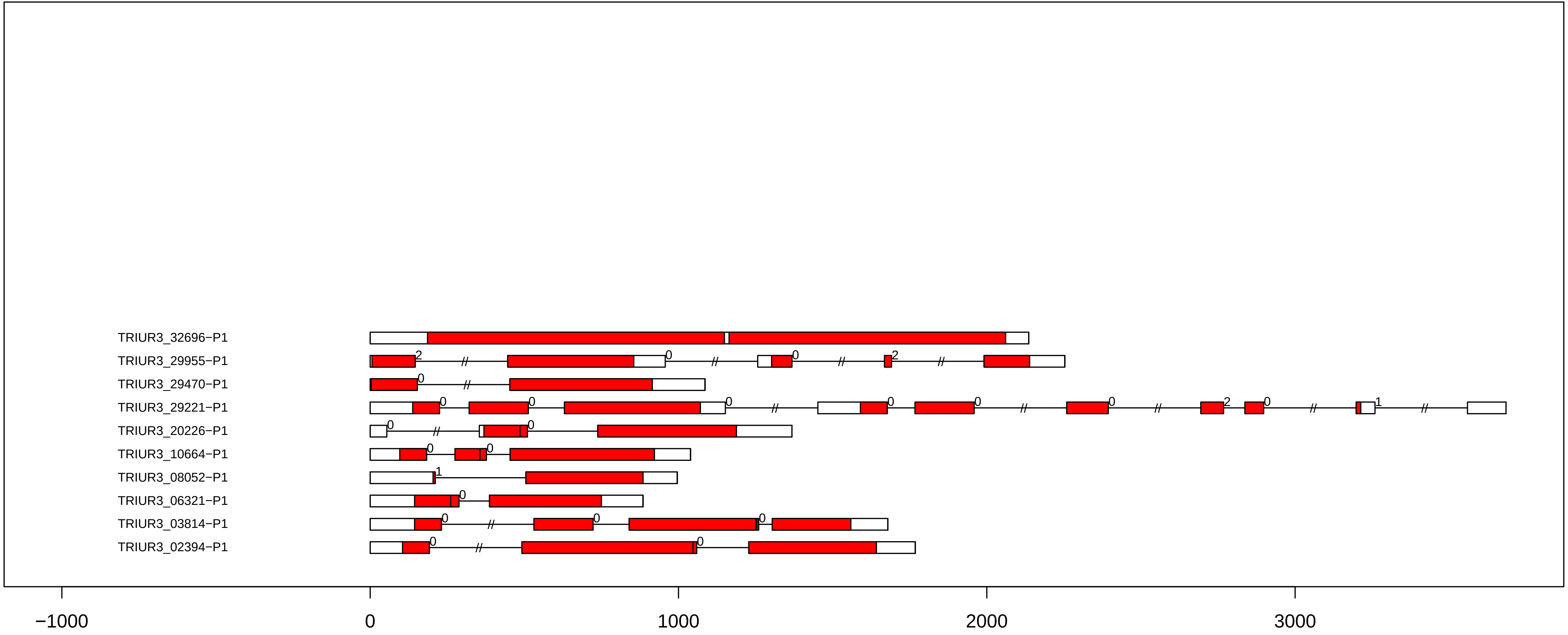

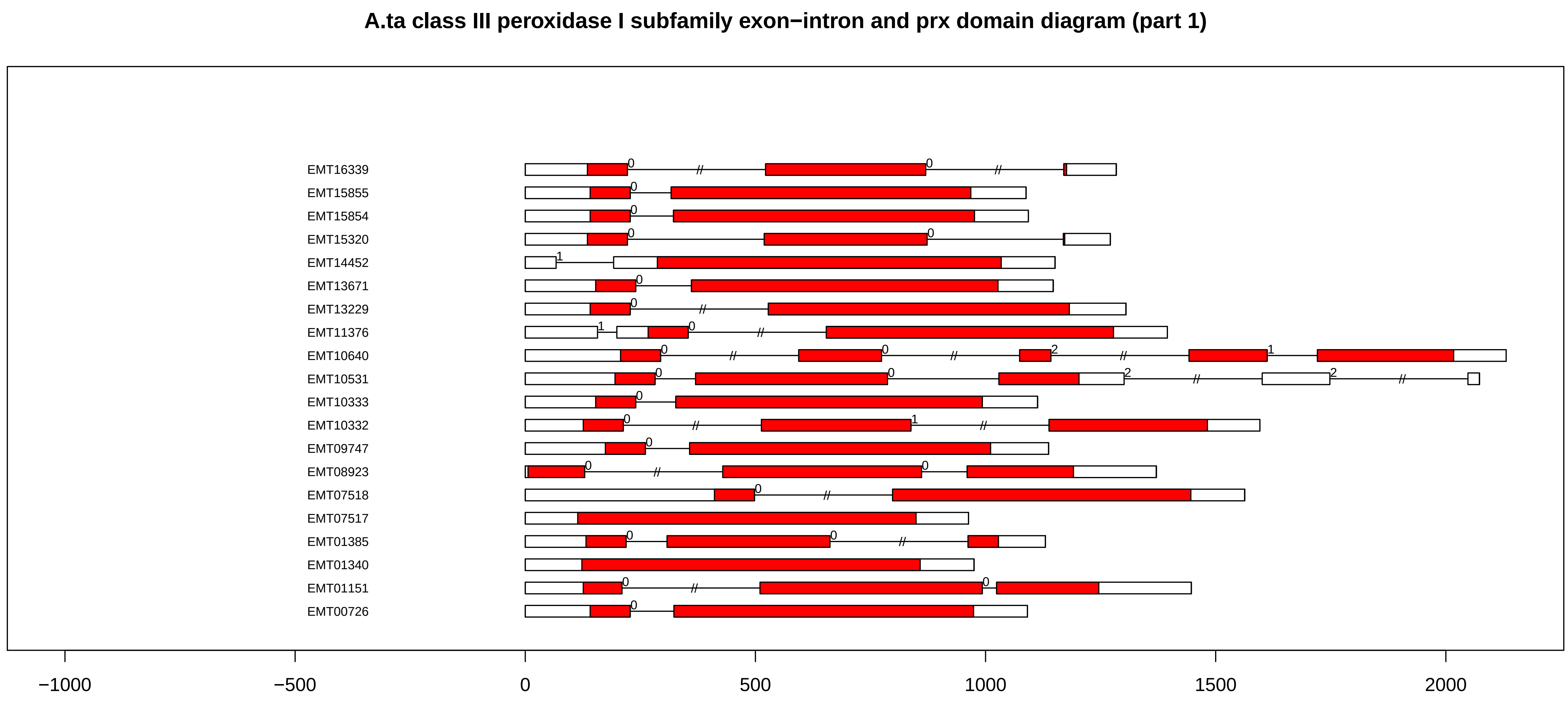
A.ta class III peroxidase I subfamily exon−intron and prx domain diagram (part 1)
EMT16339
EMT15855
EMT15854
EMT15320
EMT14452
EMT13671
EMT13229
EMT11376
EMT10640
EMT10531
EMT10333
EMT10332
EMT09747
EMT08923
EMT07518
EMT07517
EMT01385
EMT01340
EMT01151
EMT00726
−1000
−500
0
500
1000
1500
2000

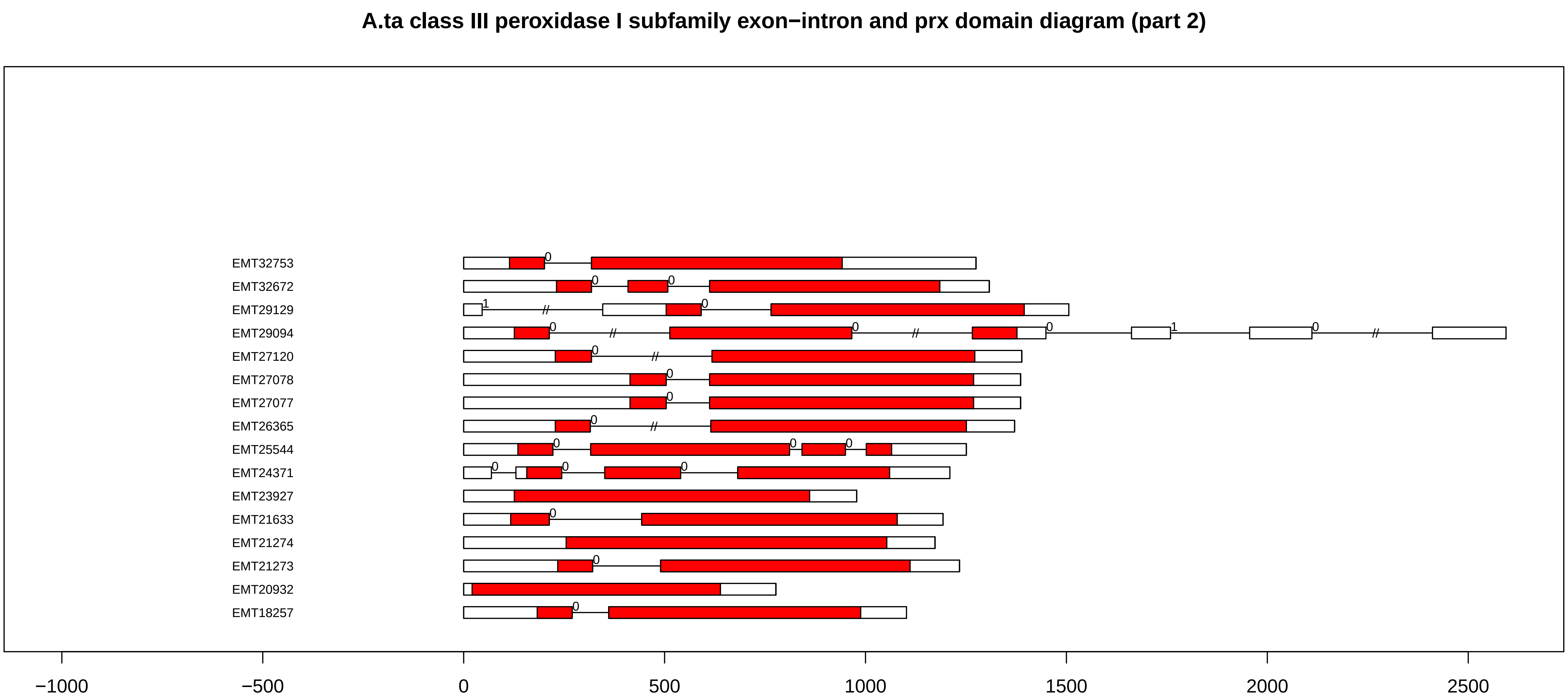

A.ta class III peroxidase I subfamily exon−intron and prx domain diagram (part 2)
EMT32753
EMT32672
EMT29129
EMT29094
EMT27120
EMT27078
EMT27077
EMT26365
EMT25544
EMT24371
EMT23927
EMT21633
EMT21274
EMT21273
EMT20932
EMT18257
−1000
−500
0
500
1000
1500
2000
2500

A.ta class III peroxidase II subfamily exon−intron and prx domain diagram (all)

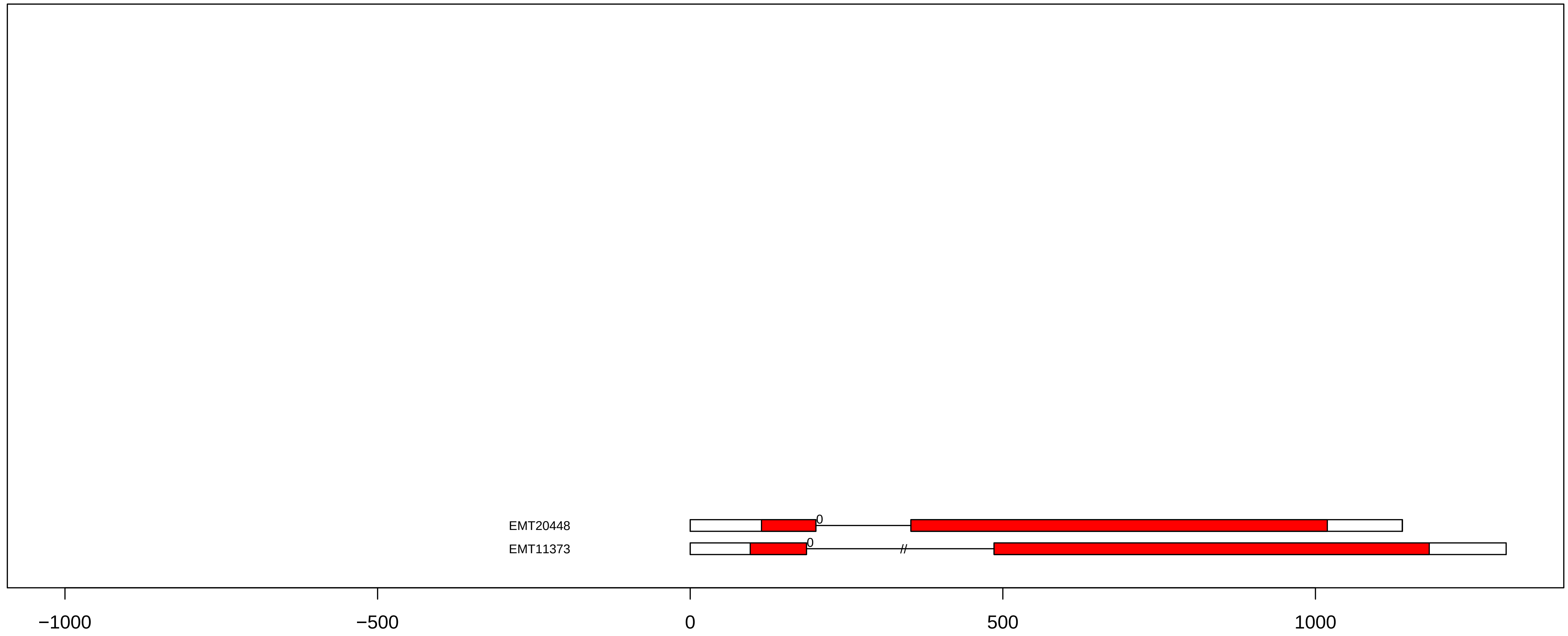

# A.ta class III peroxidase V subfamily exon−intron and prx domain diagram (part 1)

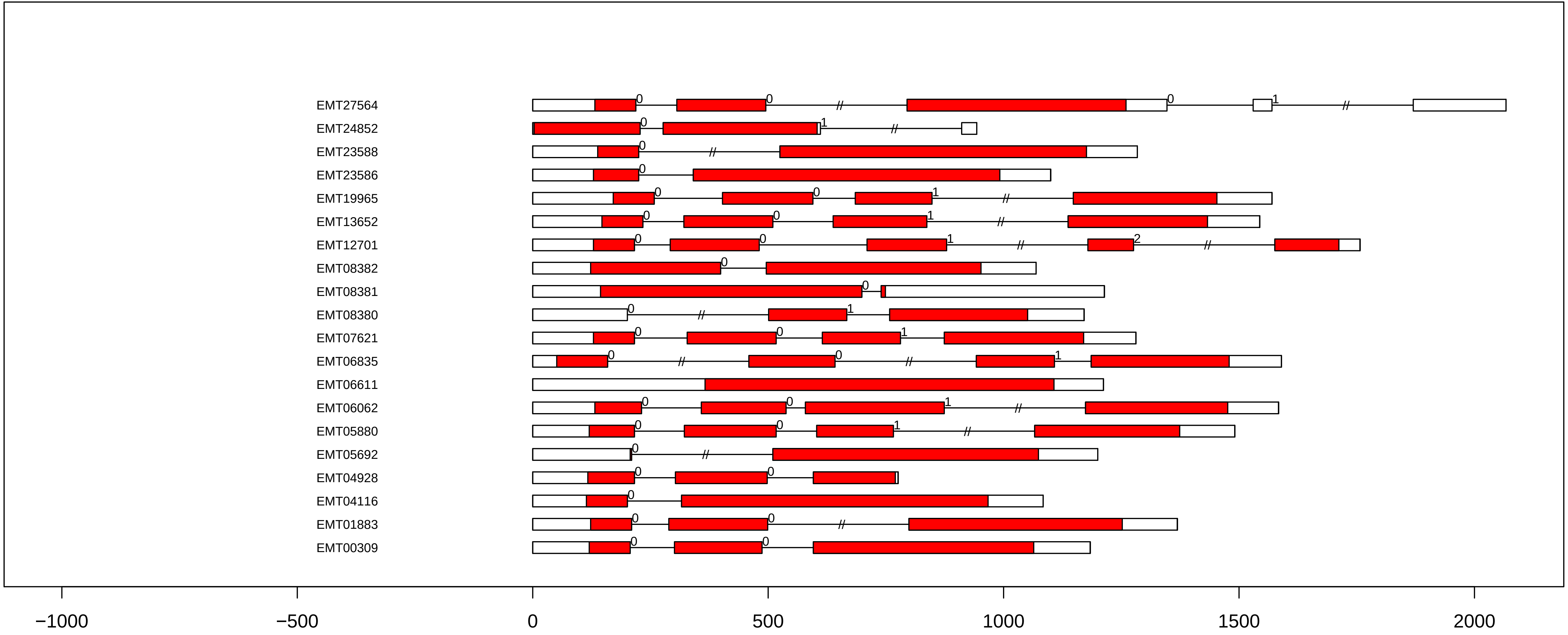

## A.ta class III peroxidase V subfamily exon−intron and prx domain diagram (part 2)

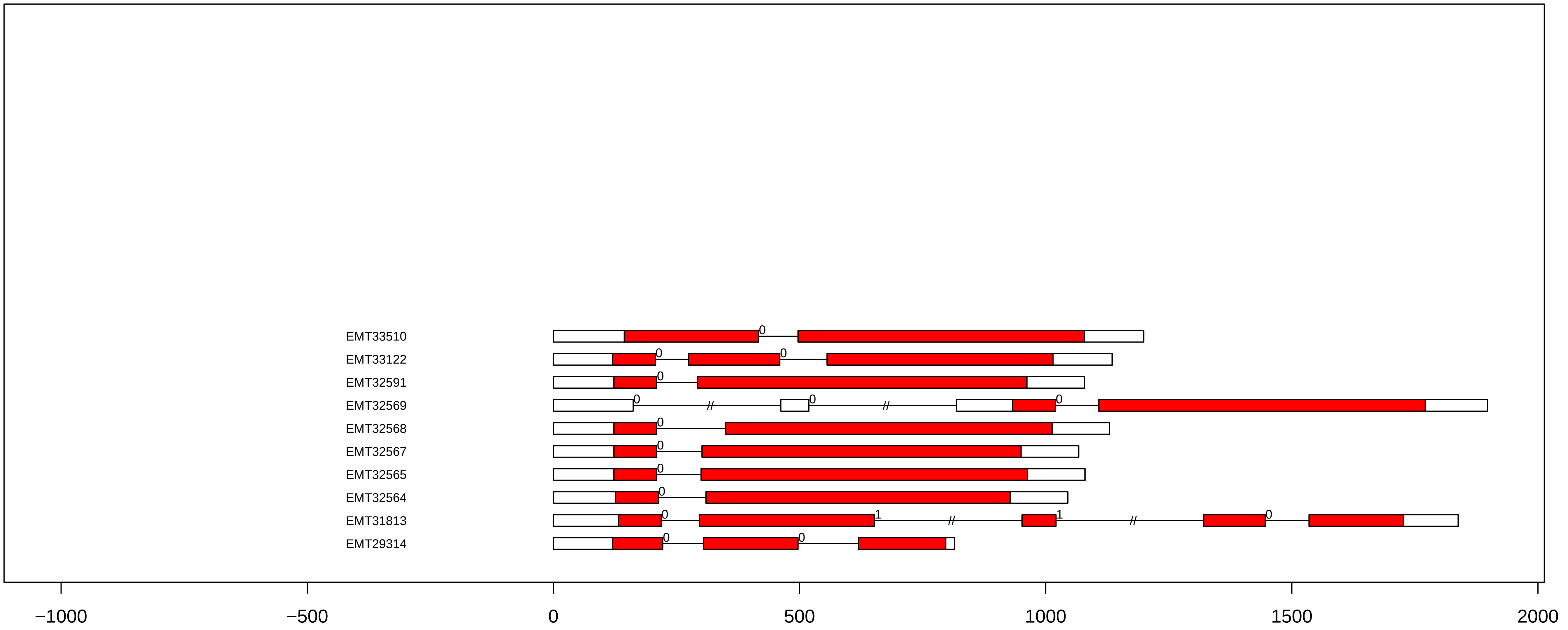

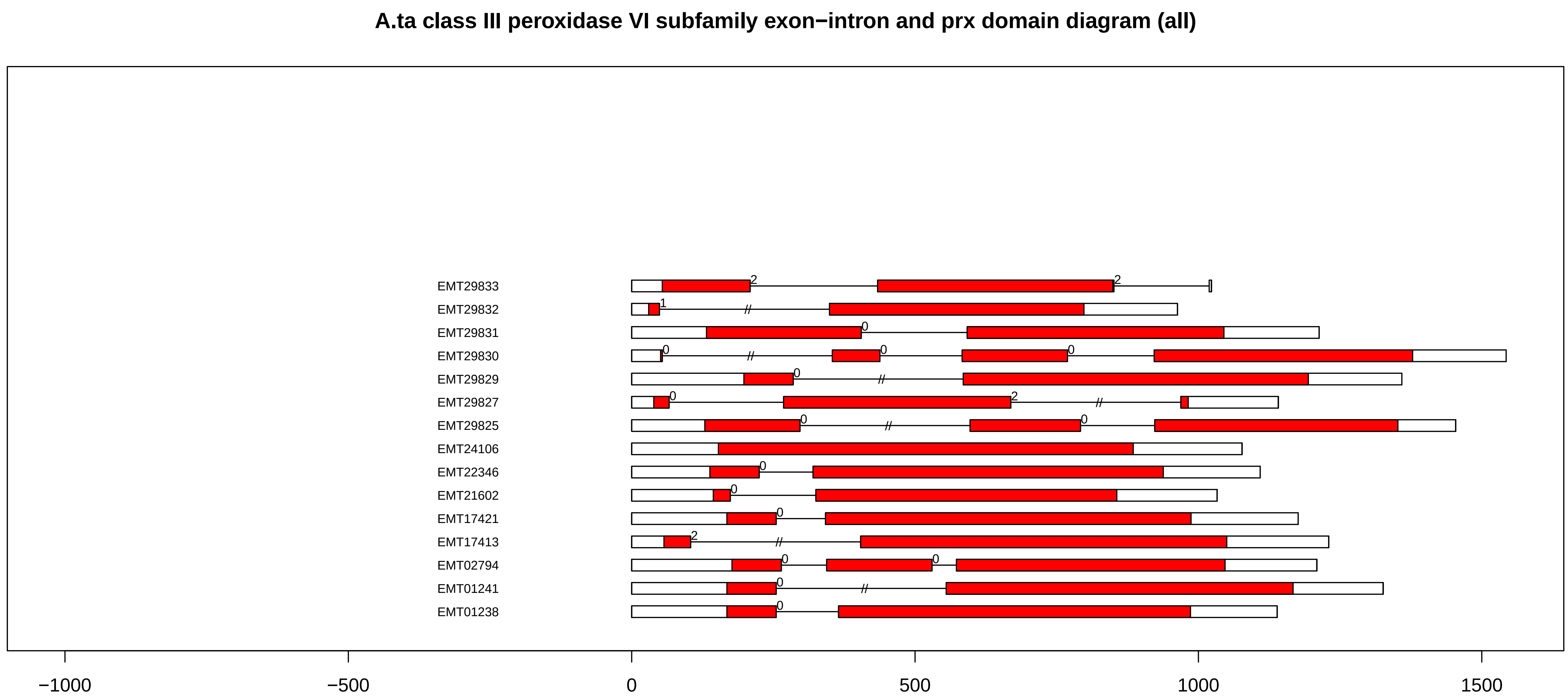

A.ta class III peroxidase VI subfamily exon−intron and prx domain diagram (all)
EMT29833
EMT29832
EMT29831
EMT29830
EMT29829
EMT29827
EMT29825
EMT24106
EMT22346
EMT21602
EMT17421
EMT17413
EMT02794
EMT01241
EMT01238
−1000
−500
0
500
1000
1500

# A.ta class III peroxidase VII subfamily exon−intron and prx domain diagram (all)

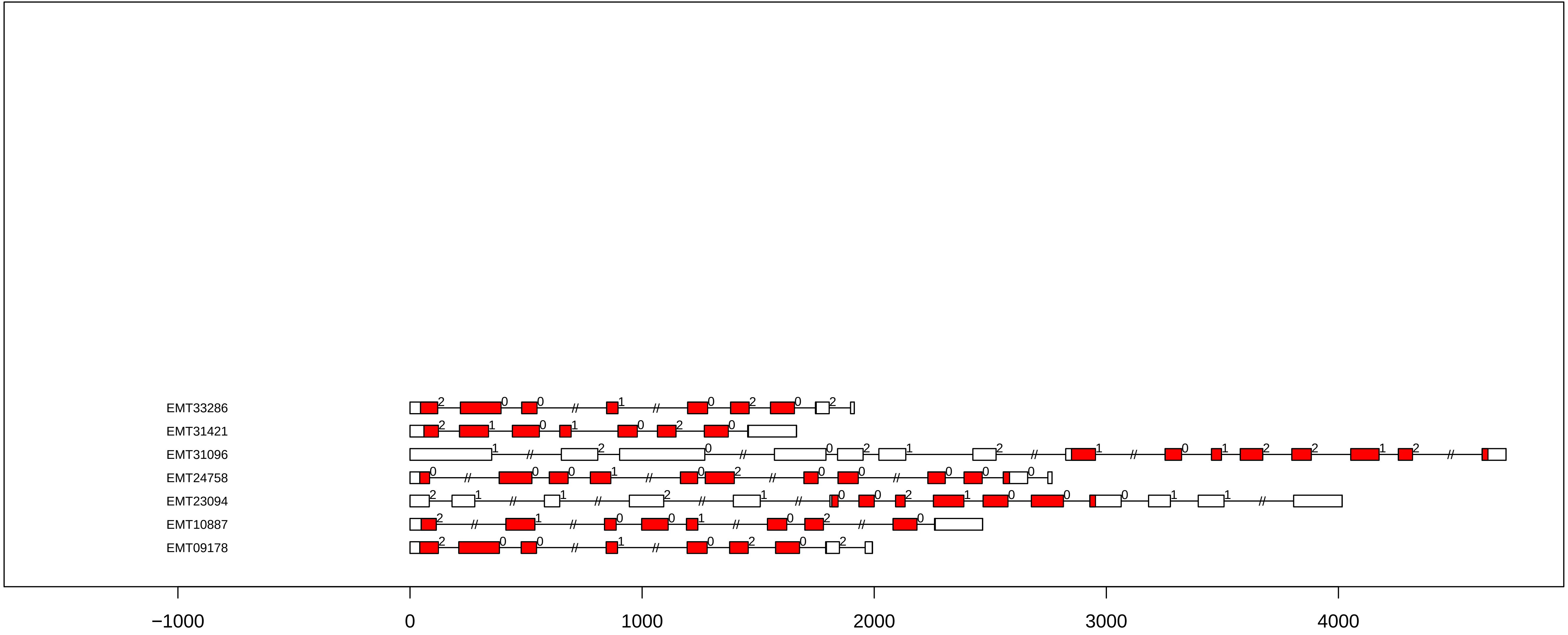

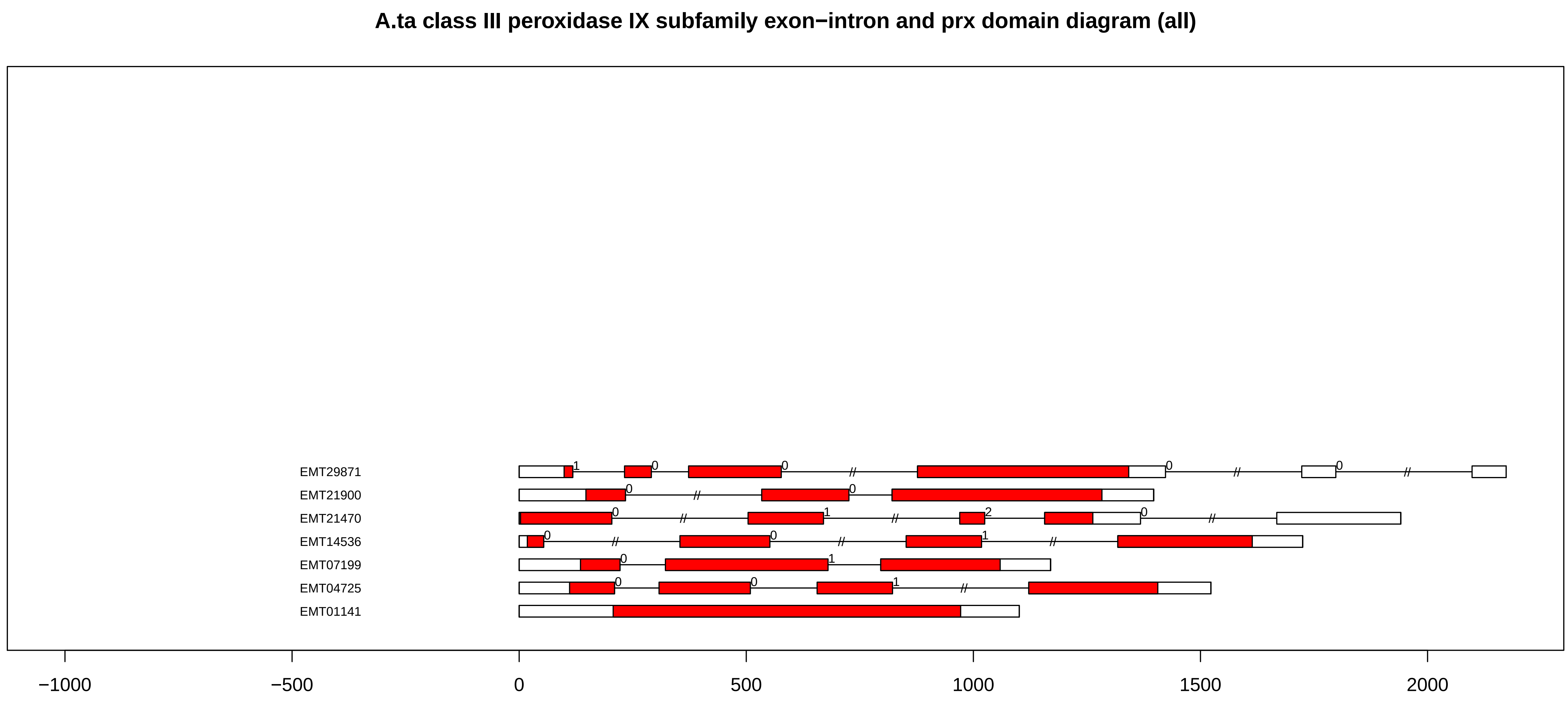
A.ta class III peroxidase IX subfamily exon−intron and prx domain diagram (all)
EMT29871
EMT21900
EMT21470
EMT14536
EMT07199
EMT04725
EMT01141
−1000
−500
0
500
1000
1500
2000

## A.ta class III peroxidase X subfamily exon−intron and prx domain diagram (all)

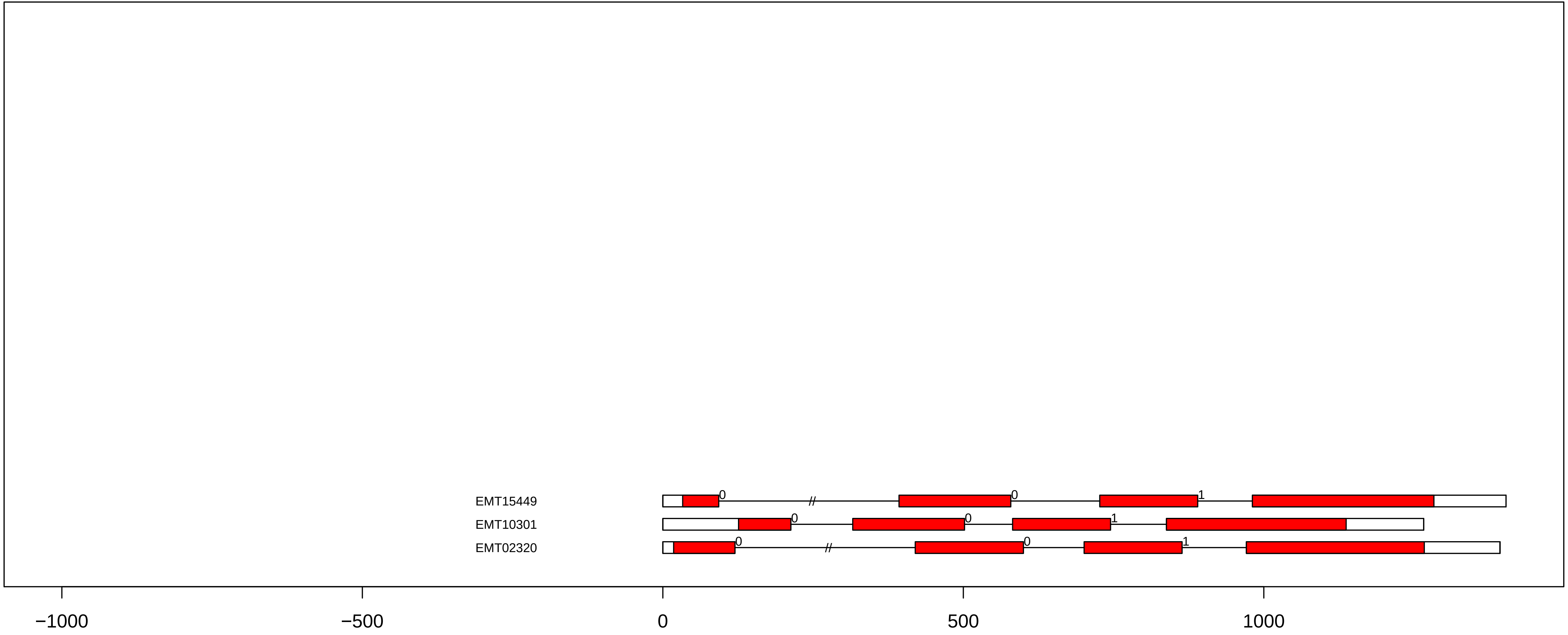

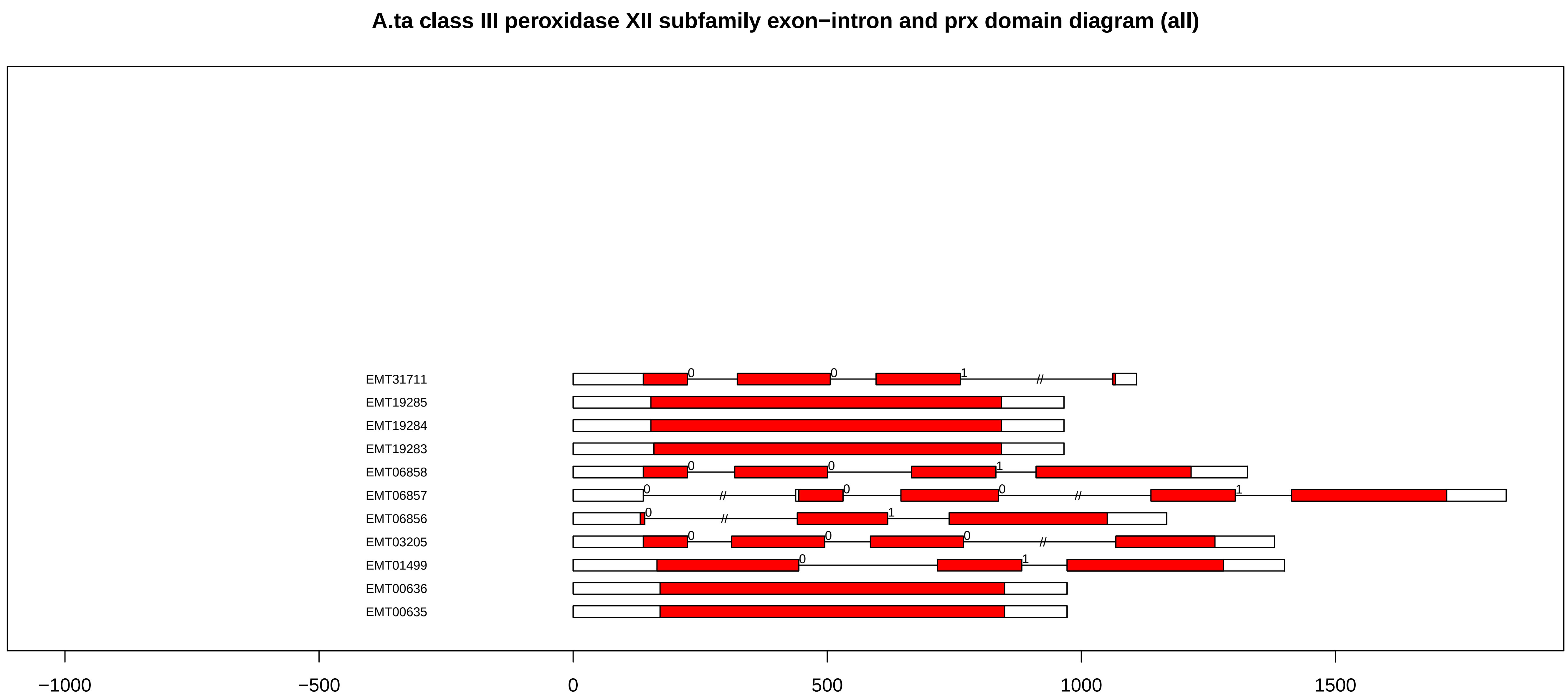

A.ta class III peroxidase XII subfamily exon−intron and prx domain diagram (all)
EMT31711
EMT19285
EMT19284
EMT19283
EMT06858
EMT06857
EMT06856
EMT03205
EMT01499
EMT00636
EMT00635
−1000
−500
0
500
1000
1500

A.ta class III peroxidase XIV subfamily exon−intron and prx domain diagram (all)

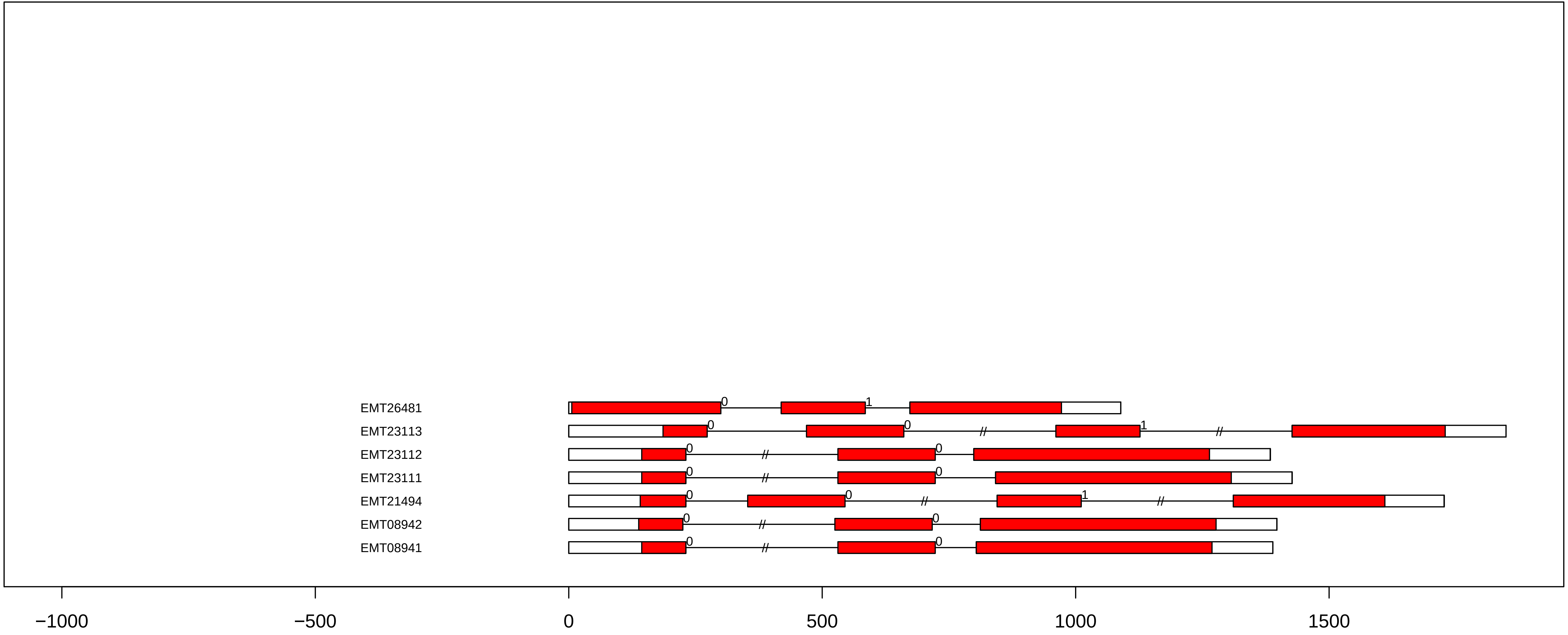

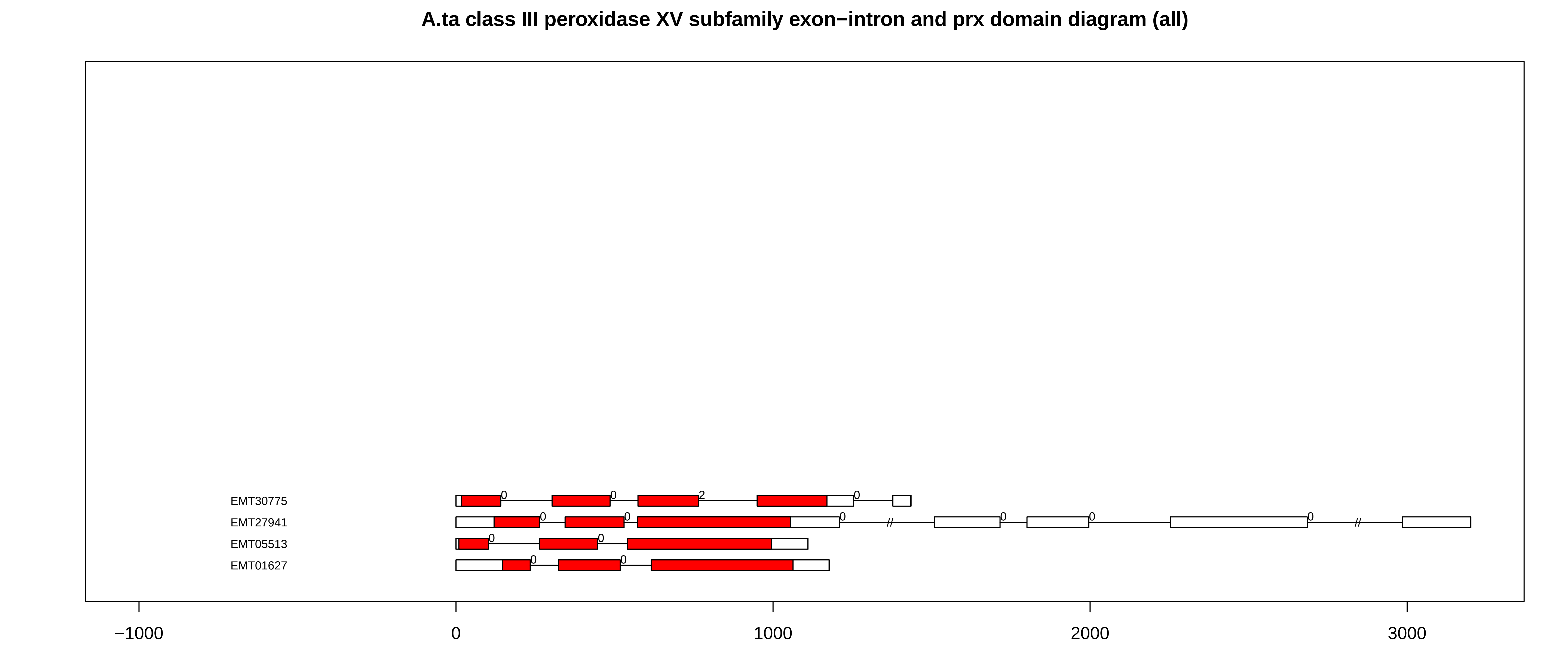
A.ta class III peroxidase XV subfamily exon−intron and prx domain diagram (all)
EMT30775
EMT27941
EMT05513
EMT01627
0
0
2
0
0
0
0
0
0
0
0
0
0
0
0
−1000
0
1000
2000
3000

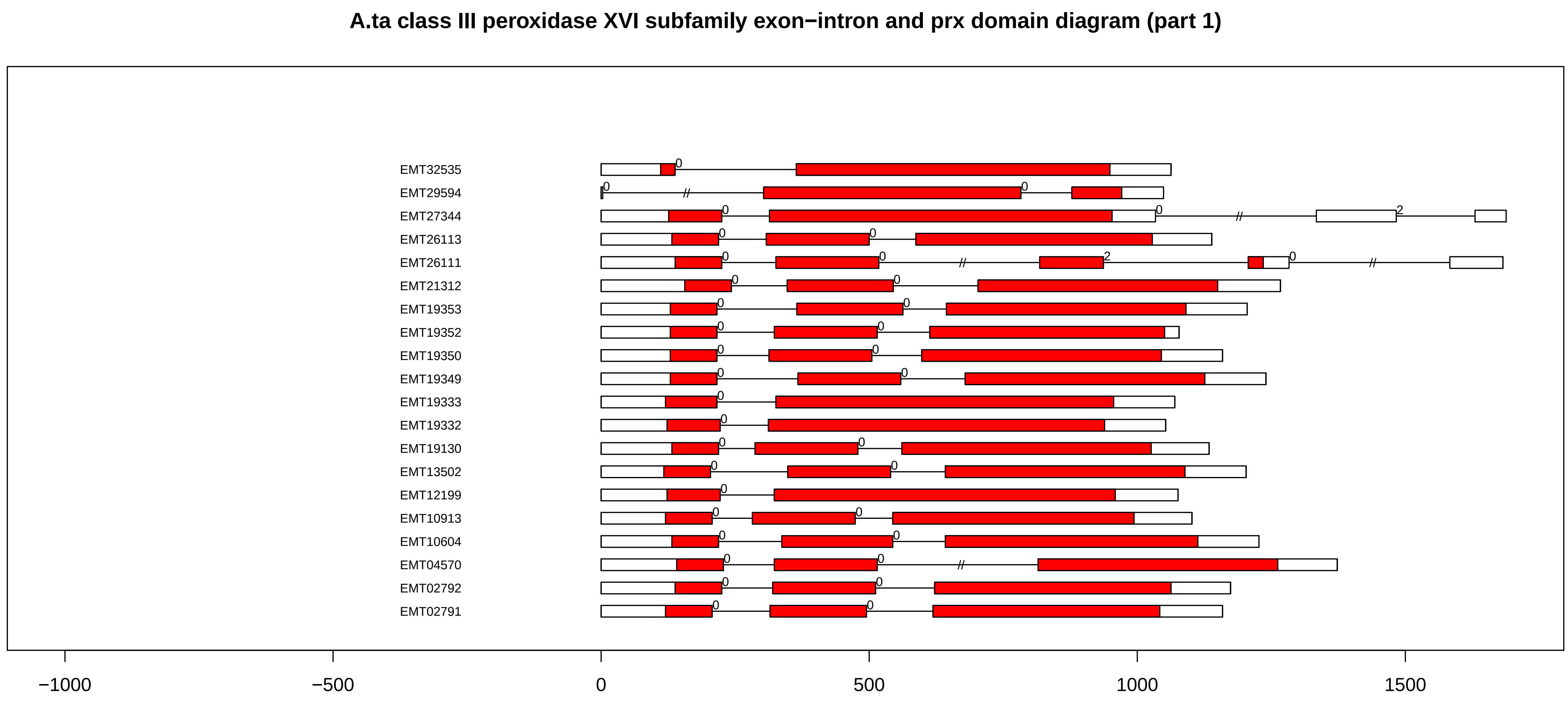
A.ta class III peroxidase XVI subfamily exon−intron and prx domain diagram (part 1)
EMT32535
EMT29594
EMT27344
EMT26113
EMT26111
EMT21312
EMT19353
EMT19352
EMT19350
EMT19349
EMT19333
EMT19332
EMT19130
EMT13502
EMT12199
EMT10913
EMT10604
EMT04570
EMT02792
EMT02791
−1000
−500
0
500
1000
1500

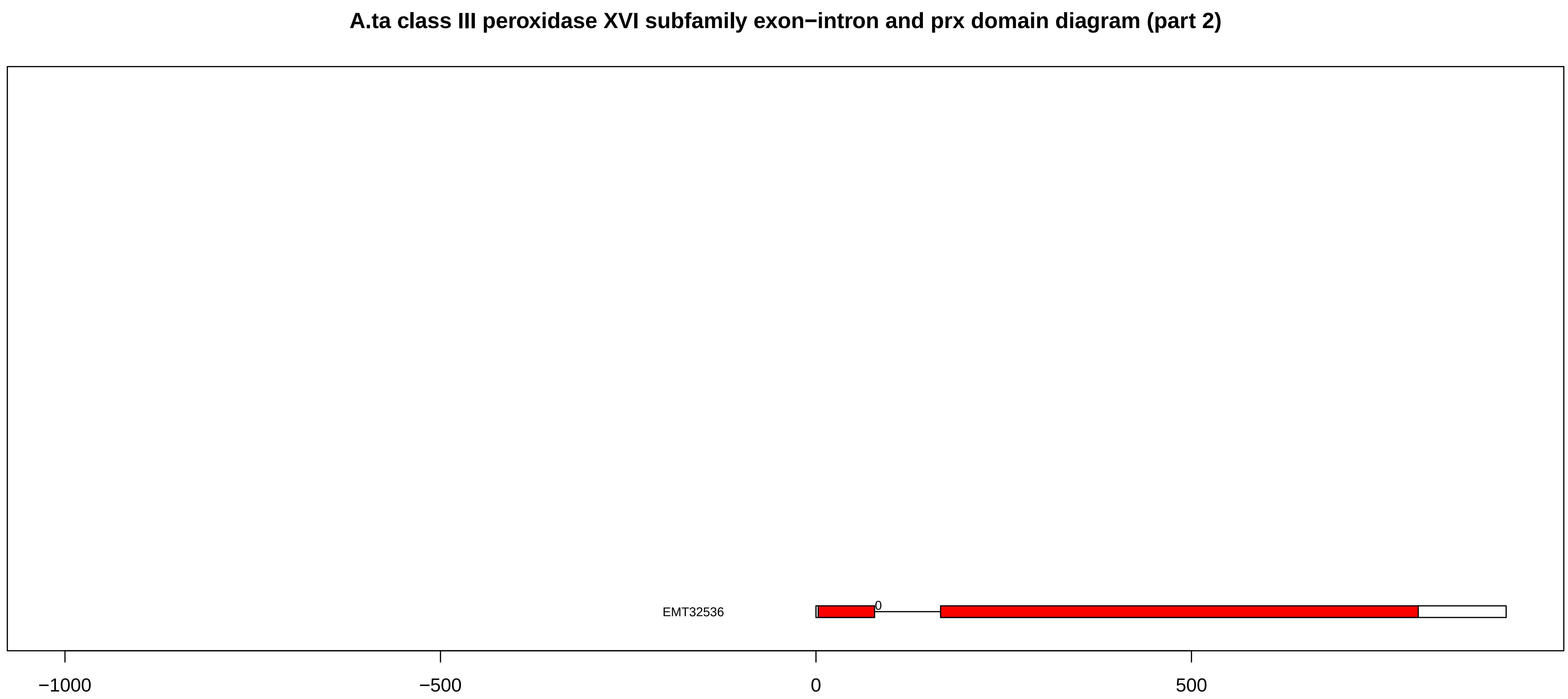
A.ta class III peroxidase XVI subfamily exon−intron and prx domain diagram (part 2)
EMT32536
0
−1000
−500
0
500

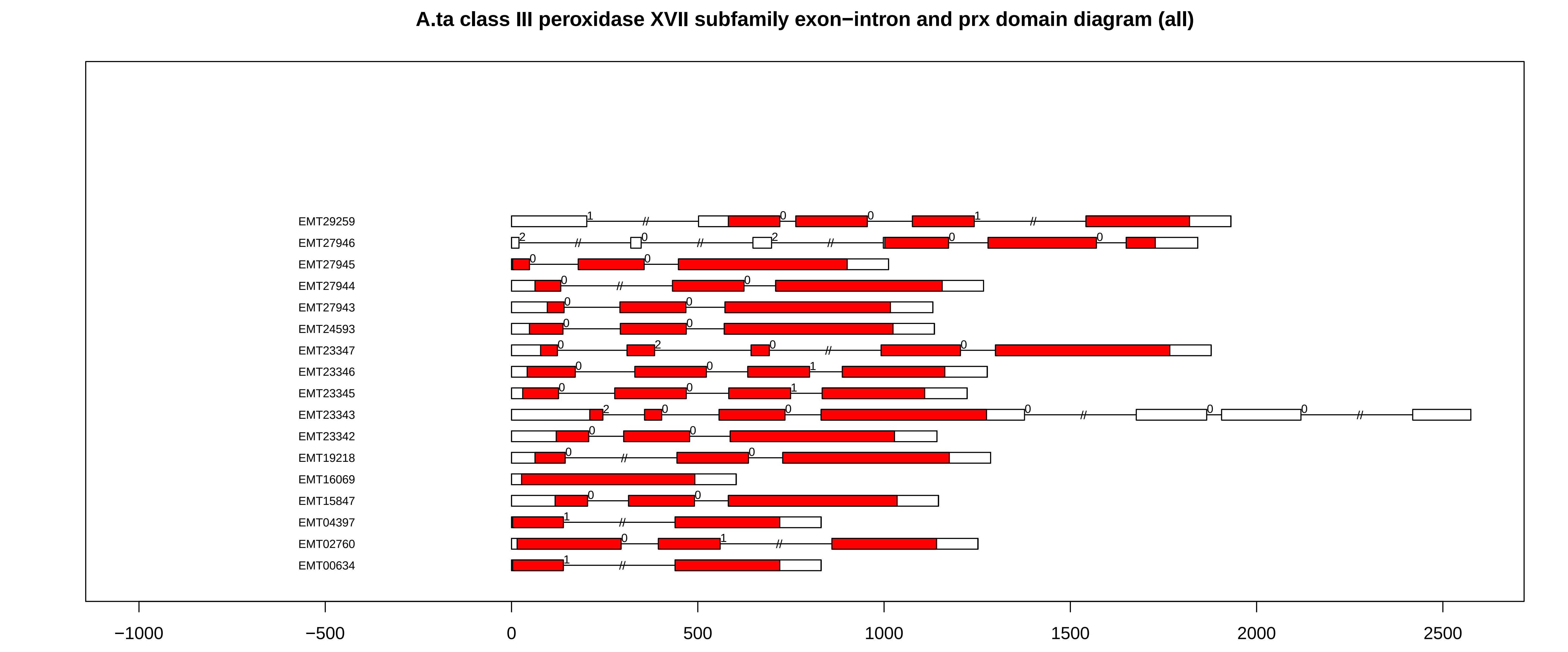

A.ta class III peroxidase XVII subfamily exon−intron and prx domain diagram (all)
EMT29259
EMT27946
EMT27945
EMT27944
EMT27943
EMT24593
EMT23347
EMT23346
EMT23345
EMT23343
EMT23342
EMT19218
EMT16069
EMT15847
EMT04397
EMT02760
EMT00634
−1000
−500
0
500
1000
1500
2000
2500

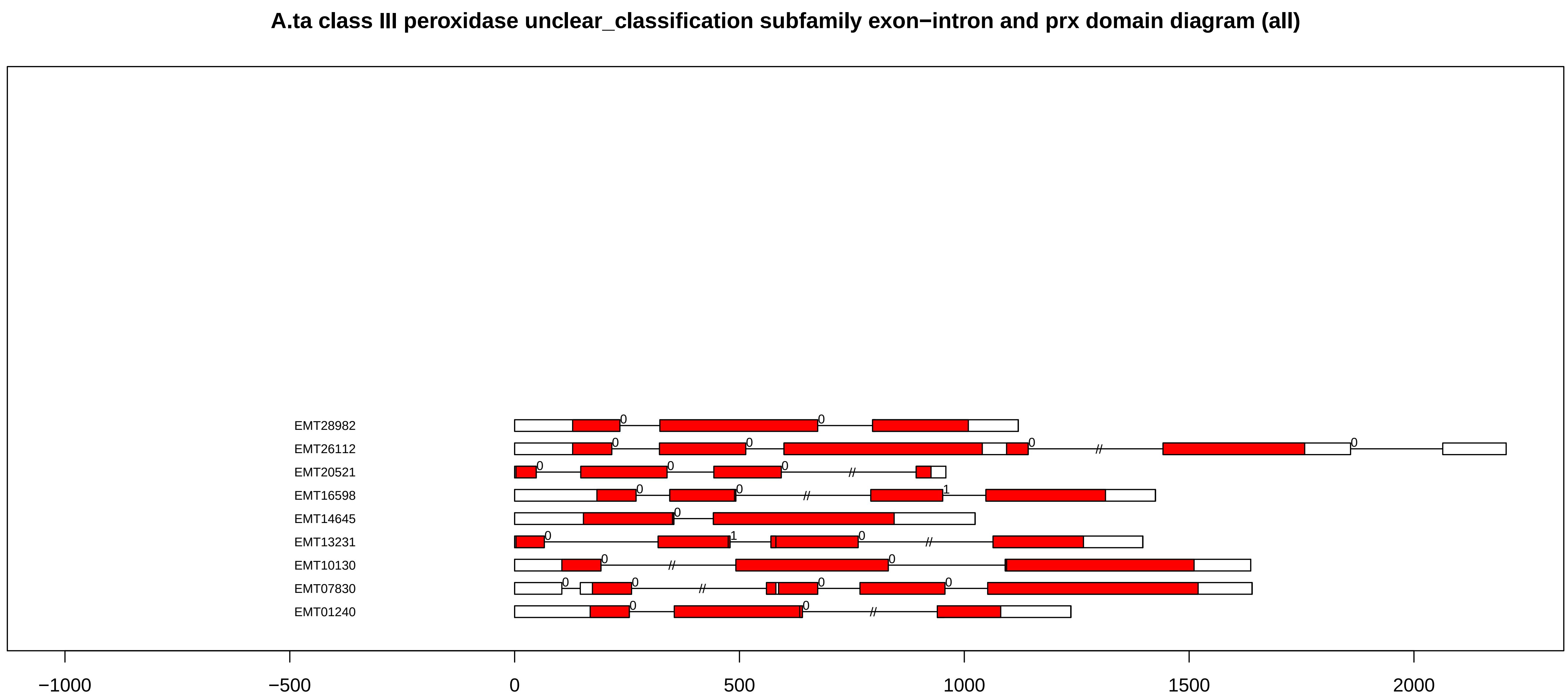

A.ta class III peroxidase unclear_classification subfamily exon−intron and prx domain diagram (all)
EMT28982
EMT26112
EMT20521
EMT16598
EMT14645
EMT13231
EMT10130
EMT07830
EMT01240
−1000
−500
0
500
1000
1500
2000

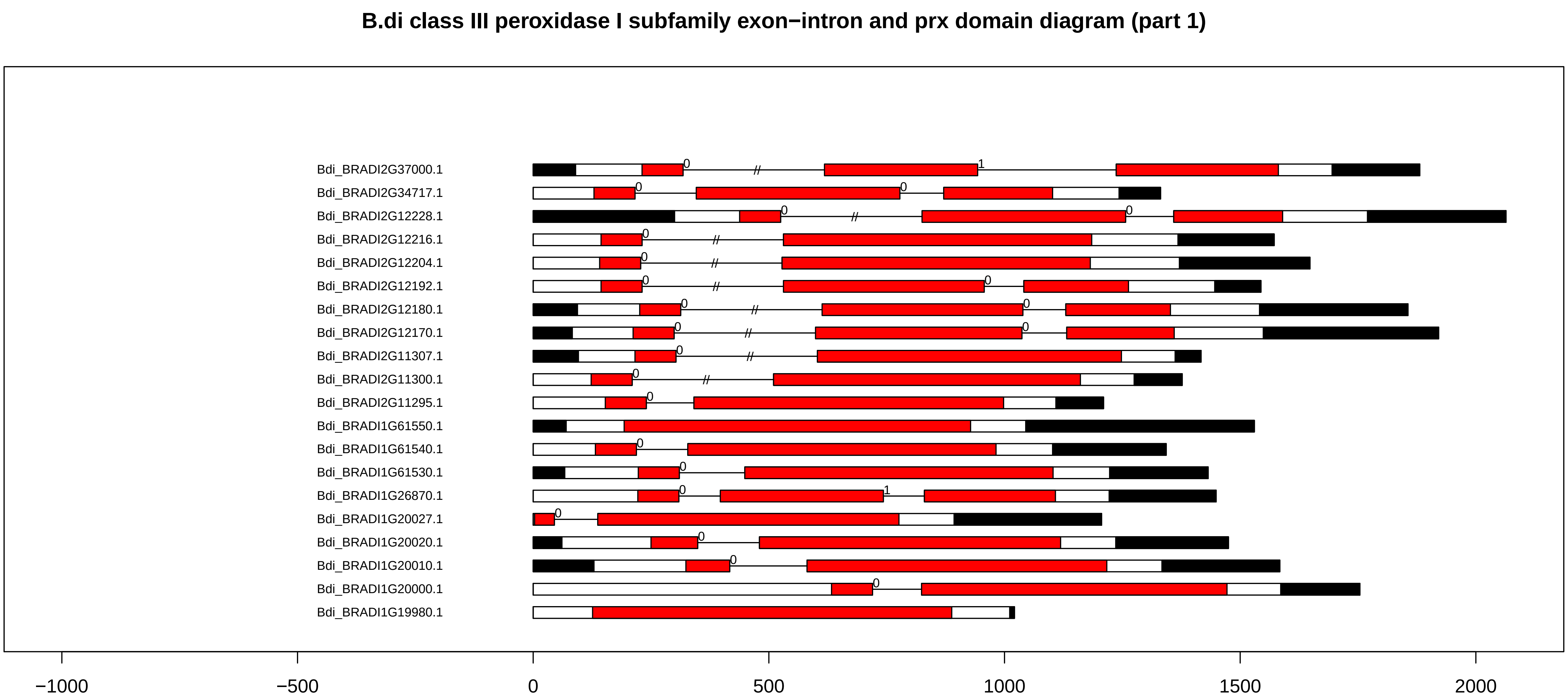
B.di class III peroxidase I subfamily exon−intron and prx domain diagram (part 1)
Bdi_BRADI2G37000.1
Bdi_BRADI2G34717.1
Bdi_BRADI2G12228.1
Bdi_BRADI2G12216.1
Bdi_BRADI2G12204.1
Bdi_BRADI2G12192.1
Bdi_BRADI2G12180.1
Bdi_BRADI2G12170.1
Bdi_BRADI2G11307.1
Bdi_BRADI2G11300.1
Bdi_BRADI2G11295.1
Bdi_BRADI1G61550.1
Bdi_BRADI1G61540.1
Bdi_BRADI1G61530.1
Bdi_BRADI1G26870.1
Bdi_BRADI1G20027.1
Bdi_BRADI1G20020.1
Bdi_BRADI1G20010.1
Bdi_BRADI1G20000.1
Bdi_BRADI1G19980.1
−1000
−500
0
500
1000
1500
2000

B.di class III peroxidase I subfamily exon−intron and prx domain diagram (part 2)

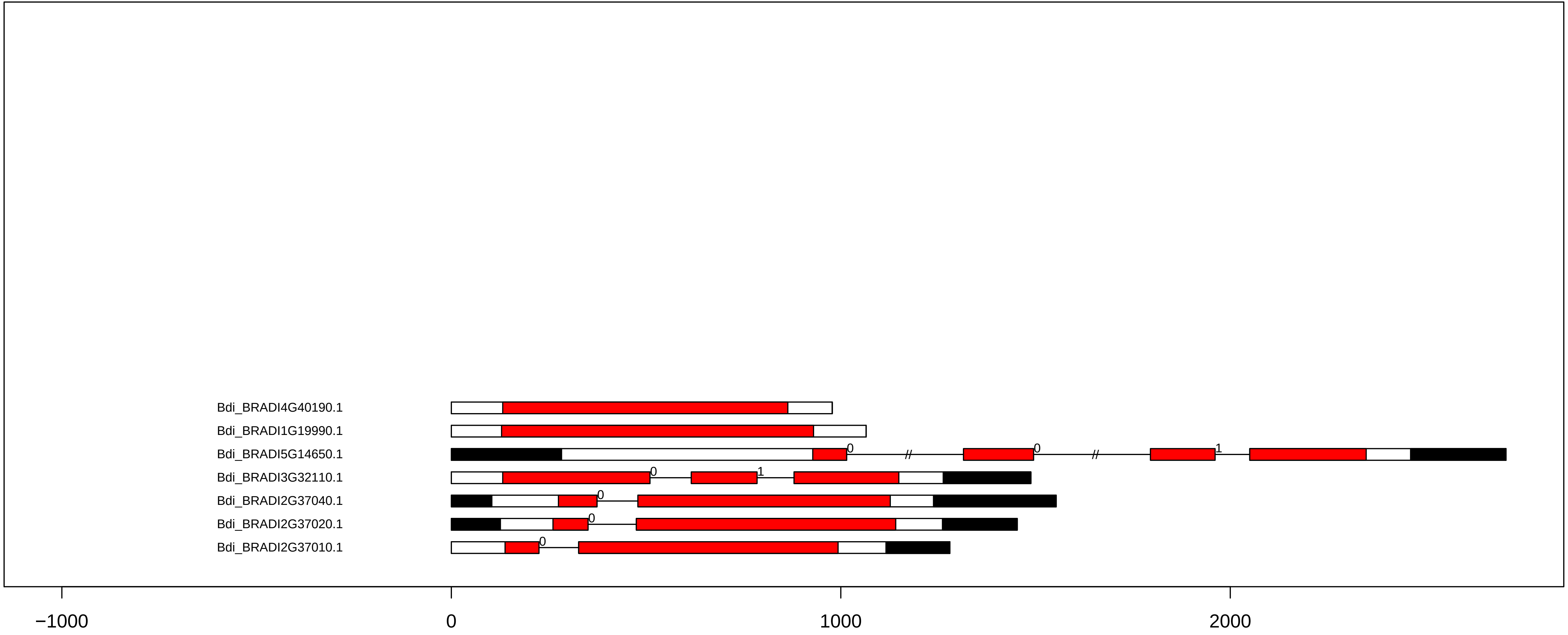

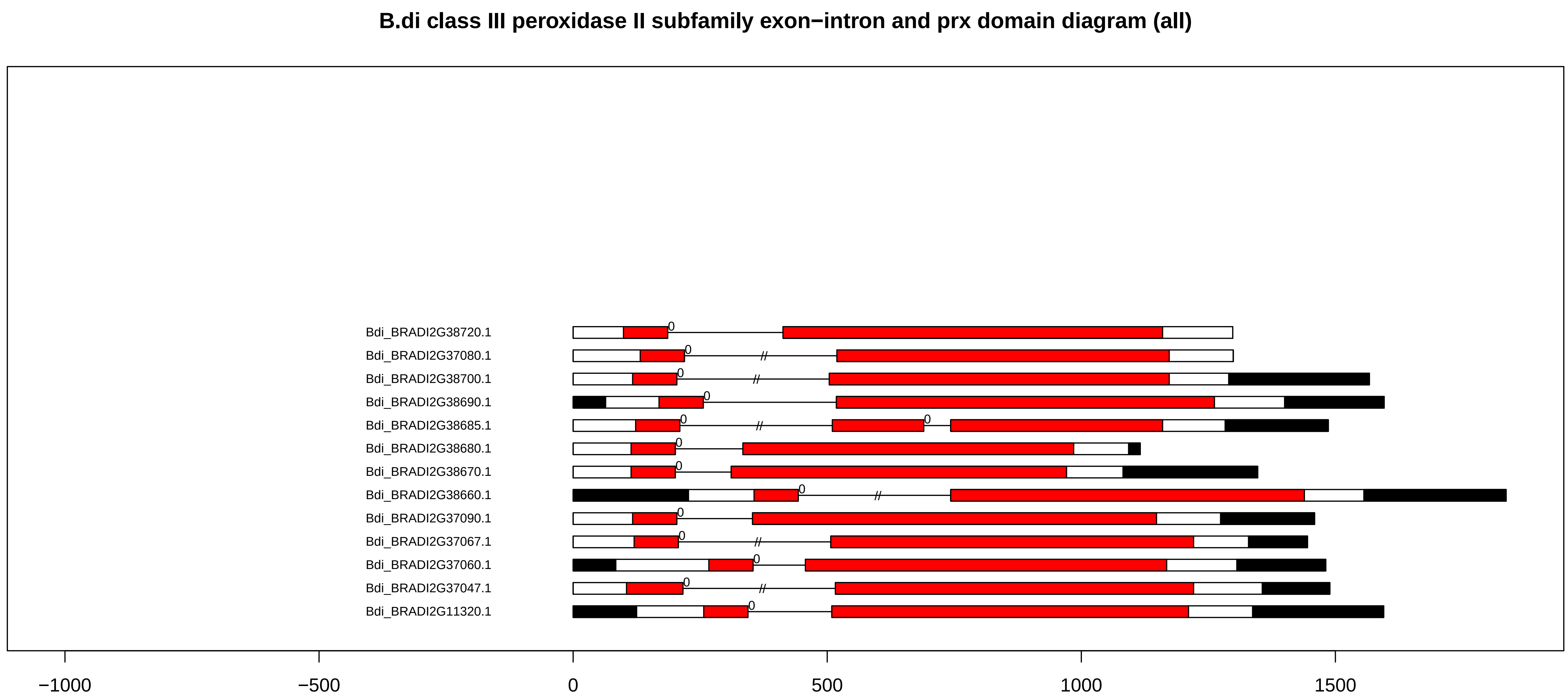

B.di class III peroxidase II subfamily exon−intron and prx domain diagram (all)
Bdi_BRADI2G38720.1
Bdi_BRADI2G37080.1
Bdi_BRADI2G38700.1
Bdi_BRADI2G38690.1
Bdi_BRADI2G38685.1
Bdi_BRADI2G38680.1
Bdi_BRADI2G38670.1
Bdi_BRADI2G38660.1
Bdi_BRADI2G37090.1
Bdi_BRADI2G37067.1
Bdi_BRADI2G37060.1
Bdi_BRADI2G37047.1
Bdi_BRADI2G11320.1
−1000
−500
0
500
1000
1500

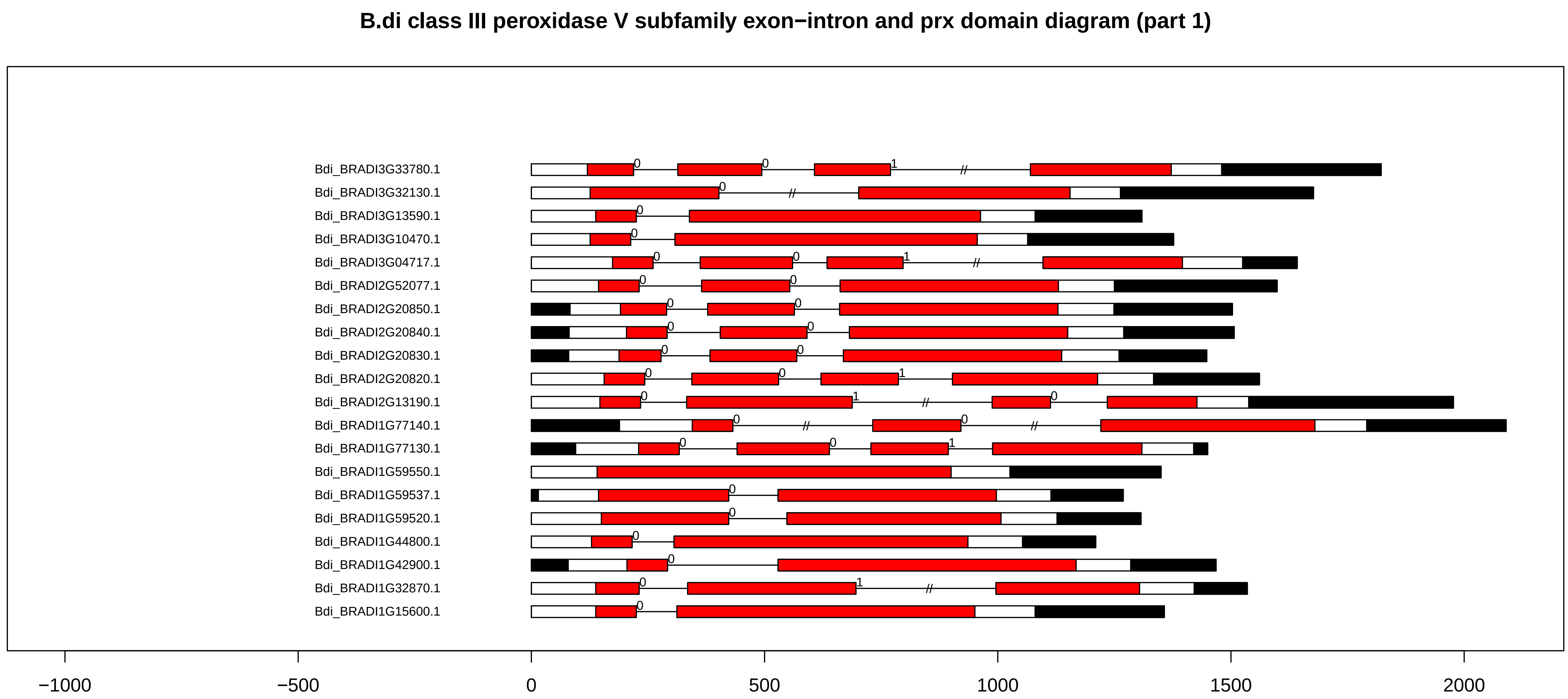
B.di class III peroxidase V subfamily exon−intron and prx domain diagram (part 1)
Bdi_BRADI3G33780.1
Bdi_BRADI3G32130.1
Bdi_BRADI3G13590.1
Bdi_BRADI3G10470.1
Bdi_BRADI3G04717.1
Bdi_BRADI2G52077.1
Bdi_BRADI2G20850.1
Bdi_BRADI2G20840.1
Bdi_BRADI2G20830.1
Bdi_BRADI2G20820.1
Bdi_BRADI2G13190.1
Bdi_BRADI1G77140.1
Bdi_BRADI1G77130.1
Bdi_BRADI1G59550.1
Bdi_BRADI1G59537.1
Bdi_BRADI1G59520.1
Bdi_BRADI1G44800.1
Bdi_BRADI1G42900.1
Bdi_BRADI1G32870.1
Bdi_BRADI1G15600.1
−1000
−500
0
500
1000
1500
2000

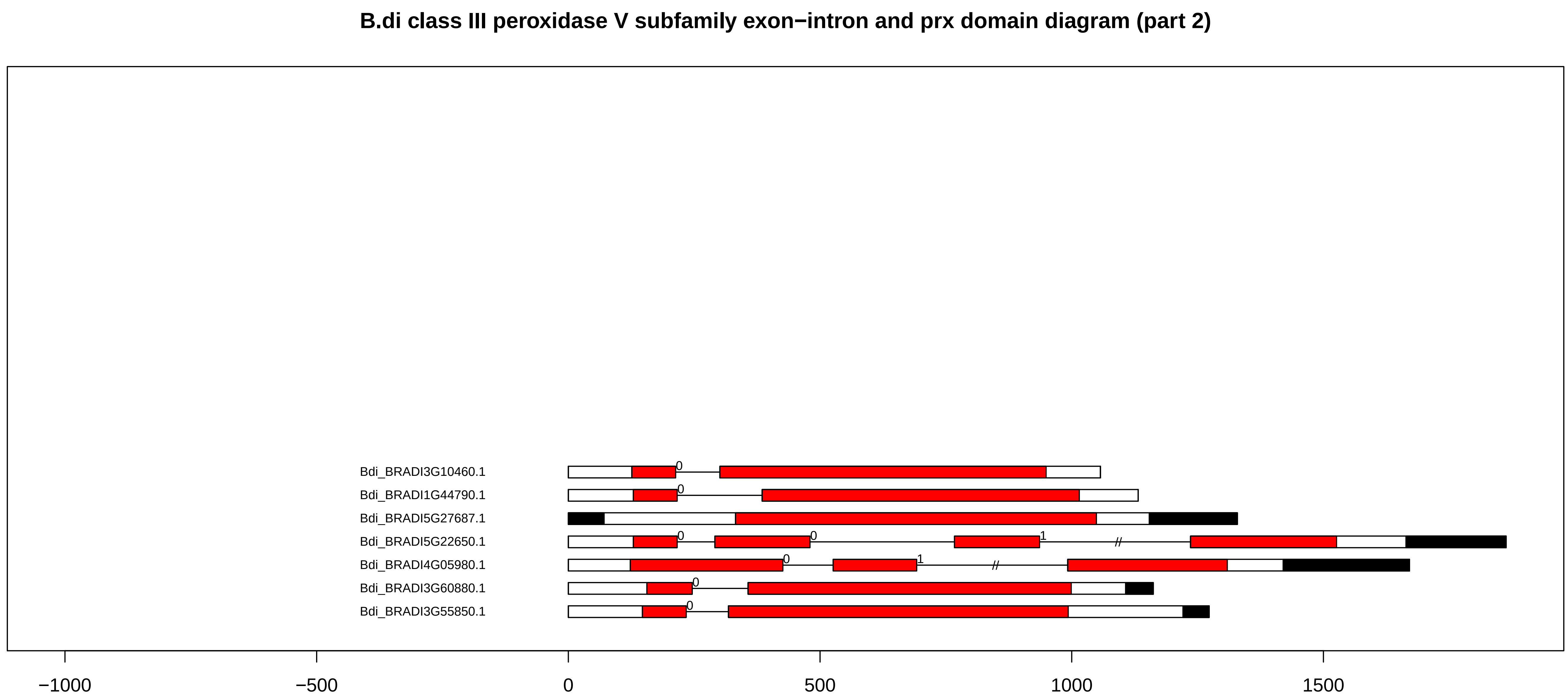

B.di class III peroxidase V subfamily exon−intron and prx domain diagram (part 2)
Bdi_BRADI3G10460.1
Bdi_BRADI1G44790.1
Bdi_BRADI5G27687.1
Bdi_BRADI5G22650.1
Bdi_BRADI4G05980.1
Bdi_BRADI3G60880.1
Bdi_BRADI3G55850.1
0
0
0
0
1
0
1
0
0
−1000
−500
0
500
1000
1500

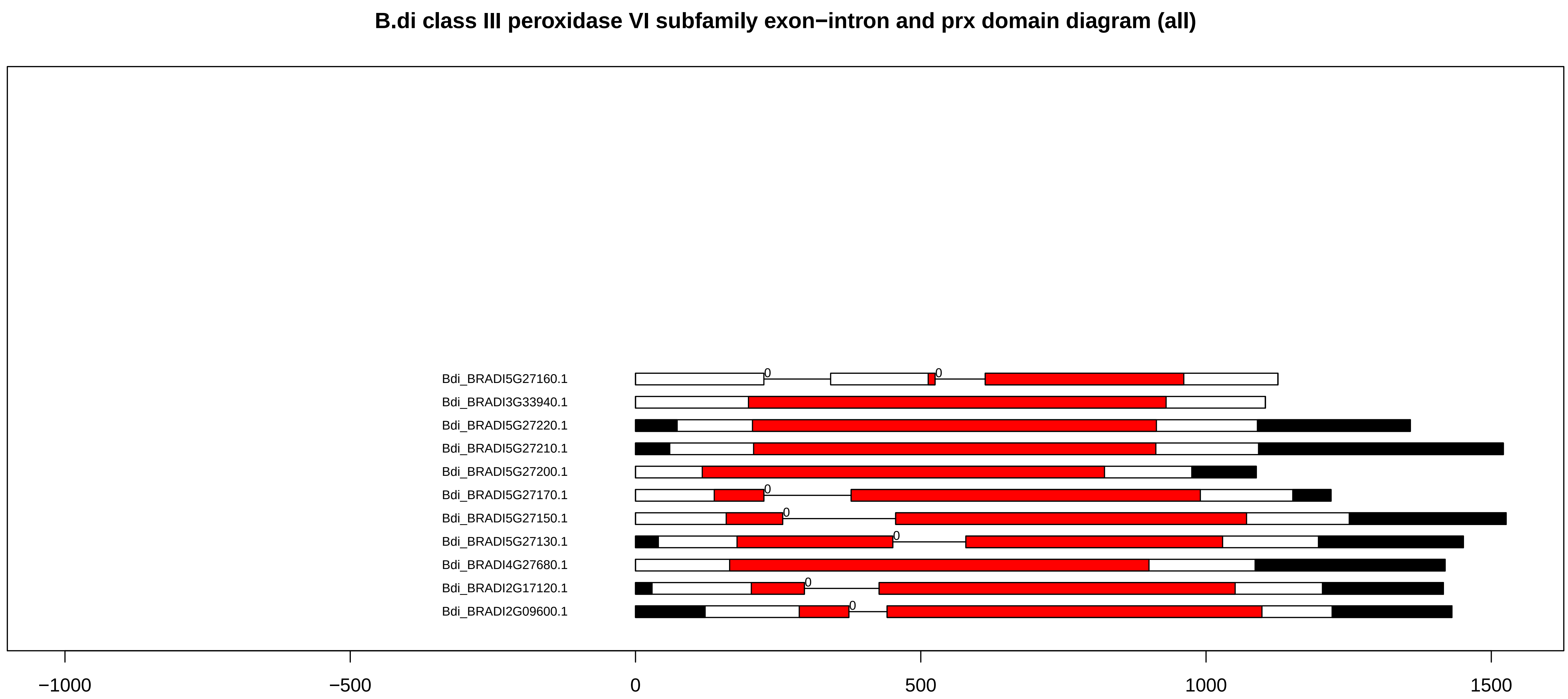

B.di class III peroxidase VI subfamily exon−intron and prx domain diagram (all)
Bdi_BRADI5G27160.1
Bdi_BRADI3G33940.1
Bdi_BRADI5G27220.1
Bdi_BRADI5G27210.1
Bdi_BRADI5G27200.1
Bdi_BRADI5G27170.1
Bdi_BRADI5G27150.1
Bdi_BRADI5G27130.1
Bdi_BRADI4G27680.1
Bdi_BRADI2G17120.1
Bdi_BRADI2G09600.1
0
0
0
0
0
0
0
−1000
−500
0
500
1000
1500

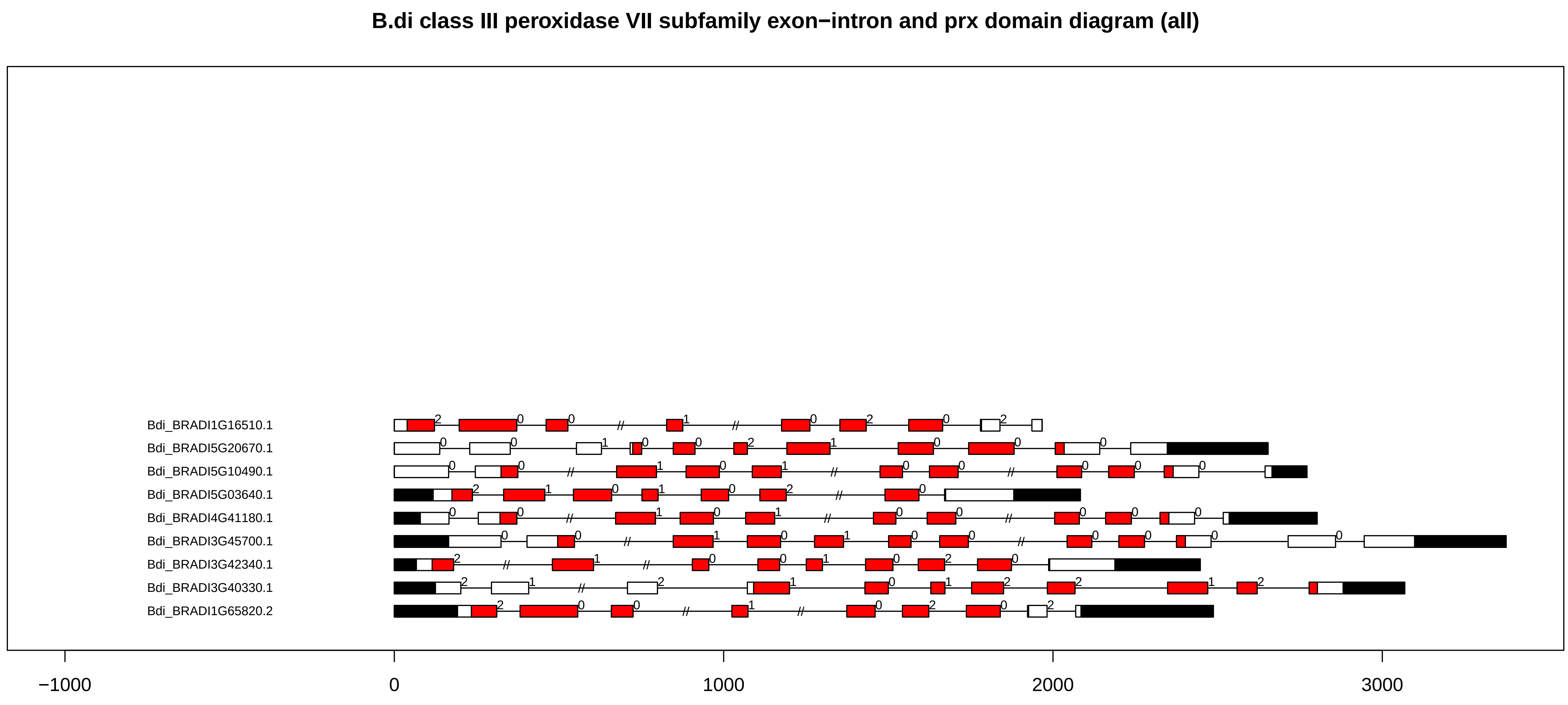
B.di class III peroxidase VII subfamily exon−intron and prx domain diagram (all)
Bdi_BRADI1G16510.1
Bdi_BRADI5G20670.1
Bdi_BRADI5G10490.1
Bdi_BRADI5G03640.1
Bdi_BRADI4G41180.1
Bdi_BRADI3G45700.1
Bdi_BRADI3G42340.1
Bdi_BRADI3G40330.1
Bdi_BRADI1G65820.2
−1000
0
1000
2000
3000

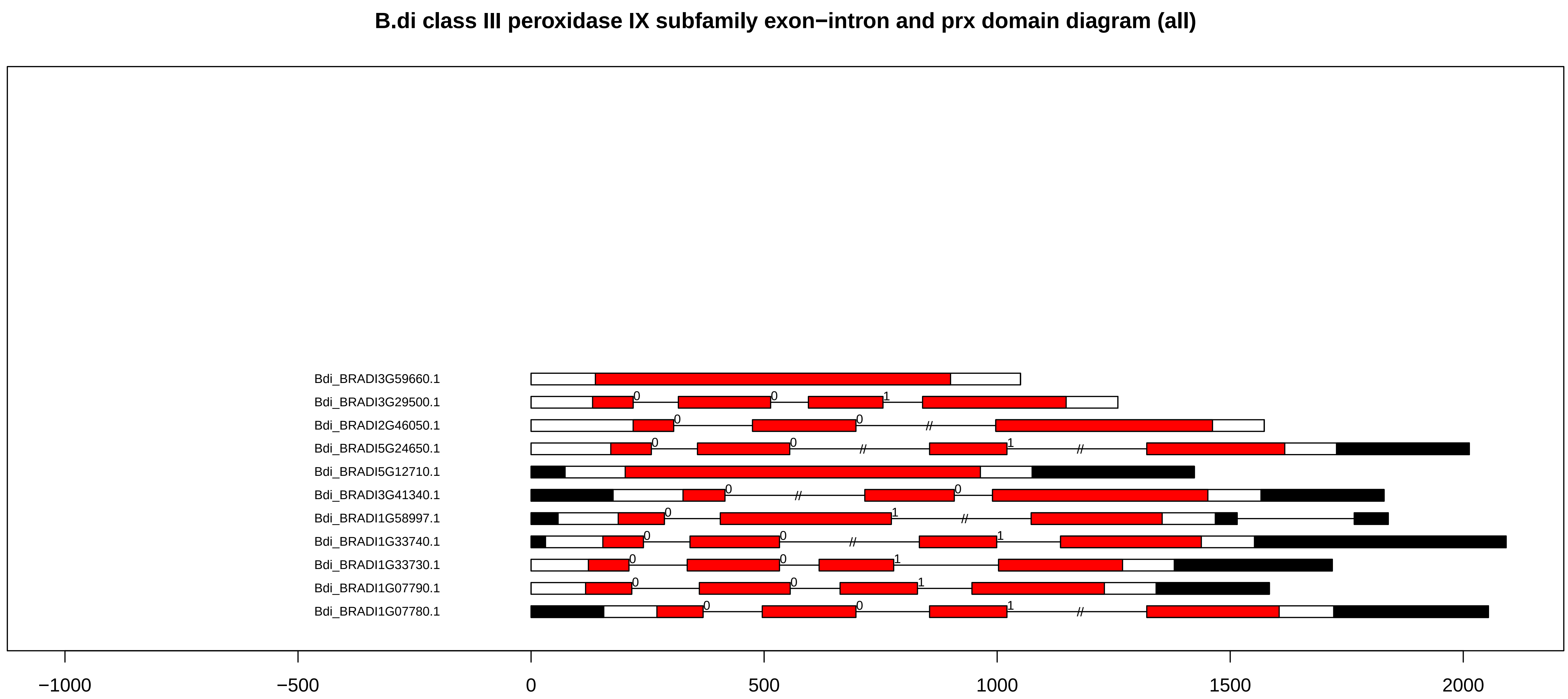

B.di class III peroxidase IX subfamily exon−intron and prx domain diagram (all)
Bdi_BRADI3G59660.1
Bdi_BRADI3G29500.1
Bdi_BRADI2G46050.1
Bdi_BRADI5G24650.1
Bdi_BRADI5G12710.1
Bdi_BRADI3G41340.1
Bdi_BRADI1G58997.1
Bdi_BRADI1G33740.1
Bdi_BRADI1G33730.1
Bdi_BRADI1G07790.1
Bdi_BRADI1G07780.1
−1000
−500
0
500
1000
1500
2000

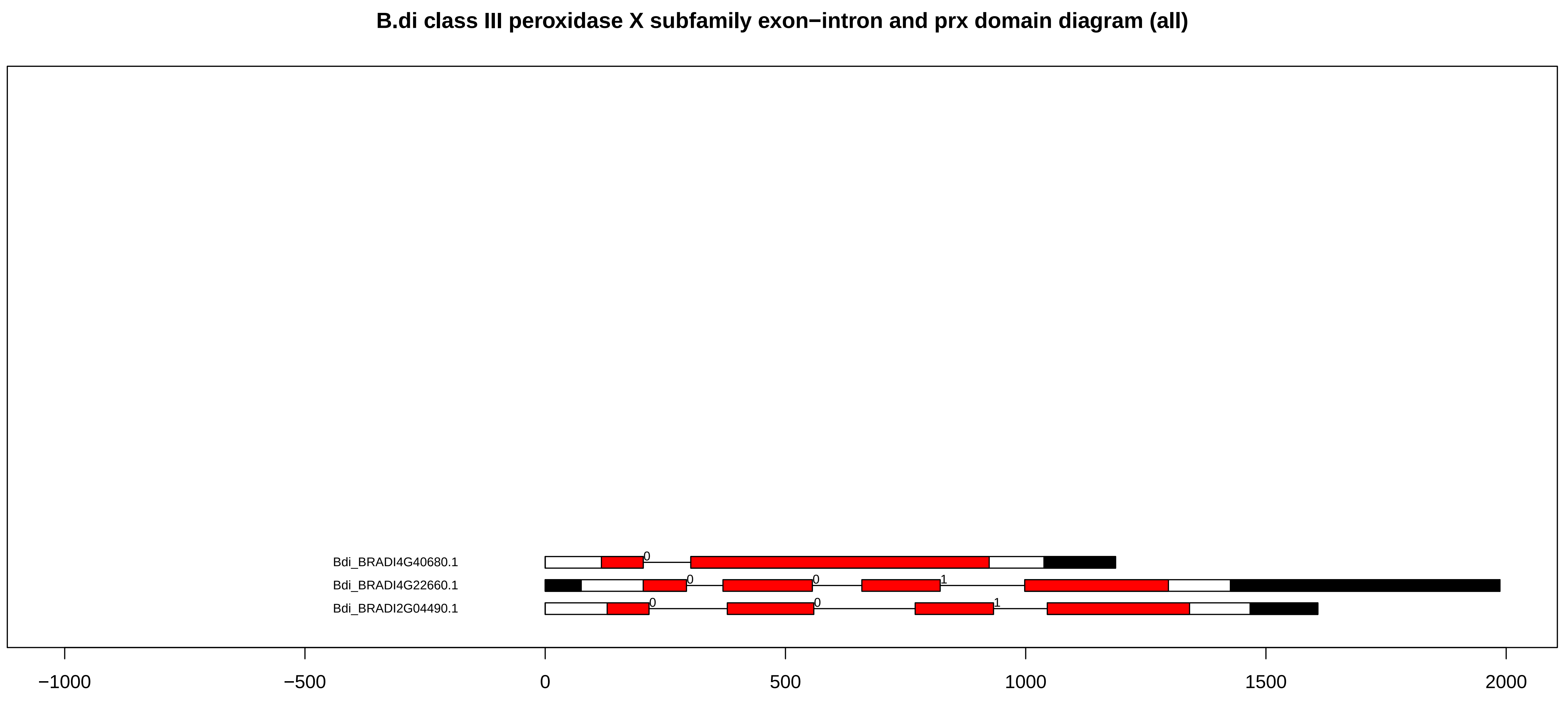
B.di class III peroxidase X subfamily exon−intron and prx domain diagram (all)
Bdi_BRADI4G40680.1
Bdi_BRADI4G22660.1
Bdi_BRADI2G04490.1
0
0
0
1
0
0
1
−1000
−500
0
500
1000
1500
2000

B.di class III peroxidase XII subfamily exon−intron and prx domain diagram (all)

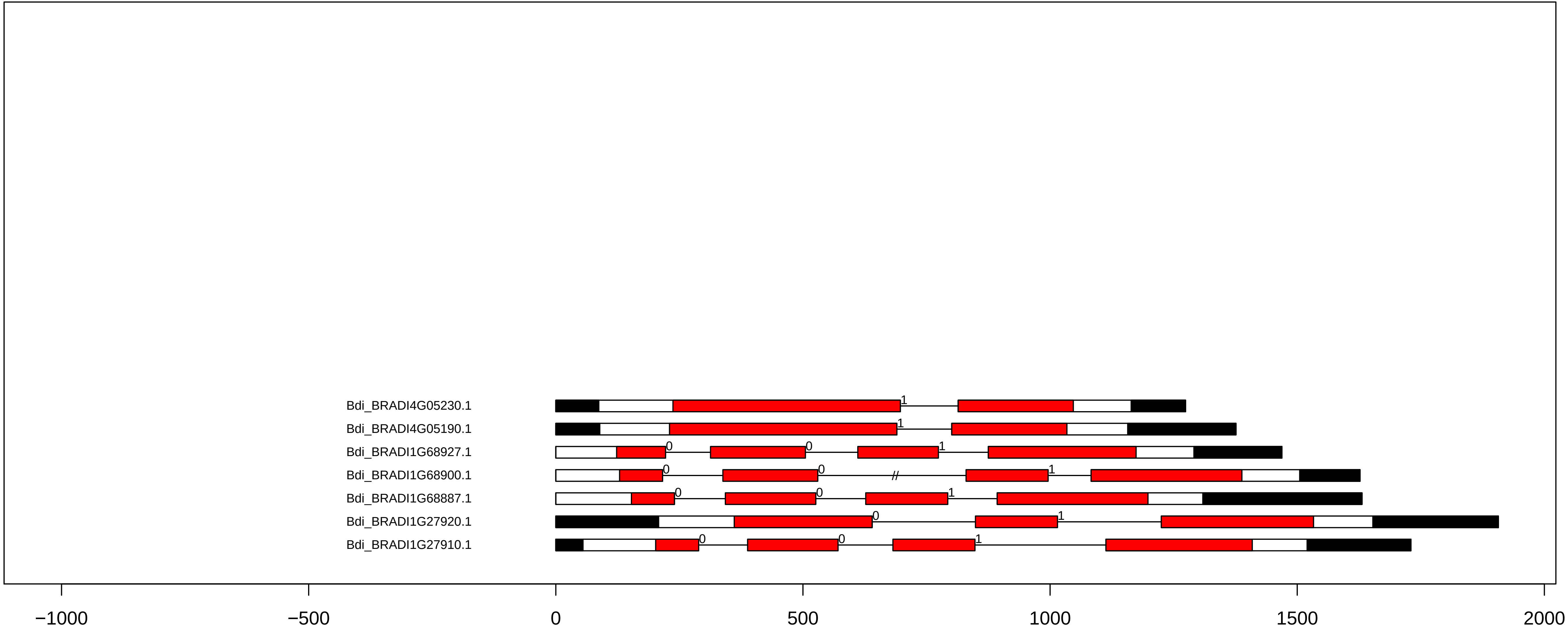

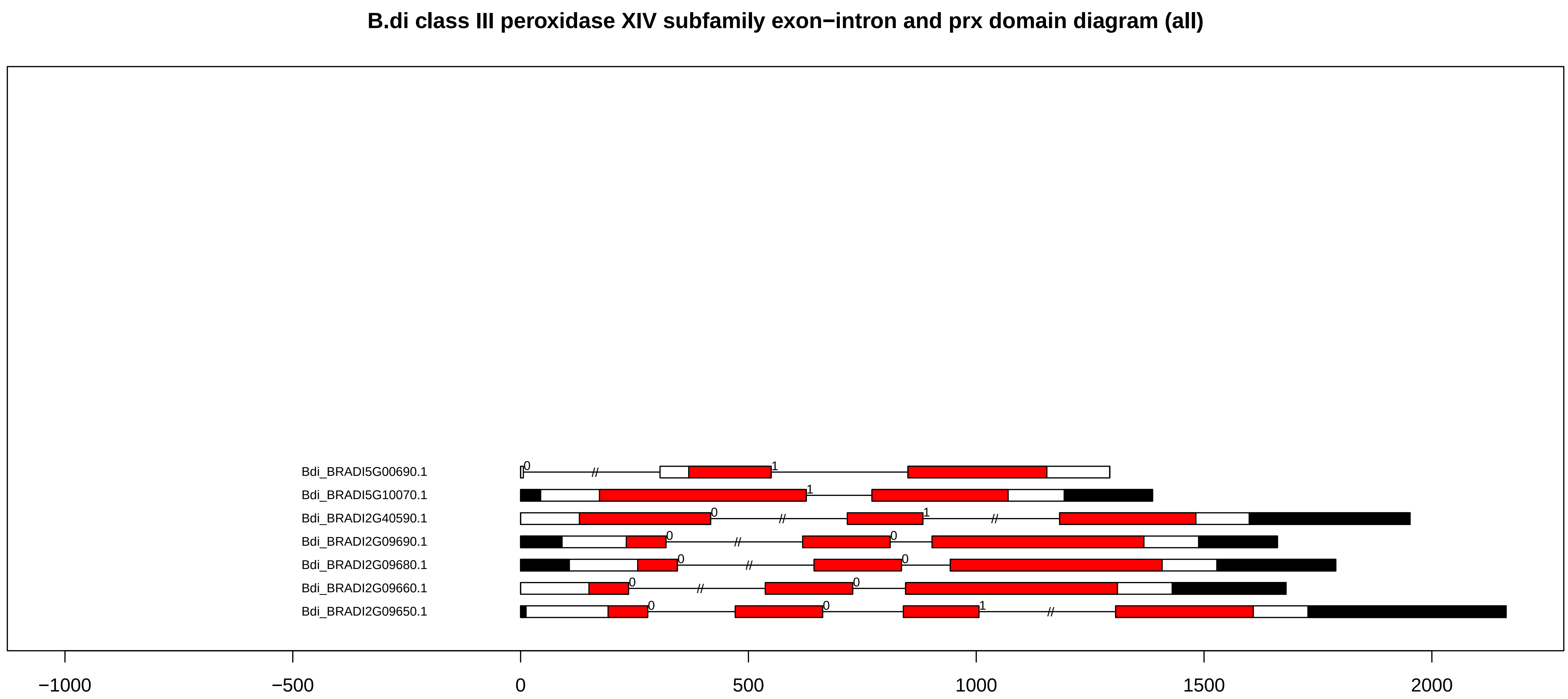
B.di class III peroxidase XIV subfamily exon−intron and prx domain diagram (all)
Bdi_BRADI5G00690.1
Bdi_BRADI5G10070.1
Bdi_BRADI2G40590.1
Bdi_BRADI2G09690.1
Bdi_BRADI2G09680.1
Bdi_BRADI2G09660.1
Bdi_BRADI2G09650.1
−1000
−500
0
500
1000
1500
2000

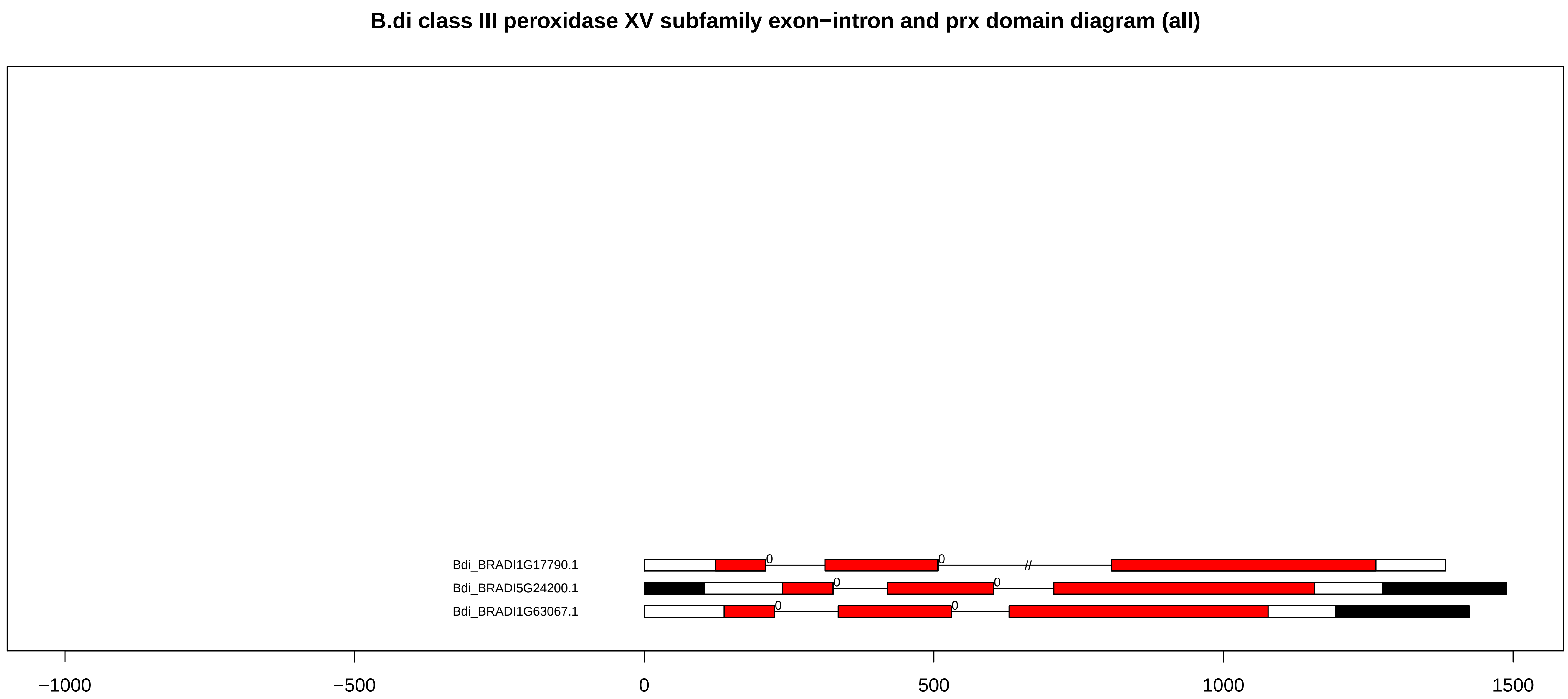
B.di class III peroxidase XV subfamily exon−intron and prx domain diagram (all)
Bdi_BRADI1G17790.1
Bdi_BRADI5G24200.1
Bdi_BRADI1G63067.1
0
0
0
0
0
0
//
−1000
−500
0
500
1000
1500

## B.di class III peroxidase XVI subfamily exon−intron and prx domain diagram (all)

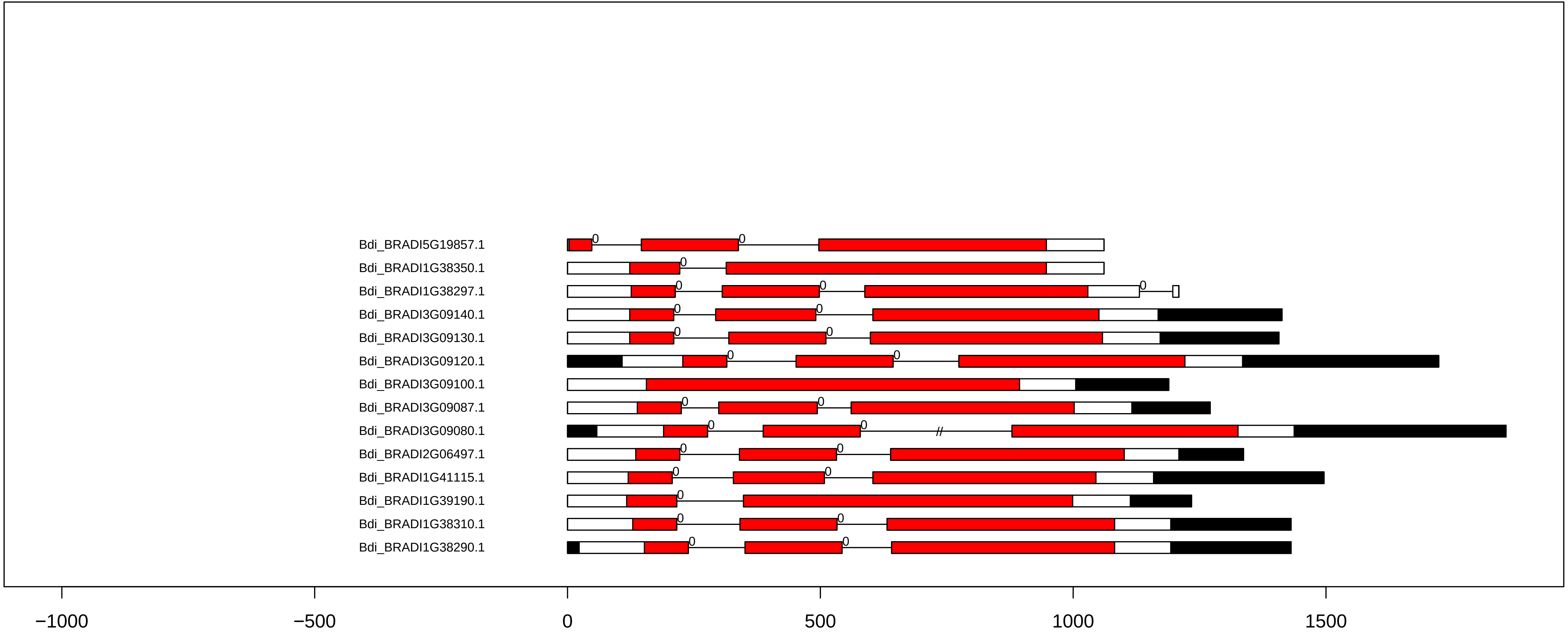

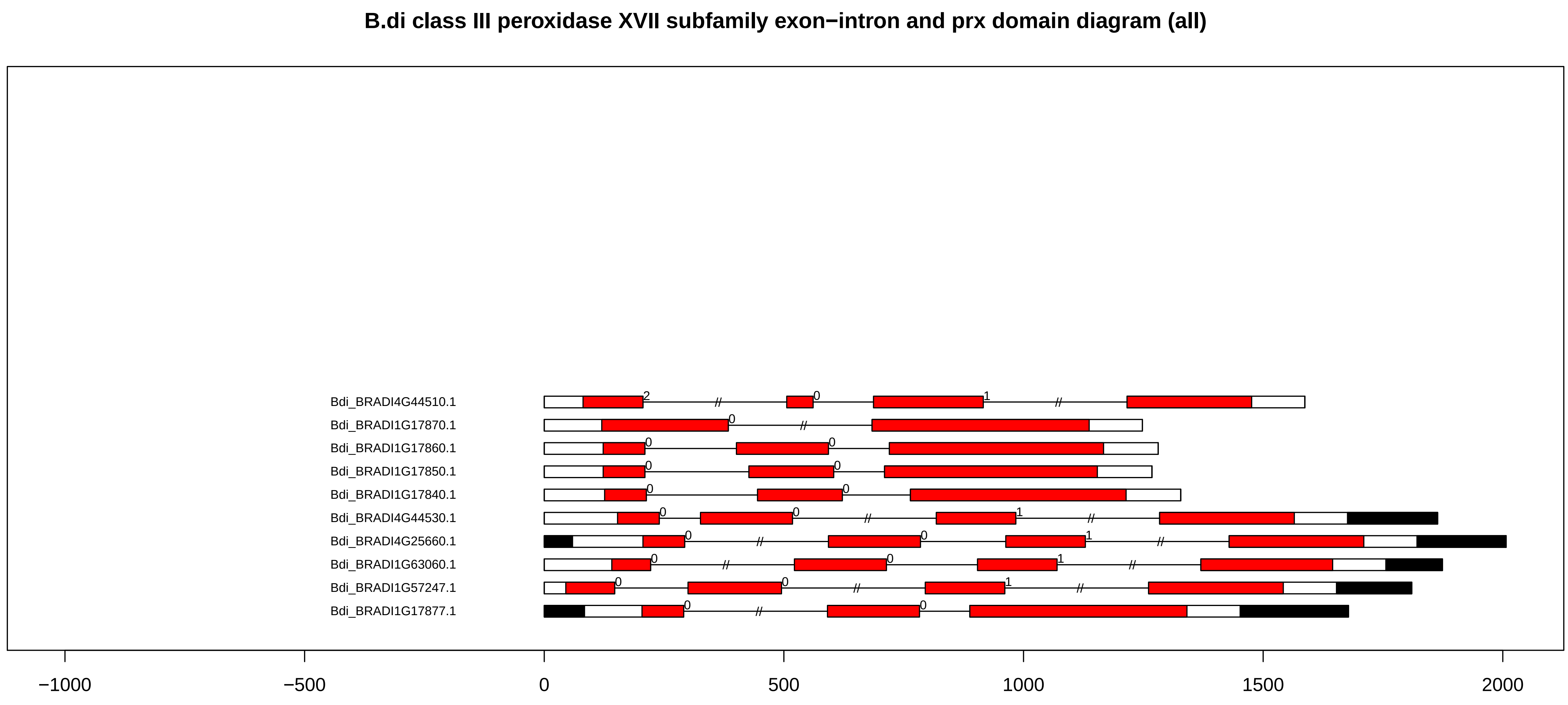

B.di class III peroxidase XVII subfamily exon−intron and prx domain diagram (all)
Bdi_BRADI4G44510.1
Bdi_BRADI1G17870.1
Bdi_BRADI1G17860.1
Bdi_BRADI1G17850.1
Bdi_BRADI1G17840.1
Bdi_BRADI4G44530.1
Bdi_BRADI4G25660.1
Bdi_BRADI1G63060.1
Bdi_BRADI1G57247.1
Bdi_BRADI1G17877.1
−1000
−500
0
500
1000
1500
2000

B.di class III peroxidase XVIII subfamily exon−intron and prx domain diagram (all)

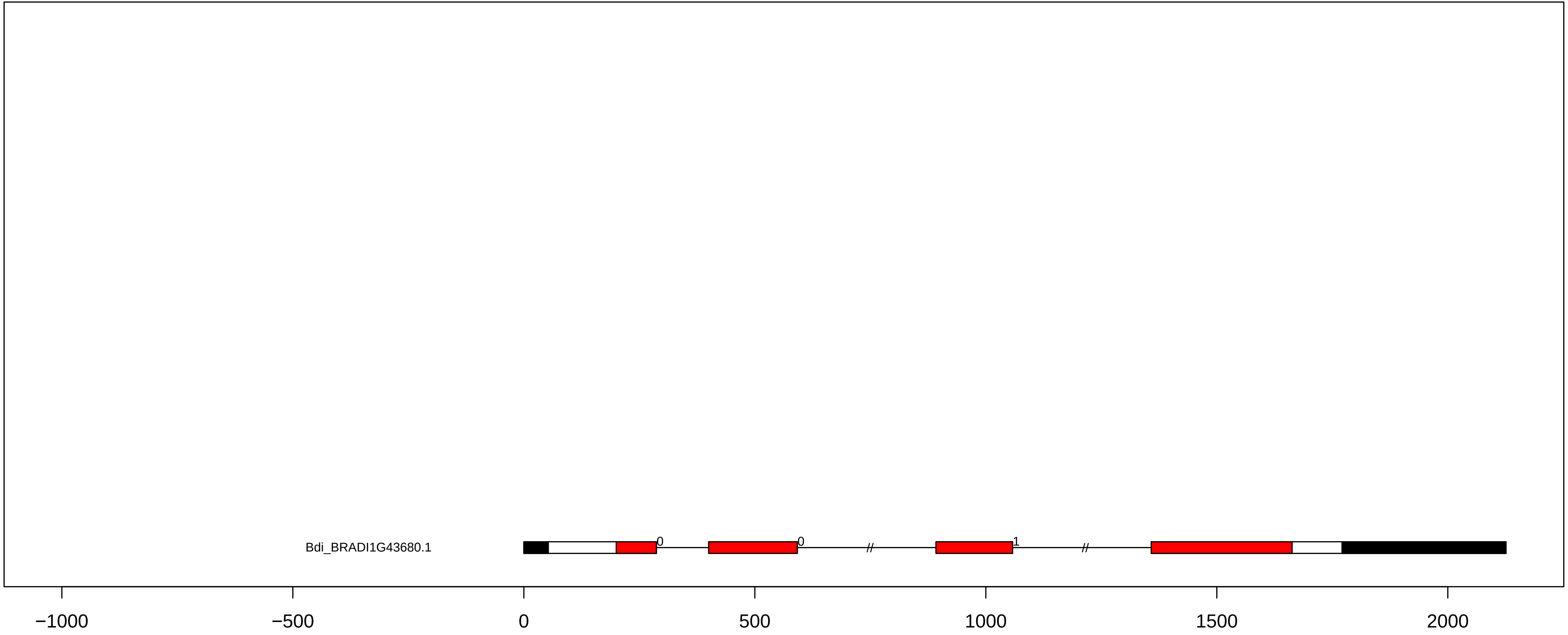

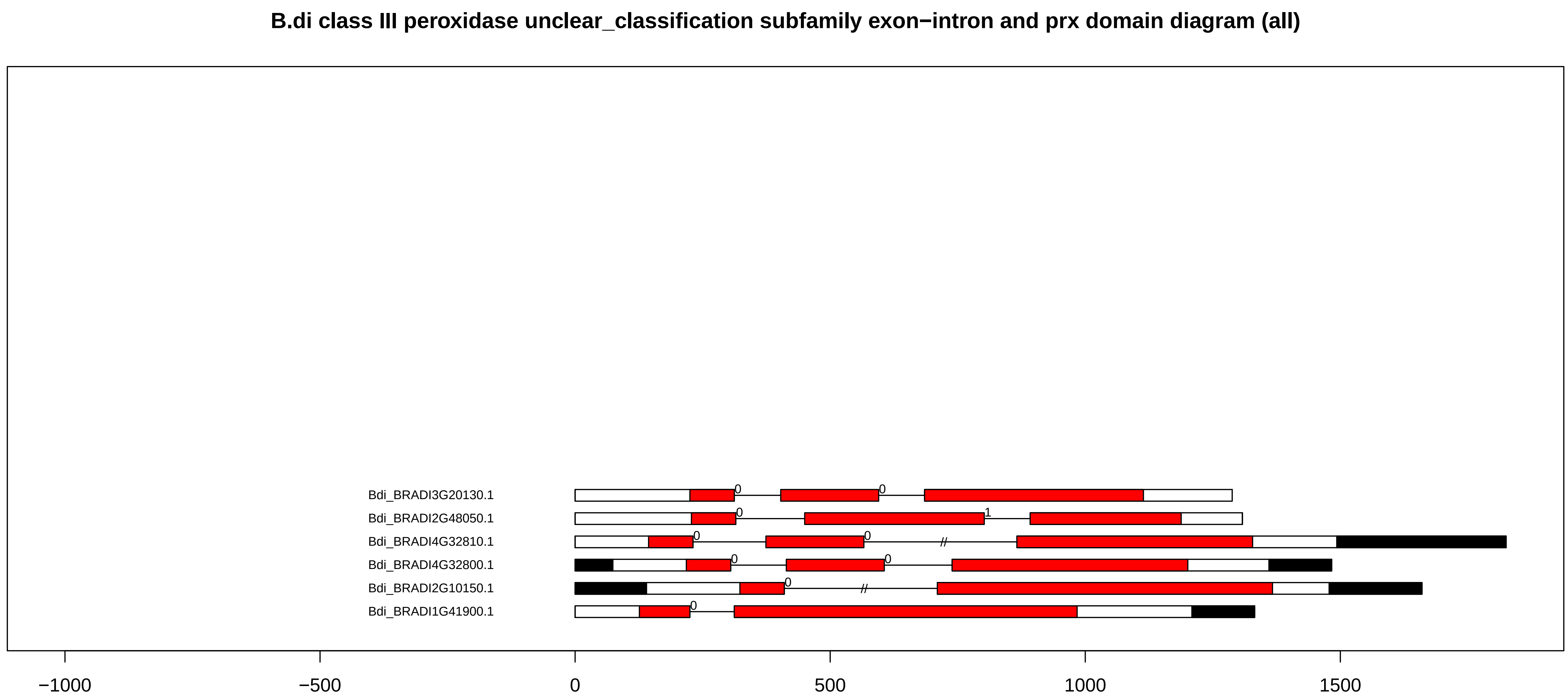
B.di class III peroxidase unclear_classification subfamily exon−intron and prx domain diagram (all)
Bdi_BRADI3G20130.1
Bdi_BRADI2G48050.1
Bdi_BRADI4G32810.1
Bdi_BRADI4G32800.1
Bdi_BRADI2G10150.1
Bdi_BRADI1G41900.1
−1000
−500
0
500
1000
1500

# O.sa class III peroxidase I subfamily exon−intron and prx domain diagram (part 1)

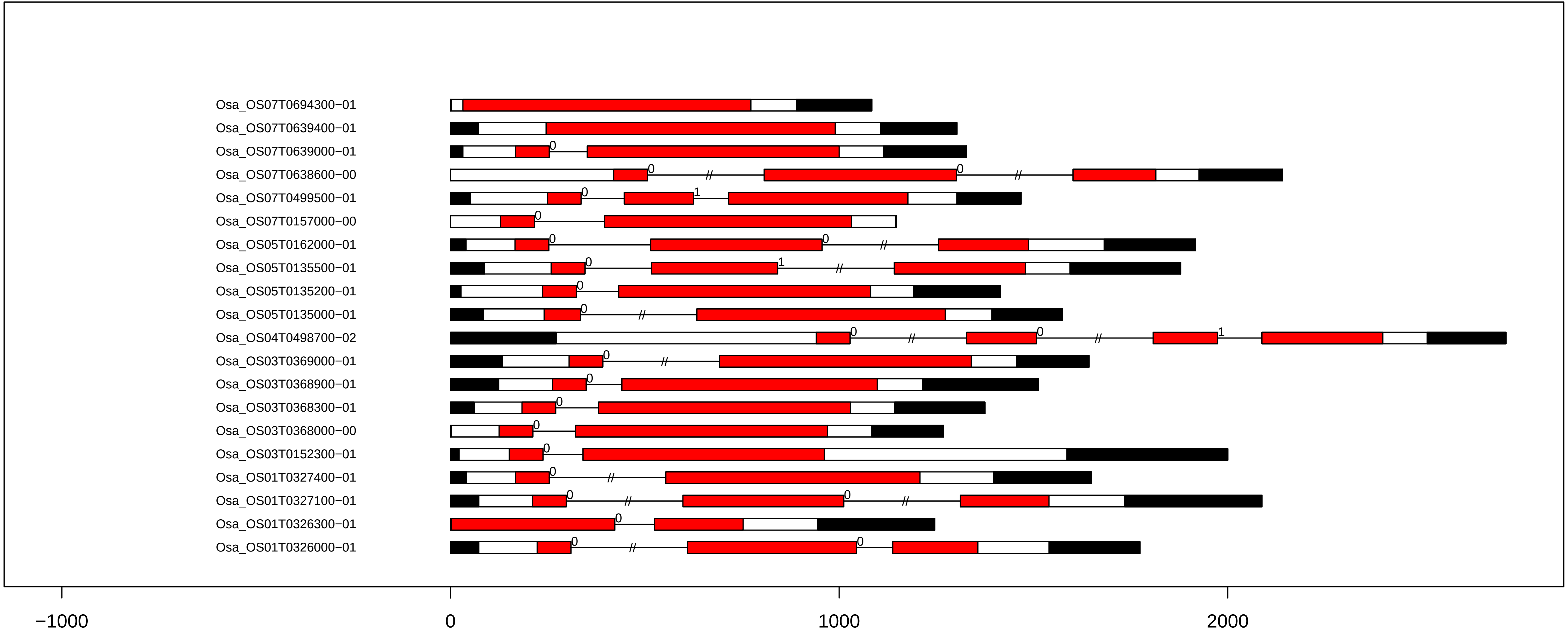

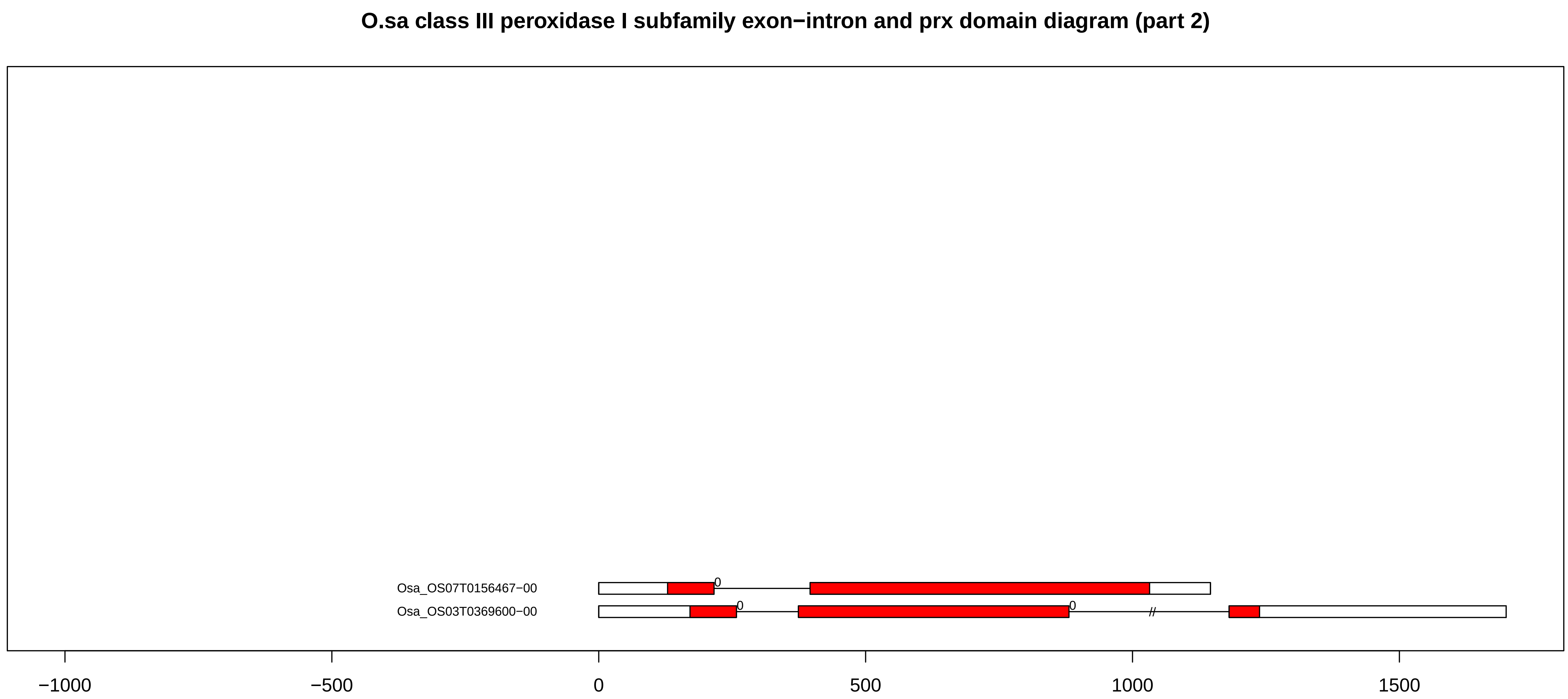
O.sa class III peroxidase I subfamily exon−intron and prx domain diagram (part 2)
Osa_OS07T0156467−00
Osa_OS03T0369600−00
0
0
0
−1000
−500
0
500
1000
1500

**O.sa class III peroxidase II subfamily exon−intron and prx domain diagram (all)**

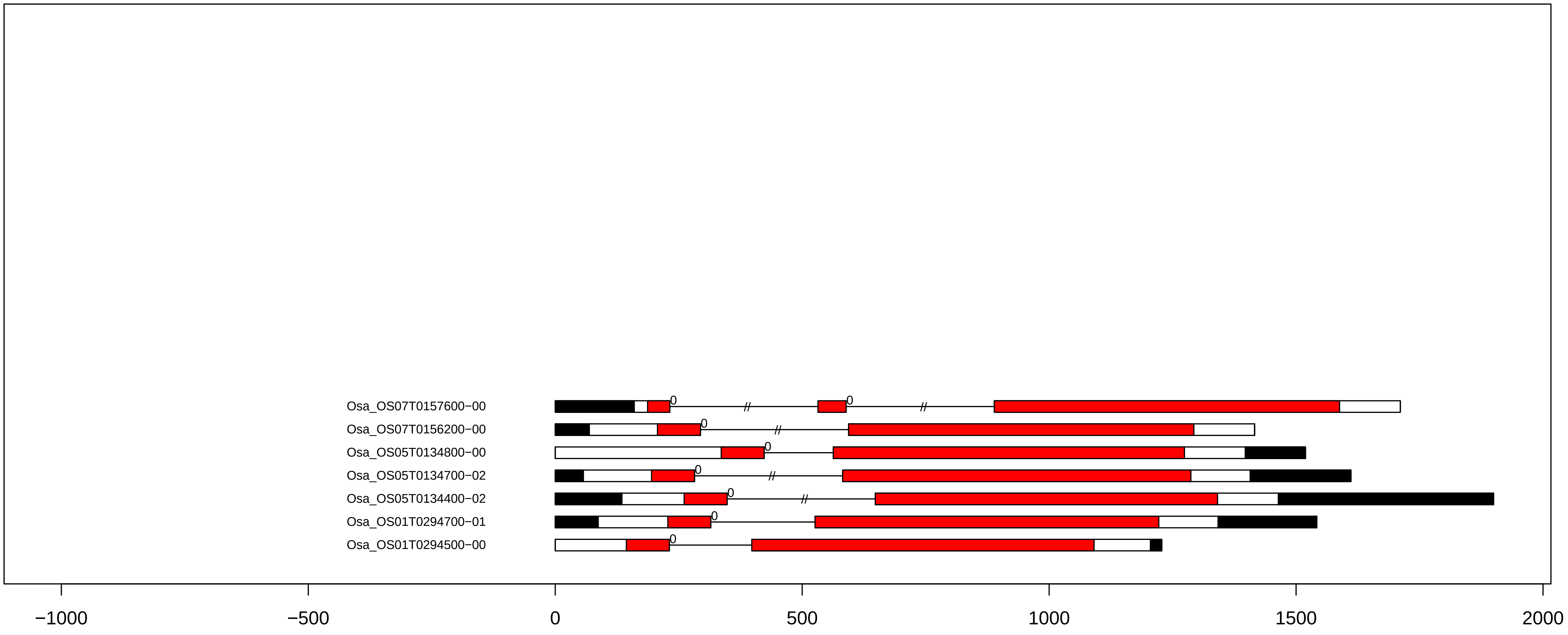

# O.sa class III peroxidase V subfamily exon−intron and prx domain diagram (all)

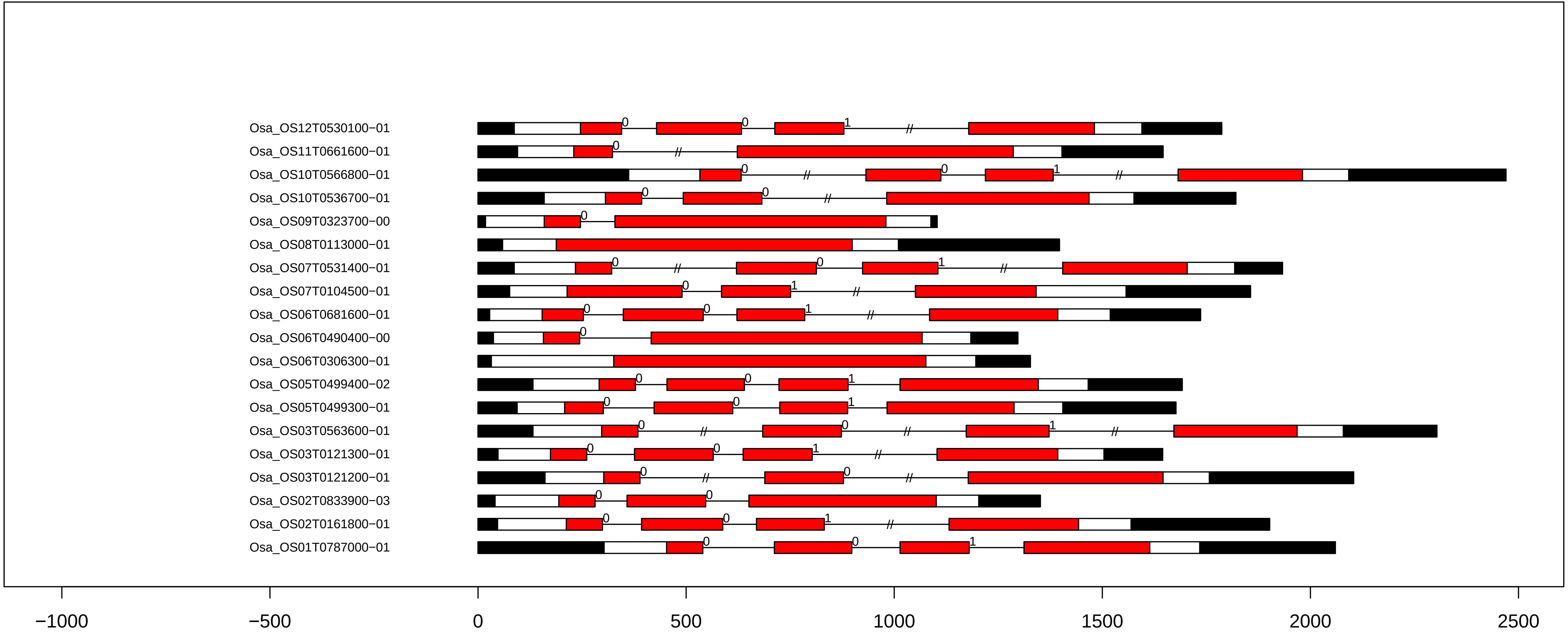

O.sa class III peroxidase VI subfamily exon−intron and prx domain diagram (all)

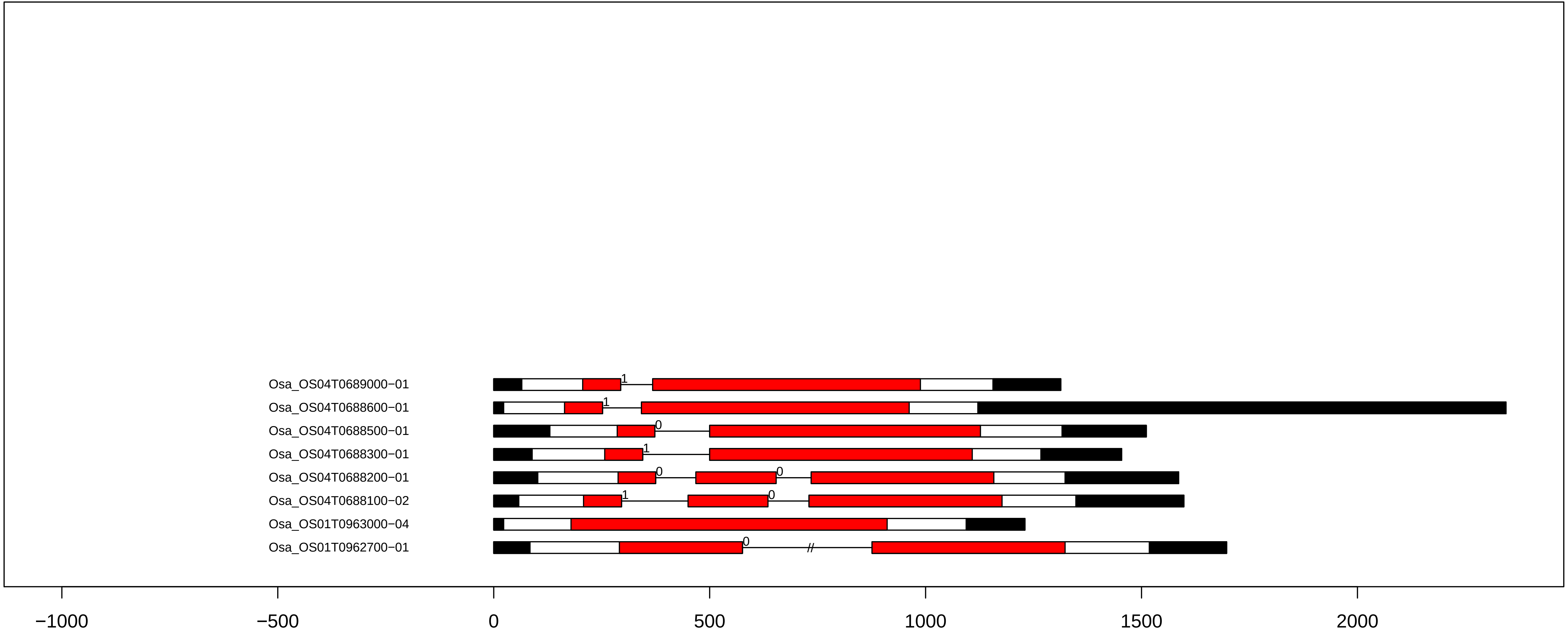

**O.sa class III peroxidase VII subfamily exon−intron and prx domain diagram (all)**

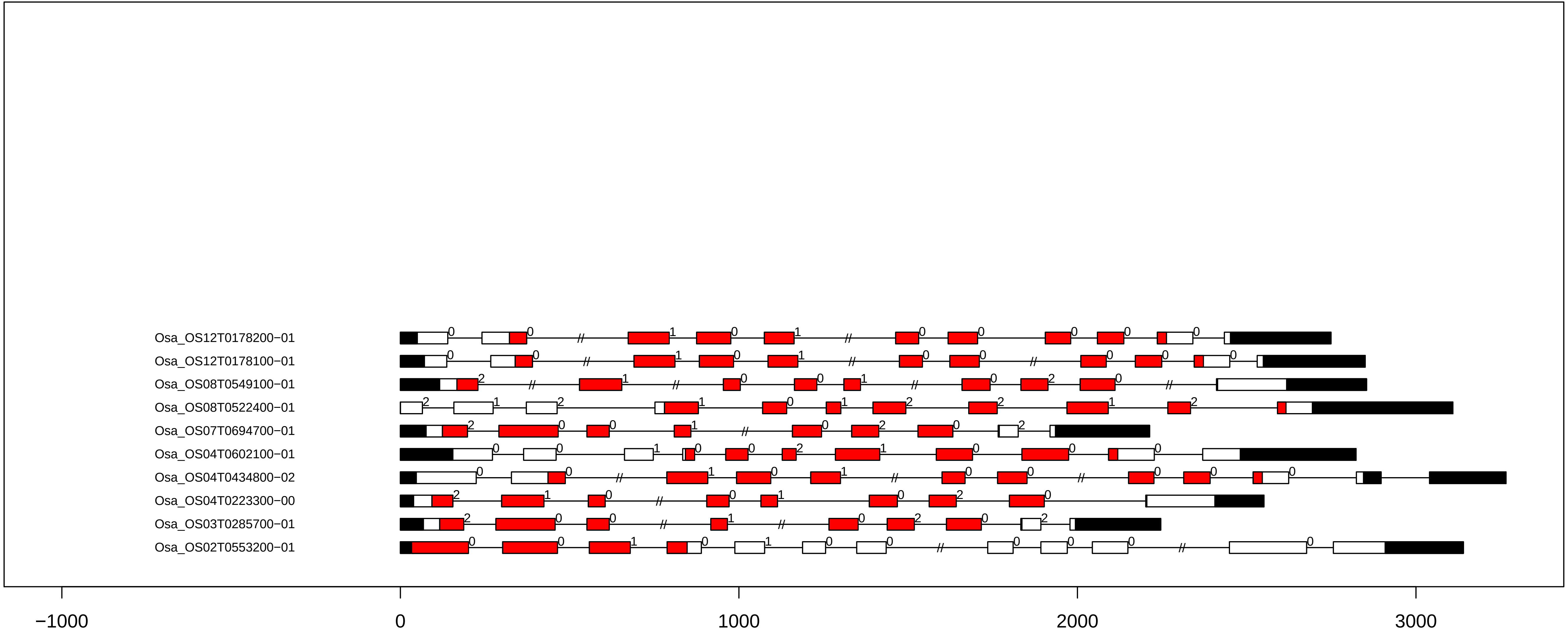

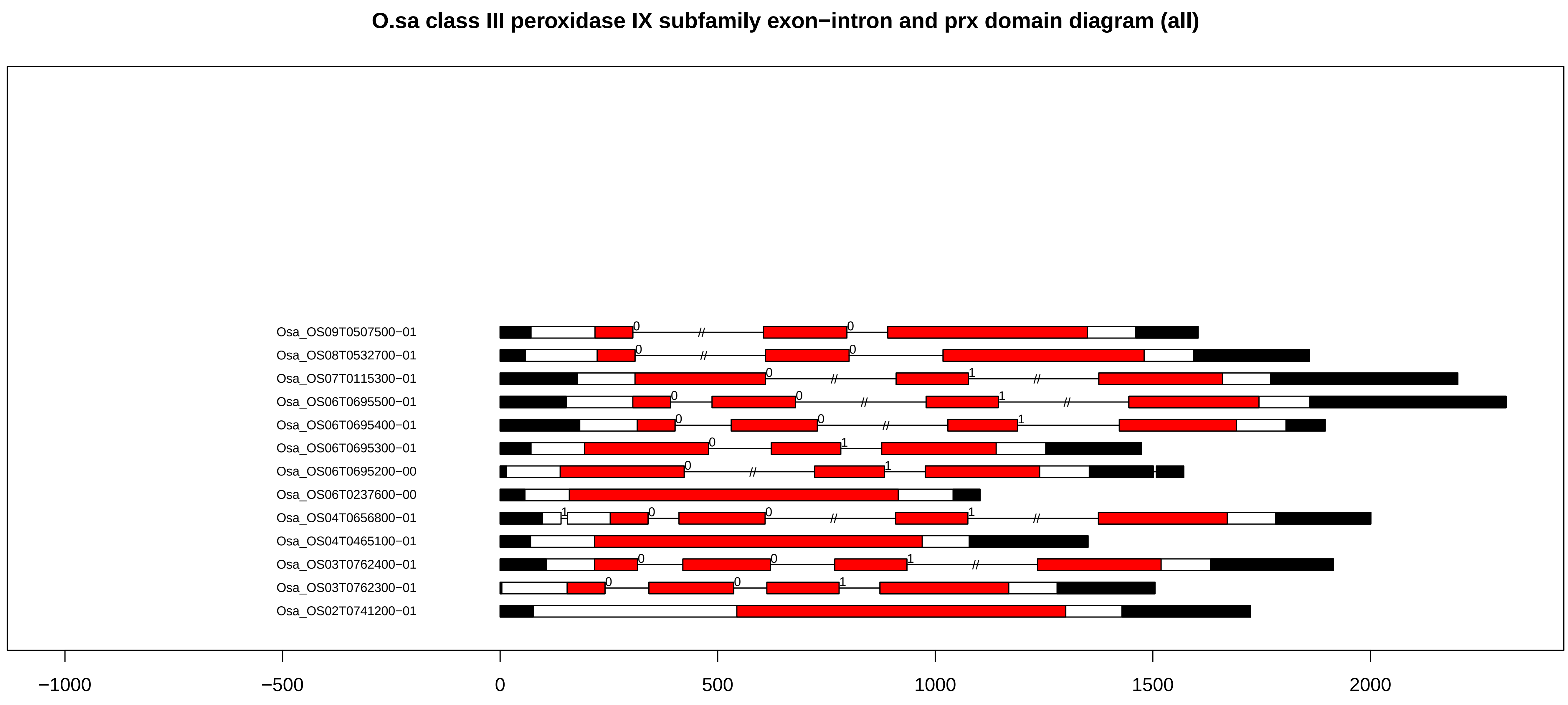

O.sa class III peroxidase IX subfamily exon−intron and prx domain diagram (all)
Osa_OS09T0507500−01
Osa_OS08T0532700−01
Osa_OS07T0115300−01
Osa_OS06T0695500−01
Osa_OS06T0695400−01
Osa_OS06T0695300−01
Osa_OS06T0695200−00
Osa_OS06T0237600−00
Osa_OS04T0656800−01
Osa_OS04T0465100−01
Osa_OS03T0762400−01
Osa_OS03T0762300−01
Osa_OS02T0741200−01
−1000
−500
0
500
1000
1500
2000

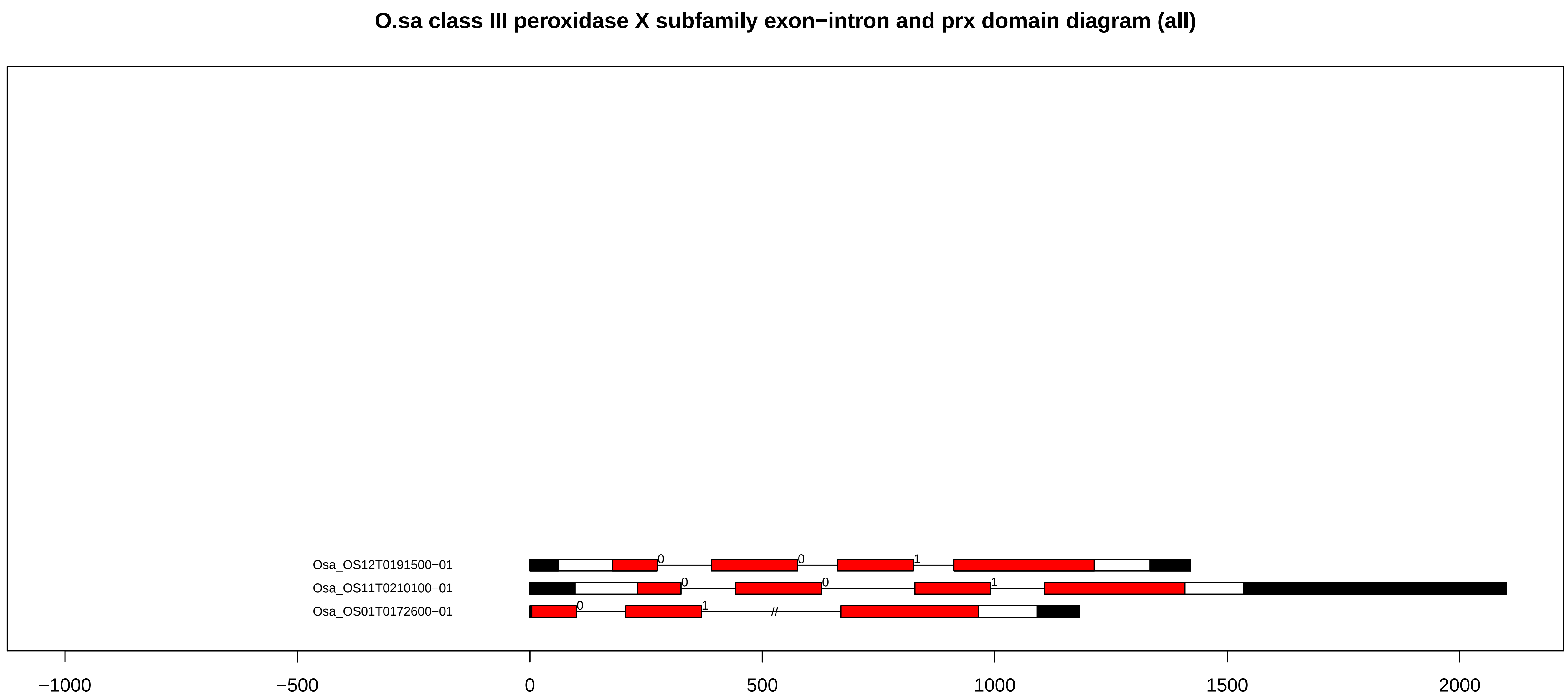

O.sa class III peroxidase X subfamily exon−intron and prx domain diagram (all)
Osa_OS12T0191500−01
Osa_OS11T0210100−01
Osa_OS01T0172600−01
0
0
1
0
0
1
0
1
−1000
−500
0
500
1000
1500
2000

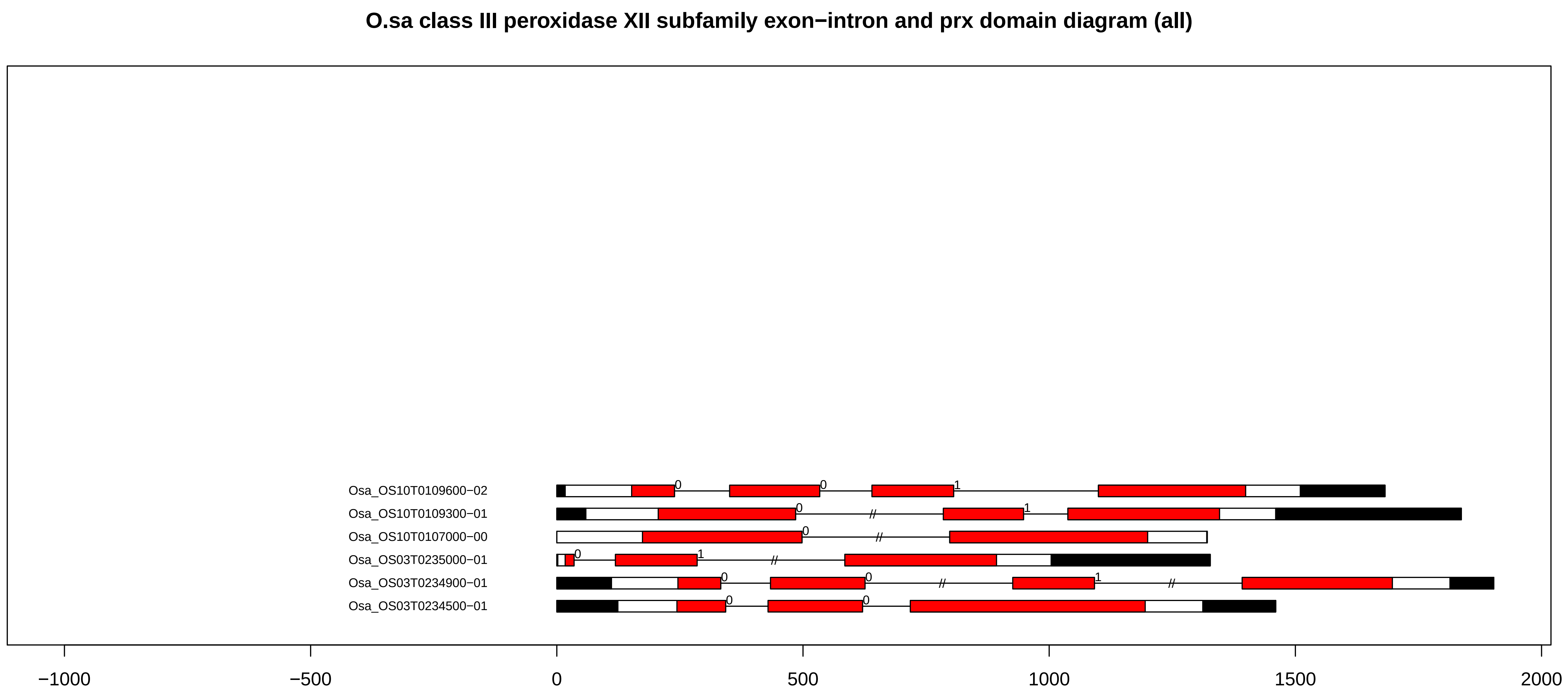

O.sa class III peroxidase XII subfamily exon−intron and prx domain diagram (all)
Osa_OS10T0109600−02
Osa_OS10T0109300−01
Osa_OS10T0107000−00
Osa_OS03T0235000−01
Osa_OS03T0234900−01
Osa_OS03T0234500−01
−1000
−500
0
500
1000
1500
2000

## O.sa class III peroxidase XIV subfamily exon−intron and prx domain diagram (all)

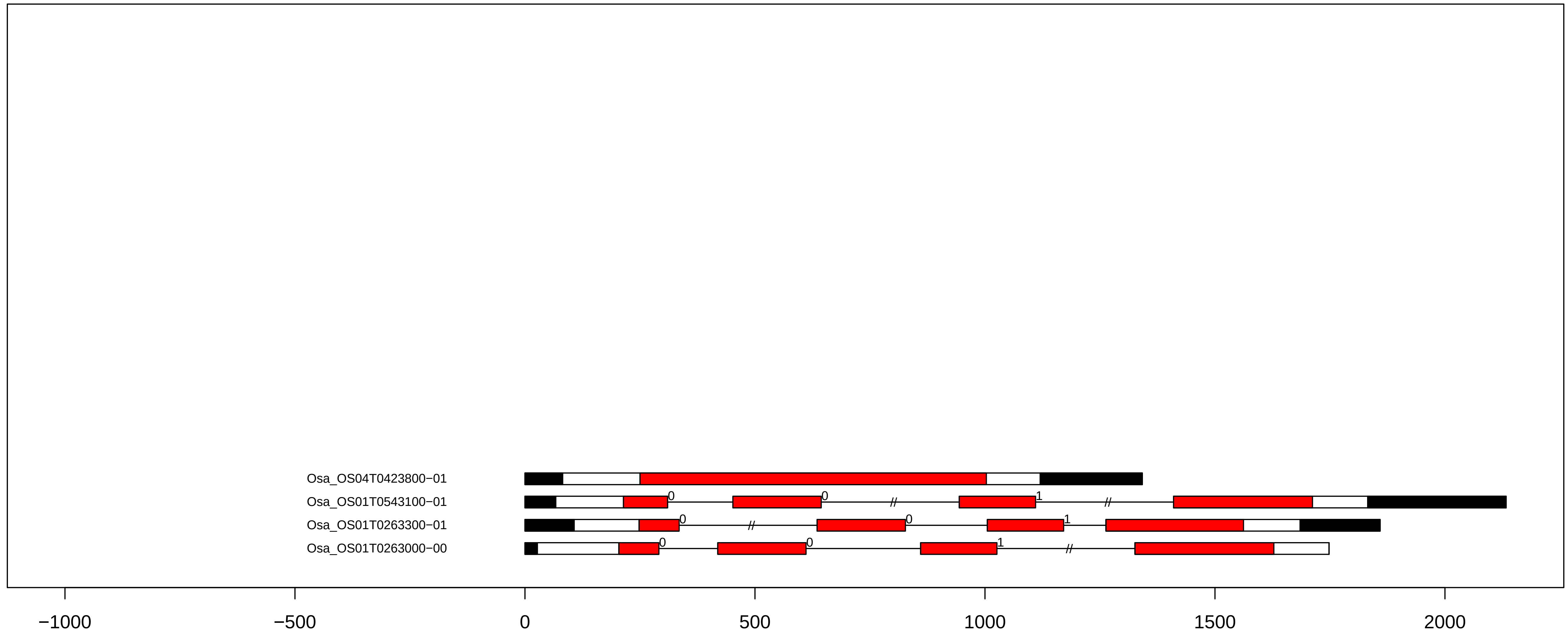

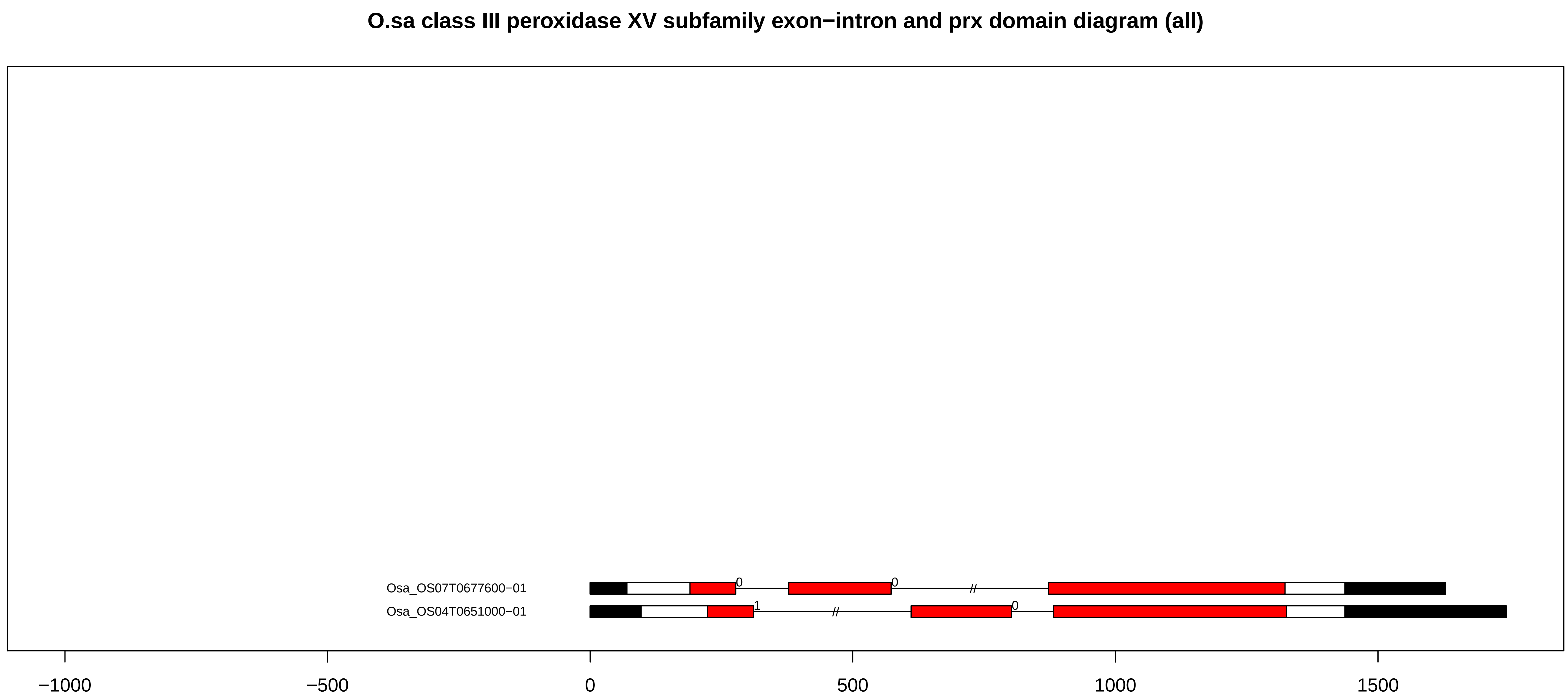
O.sa class III peroxidase XV subfamily exon−intron and prx domain diagram (all)
Osa_OS07T0677600−01
Osa_OS04T0651000−01
0
0
1
0
−1000
−500
0
500
1000
1500

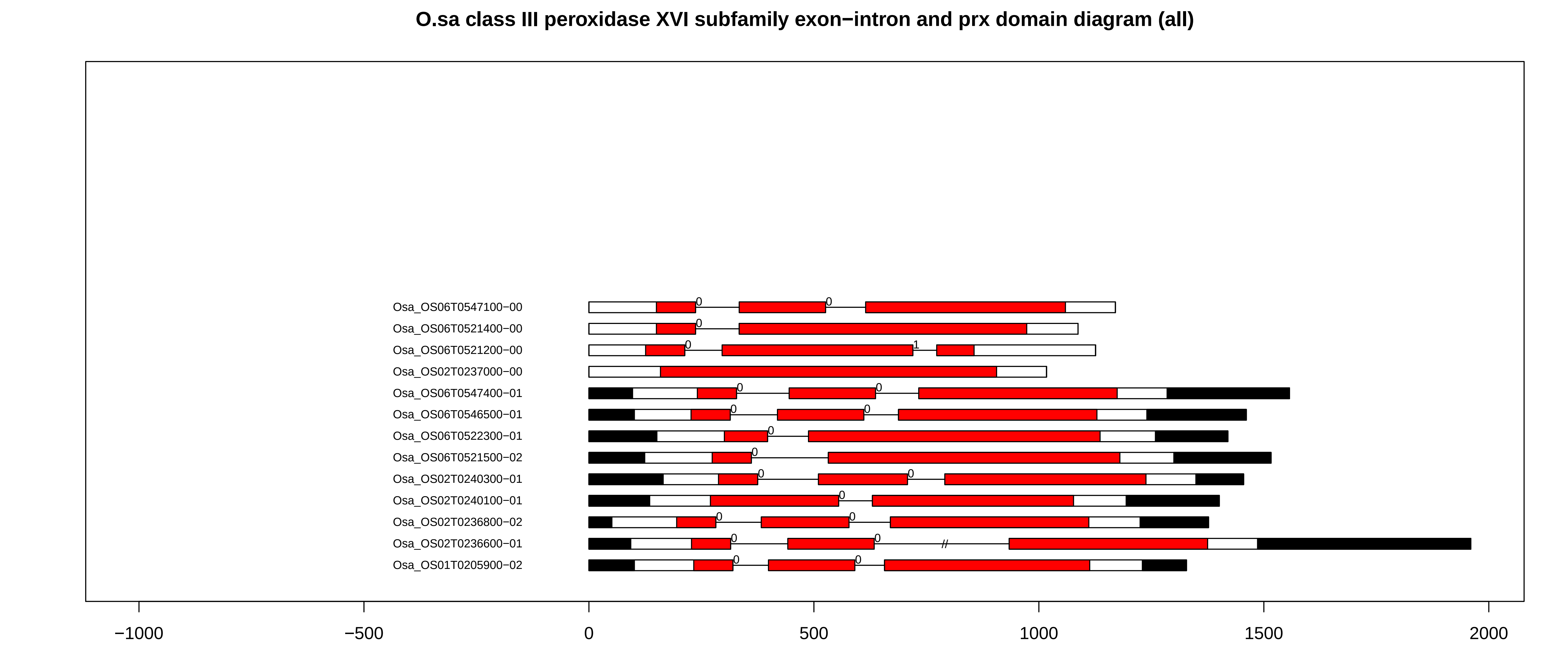
O.sa class III peroxidase XVI subfamily exon−intron and prx domain diagram (all)
Osa_OS06T0547100−00
Osa_OS06T0521400−00
Osa_OS06T0521200−00
Osa_OS02T0237000−00
Osa_OS06T0547400−01
Osa_OS06T0546500−01
Osa_OS06T0522300−01
Osa_OS06T0521500−02
Osa_OS02T0240300−01
Osa_OS02T0240100−01
Osa_OS02T0236800−02
Osa_OS02T0236600−01
Osa_OS01T0205900−02
−1000
−500
0
500
1000
1500
2000

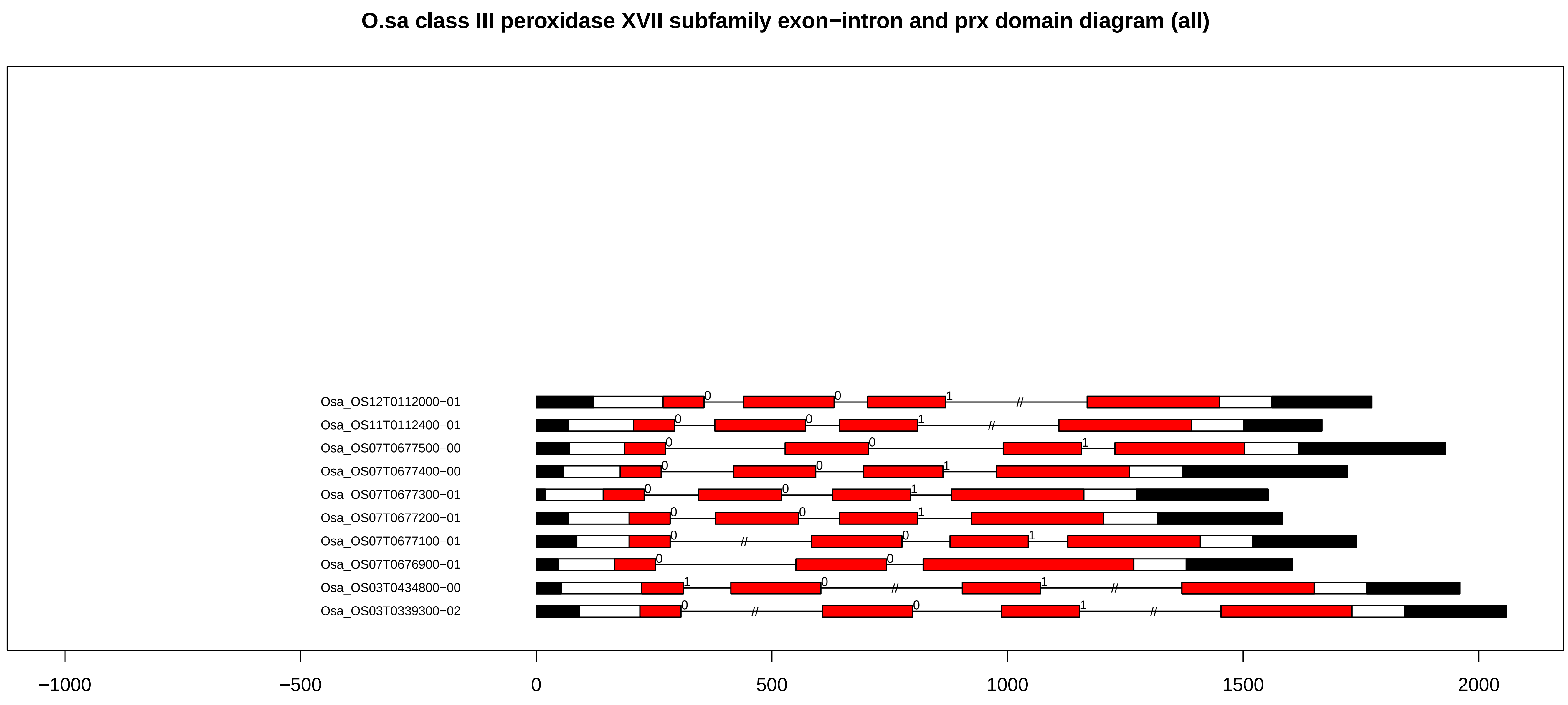

O.sa class III peroxidase XVII subfamily exon−intron and prx domain diagram (all)
Osa_OS12T0112000−01
Osa_OS11T0112400−01
Osa_OS07T0677500−00
Osa_OS07T0677400−00
Osa_OS07T0677300−01
Osa_OS07T0677200−01
Osa_OS07T0677100−01
Osa_OS07T0676900−01
Osa_OS03T0434800−00
Osa_OS03T0339300−02
−1000
−500
0
500
1000
1500
2000

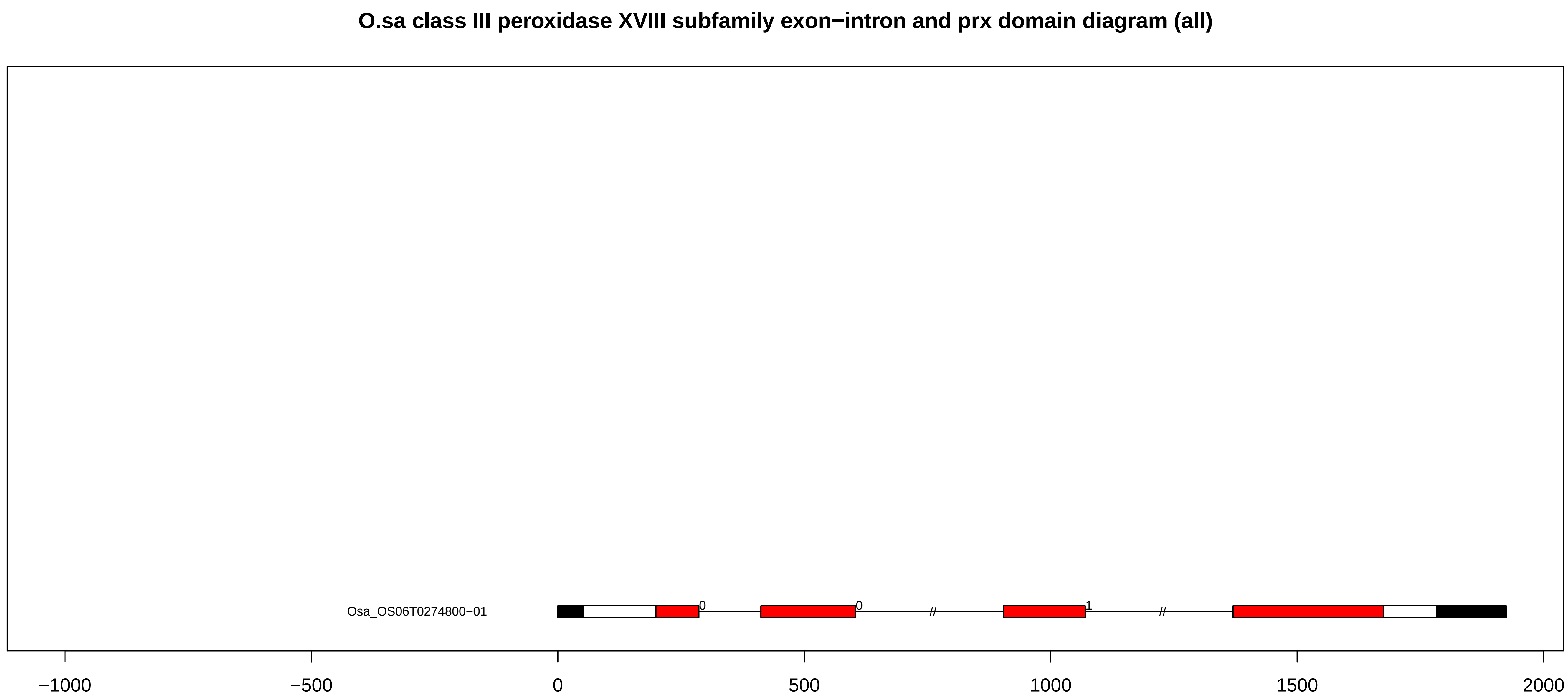
O.sa class III peroxidase XVIII subfamily exon−intron and prx domain diagram (all)
Osa_OS06T0274800−01
0
0
1
−1000
−500
0
500
1000
1500
2000

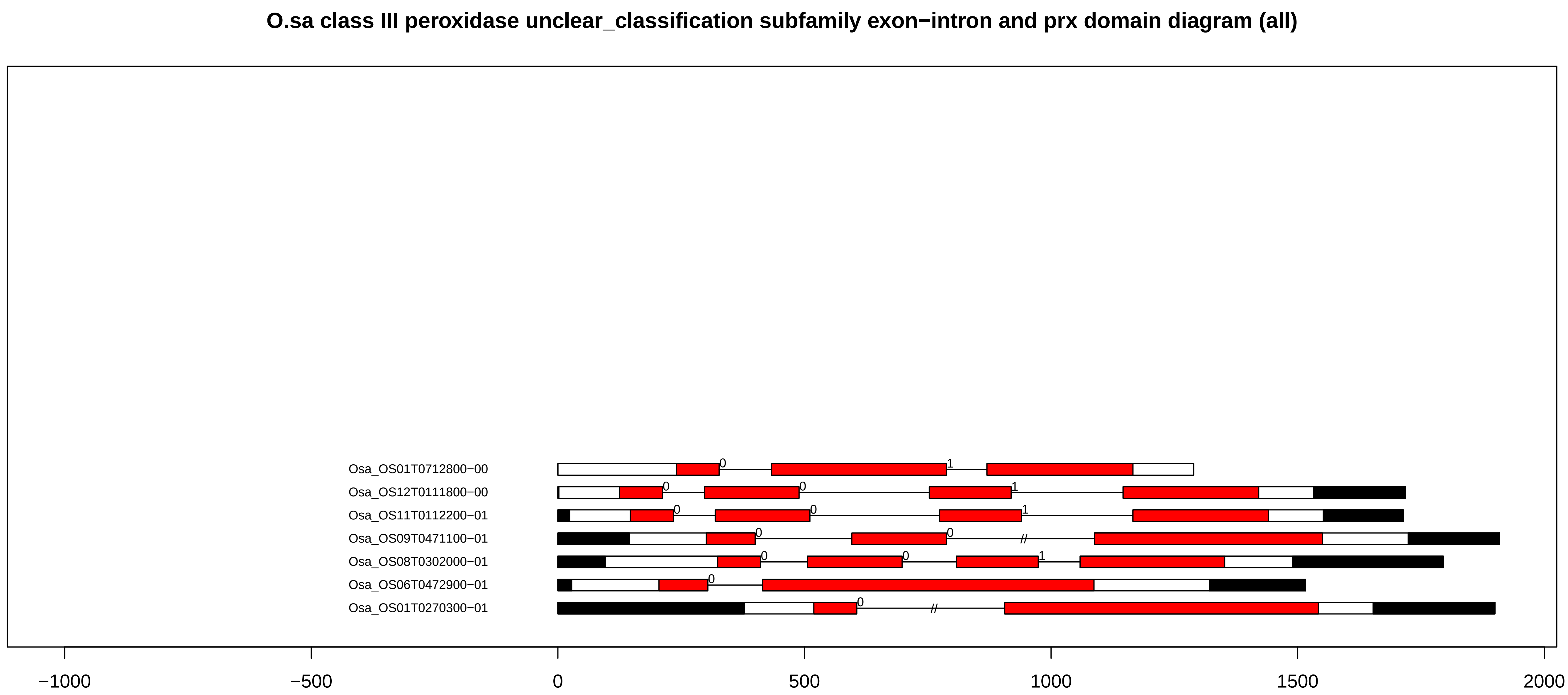
O.sa class III peroxidase unclear_classification subfamily exon−intron and prx domain diagram (all)
Osa_OS01T0712800−00
Osa_OS12T0111800−00
Osa_OS11T0112200−01
Osa_OS09T0471100−01
Osa_OS08T0302000−01
Osa_OS06T0472900−01
Osa_OS01T0270300−01
−1000
−500
0
500
1000
1500
2000

# Z.ma class III peroxidase I subfamily exon−intron and prx domain diagram (part 1)

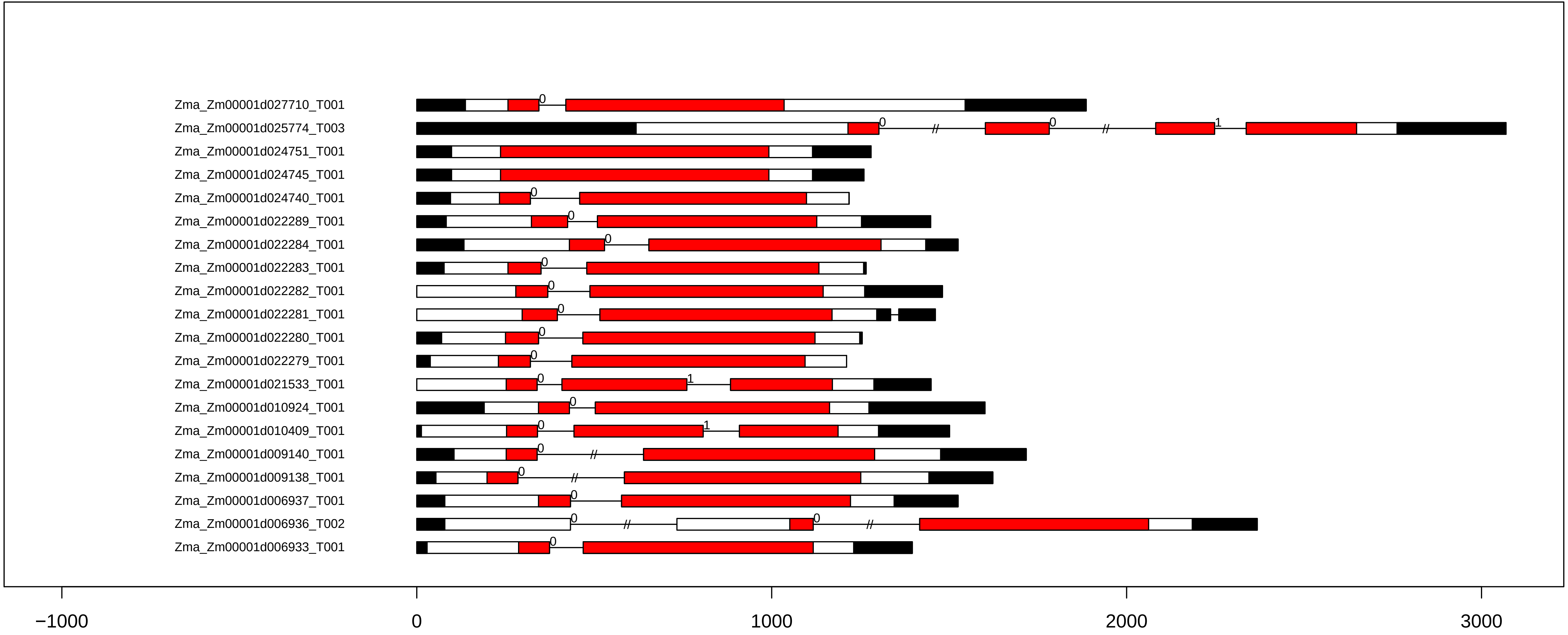

# Z.ma class III peroxidase I subfamily exon−intron and prx domain diagram (part 2)

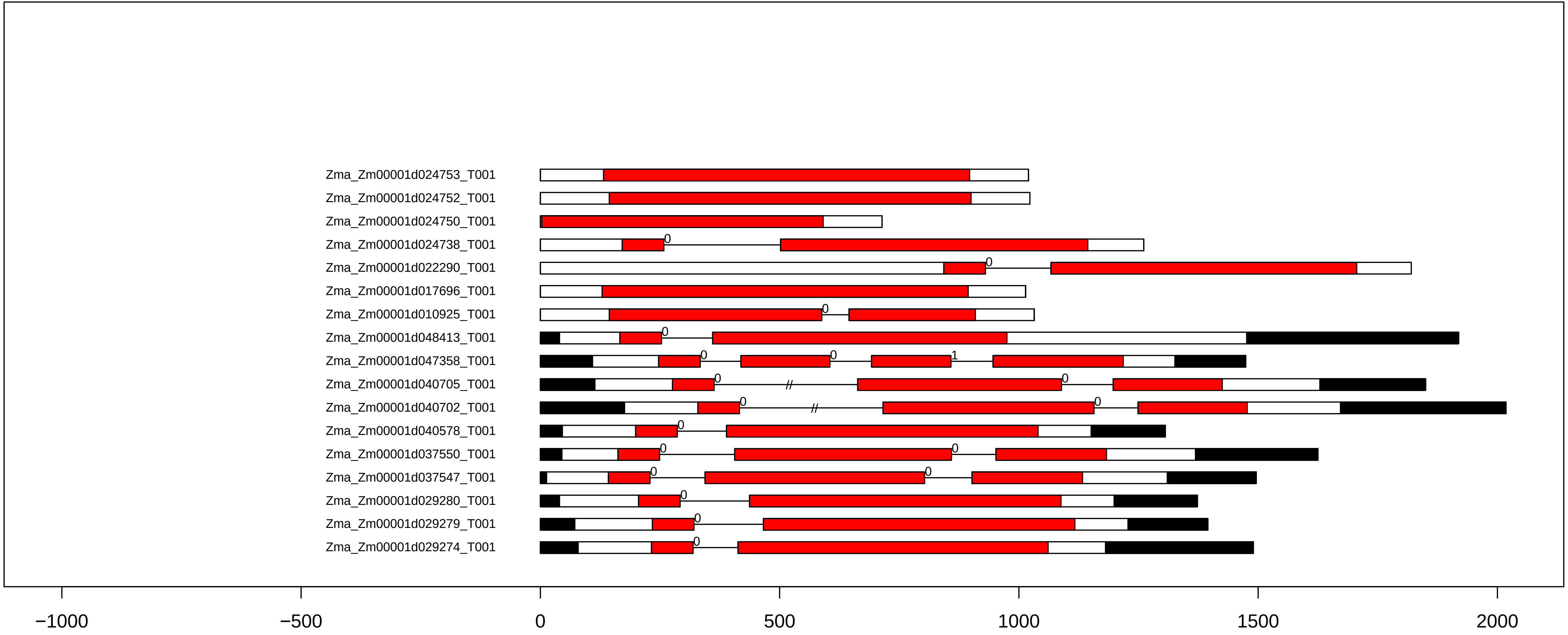

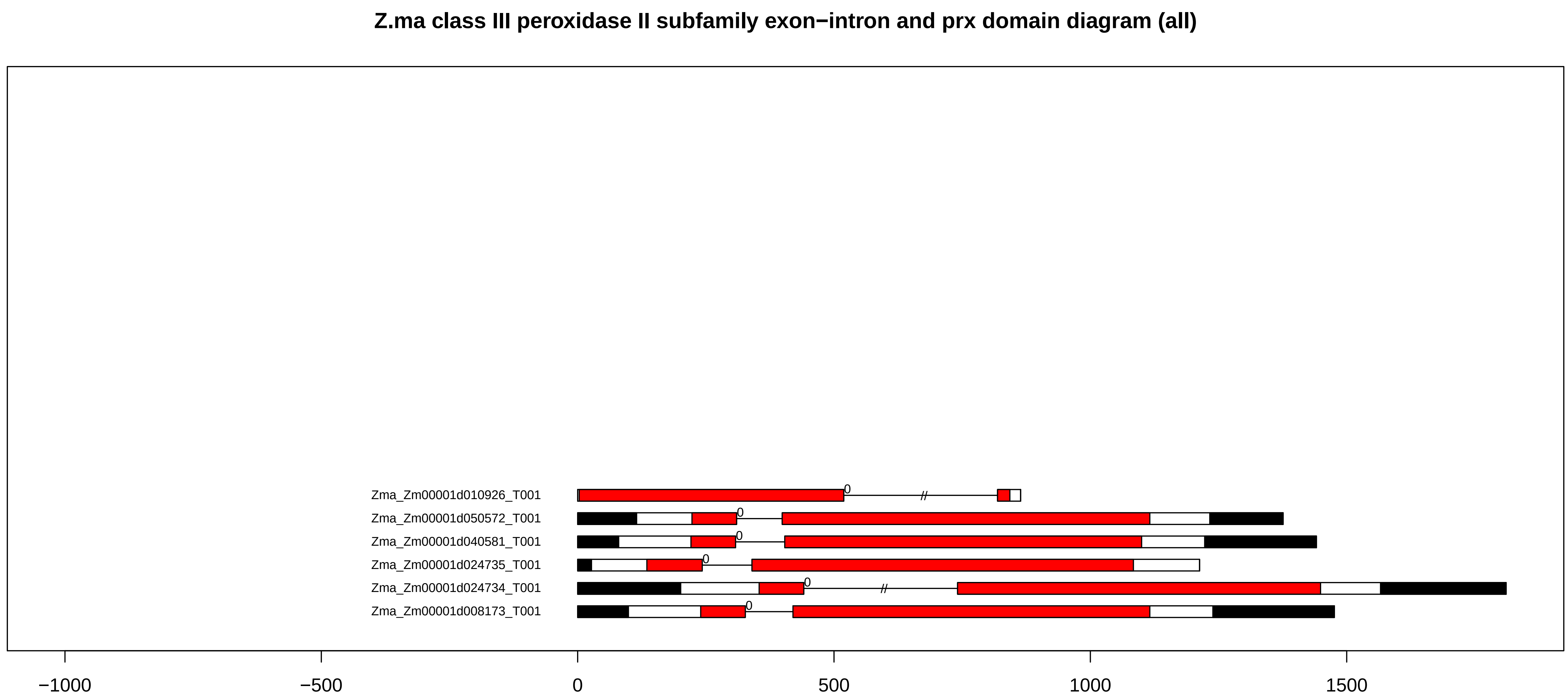
Z.ma class III peroxidase II subfamily exon−intron and prx domain diagram (all)
Zma_Zm00001d010926_T001
Zma_Zm00001d050572_T001
Zma_Zm00001d040581_T001
Zma_Zm00001d024735_T001
Zma_Zm00001d024734_T001
Zma_Zm00001d008173_T001
0
0
0
0
0
0
−1000
−500
0
500
1000
1500

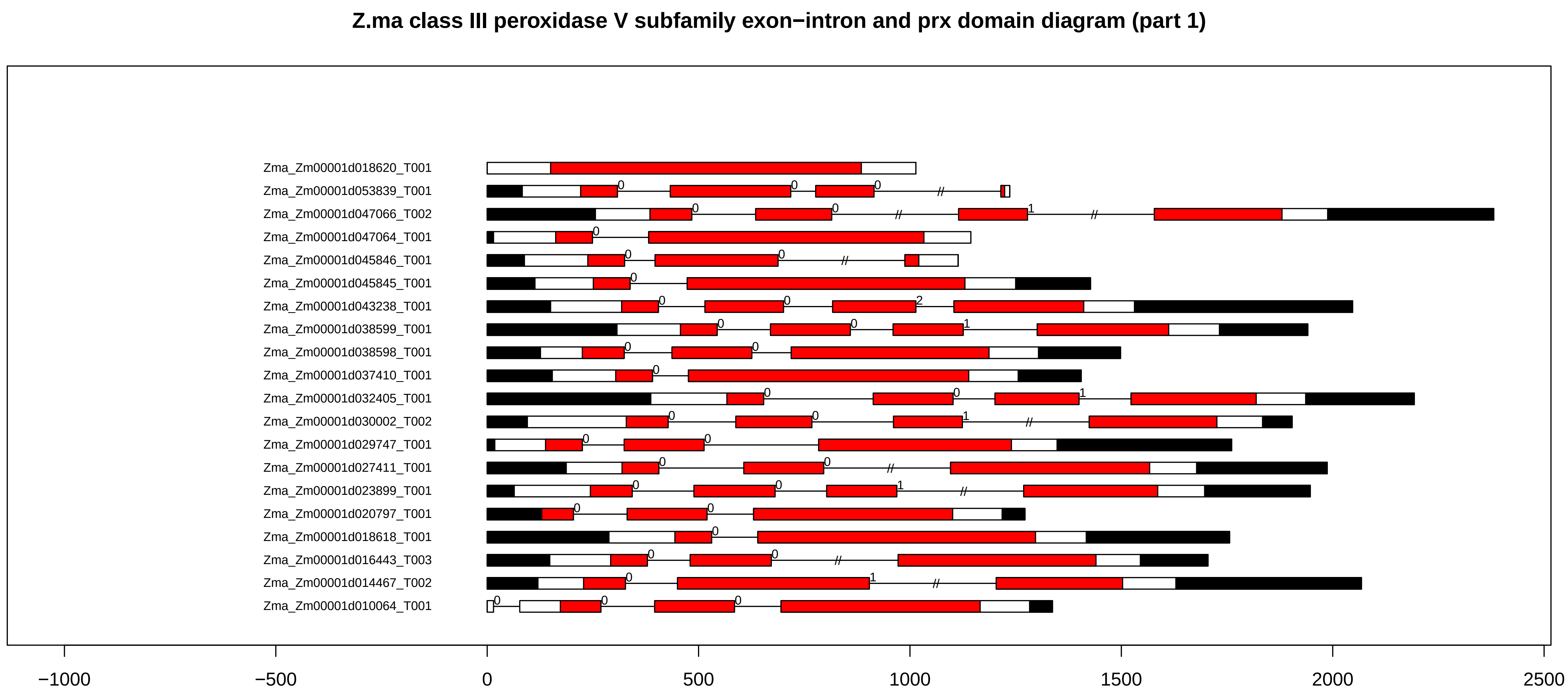
Z.ma class III peroxidase V subfamily exon−intron and prx domain diagram (part 1)
Zma_Zm00001d018620_T001
Zma_Zm00001d053839_T001
Zma_Zm00001d047066_T002
Zma_Zm00001d047064_T001
Zma_Zm00001d045846_T001
Zma_Zm00001d045845_T001
Zma_Zm00001d043238_T001
Zma_Zm00001d038599_T001
Zma_Zm00001d038598_T001
Zma_Zm00001d037410_T001
Zma_Zm00001d032405_T001
Zma_Zm00001d030002_T002
Zma_Zm00001d029747_T001
Zma_Zm00001d027411_T001
Zma_Zm00001d023899_T001
Zma_Zm00001d020797_T001
Zma_Zm00001d018618_T001
Zma_Zm00001d016443_T003
Zma_Zm00001d014467_T002
Zma_Zm00001d010064_T001
0
1
2
−1000
−500
0
500
1000
1500
2000
2500

Z.ma class III peroxidase V subfamily exon−intron and prx domain diagram (part 2)

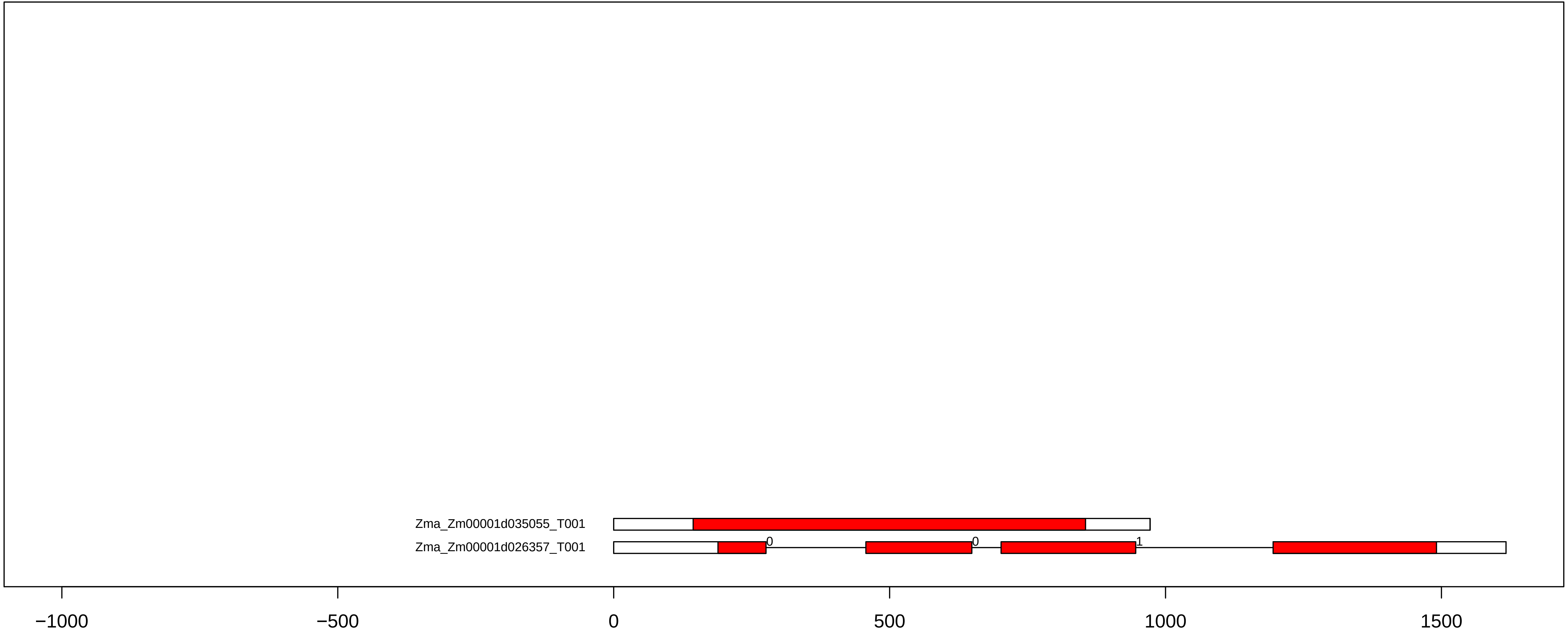

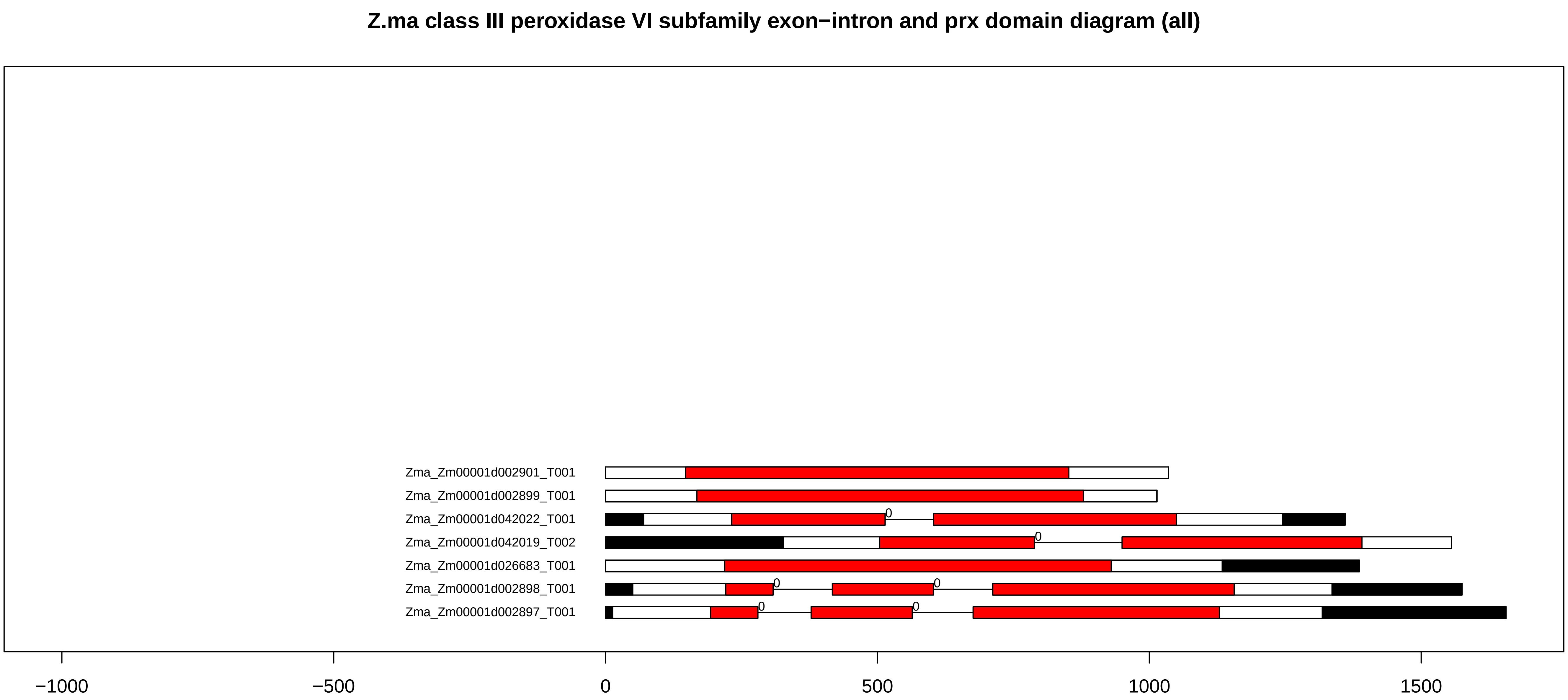
Z.ma class III peroxidase VI subfamily exon−intron and prx domain diagram (all)
Zma_Zm00001d002901_T001
Zma_Zm00001d002899_T001
Zma_Zm00001d042022_T001
Zma_Zm00001d042019_T002
Zma_Zm00001d026683_T001
Zma_Zm00001d002898_T001
Zma_Zm00001d002897_T001
0
0
0
0
0
0
−1000
−500
0
500
1000
1500

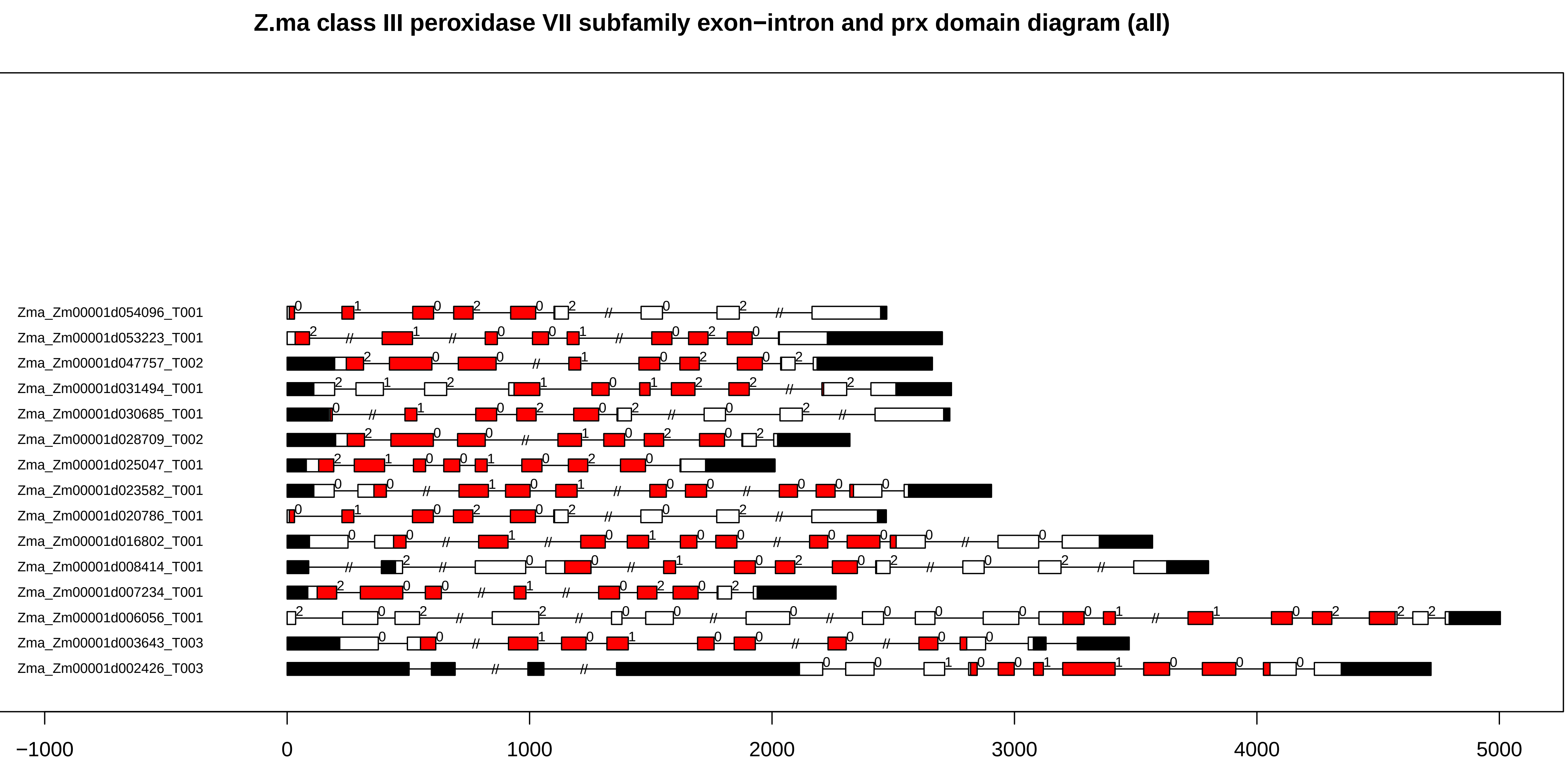

Z.ma class III peroxidase VII subfamily exon−intron and prx domain diagram (all)
Zma_Zm00001d054096_T001
Zma_Zm00001d053223_T001
Zma_Zm00001d047757_T002
Zma_Zm00001d031494_T001
Zma_Zm00001d030685_T001
Zma_Zm00001d028709_T002
Zma_Zm00001d025047_T001
Zma_Zm00001d023582_T001
Zma_Zm00001d020786_T001
Zma_Zm00001d016802_T001
Zma_Zm00001d008414_T001
Zma_Zm00001d007234_T001
Zma_Zm00001d006056_T001
Zma_Zm00001d003643_T003
Zma_Zm00001d002426_T003
−1000
0
1000
2000
3000
4000
5000

## Z.ma class III peroxidase IX subfamily exon−intron and prx domain diagram (all)

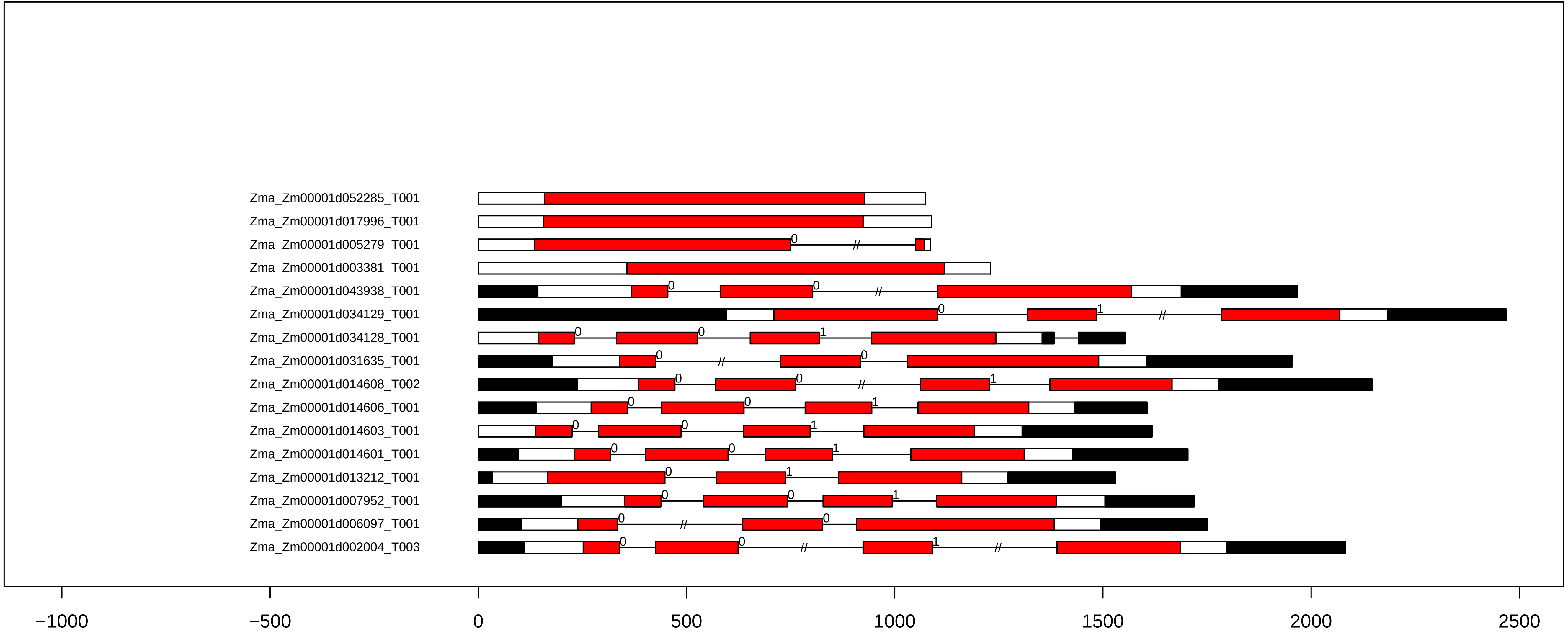

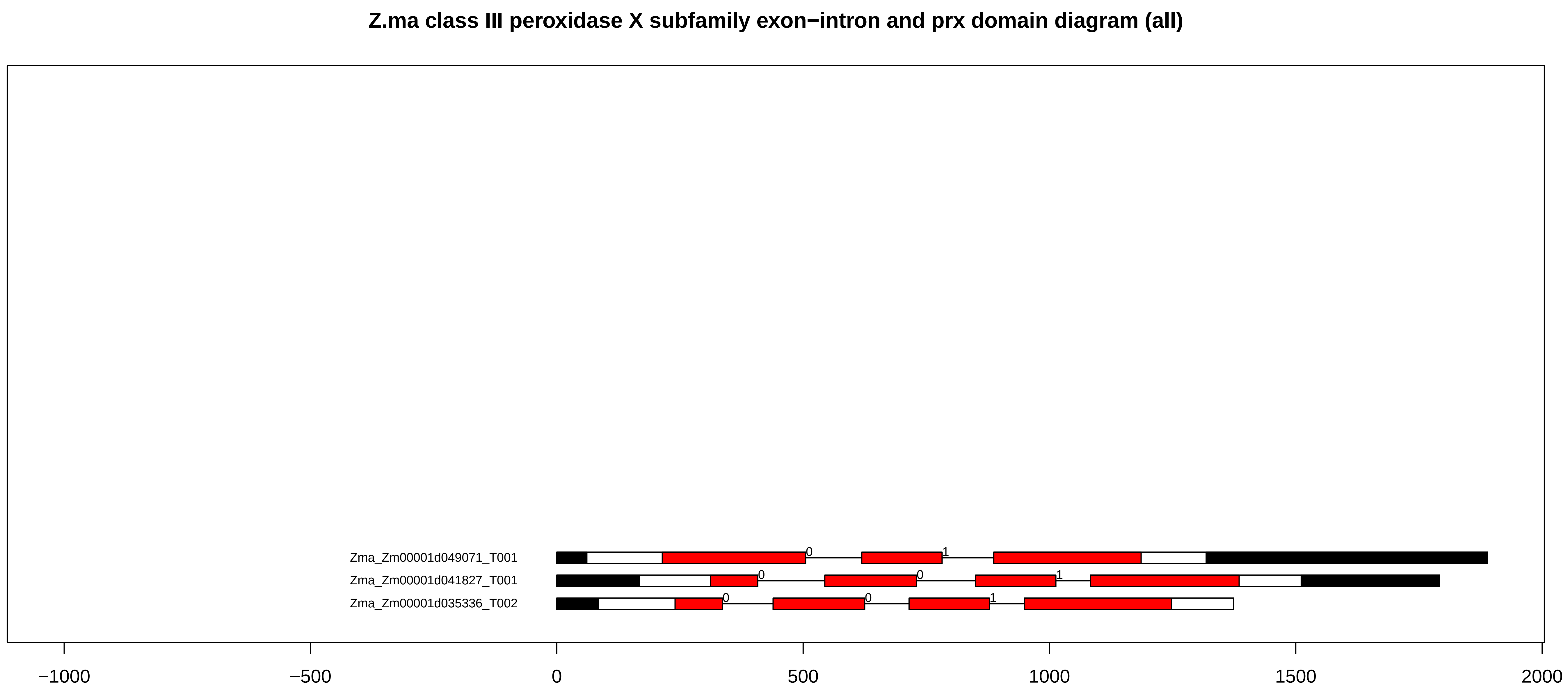

Z.ma class III peroxidase X subfamily exon−intron and prx domain diagram (all)
Zma_Zm00001d049071_T001
Zma_Zm00001d041827_T001
Zma_Zm00001d035336_T002
0
1
0
0
1
0
0
1
−1000
−500
0
500
1000
1500
2000

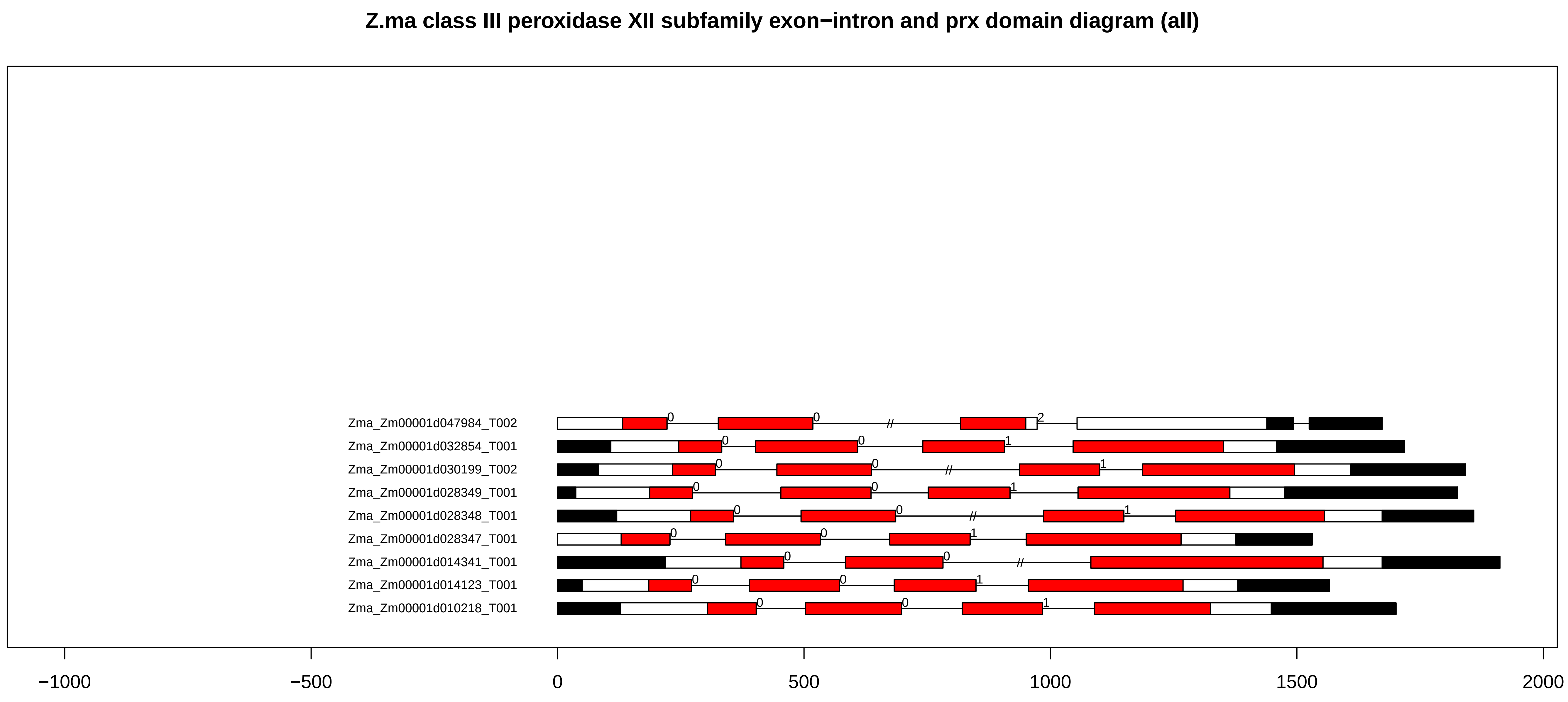
Z.ma class III peroxidase XII subfamily exon−intron and prx domain diagram (all)
Zma_Zm00001d047984_T002
Zma_Zm00001d032854_T001
Zma_Zm00001d030199_T002
Zma_Zm00001d028349_T001
Zma_Zm00001d028348_T001
Zma_Zm00001d028347_T001
Zma_Zm00001d014341_T001
Zma_Zm00001d014123_T001
Zma_Zm00001d010218_T001
−1000
−500
0
500
1000
1500
2000

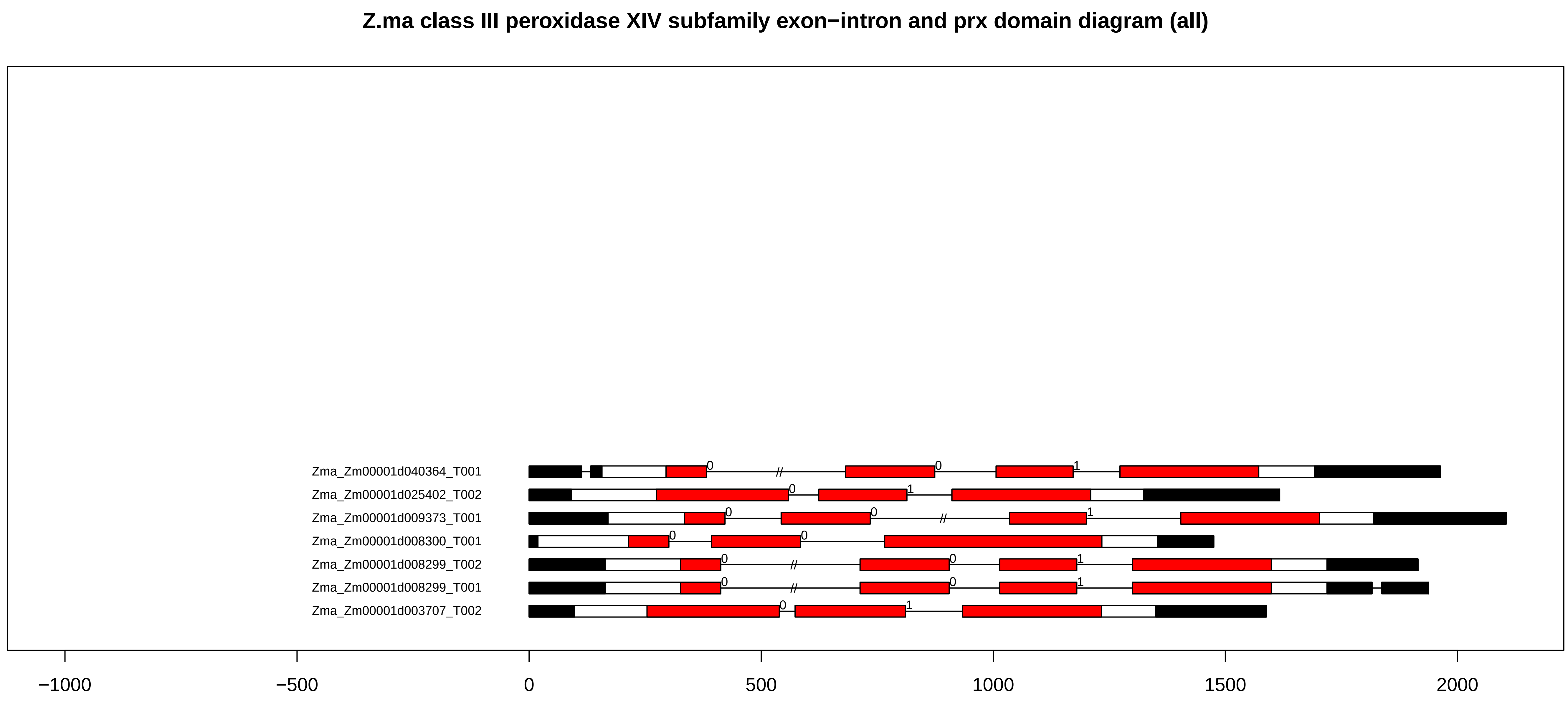

Z.ma class III peroxidase XIV subfamily exon−intron and prx domain diagram (all)
Zma_Zm00001d040364_T001
Zma_Zm00001d025402_T002
Zma_Zm00001d009373_T001
Zma_Zm00001d008300_T001
Zma_Zm00001d008299_T002
Zma_Zm00001d008299_T001
Zma_Zm00001d003707_T002
−1000
−500
0
500
1000
1500
2000

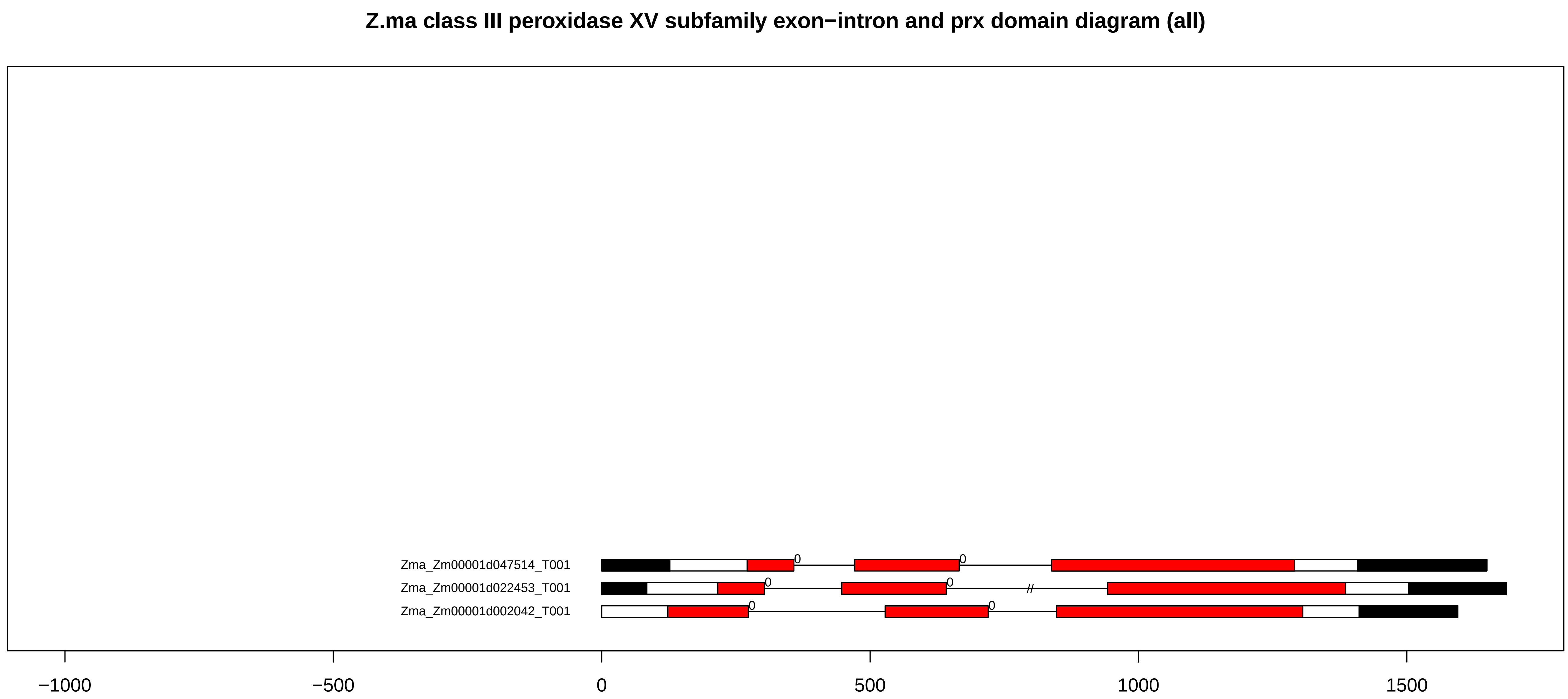
Z.ma class III peroxidase XV subfamily exon−intron and prx domain diagram (all)
Zma_Zm00001d047514_T001
Zma_Zm00001d022453_T001
Zma_Zm00001d002042_T001
0
0
0
0
0
0
−1000
−500
0
500
1000
1500

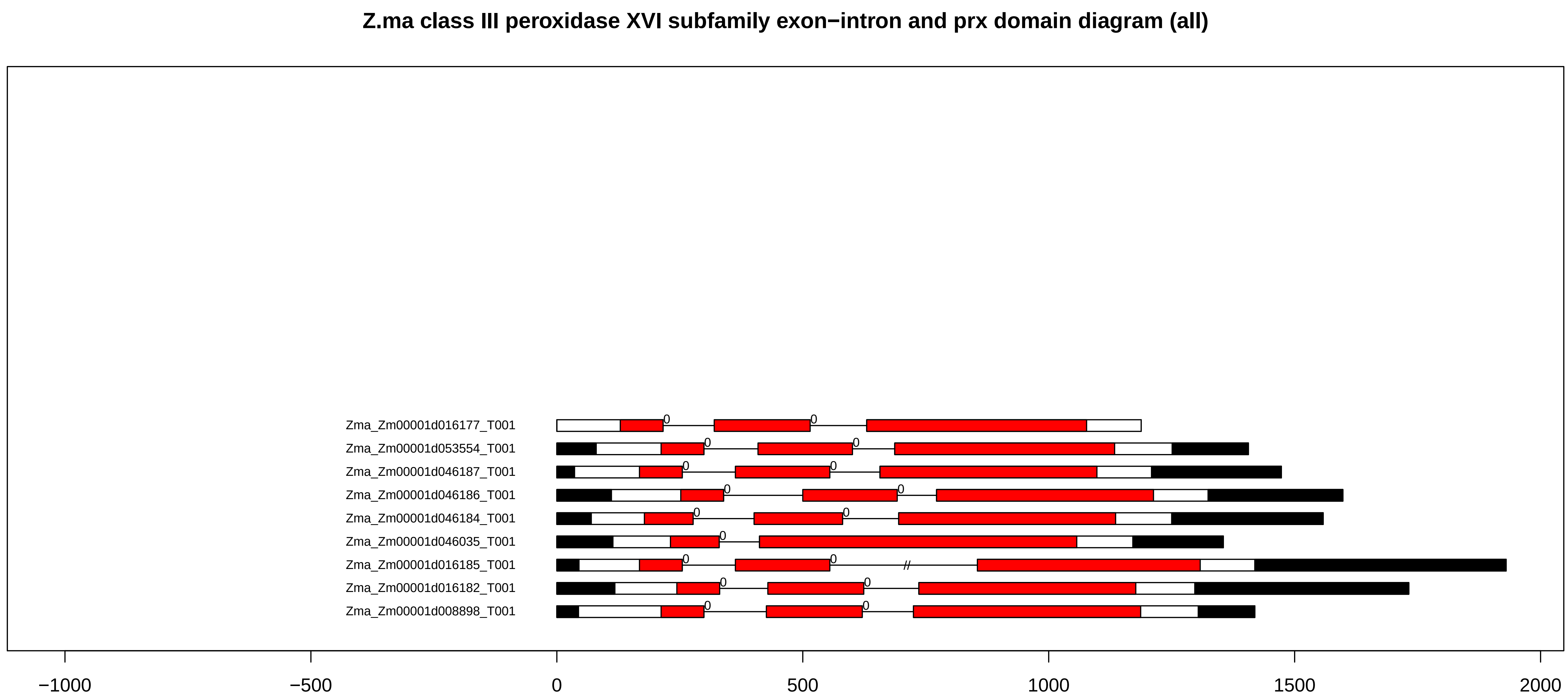

Z.ma class III peroxidase XVI subfamily exon−intron and prx domain diagram (all)
Zma_Zm00001d016177_T001
Zma_Zm00001d053554_T001
Zma_Zm00001d046187_T001
Zma_Zm00001d046186_T001
Zma_Zm00001d046184_T001
Zma_Zm00001d046035_T001
Zma_Zm00001d016185_T001
Zma_Zm00001d016182_T001
Zma_Zm00001d008898_T001
−1000
−500
0
500
1000
1500
2000

## Z.ma class III peroxidase XVII subfamily exon−intron and prx domain diagram (all)

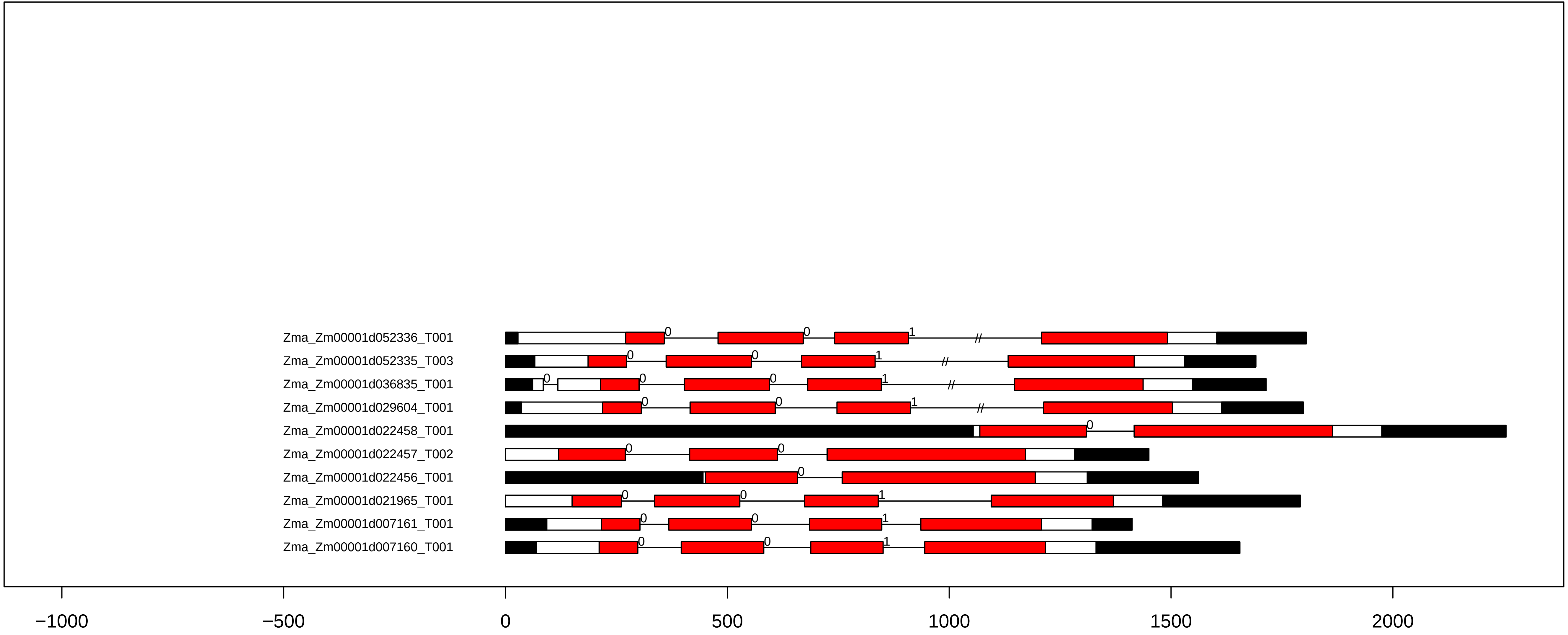

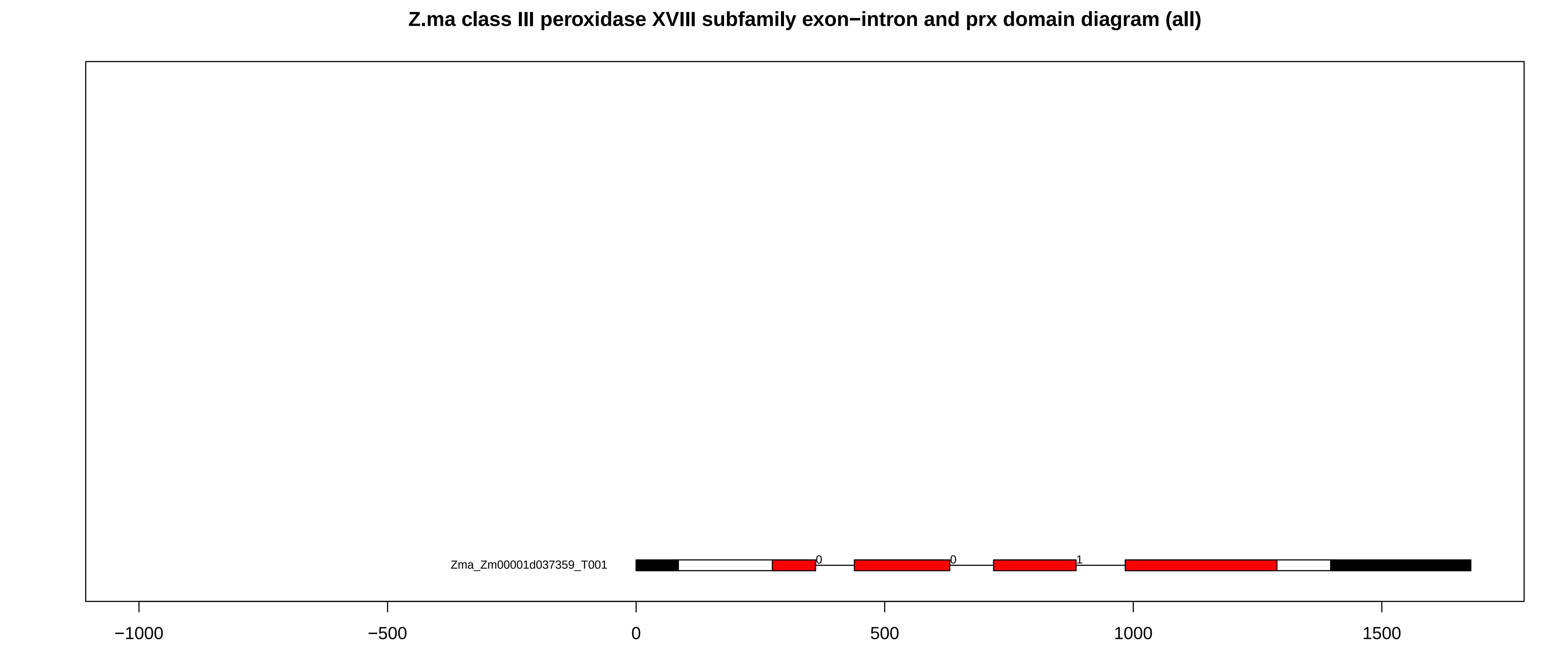
Z.ma class III peroxidase XVIII subfamily exon−intron and prx domain diagram (all)
Zma_Zm00001d037359_T001
0
0
1
−1000
−500
0
500
1000
1500

## Z.ma class III peroxidase unclear_classification subfamily exon−intron and prx domain diagram (all)

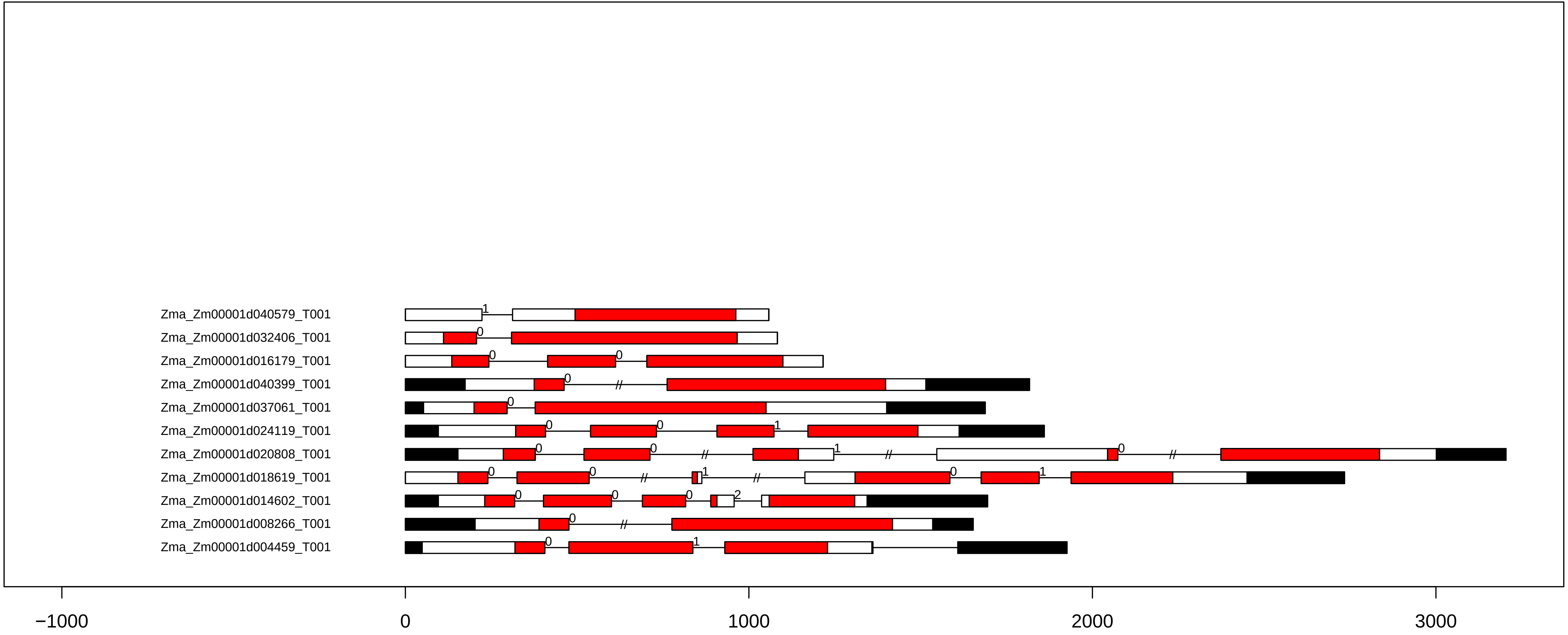

## A.th class III peroxidase I subfamily exon−intron and prx domain diagram (all)

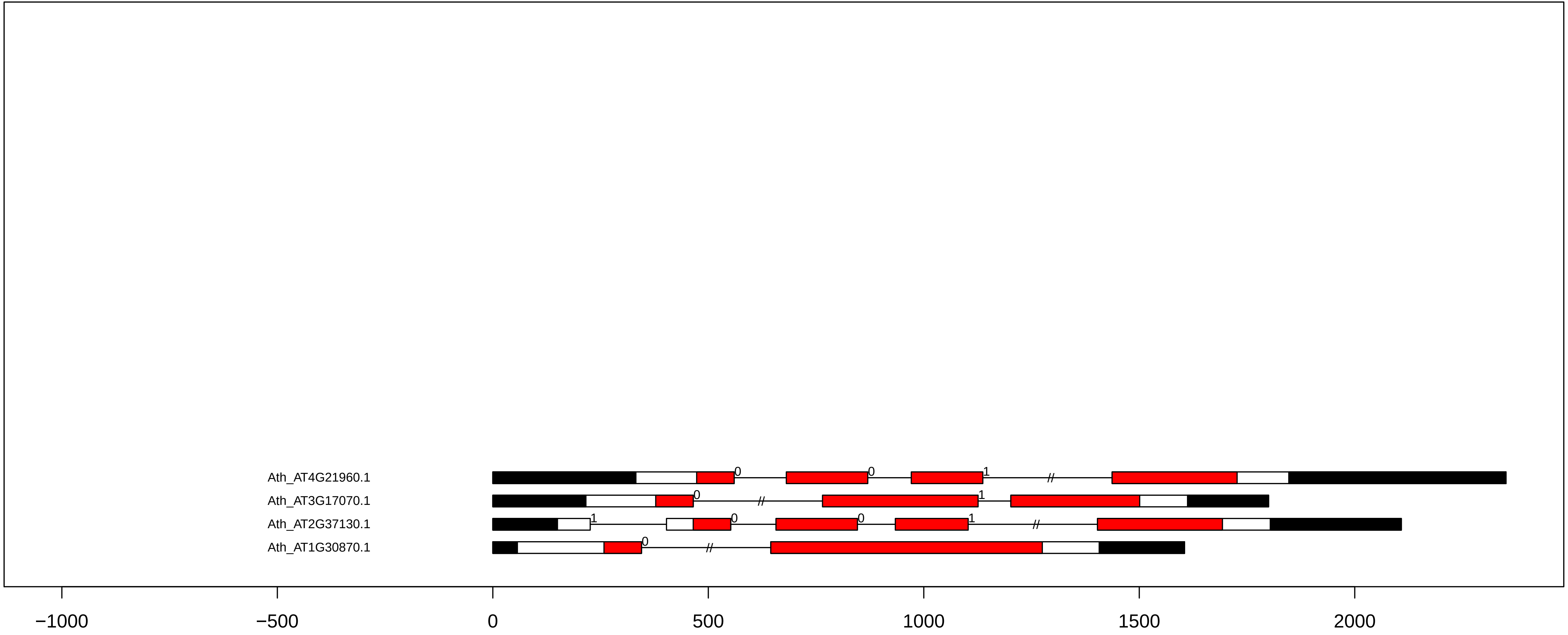

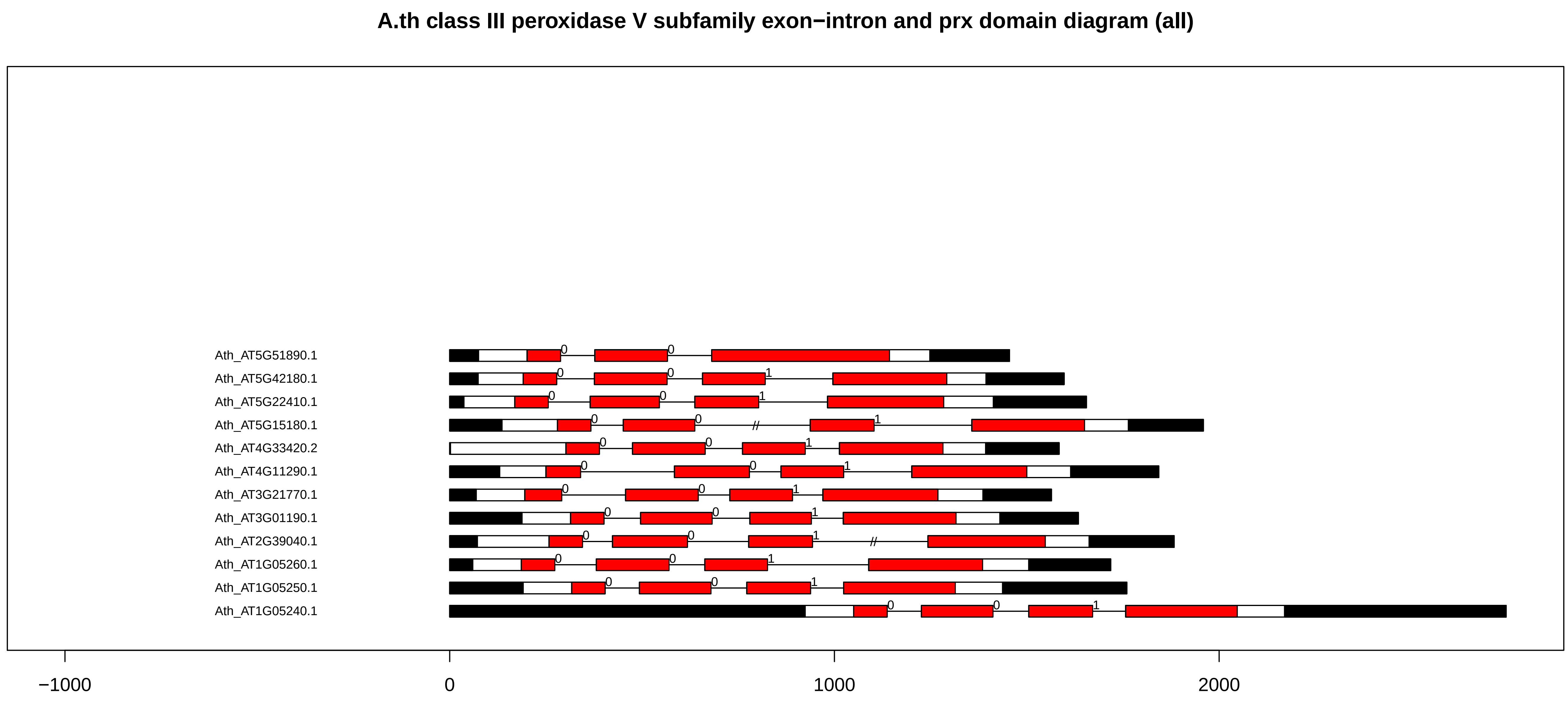
A.th class III peroxidase V subfamily exon−intron and prx domain diagram (all)
Ath_AT5G51890.1
Ath_AT5G42180.1
Ath_AT5G22410.1
Ath_AT5G15180.1
Ath_AT4G33420.2
Ath_AT4G11290.1
Ath_AT3G21770.1
Ath_AT3G01190.1
Ath_AT2G39040.1
Ath_AT1G05260.1
Ath_AT1G05250.1
Ath_AT1G05240.1
−1000
0
1000
2000

## A.th class III peroxidase VI subfamily exon−intron and prx domain diagram (all)

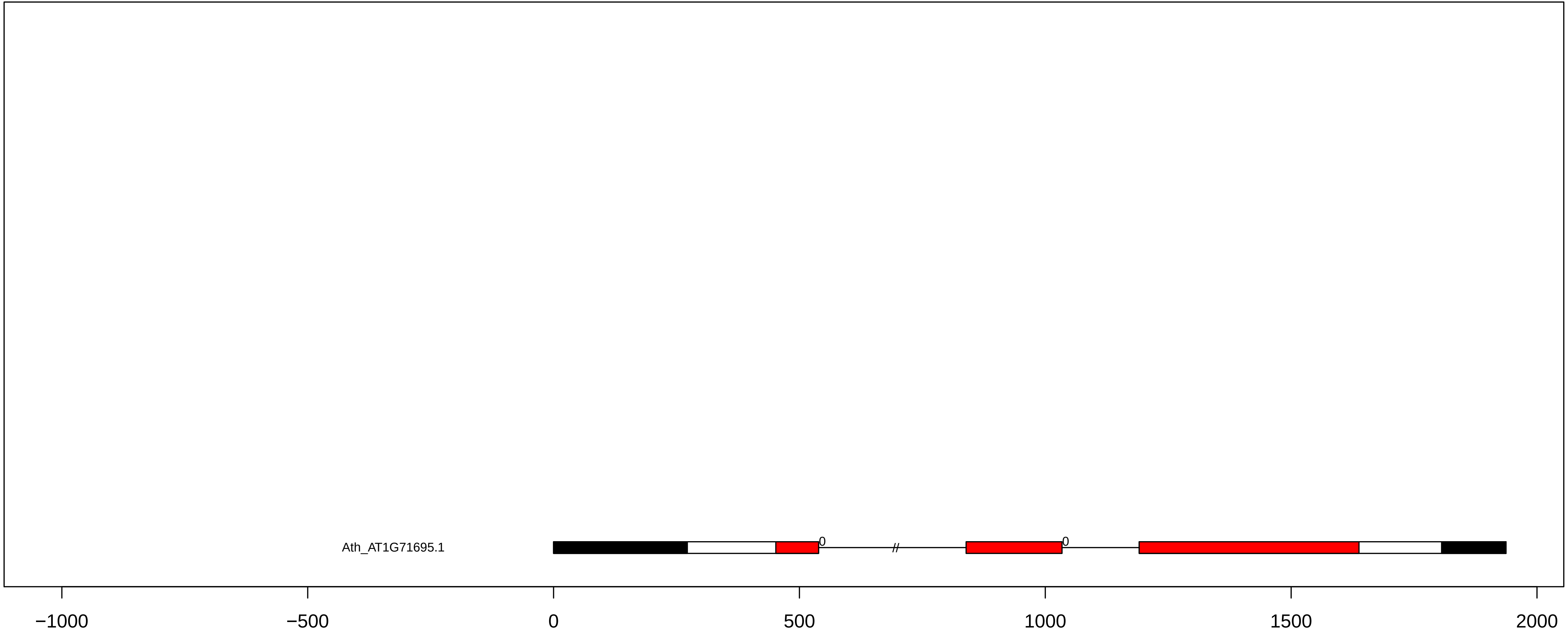

# A.th class III peroxidase VII subfamily exon−intron and prx domain diagram (all)

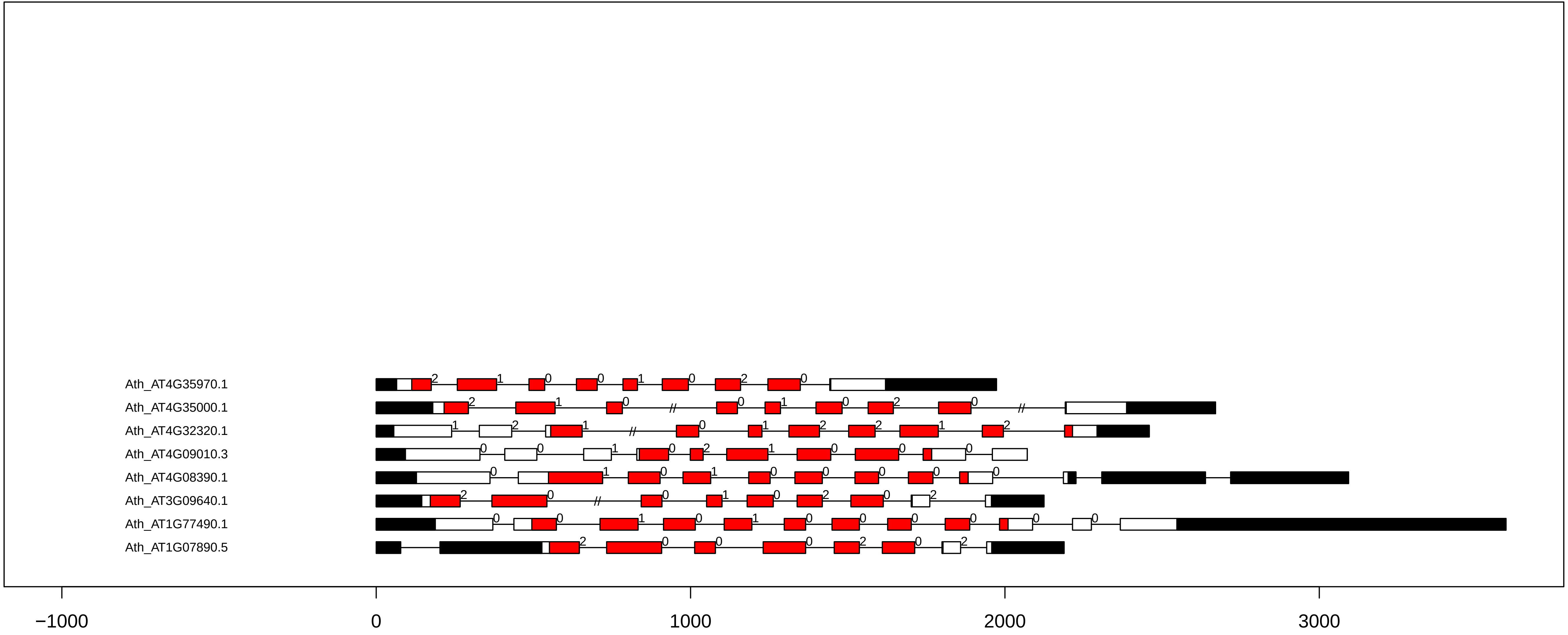

# A.th class III peroxidase IX subfamily exon−intron and prx domain diagram (all)

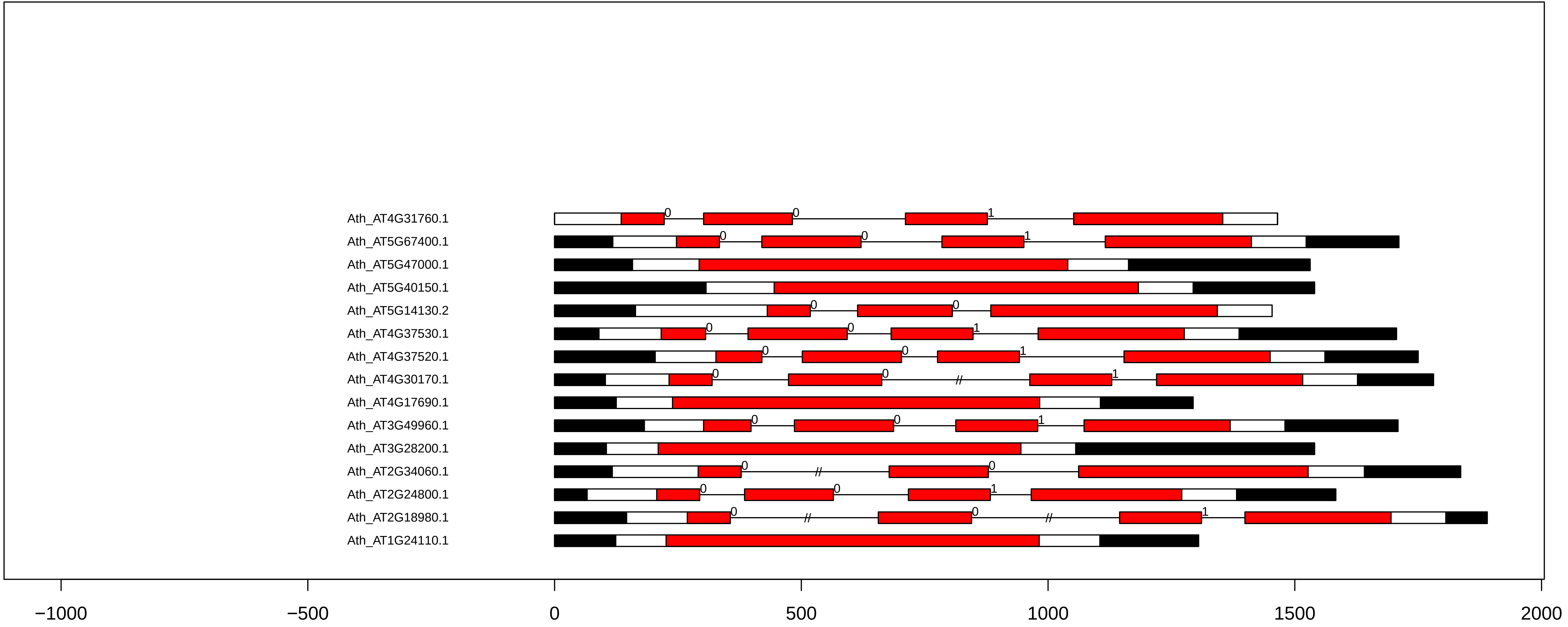

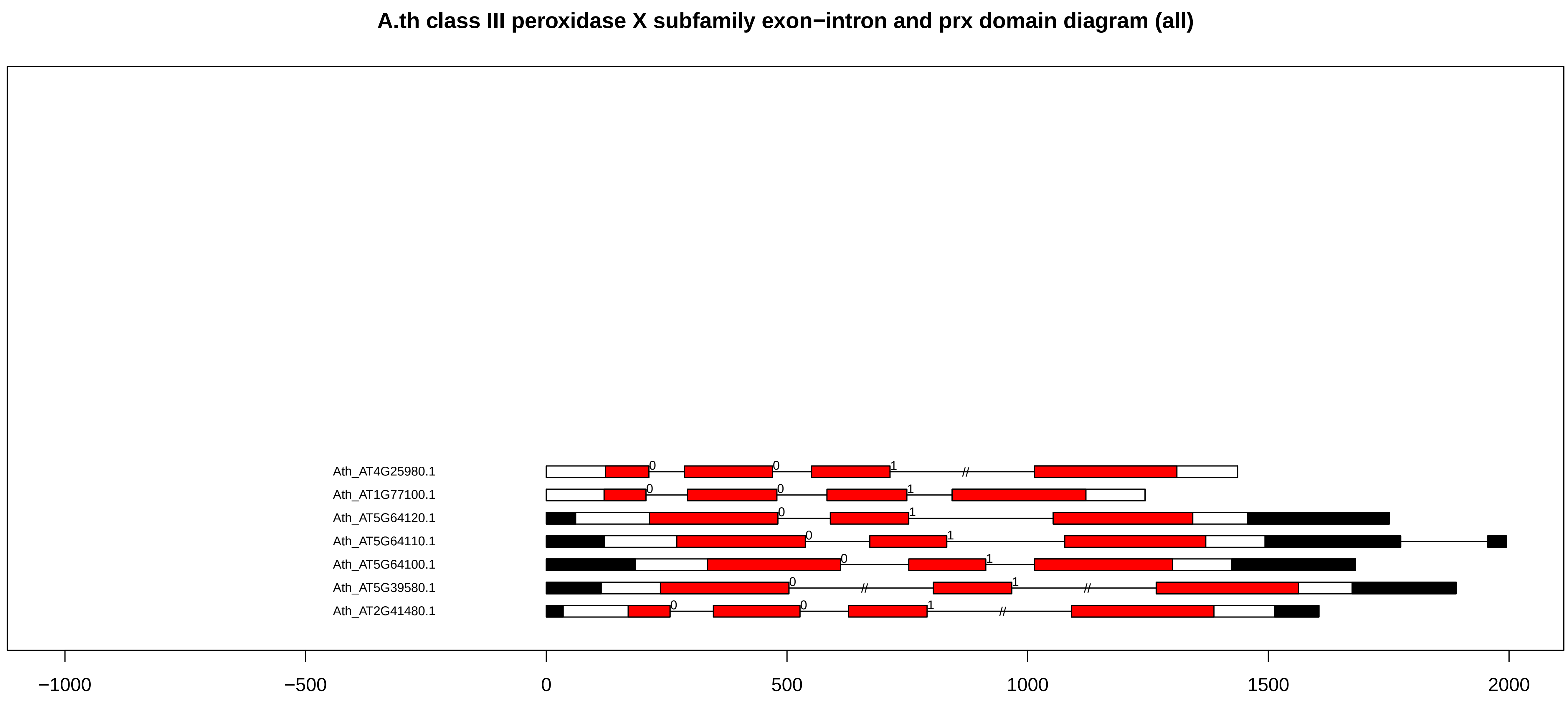
A.th class III peroxidase X subfamily exon−intron and prx domain diagram (all)
Ath_AT4G25980.1
Ath_AT1G77100.1
Ath_AT5G64120.1
Ath_AT5G64110.1
Ath_AT5G64100.1
Ath_AT5G39580.1
Ath_AT2G41480.1
−1000
−500
0
500
1000
1500
2000

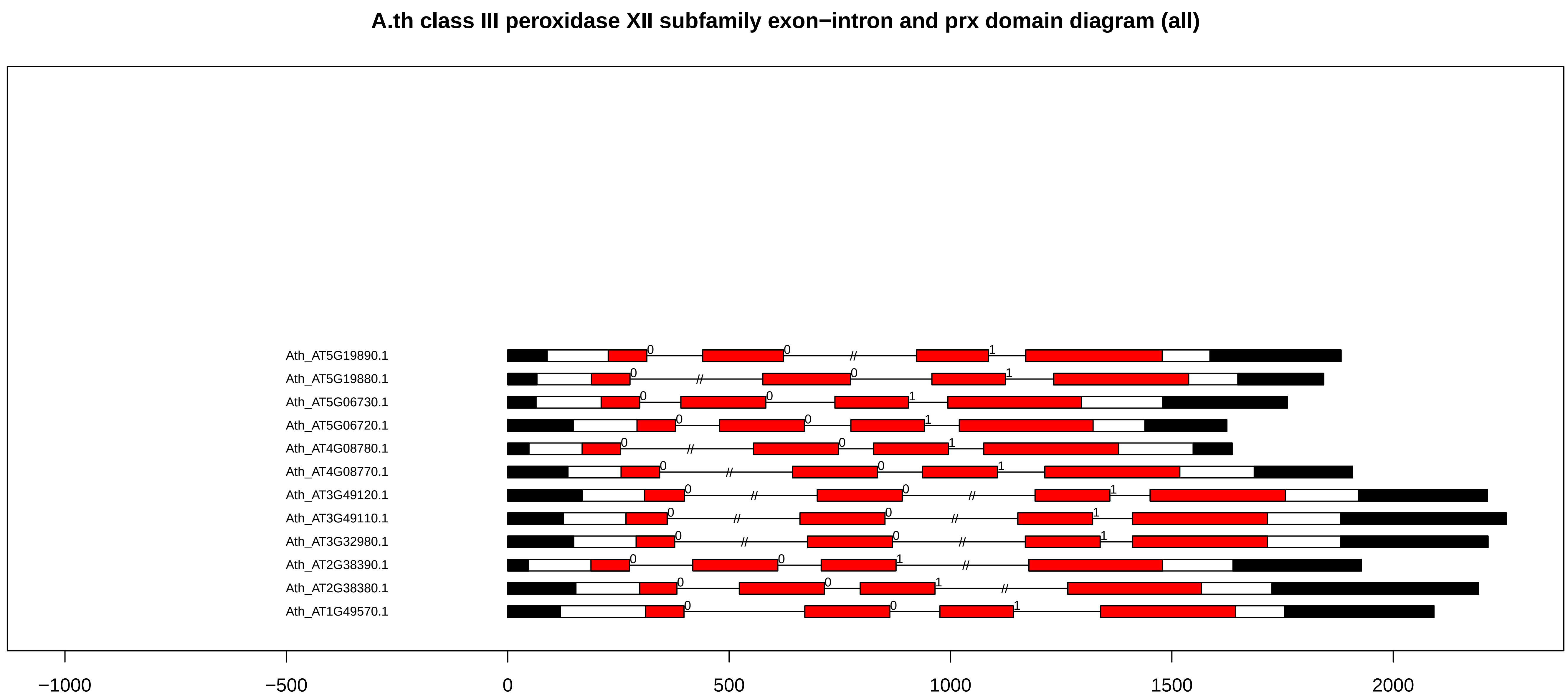

A.th class III peroxidase XII subfamily exon−intron and prx domain diagram (all)
Ath_AT5G19890.1
Ath_AT5G19880.1
Ath_AT5G06730.1
Ath_AT5G06720.1
Ath_AT4G08780.1
Ath_AT4G08770.1
Ath_AT3G49120.1
Ath_AT3G49110.1
Ath_AT3G32980.1
Ath_AT2G38390.1
Ath_AT2G38380.1
Ath_AT1G49570.1
−1000
−500
0
500
1000
1500
2000

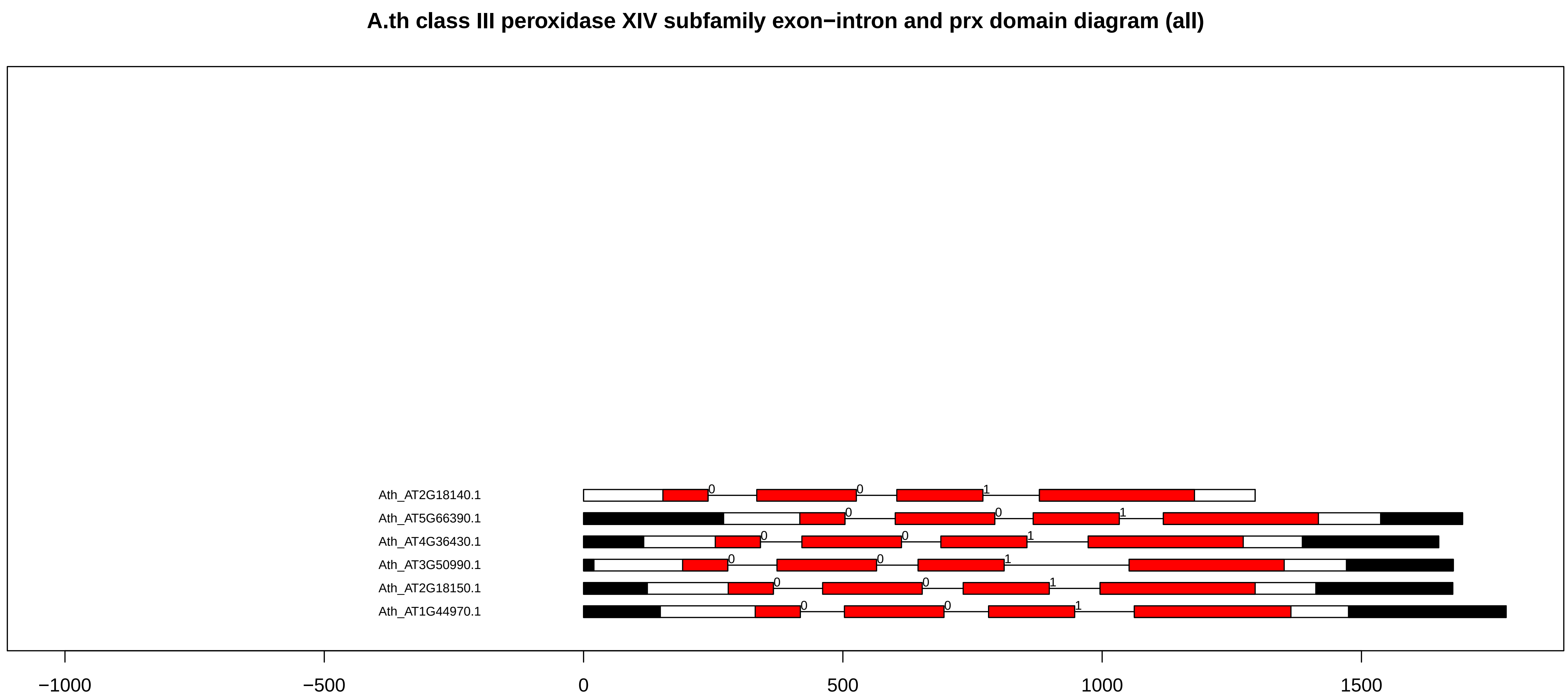
A.th class III peroxidase XIV subfamily exon−intron and prx domain diagram (all)
Ath_AT2G18140.1
Ath_AT5G66390.1
Ath_AT4G36430.1
Ath_AT3G50990.1
Ath_AT2G18150.1
Ath_AT1G44970.1
−1000
−500
0
500
1000
1500

# A.th class III peroxidase XVII subfamily exon−intron and prx domain diagram (all)

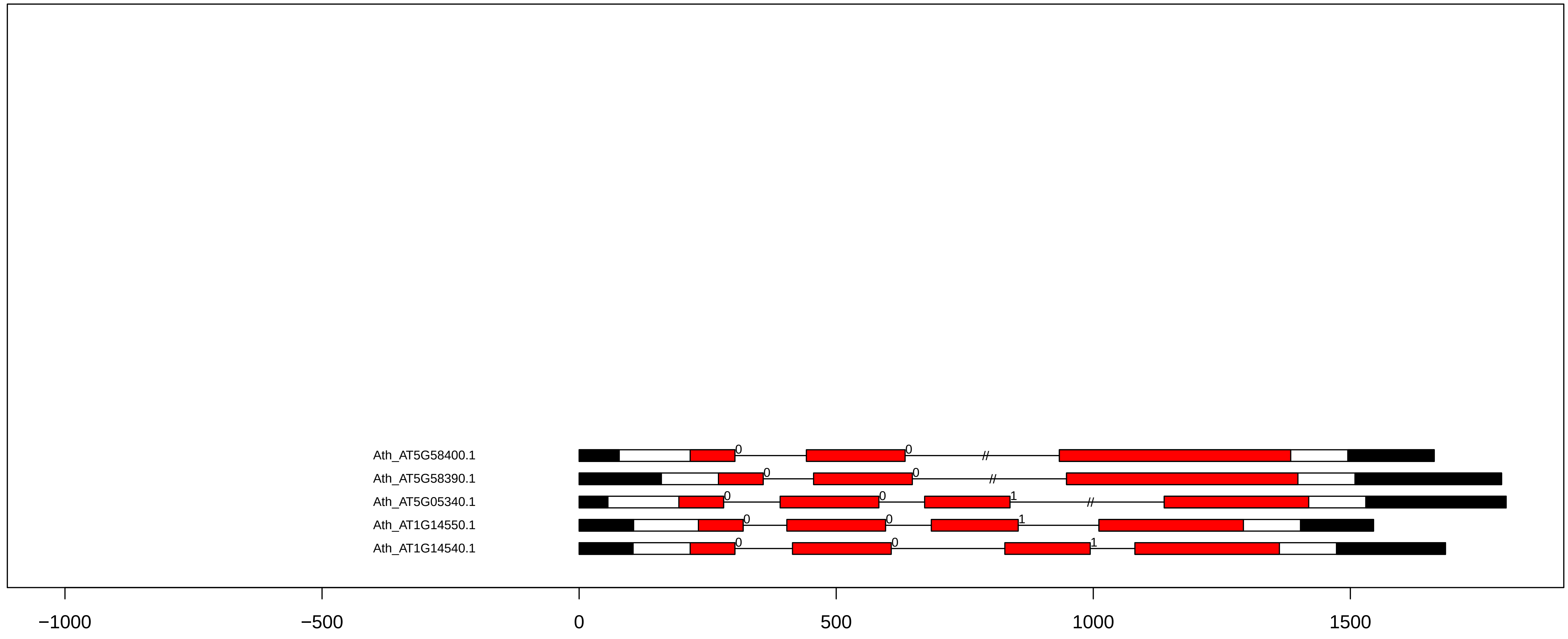

## A.th class III peroxidase XVIII subfamily exon−intron and prx domain diagram (all)

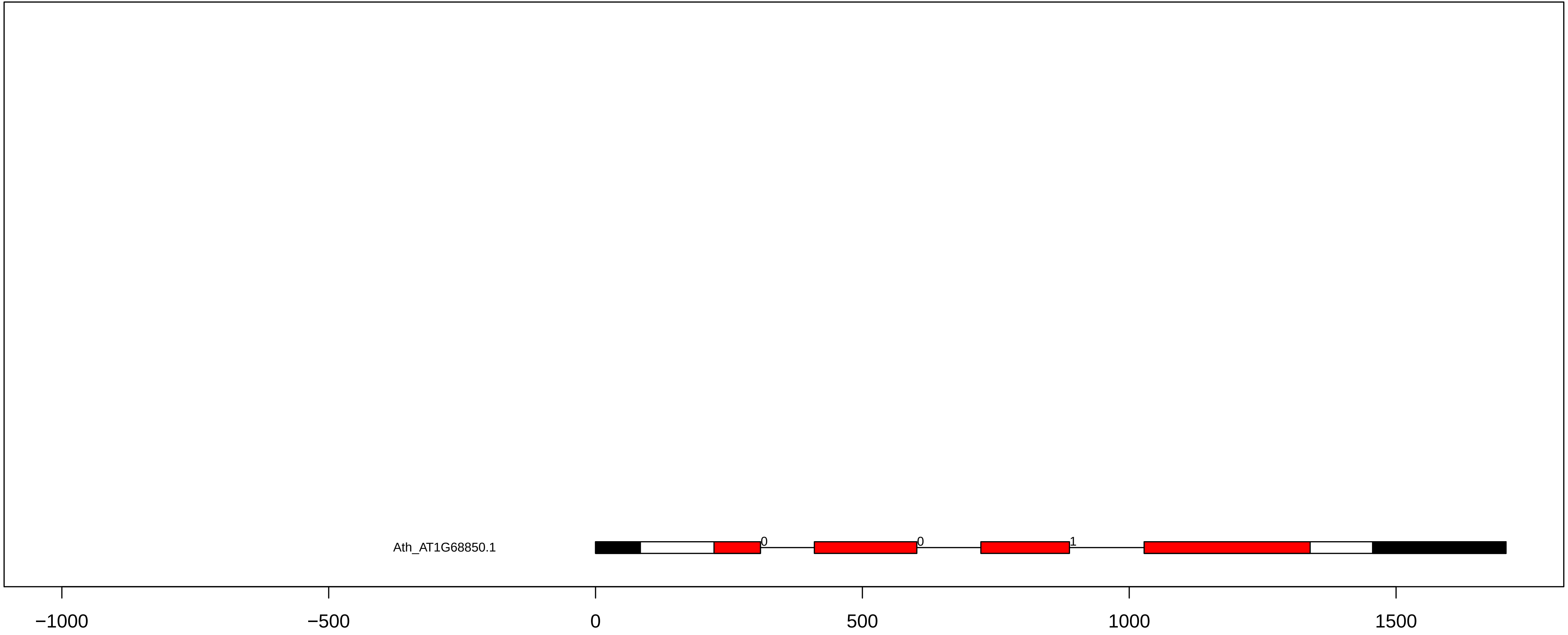

A.th class III peroxidase unclear_classification subfamily exon−intron and prx domain diagram (all)

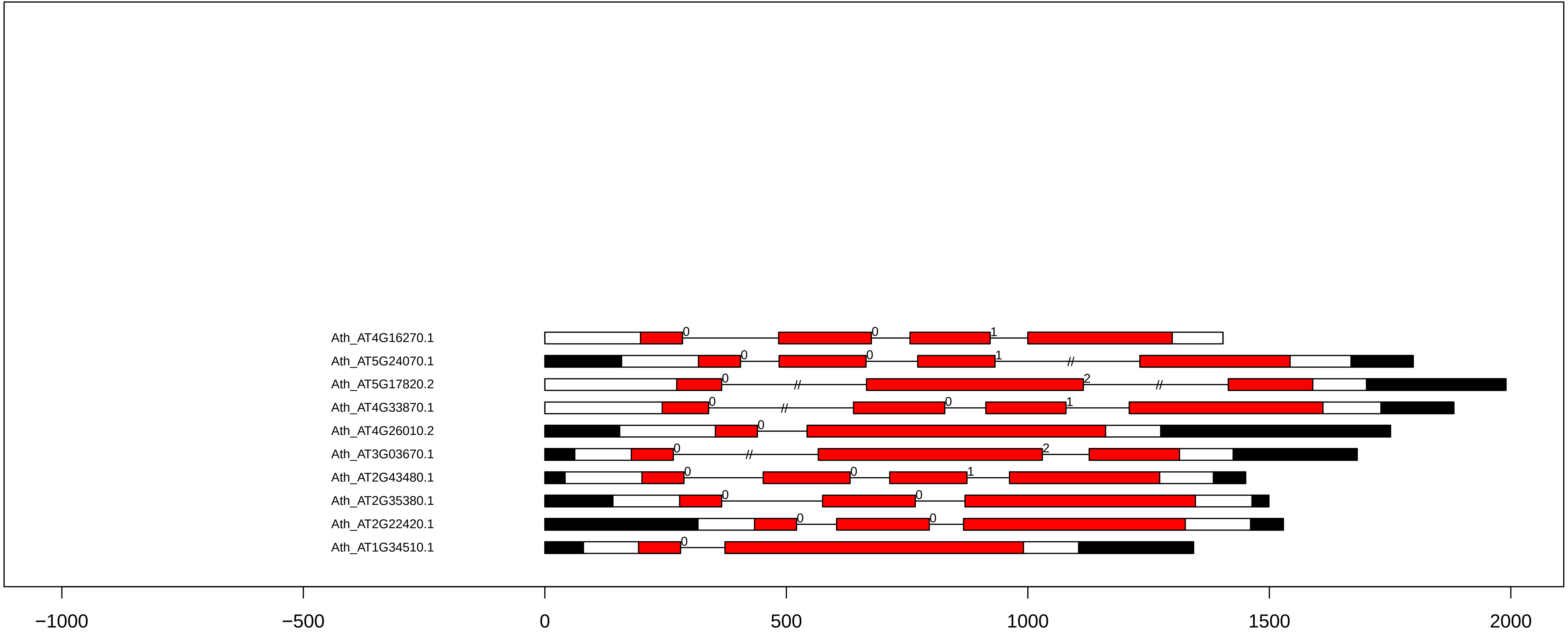

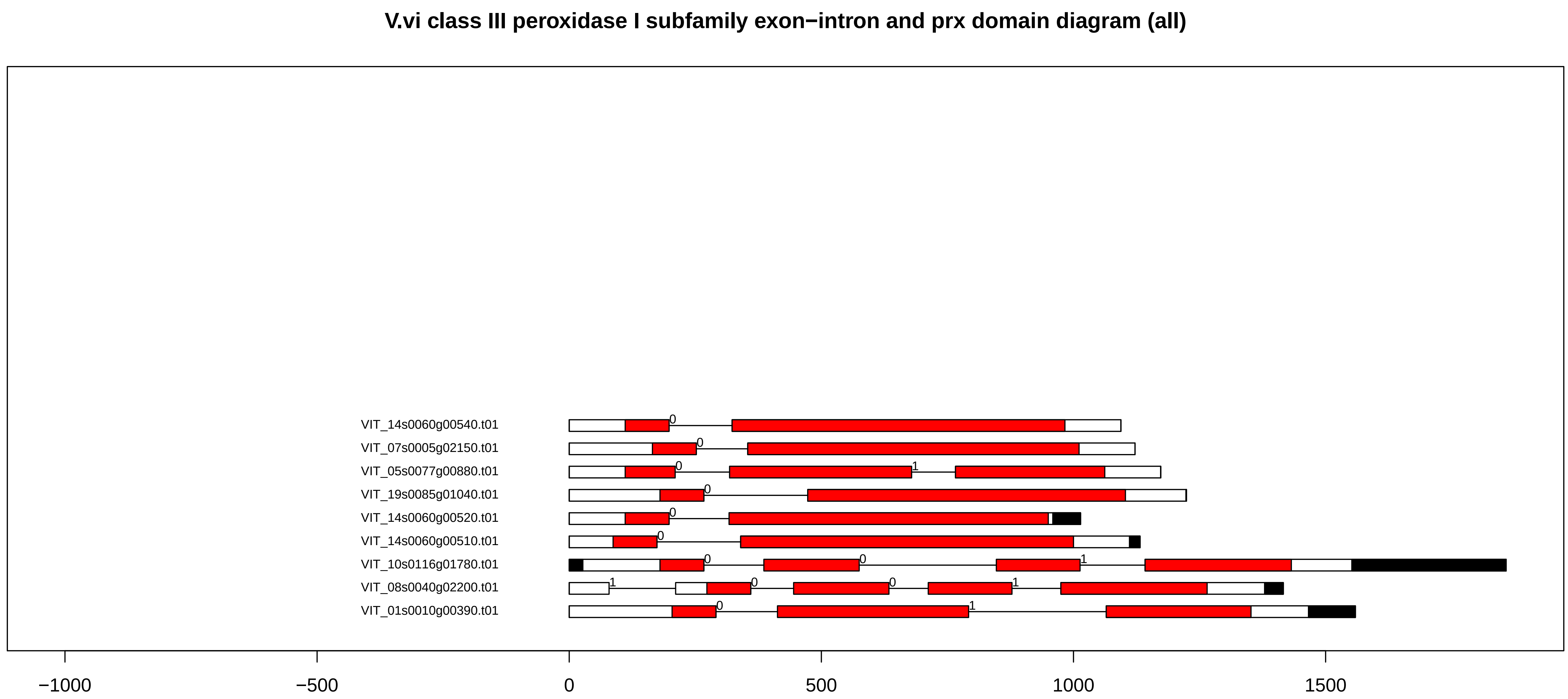

V.vi class III peroxidase I subfamily exon−intron and prx domain diagram (all)
VIT_14s0060g00540.t01
VIT_07s0005g02150.t01
VIT_05s0077g00880.t01
VIT_19s0085g01040.t01
VIT_14s0060g00520.t01
VIT_14s0060g00510.t01
VIT_10s0116g01780.t01
VIT_08s0040g02200.t01
VIT_01s0010g00390.t01
−1000
−500
0
500
1000
1500

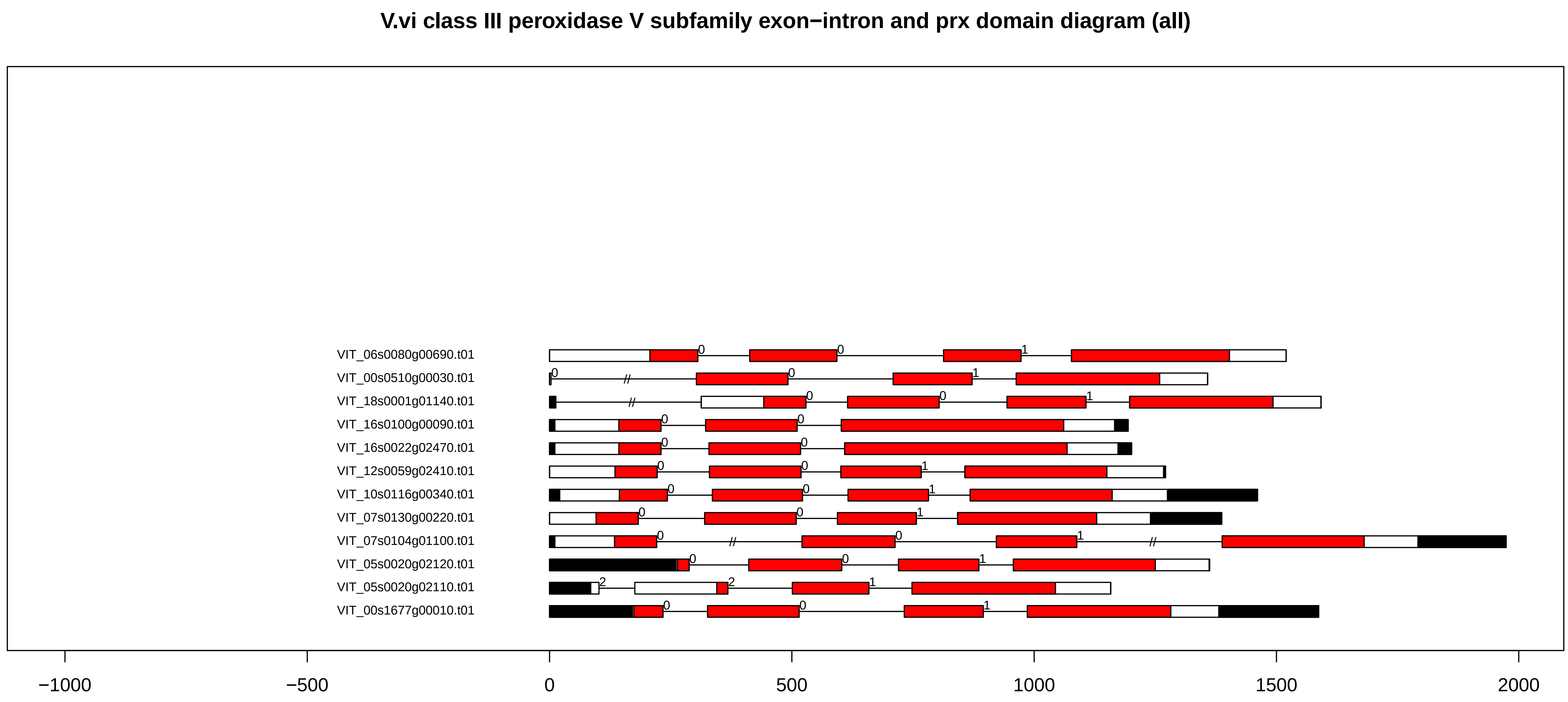

V.vi class III peroxidase V subfamily exon−intron and prx domain diagram (all)
VIT_06s0080g00690.t01
VIT_00s0510g00030.t01
VIT_18s0001g01140.t01
VIT_16s0100g00090.t01
VIT_16s0022g02470.t01
VIT_12s0059g02410.t01
VIT_10s0116g00340.t01
VIT_07s0130g00220.t01
VIT_07s0104g01100.t01
VIT_05s0020g02120.t01
VIT_05s0020g02110.t01
VIT_00s1677g00010.t01
−1000
−500
0
500
1000
1500
2000

V.vi class III peroxidase VI subfamily exon−intron and prx domain diagram (all)

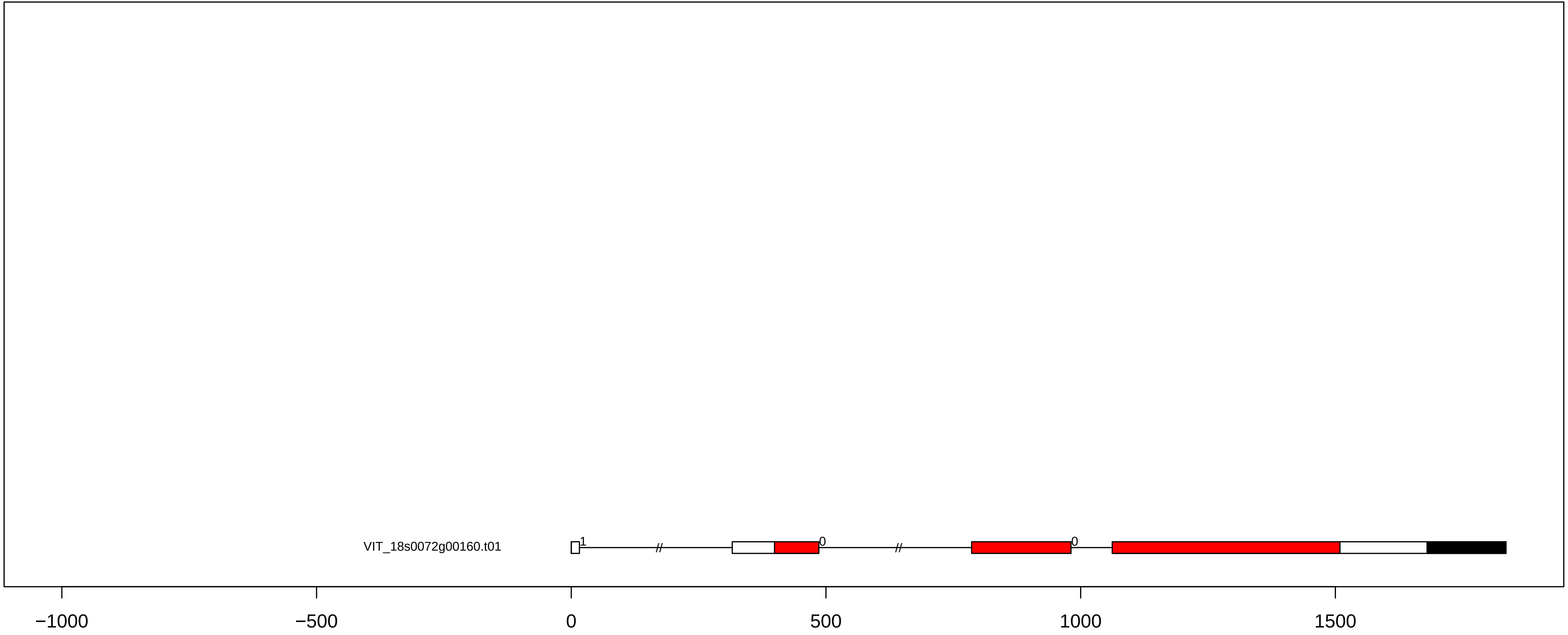

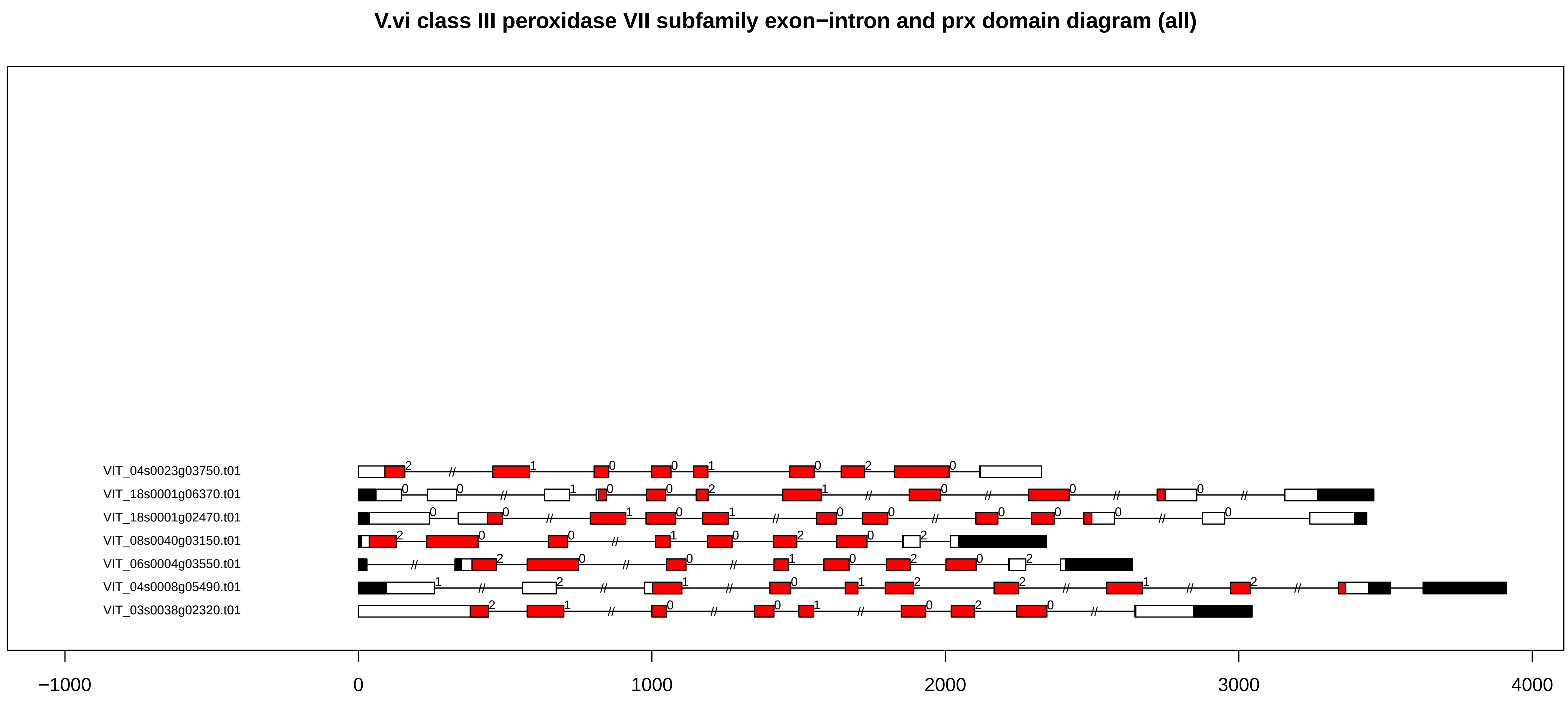

V.vi class III peroxidase VII subfamily exon−intron and prx domain diagram (all)
VIT_04s0023g03750.t01
VIT_18s0001g06370.t01
VIT_18s0001g02470.t01
VIT_08s0040g03150.t01
VIT_06s0004g03550.t01
VIT_04s0008g05490.t01
VIT_03s0038g02320.t01
−1000
0
1000
2000
3000
4000

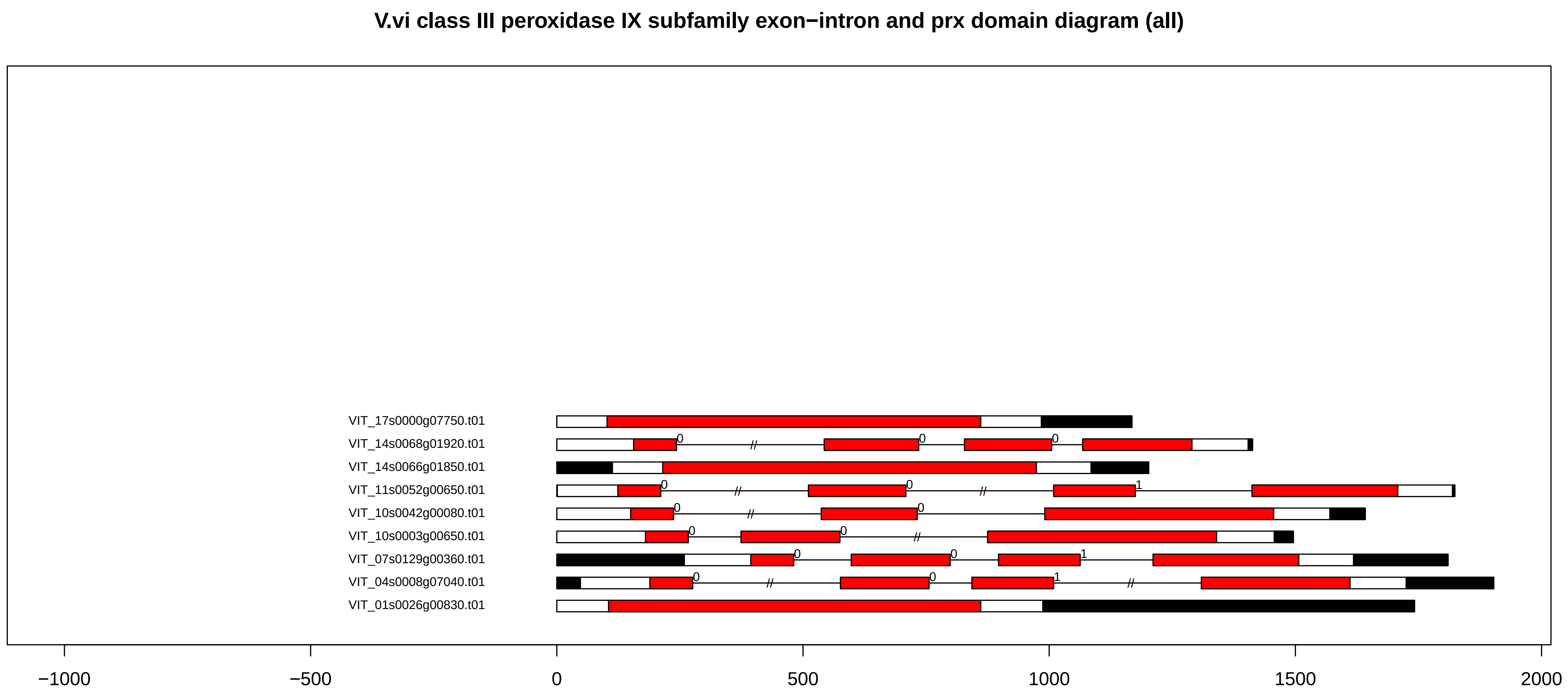
V.vi class III peroxidase IX subfamily exon−intron and prx domain diagram (all)
VIT_17s0000g07750.t01
VIT_14s0068g01920.t01
VIT_14s0066g01850.t01
VIT_11s0052g00650.t01
VIT_10s0042g00080.t01
VIT_10s0003g00650.t01
VIT_07s0129g00360.t01
VIT_04s0008g07040.t01
VIT_01s0026g00830.t01
−1000
−500
0
500
1000
1500
2000

# V.vi class III peroxidase X subfamily exon−intron and prx domain diagram (all)

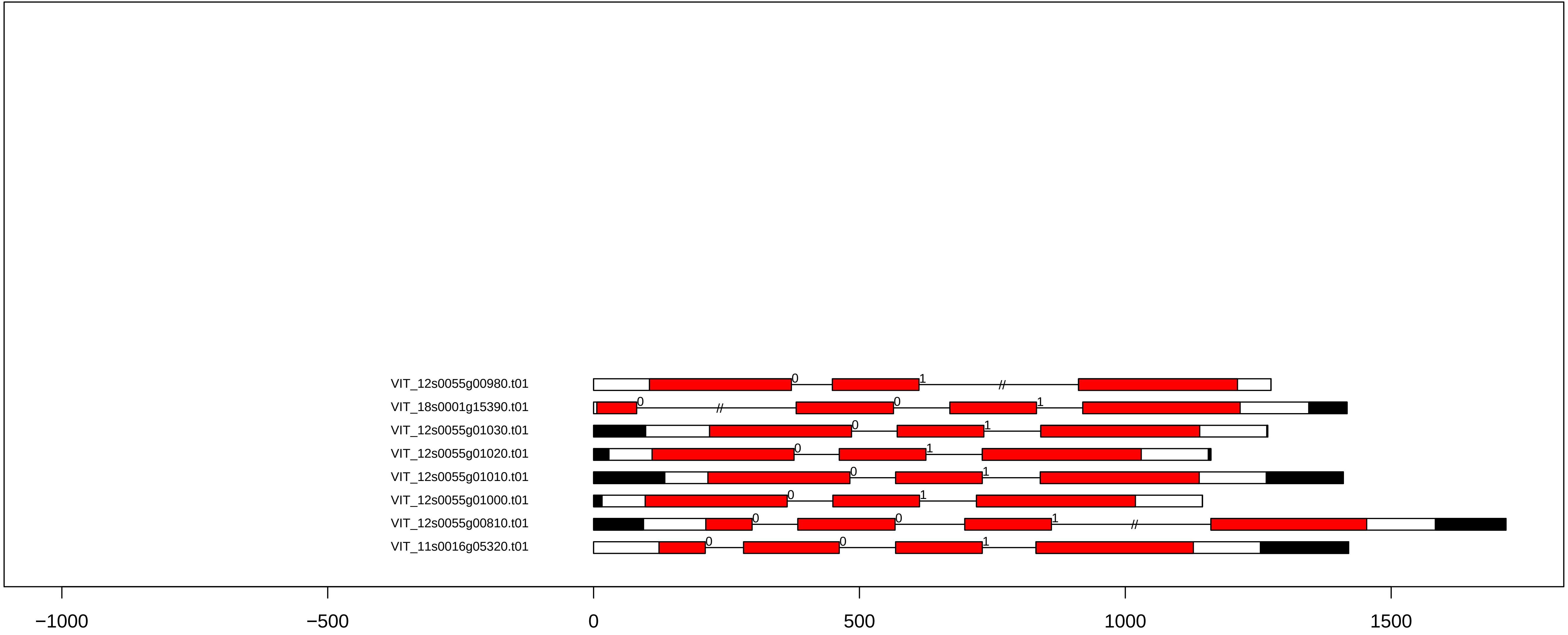

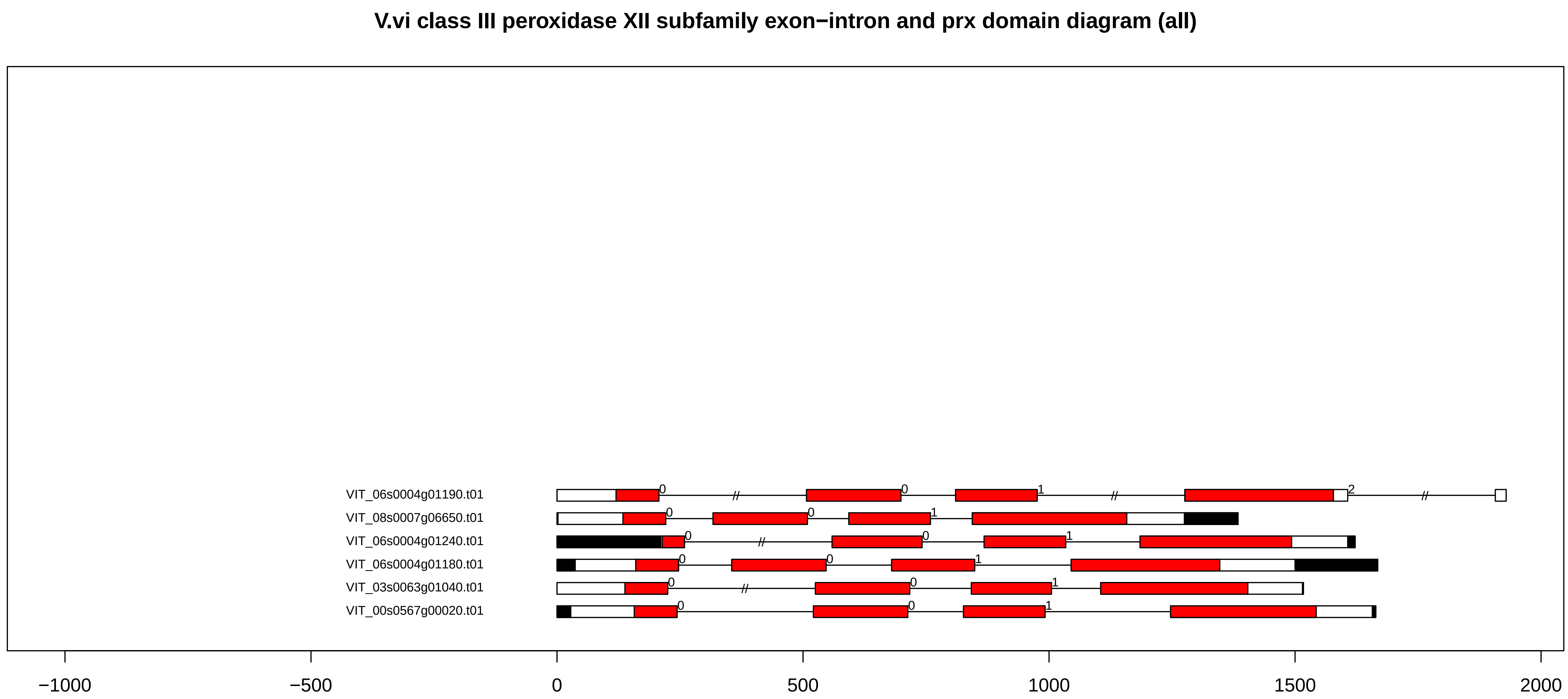
V.vi class III peroxidase XII subfamily exon−intron and prx domain diagram (all)
VIT_06s0004g01190.t01
VIT_08s0007g06650.t01
VIT_06s0004g01240.t01
VIT_06s0004g01180.t01
VIT_03s0063g01040.t01
VIT_00s0567g00020.t01
−1000
−500
0
500
1000
1500
2000

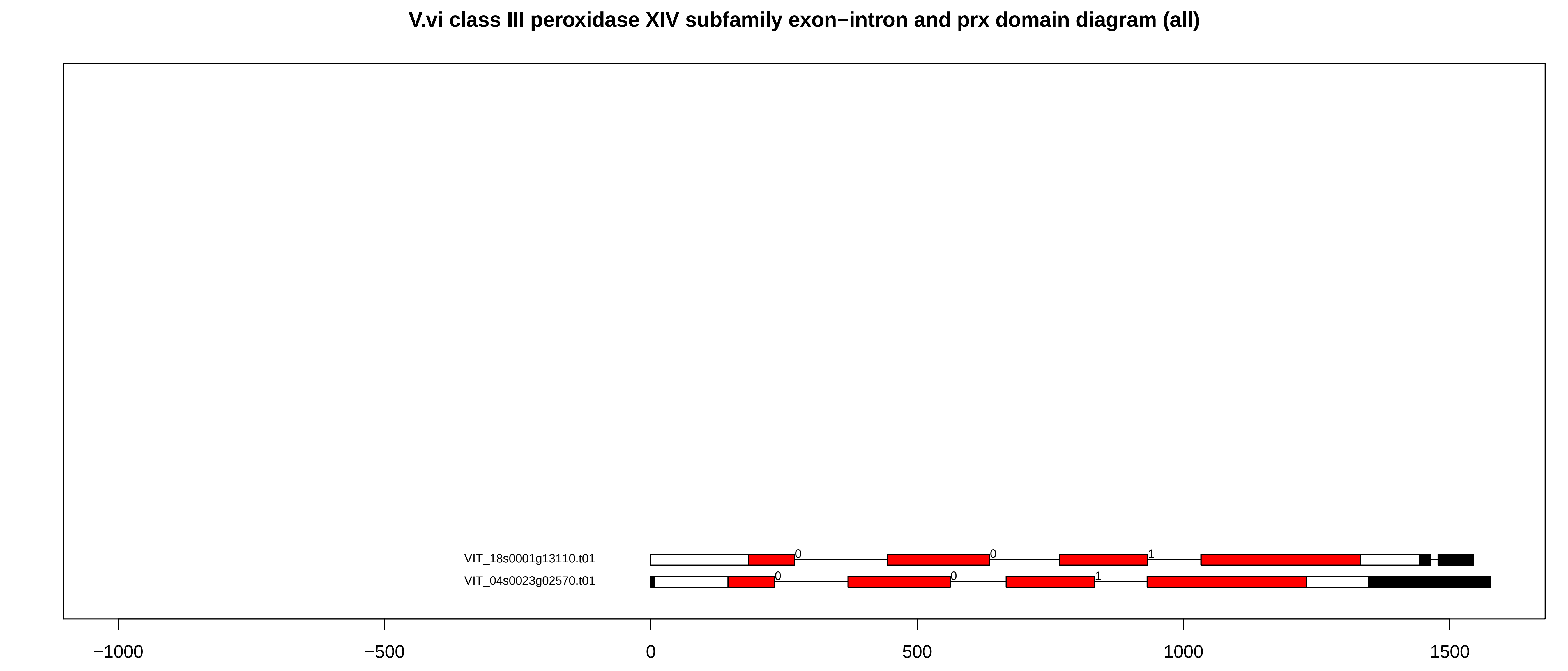
V.vi class III peroxidase XIV subfamily exon−intron and prx domain diagram (all)
VIT_18s0001g13110.t01
VIT_04s0023g02570.t01
0
0
1
0
0
1
−1000
−500
0
500
1000
1500

## V.vi class III peroxidase XV subfamily exon−intron and prx domain diagram (all)

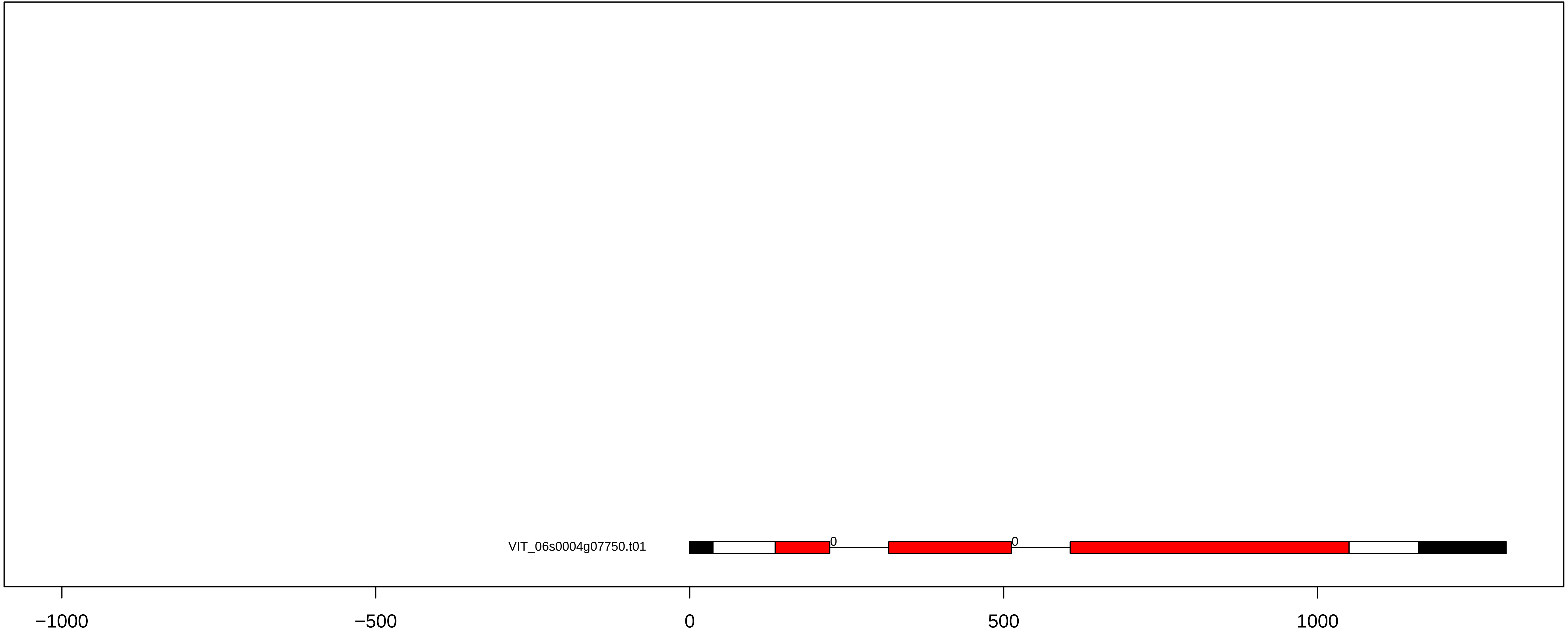

# V.vi class III peroxidase XVI subfamily exon−intron and prx domain diagram (all)

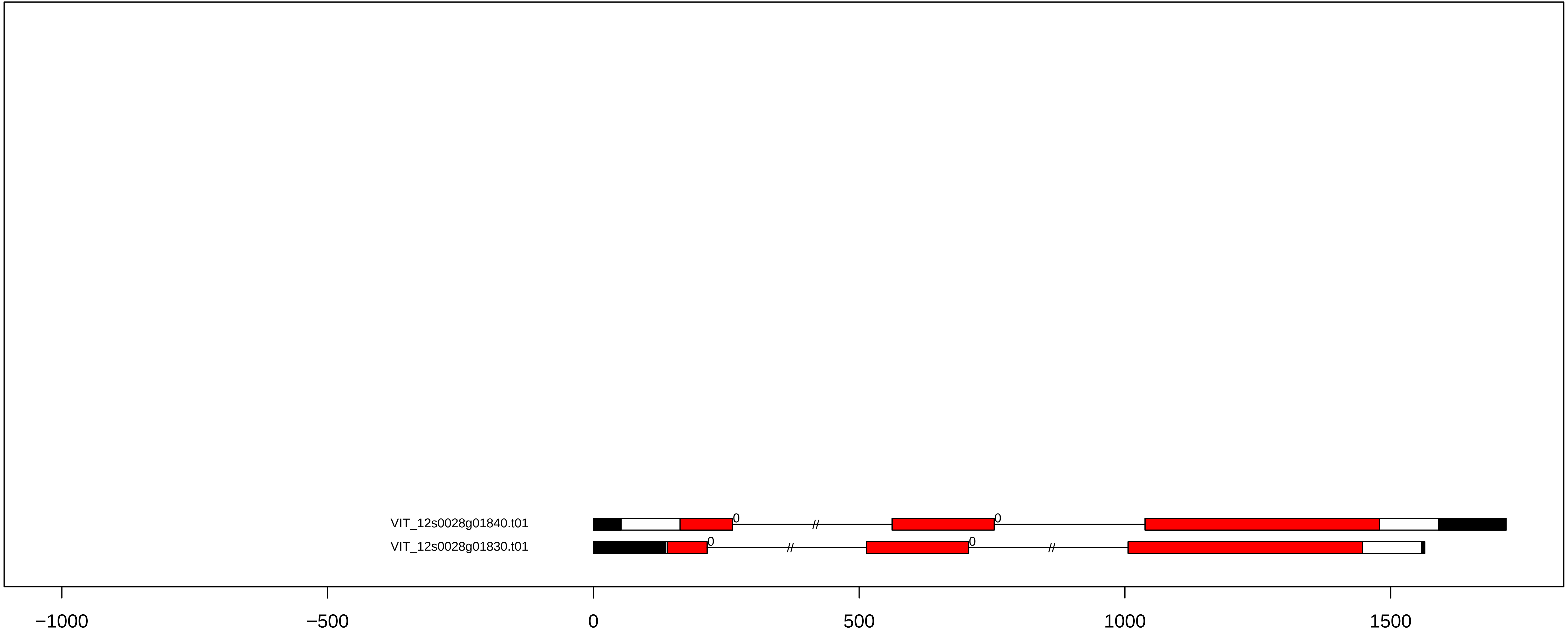

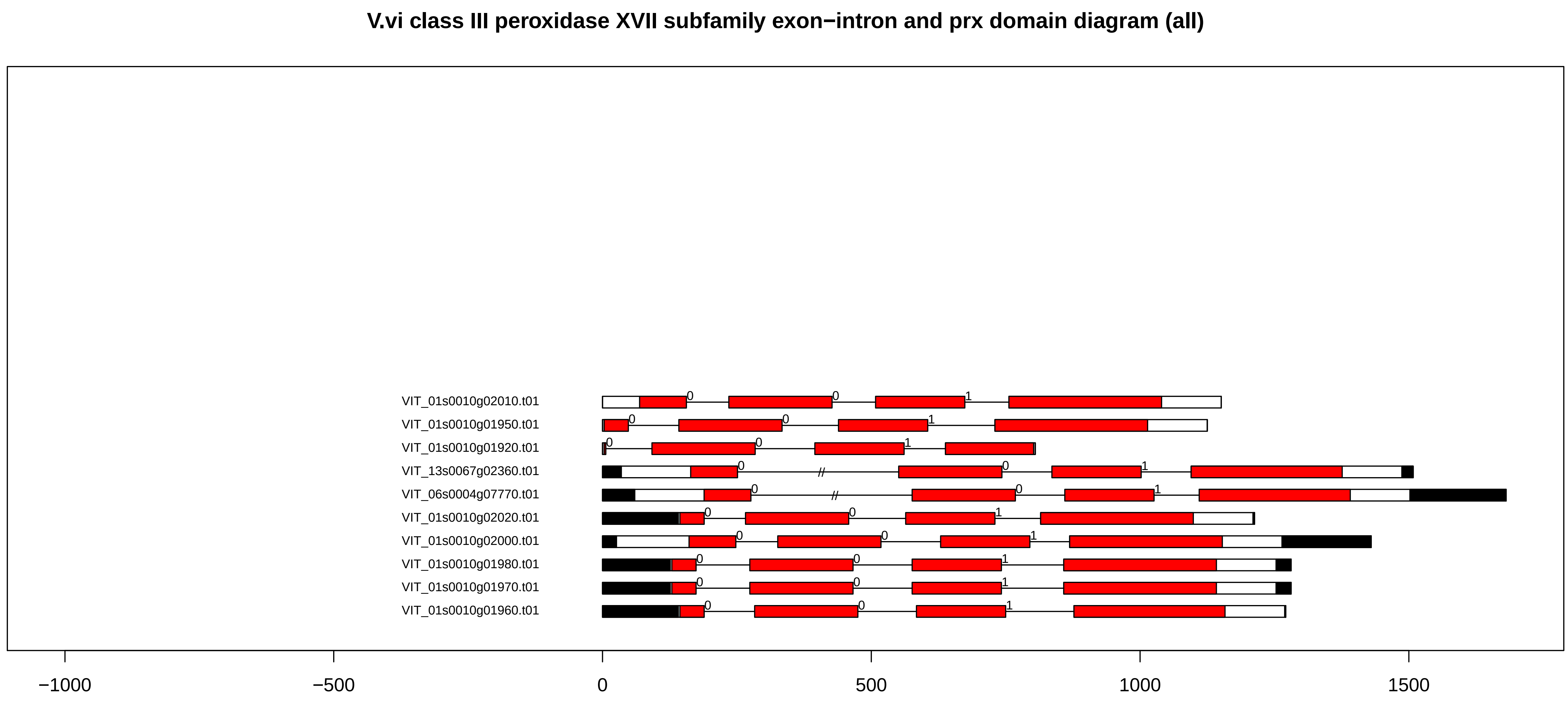

V.vi class III peroxidase XVII subfamily exon−intron and prx domain diagram (all)
VIT_01s0010g02010.t01
VIT_01s0010g01950.t01
VIT_01s0010g01920.t01
VIT_13s0067g02360.t01
VIT_06s0004g07770.t01
VIT_01s0010g02020.t01
VIT_01s0010g02000.t01
VIT_01s0010g01980.t01
VIT_01s0010g01970.t01
VIT_01s0010g01960.t01
−1000
−500
0
500
1000
1500

**V.vi class III peroxidase XVIII subfamily exon−intron and prx domain diagram (all)**

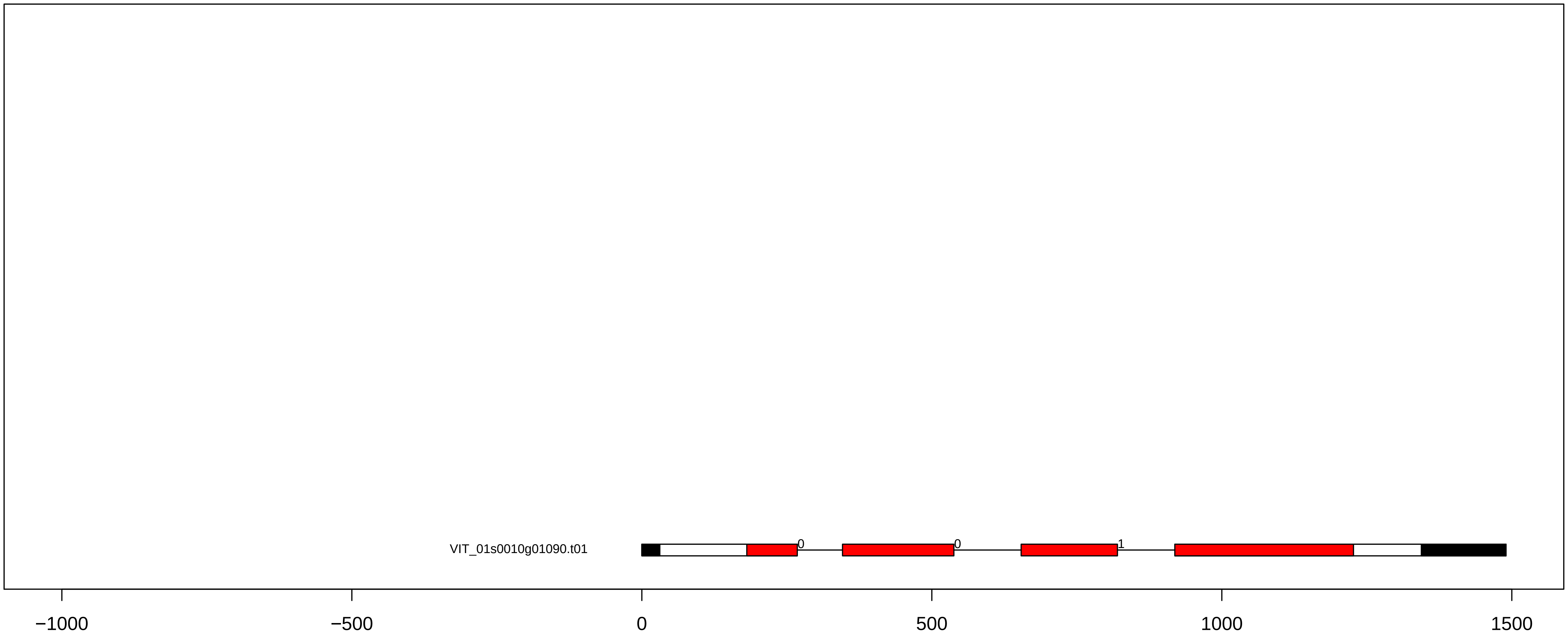

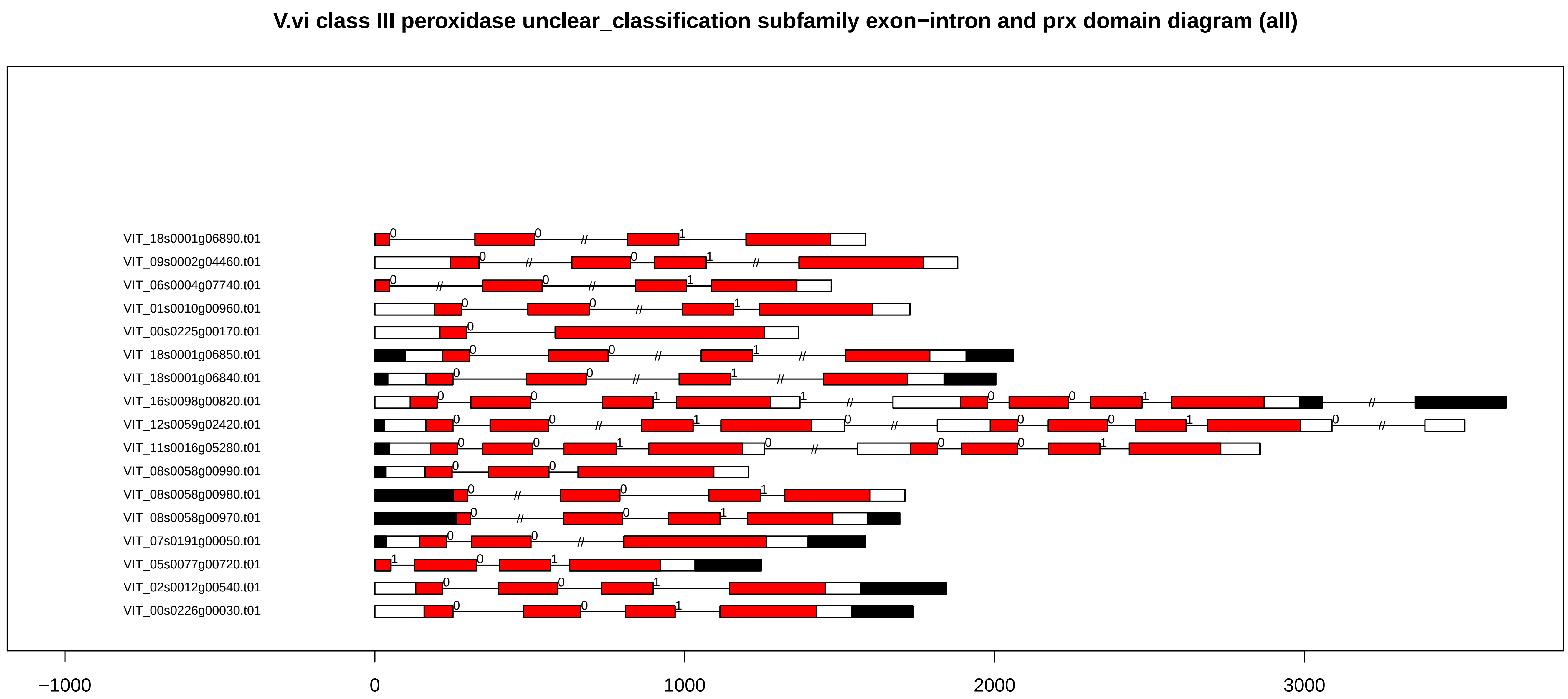
V.vi class III peroxidase unclear_classification subfamily exon−intron and prx domain diagram (all)
VIT_18s0001g06890.t01
VIT_09s0002g04460.t01
VIT_06s0004g07740.t01
VIT_01s0010g00960.t01
VIT_00s0225g00170.t01
VIT_18s0001g06850.t01
VIT_18s0001g06840.t01
VIT_16s0098g00820.t01
VIT_12s0059g02420.t01
VIT_11s0016g05280.t01
VIT_08s0058g00990.t01
VIT_08s0058g00980.t01
VIT_08s0058g00970.t01
VIT_07s0191g00050.t01
VIT_05s0077g00720.t01
VIT_02s0012g00540.t01
VIT_00s0226g00030.t01
−1000
0
1000
2000
3000

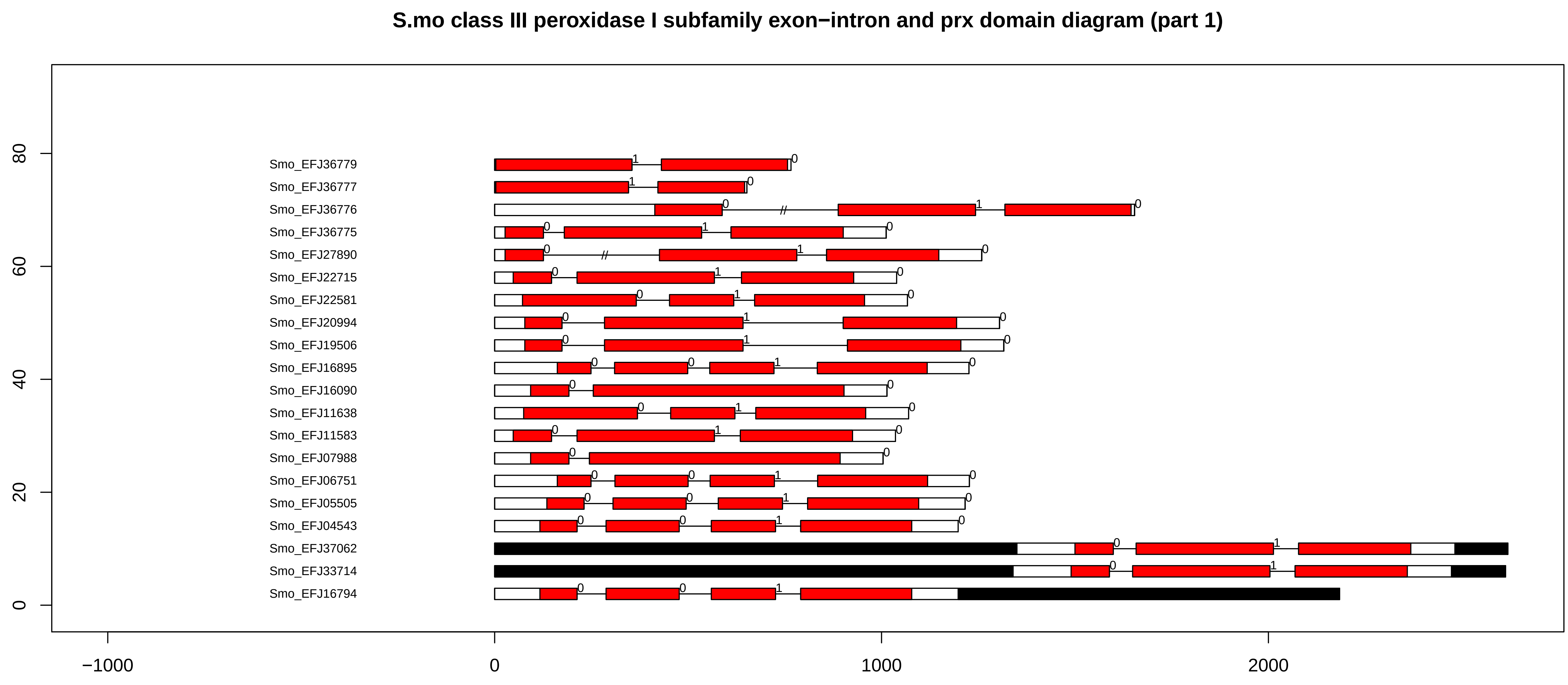
S.mo class III peroxidase I subfamily exon−intron and prx domain diagram (part 1)
Smo_EFJ36779
Smo_EFJ36777
Smo_EFJ36776
Smo_EFJ36775
Smo_EFJ27890
Smo_EFJ22715
Smo_EFJ22581
Smo_EFJ20994
Smo_EFJ19506
Smo_EFJ16895
Smo_EFJ16090
Smo_EFJ11638
Smo_EFJ11583
Smo_EFJ07988
Smo_EFJ06751
Smo_EFJ05505
Smo_EFJ04543
Smo_EFJ37062
Smo_EFJ33714
Smo_EFJ16794
0
20
40
60
80
−1000
0
1000
2000

## S.mo class III peroxidase I subfamily exon−intron and prx domain diagram (part 2)

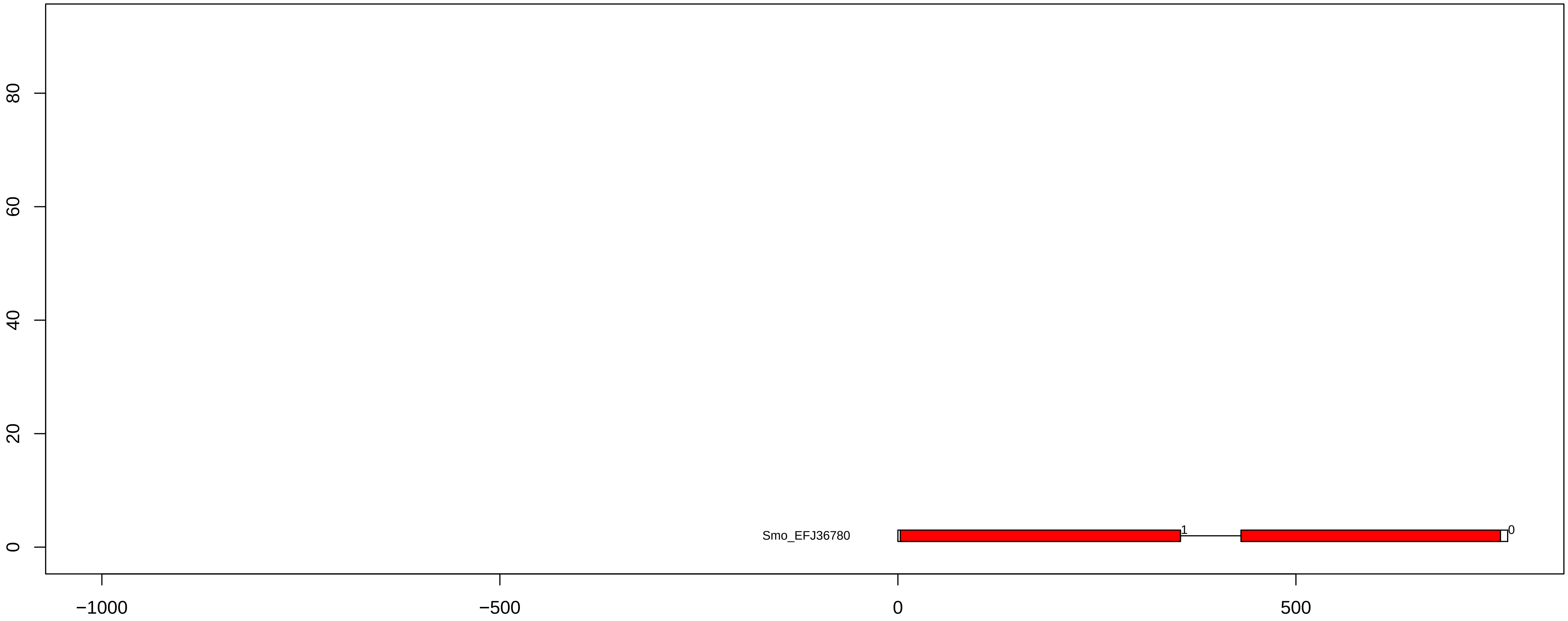

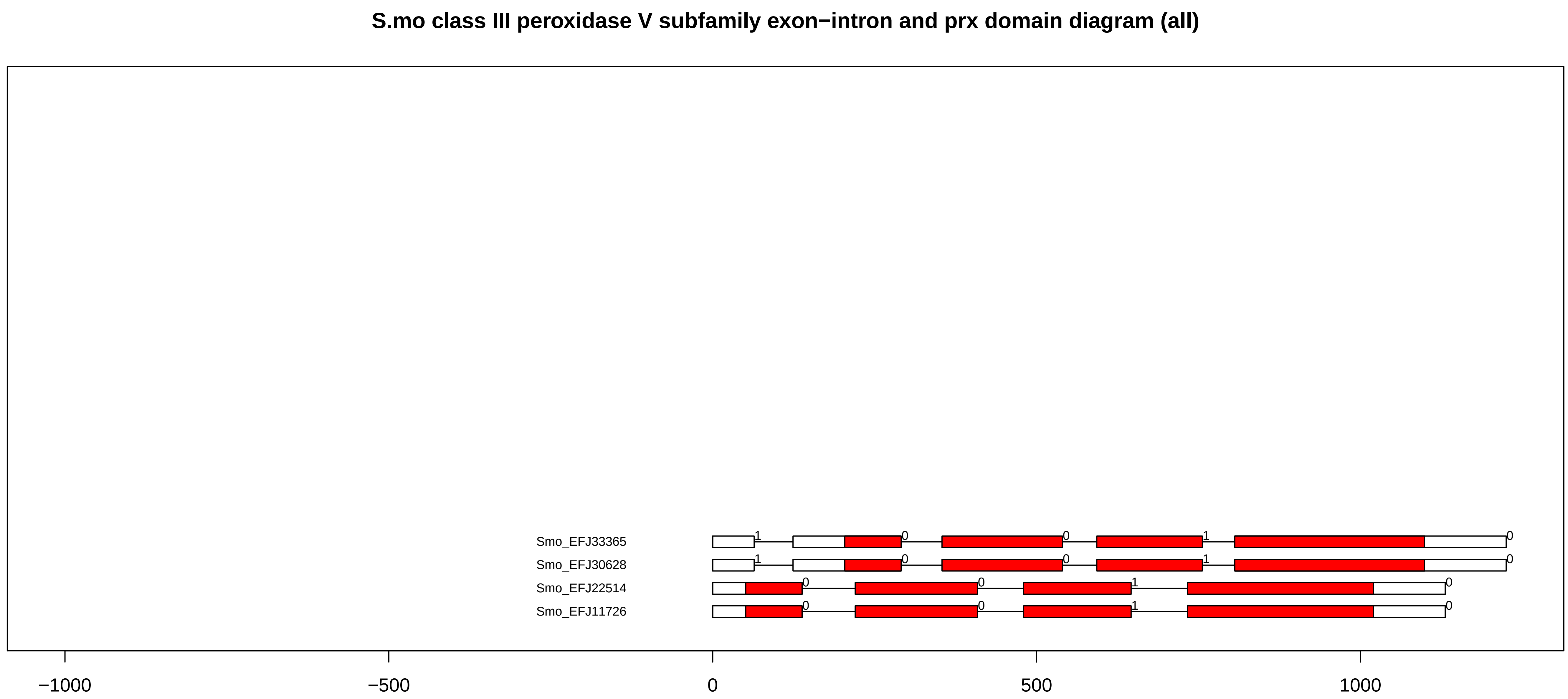
S.mo class III peroxidase V subfamily exon−intron and prx domain diagram (all)
Smo_EFJ33365
Smo_EFJ30628
Smo_EFJ22514
Smo_EFJ11726
1
0
−1000
−500
0
500
1000

## S.mo class III peroxidase VI subfamily exon−intron and prx domain diagram (all)

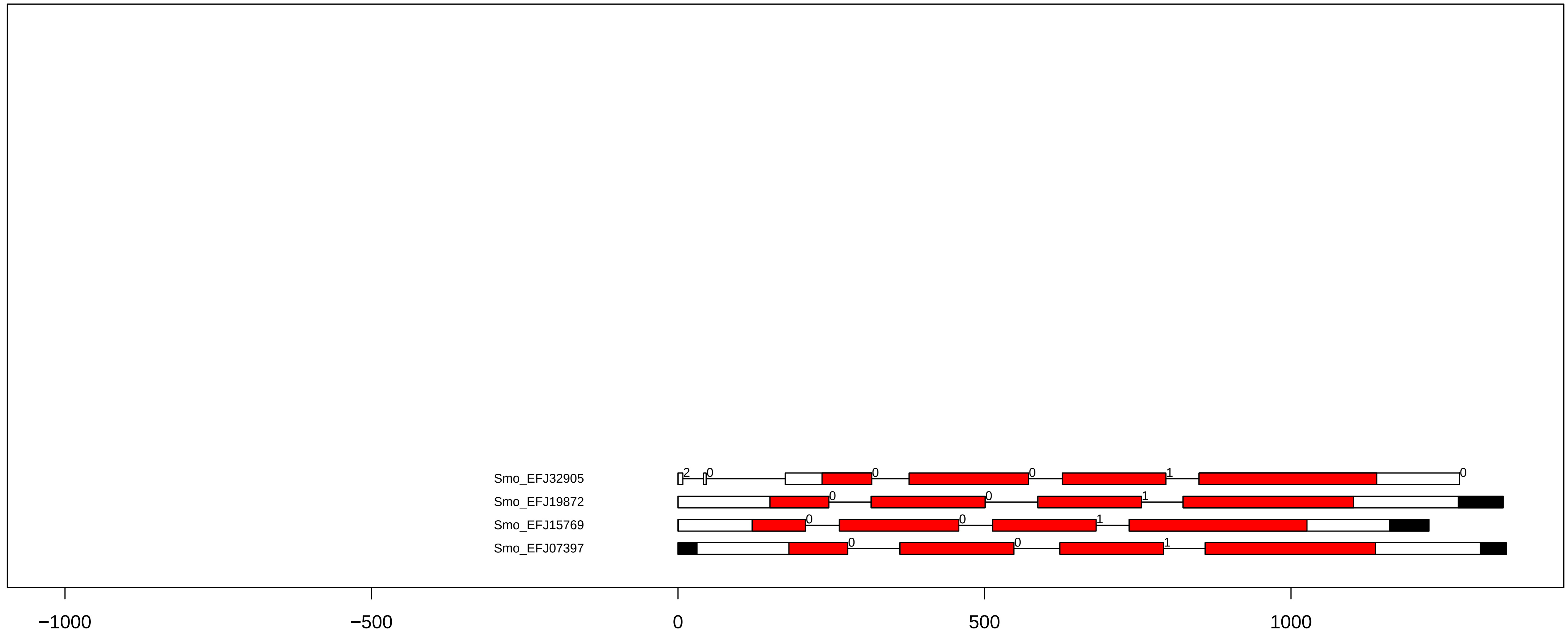

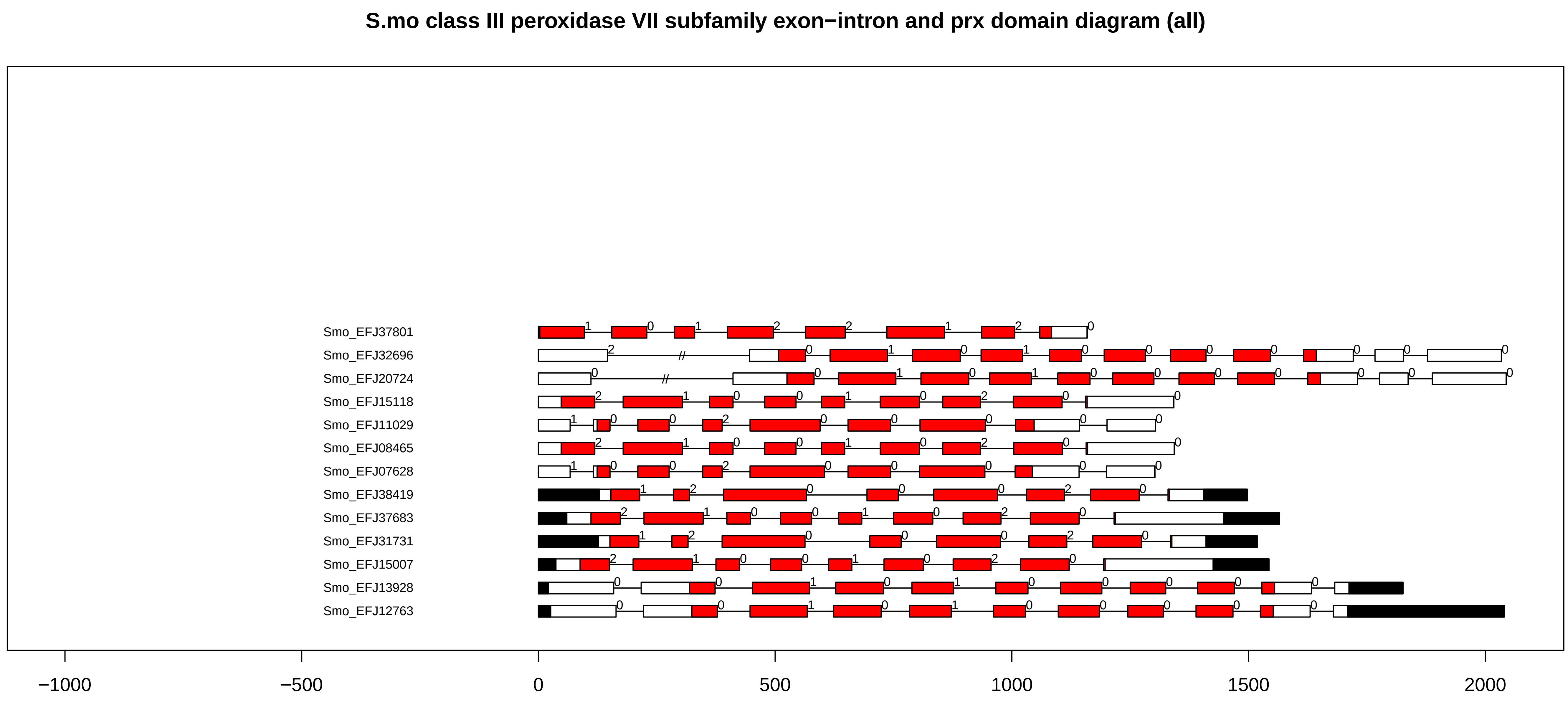
S.mo class III peroxidase VII subfamily exon−intron and prx domain diagram (all)
Smo_EFJ37801
Smo_EFJ32696
Smo_EFJ20724
Smo_EFJ15118
Smo_EFJ11029
Smo_EFJ08465
Smo_EFJ07628
Smo_EFJ38419
Smo_EFJ37683
Smo_EFJ31731
Smo_EFJ15007
Smo_EFJ13928
Smo_EFJ12763
−1000
−500
0
500
1000
1500
2000

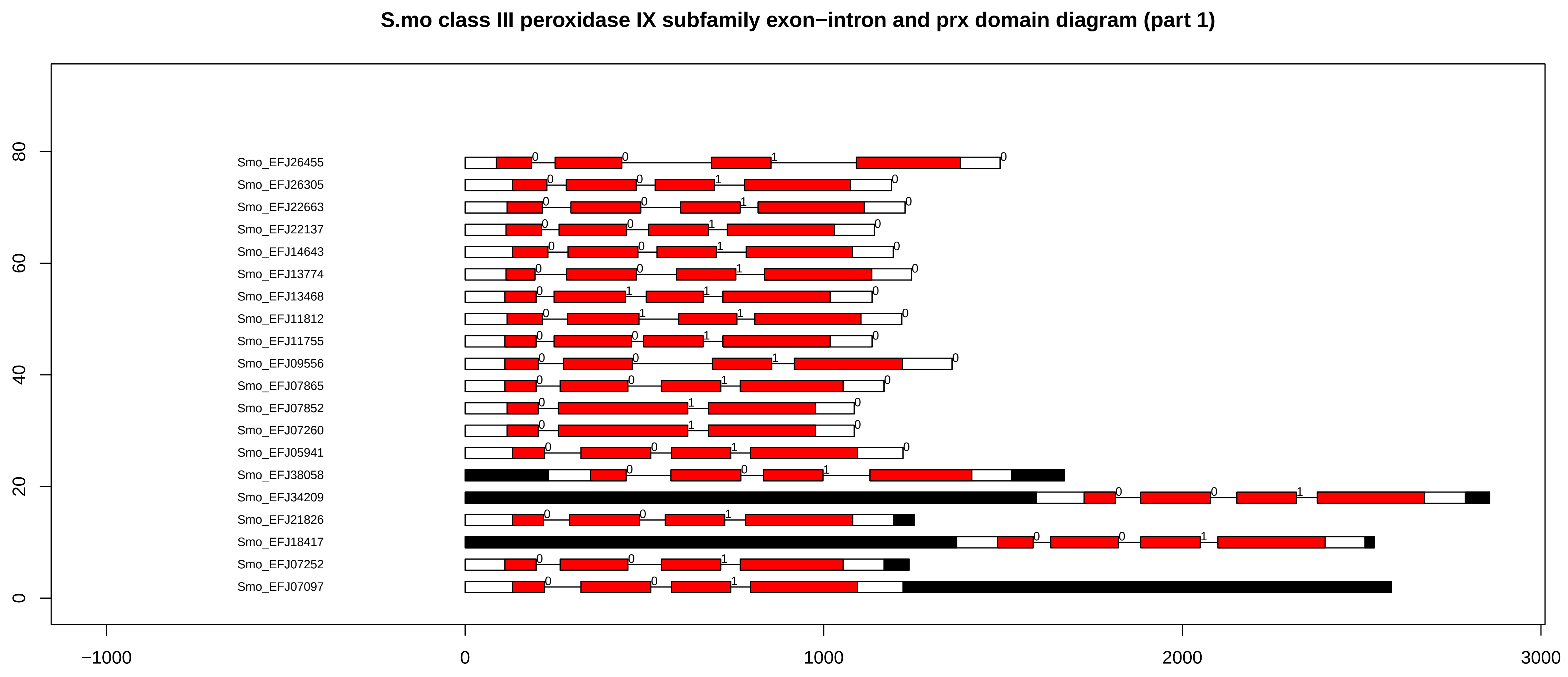
S.mo class III peroxidase IX subfamily exon−intron and prx domain diagram (part 1)
Smo_EFJ26455
Smo_EFJ26305
Smo_EFJ22663
Smo_EFJ22137
Smo_EFJ14643
Smo_EFJ13774
Smo_EFJ13468
Smo_EFJ11812
Smo_EFJ11755
Smo_EFJ09556
Smo_EFJ07865
Smo_EFJ07852
Smo_EFJ07260
Smo_EFJ05941
Smo_EFJ38058
Smo_EFJ34209
Smo_EFJ21826
Smo_EFJ18417
Smo_EFJ07252
Smo_EFJ07097
0
20
40
60
80
−1000
0
1000
2000
3000

## S.mo class III peroxidase IX subfamily exon−intron and prx domain diagram (part 2)

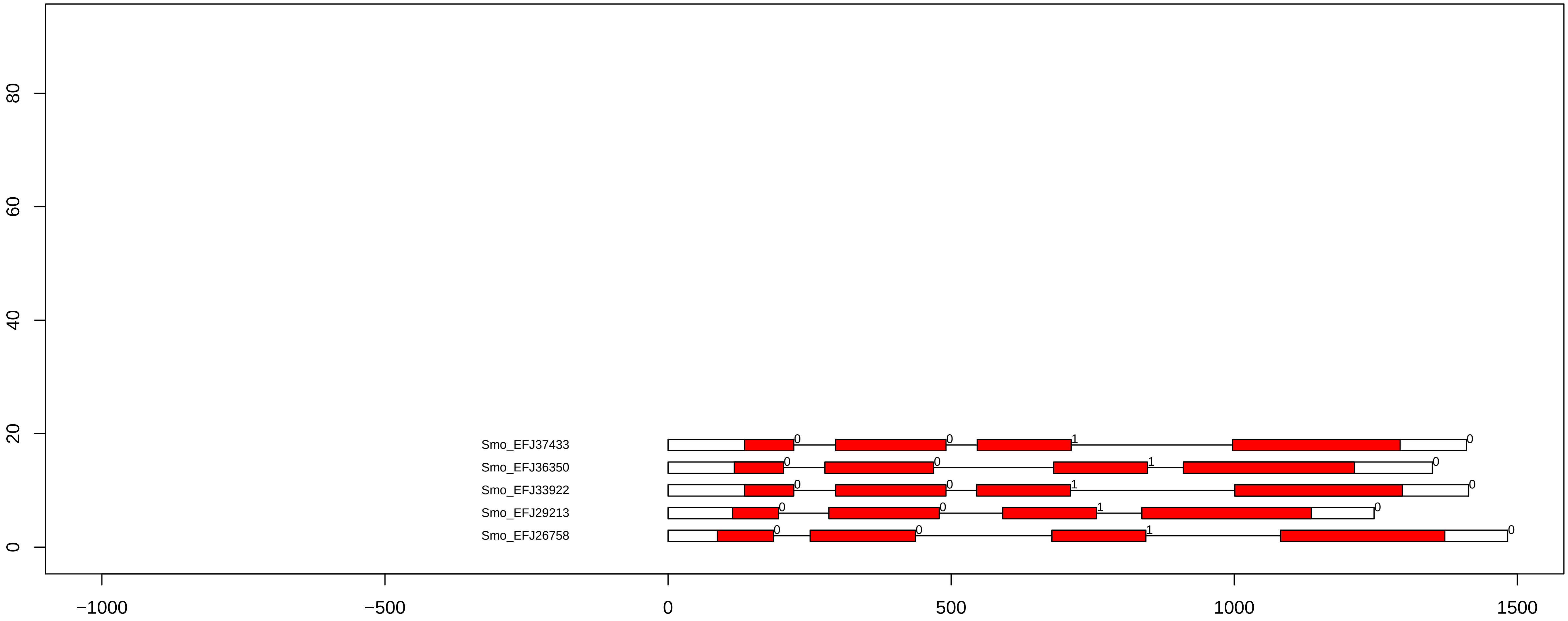

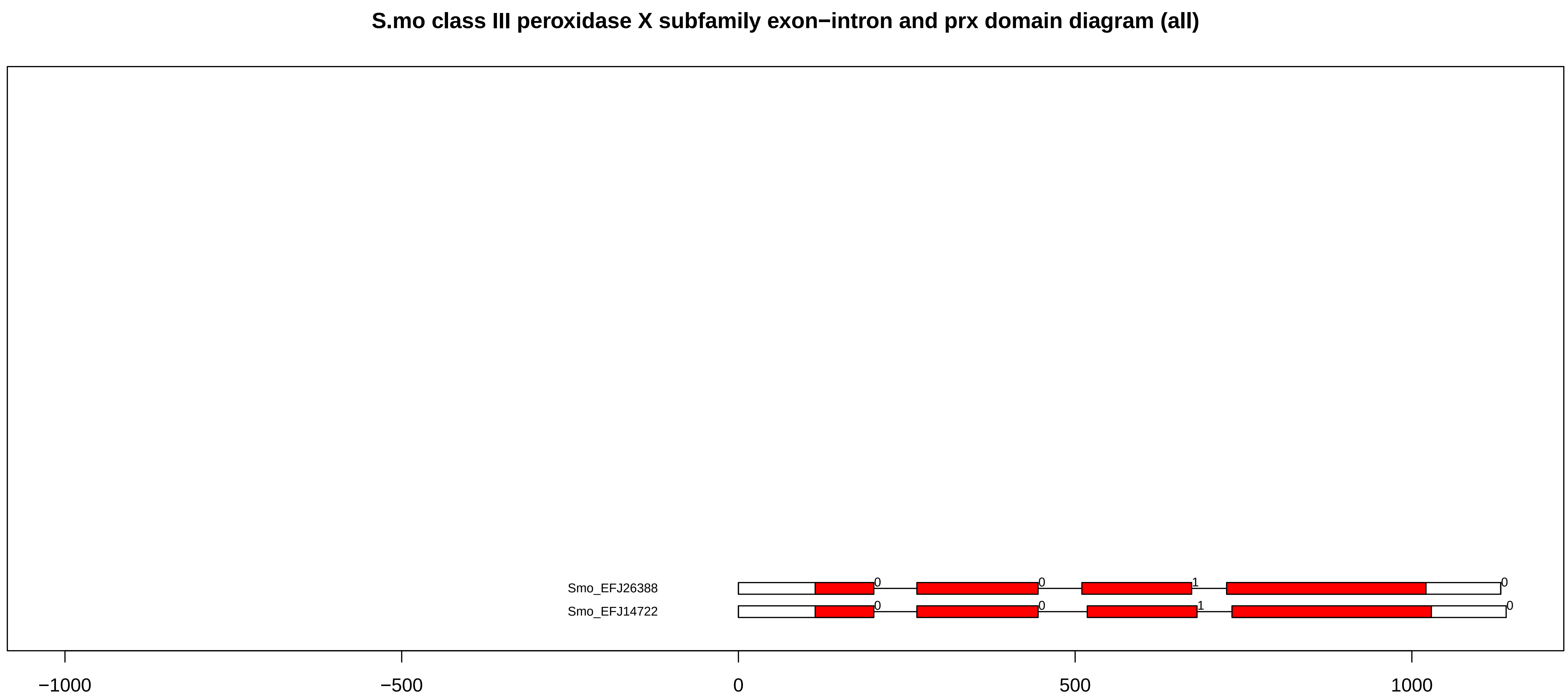
S.mo class III peroxidase X subfamily exon−intron and prx domain diagram (all)
Smo_EFJ26388
Smo_EFJ14722
0
0
1
0
0
0
1
0
−1000
−500
0
500
1000

# S.mo class III peroxidase unclear_classification subfamily exon−intron and prx domain diagram (part 1)

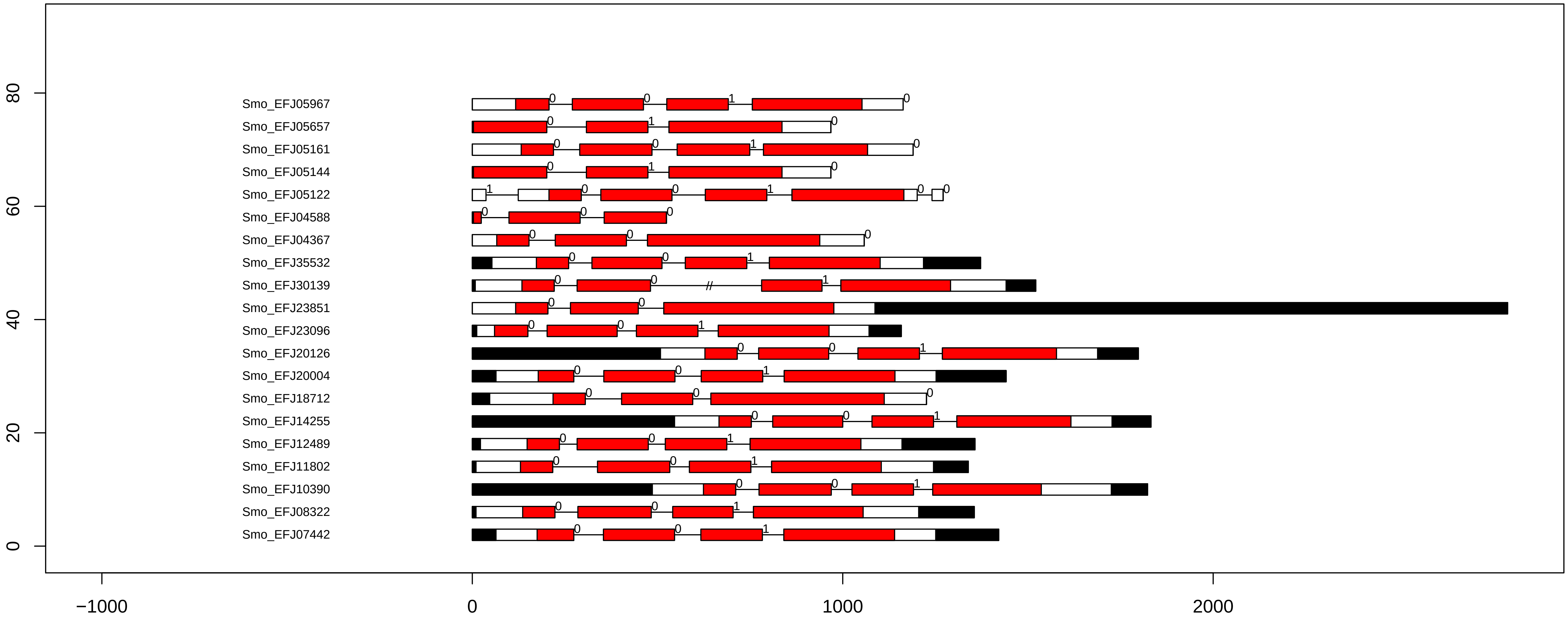

S.mo class III peroxidase unclear_classification subfamily exon−intron and prx domain diagram (part 2)

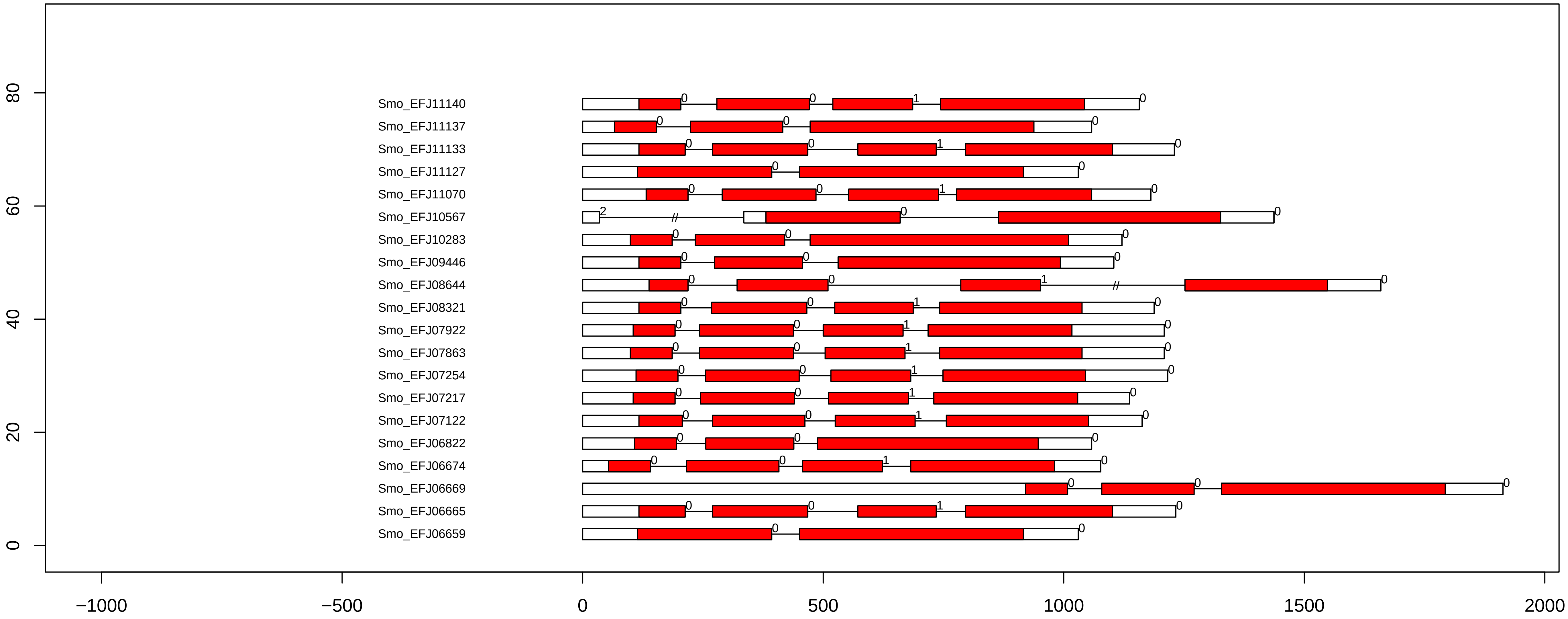

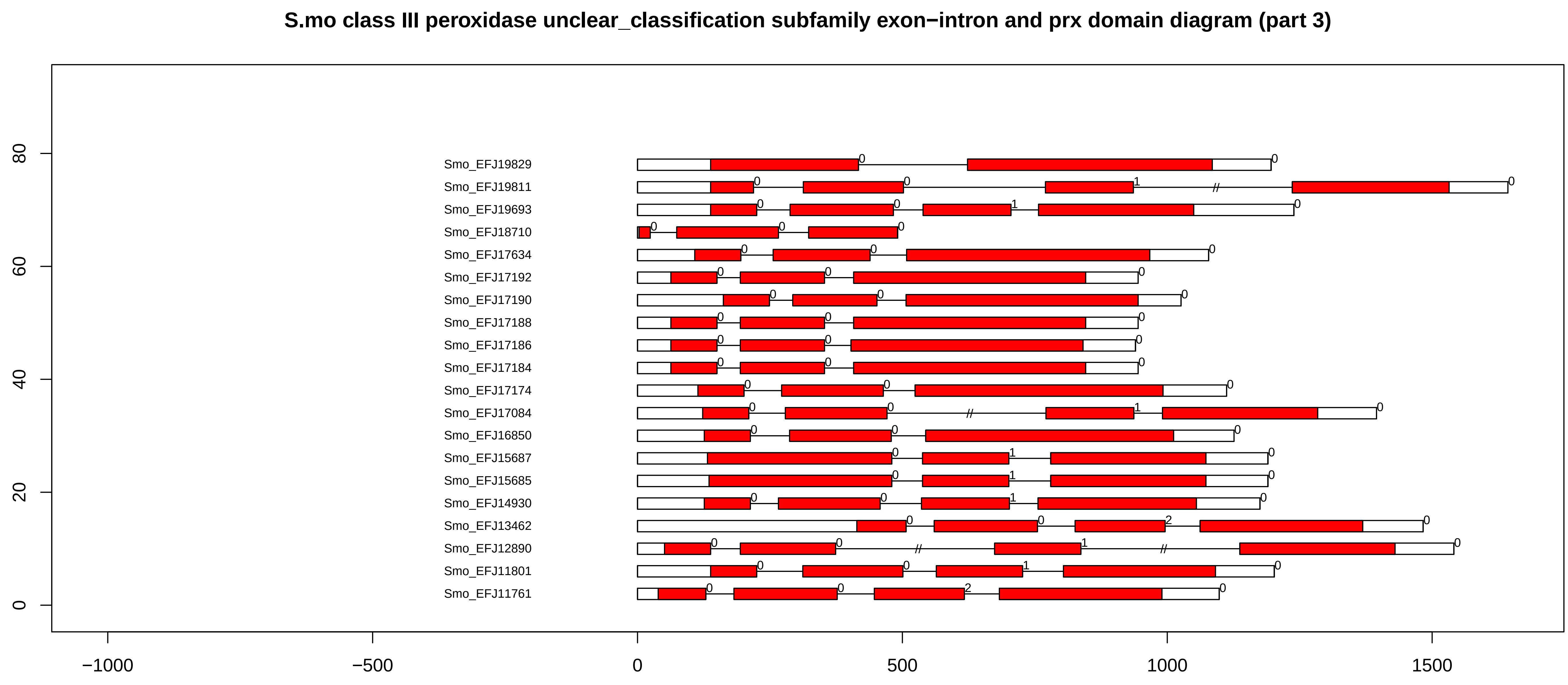
S.mo class III peroxidase unclear_classification subfamily exon−intron and prx domain diagram (part 3)
Smo_EFJ19829
Smo_EFJ19811
Smo_EFJ19693
Smo_EFJ18710
Smo_EFJ17634
Smo_EFJ17192
Smo_EFJ17190
Smo_EFJ17188
Smo_EFJ17186
Smo_EFJ17184
Smo_EFJ17174
Smo_EFJ17084
Smo_EFJ16850
Smo_EFJ15687
Smo_EFJ15685
Smo_EFJ14930
Smo_EFJ13462
Smo_EFJ12890
Smo_EFJ11801
Smo_EFJ11761
0
20
40
60
80
−1000
−500
0
500
1000
1500

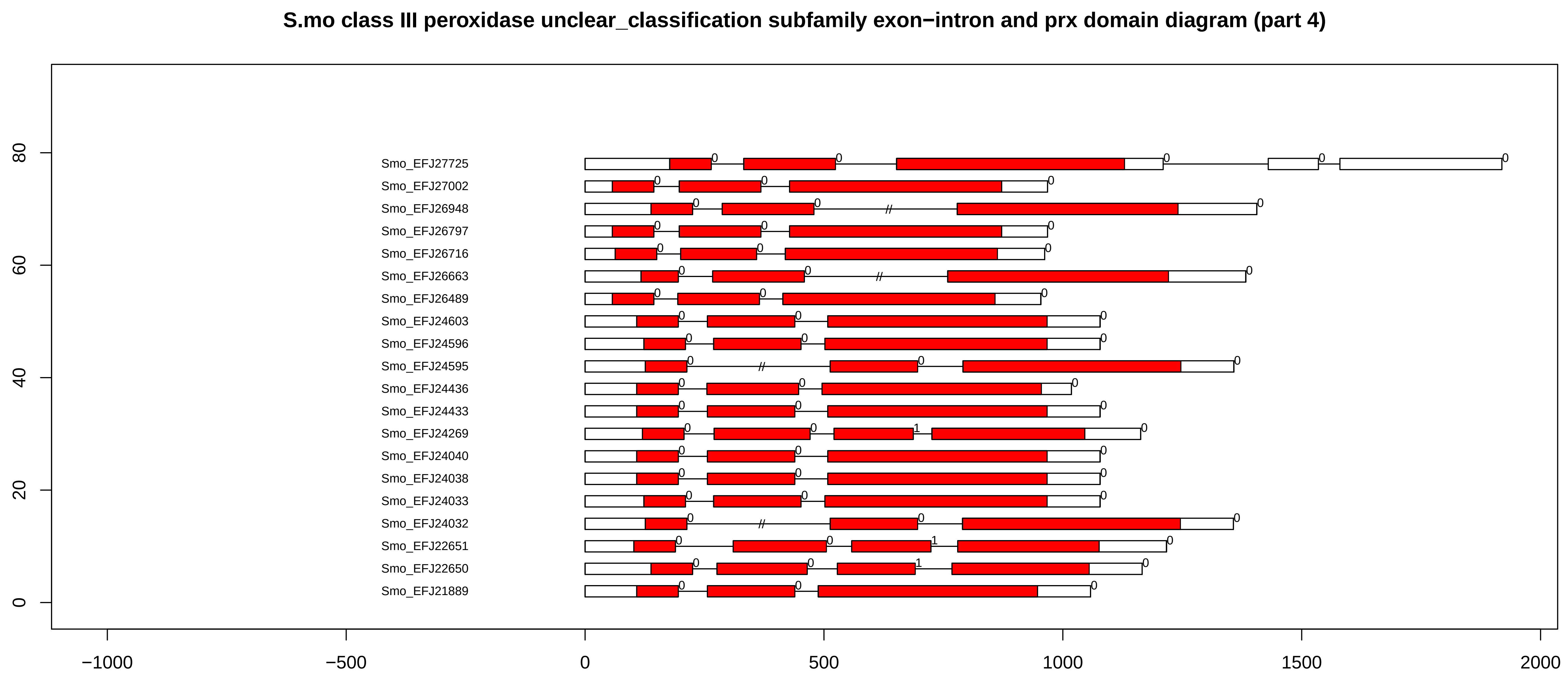
S.mo class III peroxidase unclear_classification subfamily exon−intron and prx domain diagram (part 4)
Smo_EFJ27725
Smo_EFJ27002
Smo_EFJ26948
Smo_EFJ26797
Smo_EFJ26716
Smo_EFJ26663
Smo_EFJ26489
Smo_EFJ24603
Smo_EFJ24596
Smo_EFJ24595
Smo_EFJ24436
Smo_EFJ24433
Smo_EFJ24269
Smo_EFJ24040
Smo_EFJ24038
Smo_EFJ24033
Smo_EFJ24032
Smo_EFJ22651
Smo_EFJ22650
Smo_EFJ21889
0
20
40
60
80
−1000
−500
0
500
1000
1500
2000

S.mo class III peroxidase unclear_classification subfamily exon−intron and prx domain diagram (part 5)

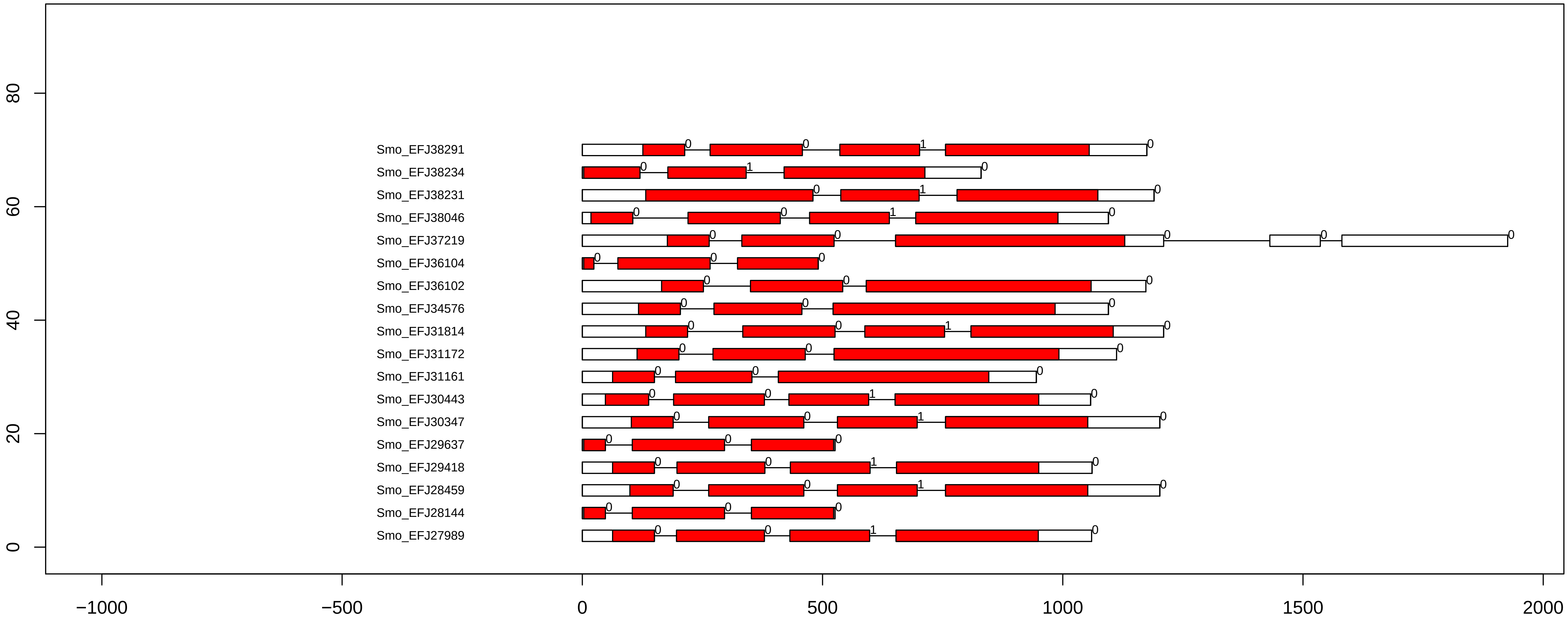

# P.pa class III peroxidase I subfamily exon−intron and prx domain diagram (part 1)

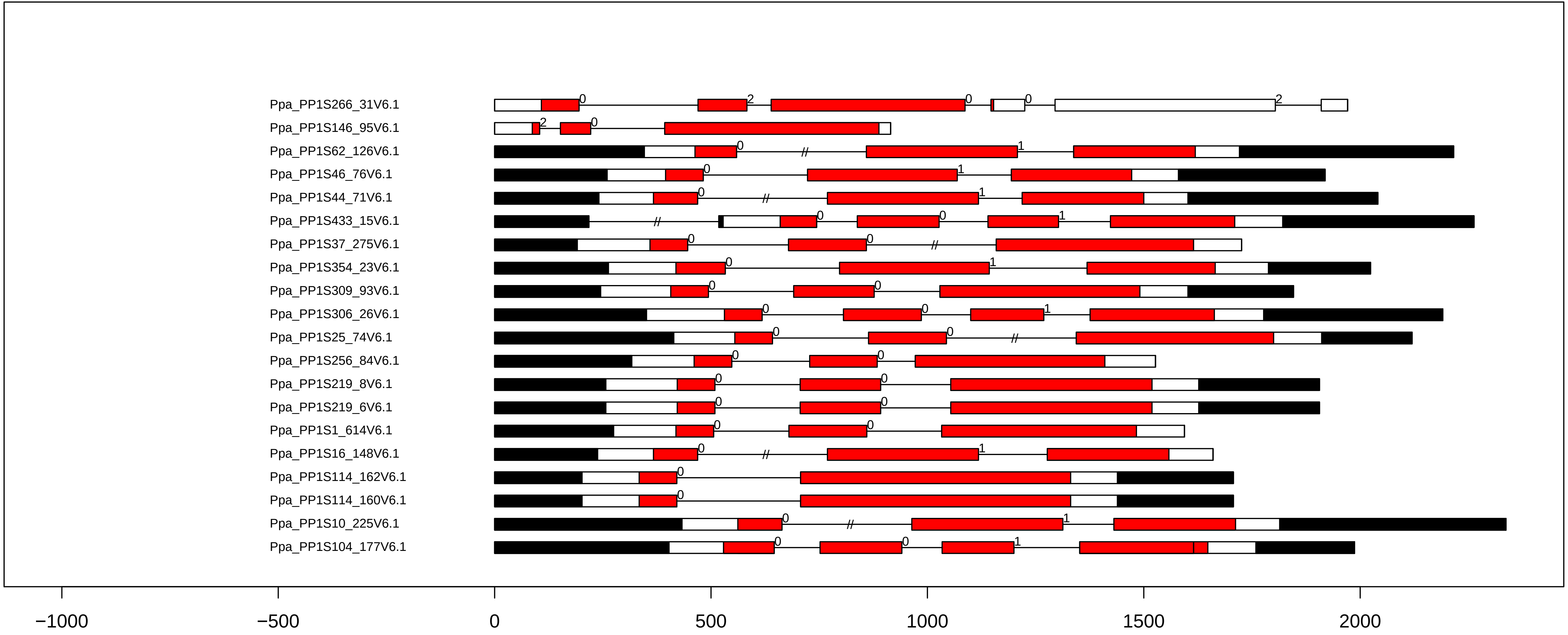

# P.pa class III peroxidase I subfamily exon−intron and prx domain diagram (part 2)

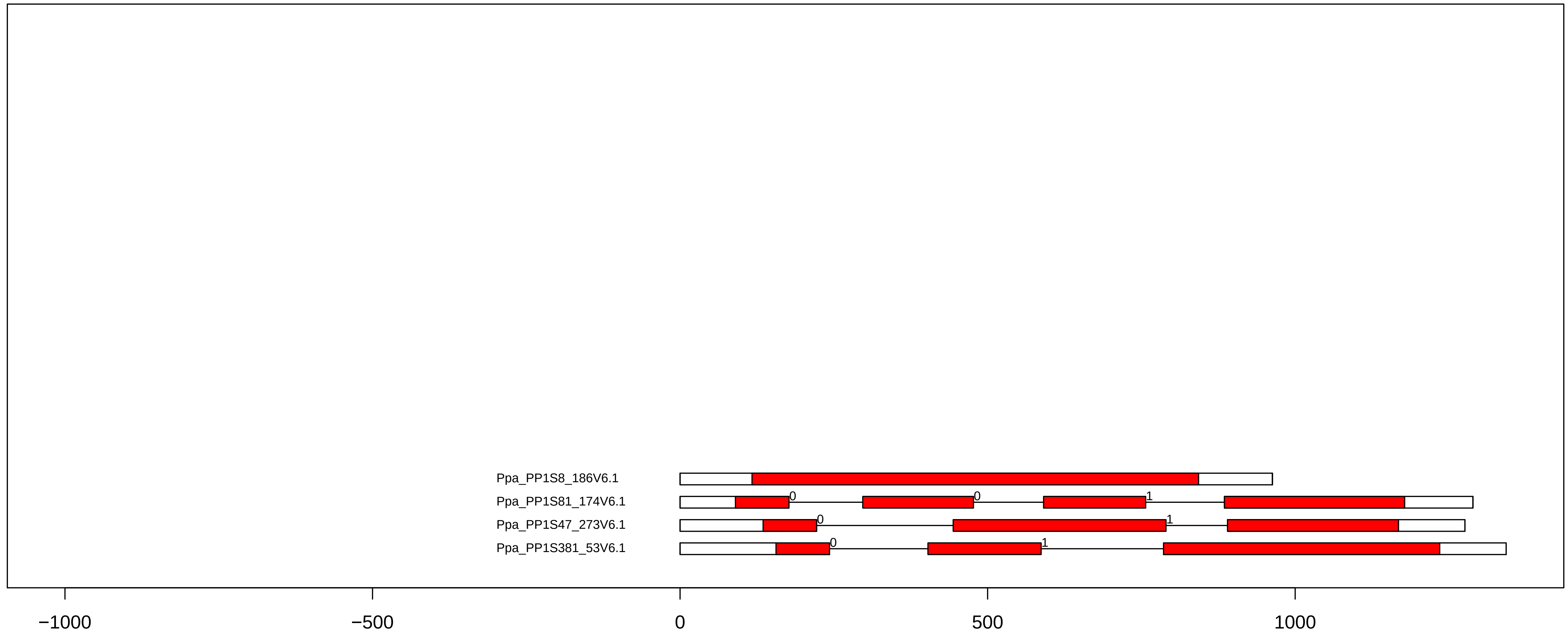

# P.pa class III peroxidase VII subfamily exon−intron and prx domain diagram (all)

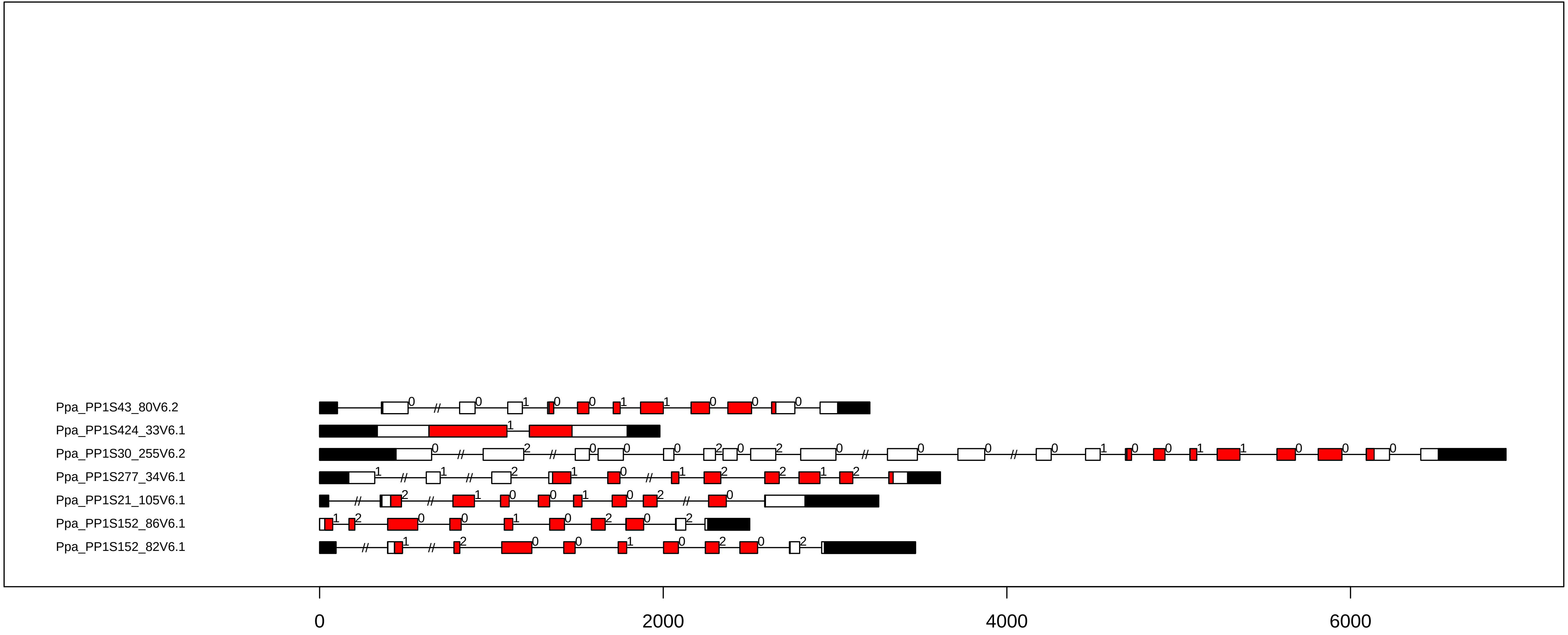

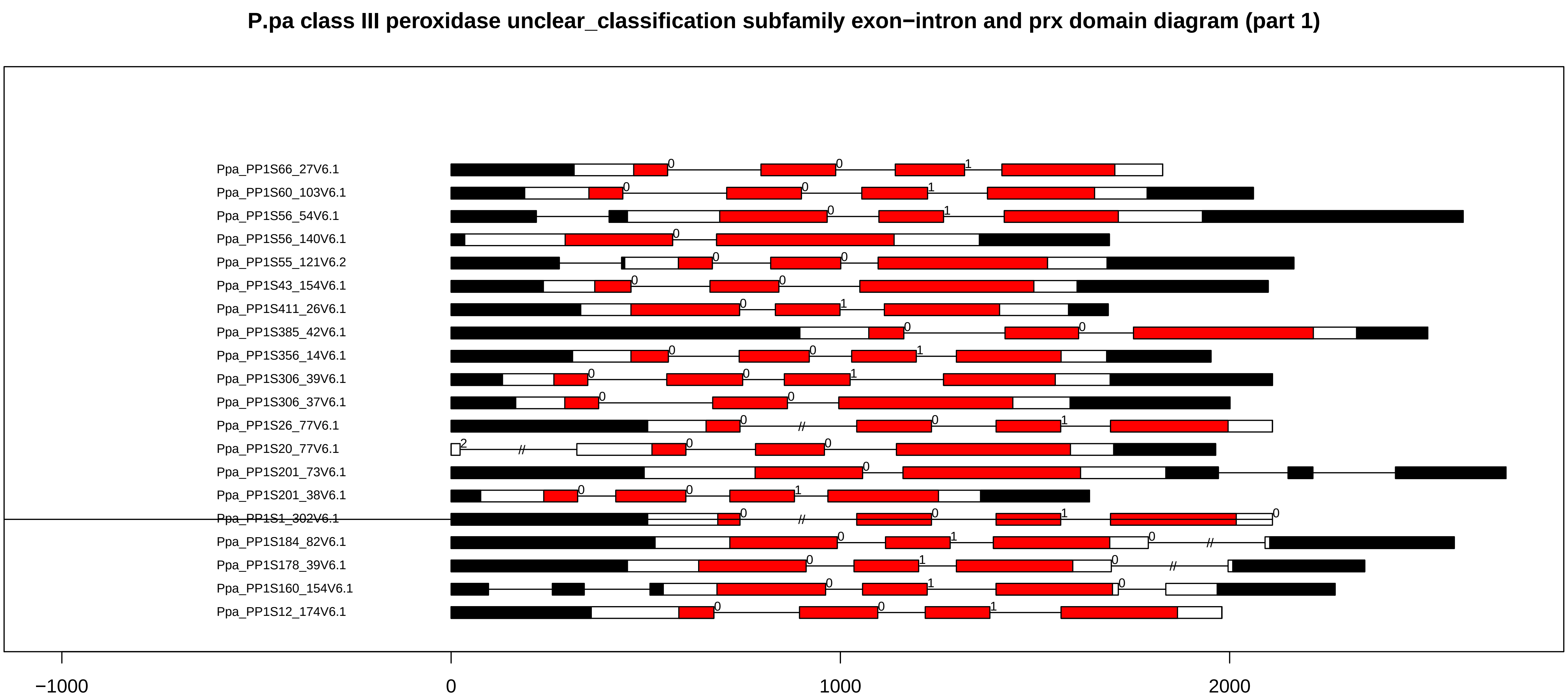
P.pa class III peroxidase unclear_classification subfamily exon−intron and prx domain diagram (part 1)
Ppa_PP1S66_27V6.1
Ppa_PP1S60_103V6.1
Ppa_PP1S56_54V6.1
Ppa_PP1S56_140V6.1
Ppa_PP1S55_121V6.2
Ppa_PP1S43_154V6.1
Ppa_PP1S411_26V6.1
Ppa_PP1S385_42V6.1
Ppa_PP1S356_14V6.1
Ppa_PP1S306_39V6.1
Ppa_PP1S306_37V6.1
Ppa_PP1S26_77V6.1
Ppa_PP1S20_77V6.1
Ppa_PP1S201_73V6.1
Ppa_PP1S201_38V6.1
Ppa_PP1S1_302V6.1
Ppa_PP1S184_82V6.1
Ppa_PP1S178_39V6.1
Ppa_PP1S160_154V6.1
Ppa_PP1S12_174V6.1
−1000
0
1000
2000

# P.pa class III peroxidase unclear_classification subfamily exon−intron and prx domain diagram (part 2)

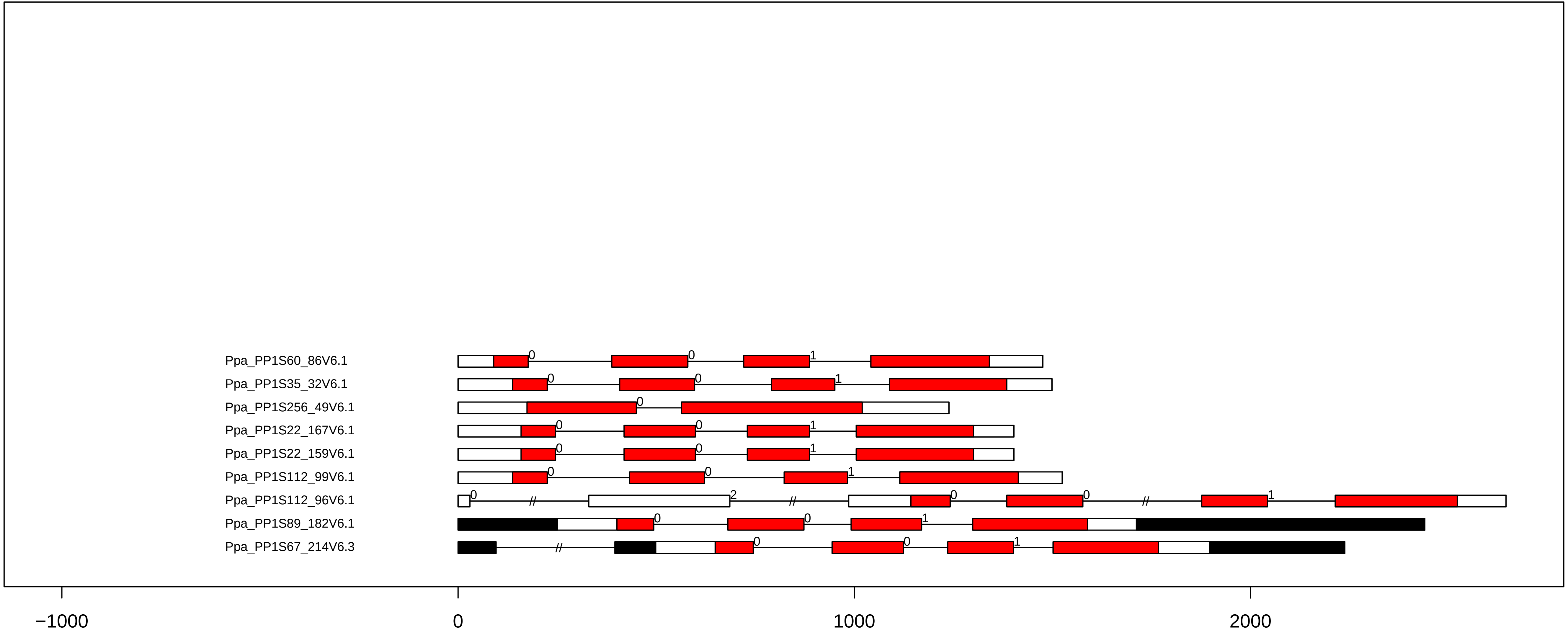

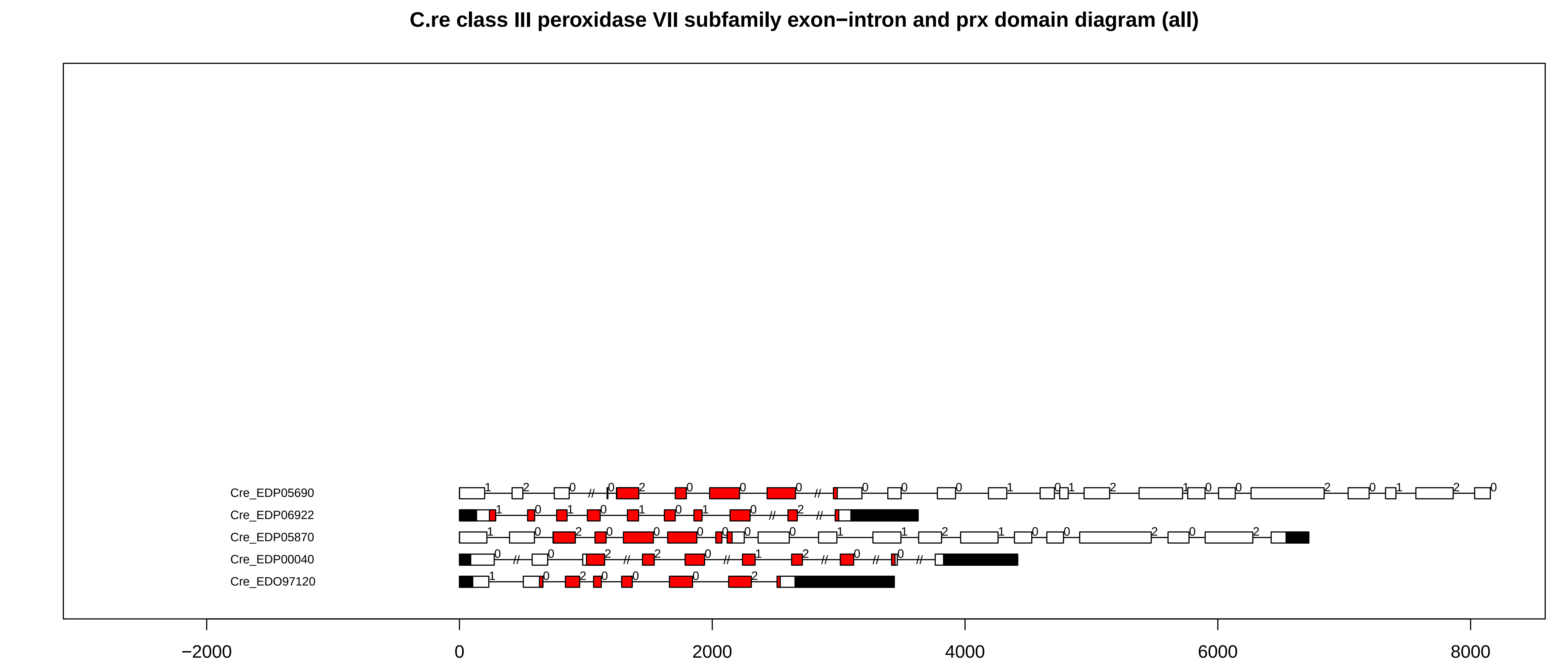
C.re class III peroxidase VII subfamily exon−intron and prx domain diagram (all)
Cre_EDP05690
Cre_EDP06922
Cre_EDP05870
Cre_EDP00040
Cre_EDO97120
−2000
0
2000
4000
6000
8000

## C.re class III peroxidase unclear_classification subfamily exon−intron and prx domain diagram (all)

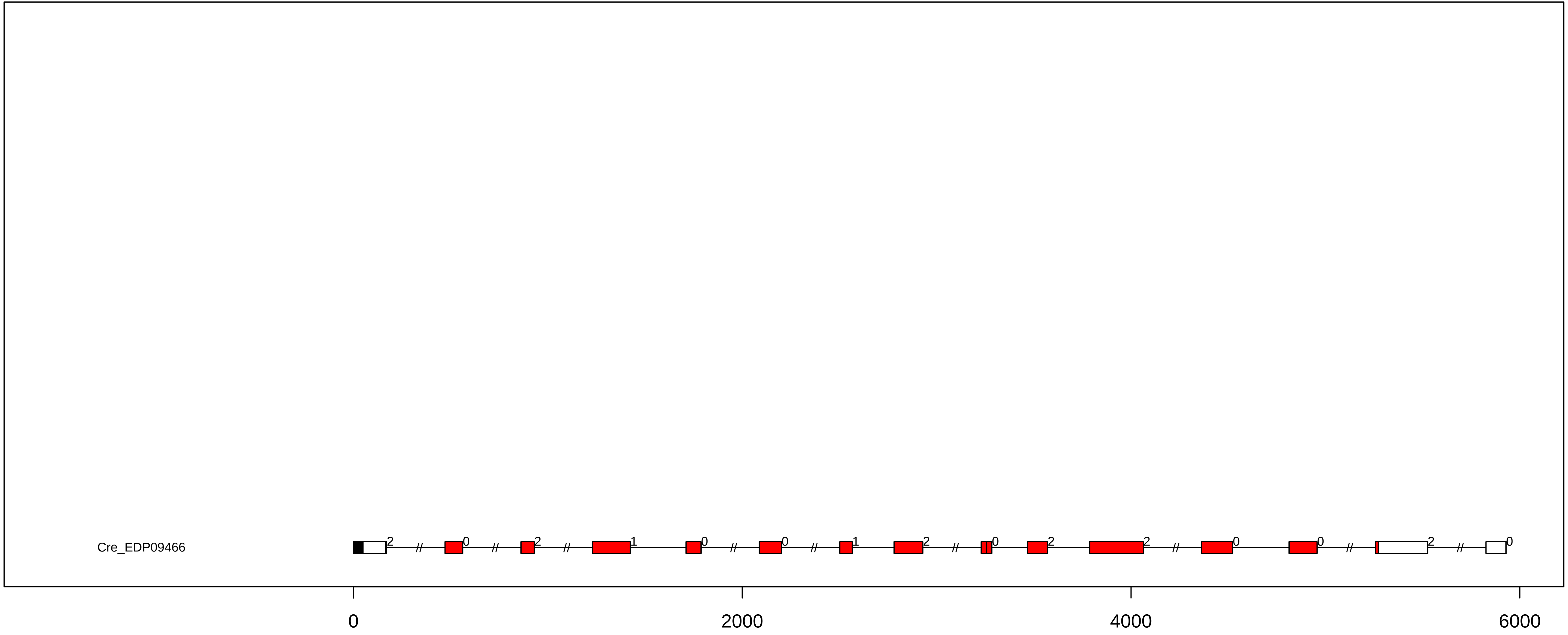

Supplement: Supplementary file 2 — Figure S2. Exon−intron and domain diagrams of class III peroxidases in T. aestivum, T. urartu, Ae. tauschii, B. distachyon, Z. mays, O. sativa, A. thaliana, V. vinifera, S. moellendorffii, P. patens and C. reinhardtii. The descriptions of the domain and exon phases are the same as those in Fig. 2. The lengths of the boxes and lines are scaled based on the lengths of the genes. (PDF 487 kb) [file 12864_2019_6006_MOESM2_ESM.pdf]
