# Supplementary material for: Genome-wide and evolutionary analysis of the class III peroxidase gene family in wheat and Aegilops tauschii reveals that some members are involved in stress responses
Source: BMC Genomics. 2019 Aug 22;20:666. doi: 10.1186/s12864-019-6006-5 (PMC6704529; doi:10.1186/s12864-019-6006-5)
Supplement: Supplementary file 3 — Figure S3. Conserved exon−intron and domain diagrams of class III peroxidases in T. aestivum, B. distachyon, S. moellendorffii and P. patens. The descriptions of the domain and exon phases are the same as those in Fig. 2. The lengths of the boxes and lines are scaled based on the lengths of the genes. (PDF 30 kb) [file 12864_2019_6006_MOESM3_ESM.pdf]

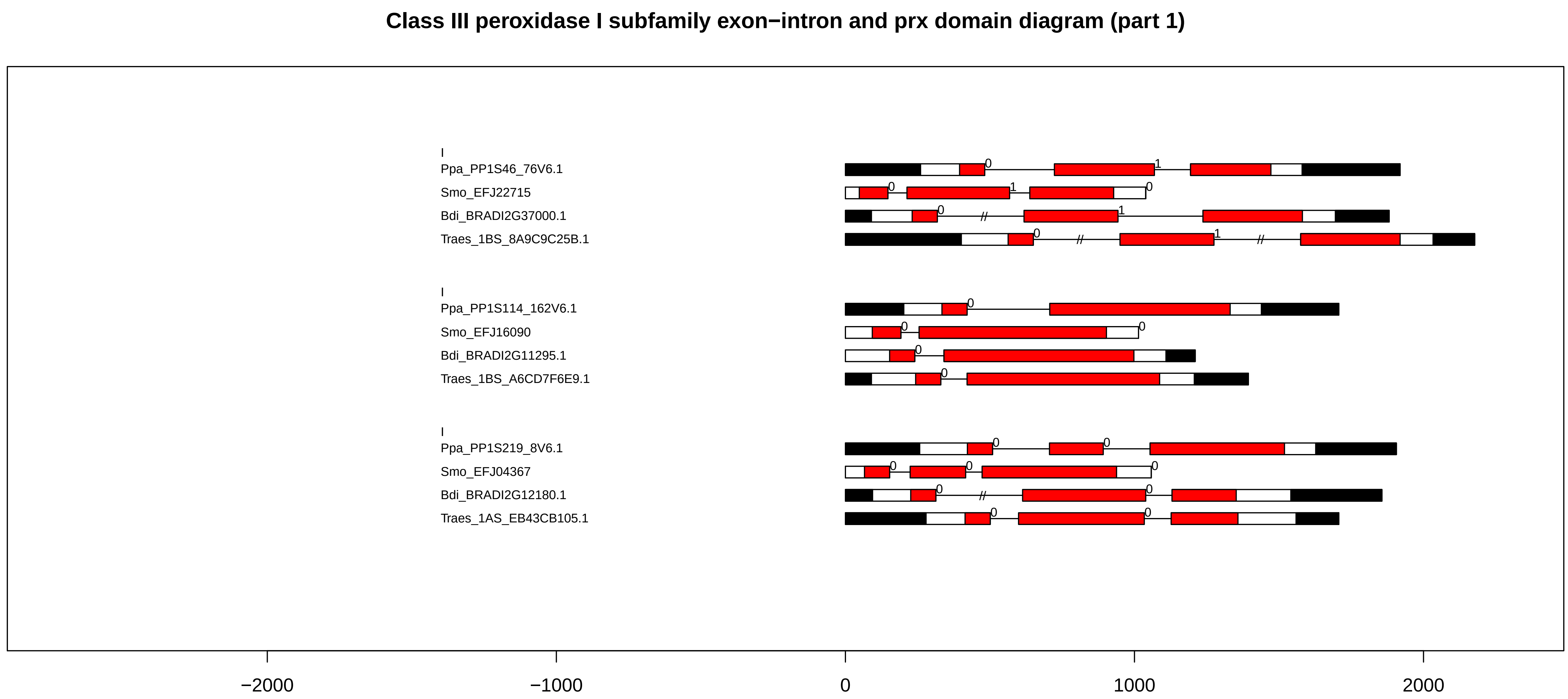
Class III peroxidase I subfamily exon−intron and prx domain diagram (part 1)
I
Ppa_PP1S46_76V6.1
Smo_EFJ22715
Bdi_BRADI2G37000.1
Traes_1BS_8A9C9C25B.1
I
Ppa_PP1S114_162V6.1
Smo_EFJ16090
Bdi_BRADI2G11295.1
Traes_1BS_A6CD7F6E9.1
I
Ppa_PP1S219_8V6.1
Smo_EFJ04367
Bdi_BRADI2G12180.1
Traes_1AS_EB43CB105.1
−2000
−1000
0
1000
2000

Class III peroxidase I subfamily exon−intron and prx domain diagram (part 2)

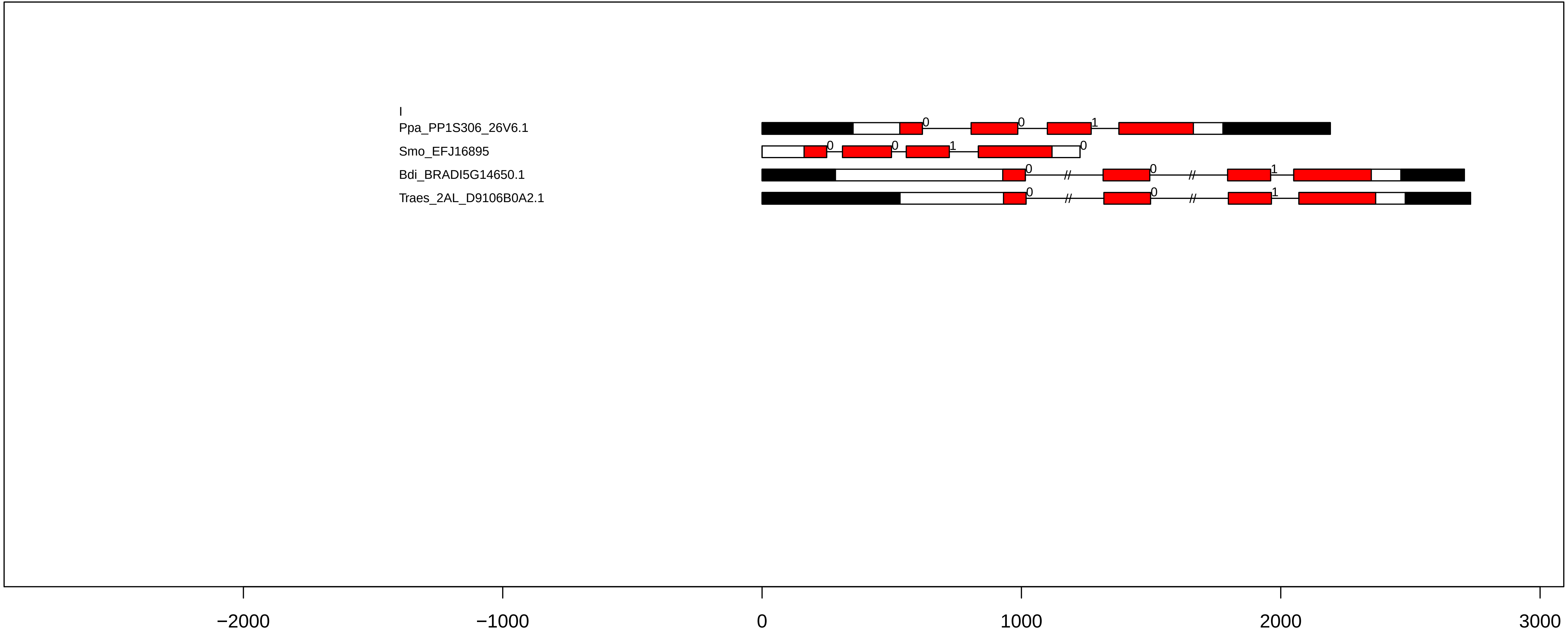

Class III peroxidase VII subfamily exon−intron and prx domain diagram (all)

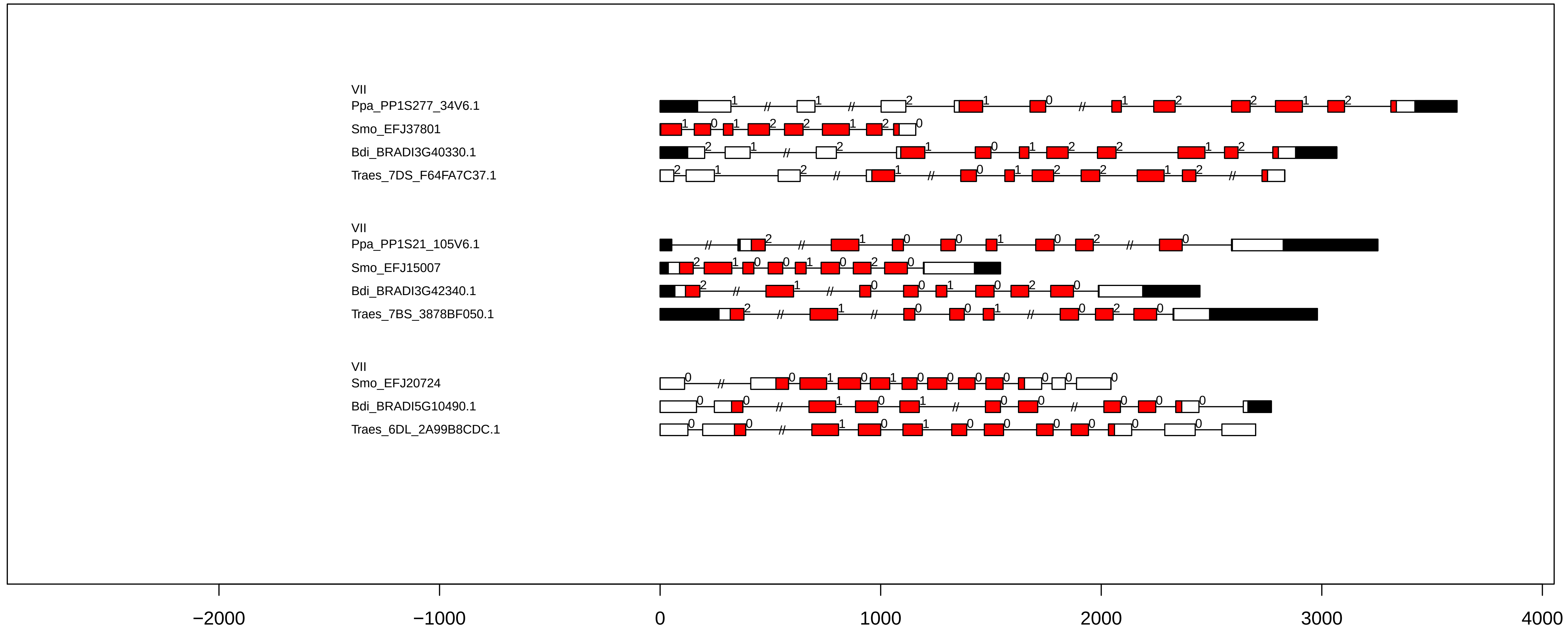

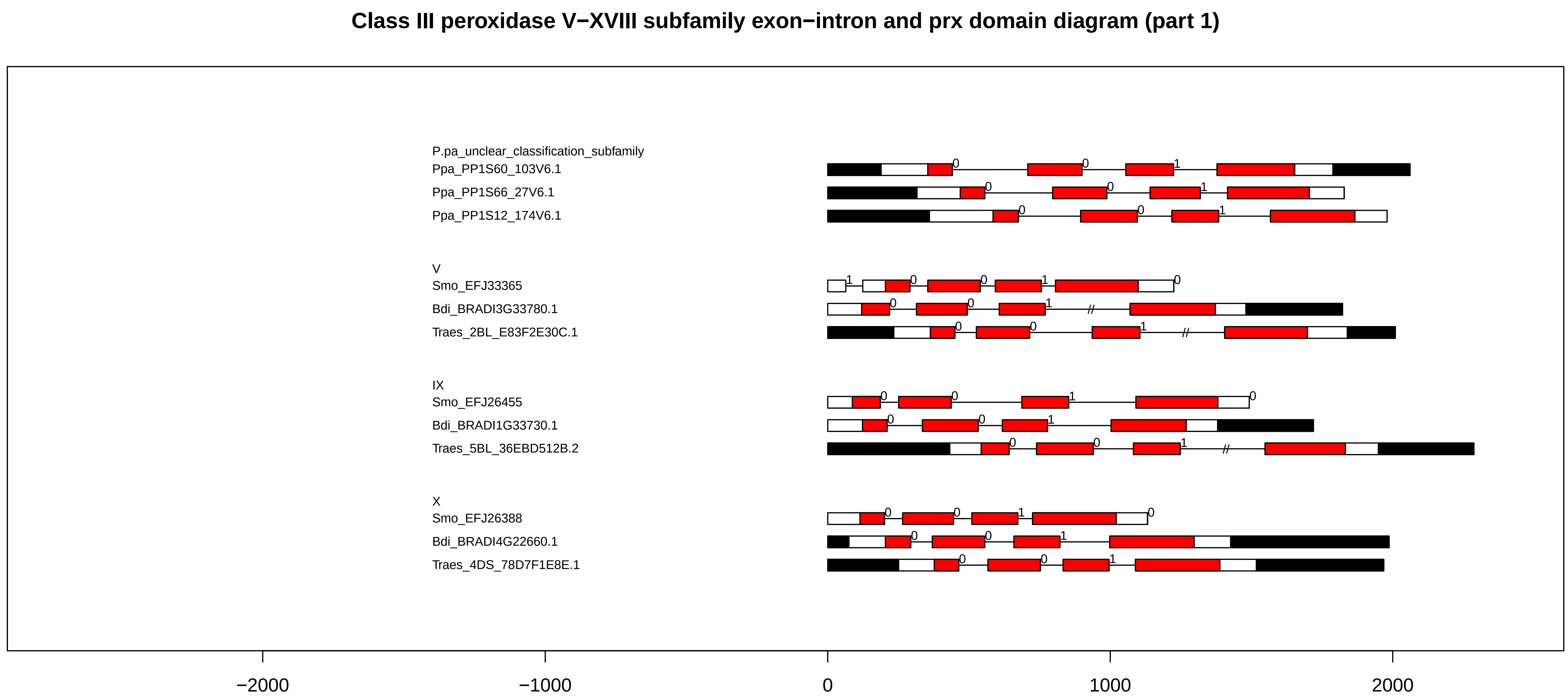
Class III peroxidase V−XVIII subfamily exon−intron and prx domain diagram (part 1)
P.pa_unclear_classification_subfamily
Ppa_PP1S60_103V6.1
Ppa_PP1S66_27V6.1
Ppa_PP1S12_174V6.1
V
Smo_EFJ33365
Bdi_BRADI3G33780.1
Traes_2BL_E83F2E30C.1
IX
Smo_EFJ26455
Bdi_BRADI1G33730.1
Traes_5BL_36EBD512B.2
X
Smo_EFJ26388
Bdi_BRADI4G22660.1
Traes_4DS_78D7F1E8E.1
−2000
−1000
0
1000
2000

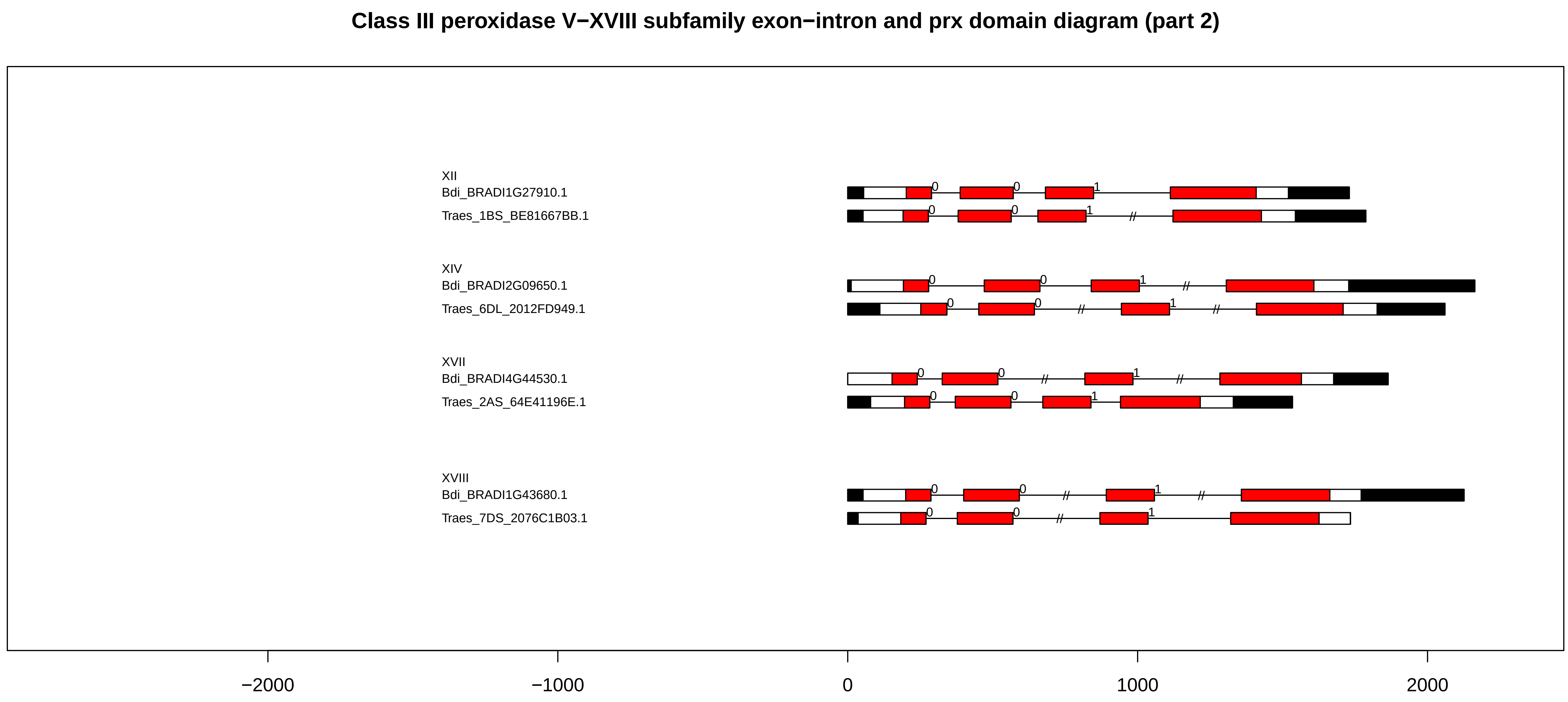

Class III peroxidase V−XVIII subfamily exon−intron and prx domain diagram (part 2)
XII
Bdi_BRADI1G27910.1
Traes_1BS_BE81667BB.1
XIV
Bdi_BRADI2G09650.1
Traes_6DL_2012FD949.1
XVII
Bdi_BRADI4G44530.1
Traes_2AS_64E41196E.1
XVIII
Bdi_BRADI1G43680.1
Traes_7DS_2076C1B03.1
−2000
−1000
0
1000
2000
