# Supplementary figures and images for: Genome-wide and evolutionary analysis of the class III peroxidase gene family in wheat and Aegilops tauschii reveals that some members are involved in stress responses
Source: BMC Genomics. 2019 Aug 22;20:666. doi: 10.1186/s12864-019-6006-5 (PMC6704529; doi:10.1186/s12864-019-6006-5)

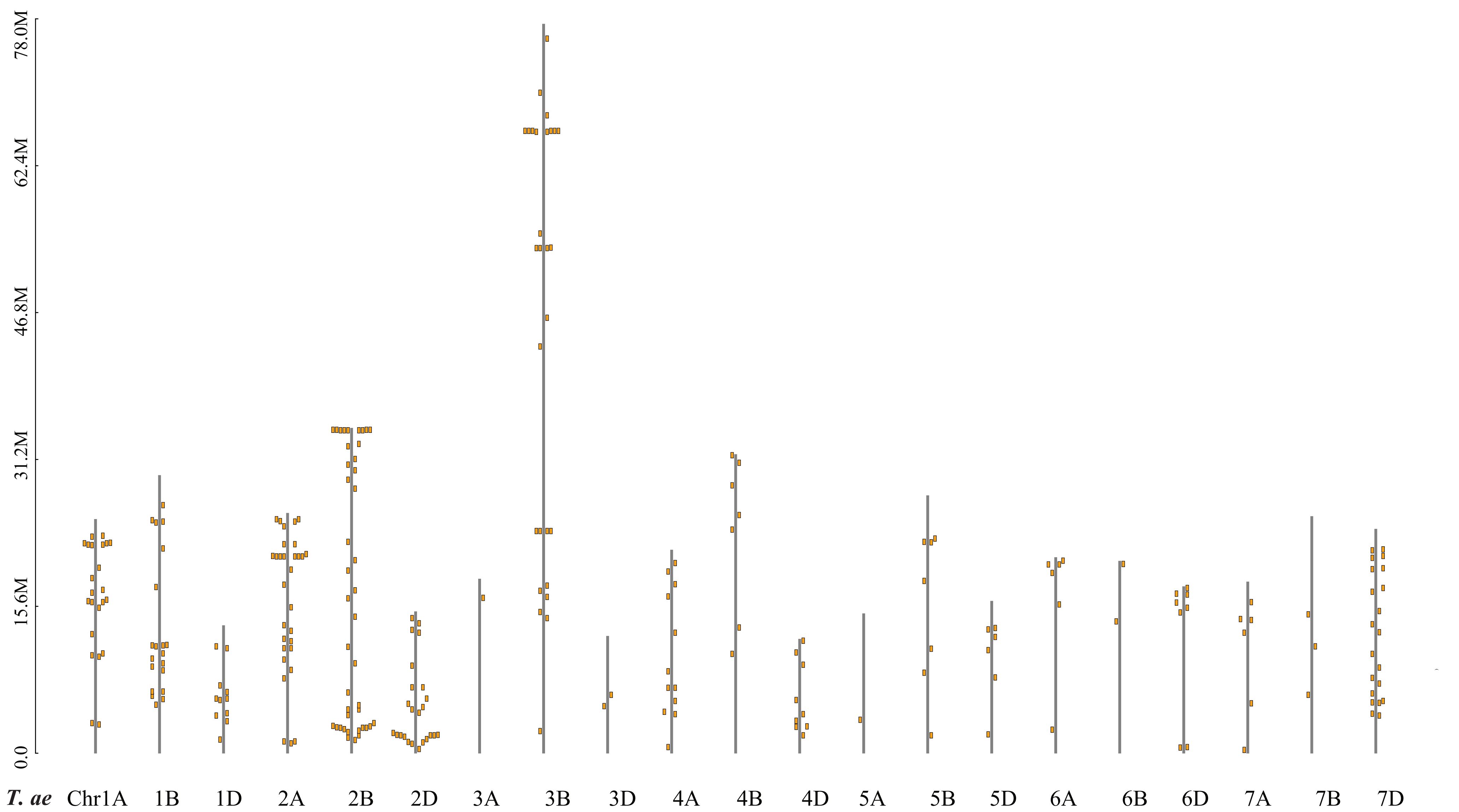

Supplement: Supplementary file 4 — Figure S4. Chromosome locations of class III peroxidases in T. aestivum. (PDF 49 kb) [file 12864_2019_6006_MOESM4_ESM.pdf]

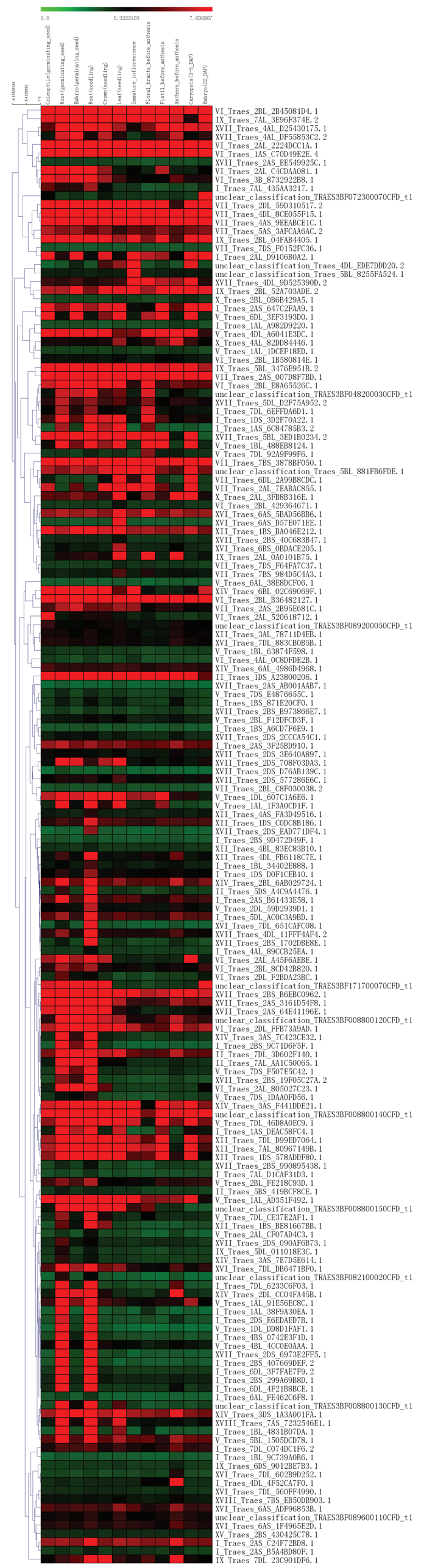

Supplement: Supplementary file 6 — Figure S6. Heatmap of the expression patterns of T. aestivum class III peroxidase genes in different tissues. The expression patterns of 170 class III peroxidase genes in different tissues: coleoptile, root and embryo of germinating seed; root, crown and leaf of seedling; immature inflorescence; floral bracts, pistil and anthers before anthesis; 3-5 DAP (day after planting) caryopsis; 22 DAP embryos. The heatmap was generated using MeV (Multiple Experiment Viewer) software, version 4.9. Red and green correspond to upregulation and downregulation, respectively. Normalized gene expression values and p values are provided in Additional file 20: Table S8. (PDF 1350 kb) [file 12864_2019_6006_MOESM6_ESM.pdf]

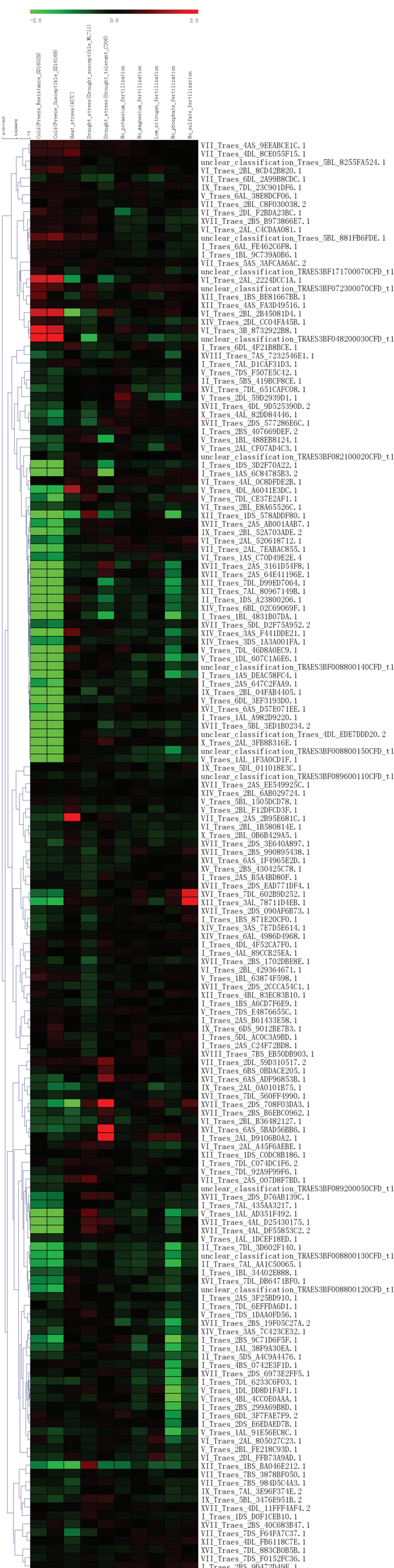

Supplement: Supplementary file 7 — Figure S7. Heatmap of the expression patterns of T. aestivum class III peroxidase genes under abiotic stress treatments. The expression patterns of 170 class III peroxidase genes under abiotic stress treatments (cold, heat, drought and nutrient deficiency) are presented. Normalized gene expression values are provided in Additional file 21: Table S9. (PDF 1333 kb) [file 12864_2019_6006_MOESM7_ESM.pdf]

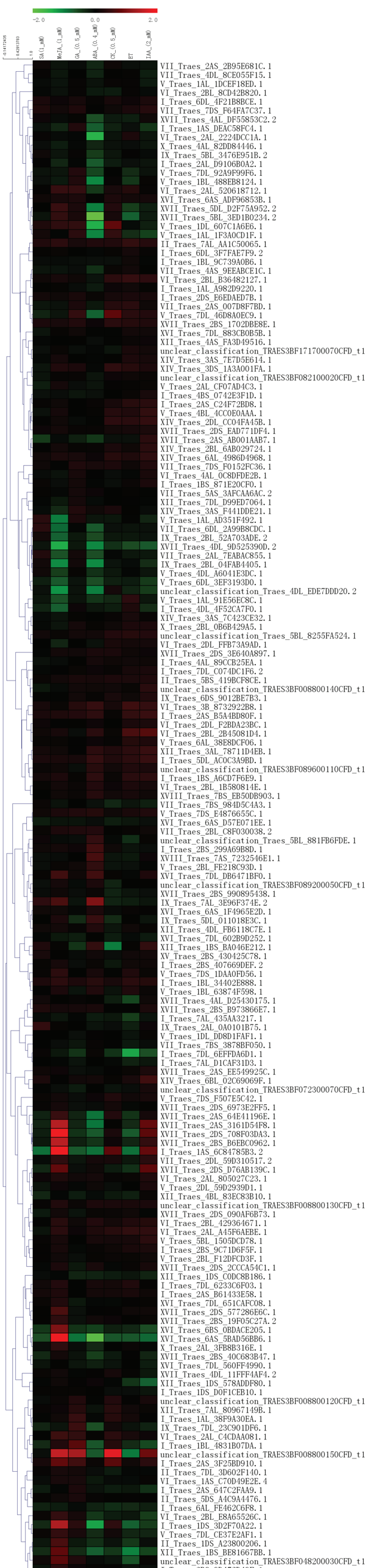

Supplement: Supplementary file 8 — Figure S8. Heatmap of the expression patterns of T. aestivum class III peroxidase genes under seven phytohormone treatments. The expression patterns of 170 class III peroxidase genes under seven phytohormone treatments, including IAA, GA (GA3), ABA, ET, CK (trans-zeatin), SA and MeJA, are presented. Normalized gene expression values are provided in Additional file 21: Table S9. (PDF 1226 kb) [file 12864_2019_6006_MOESM8_ESM.pdf]

A

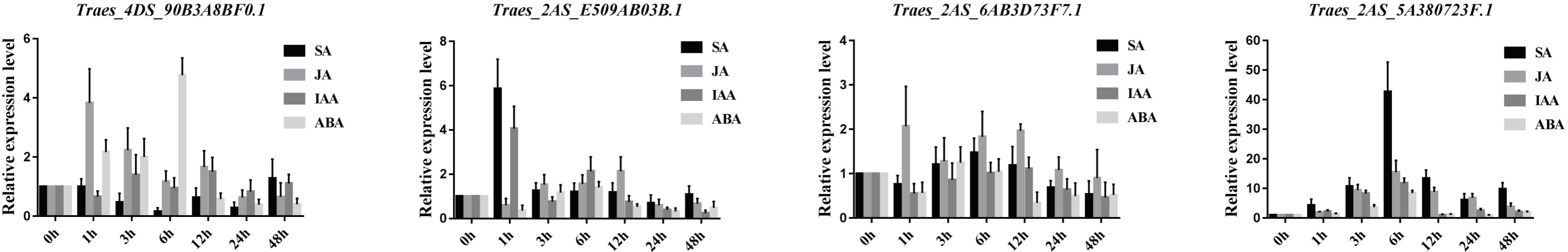

B

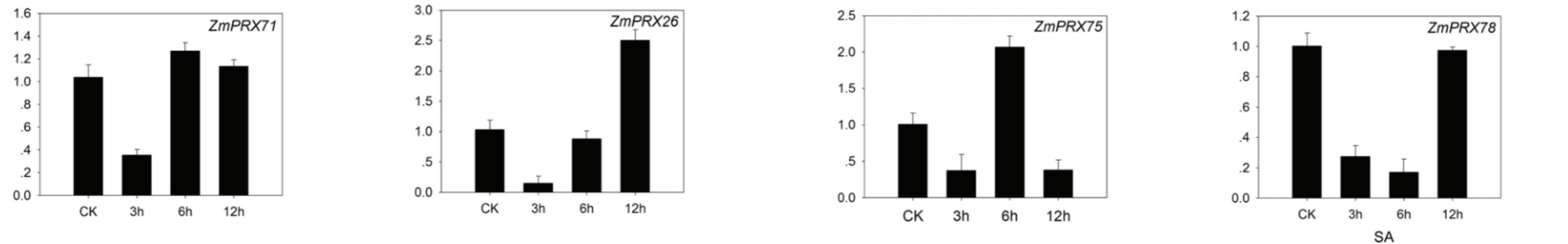

Supplement: Supplementary file 11 — Figure S11. qRT-PCR of four T. aestivum PRXs under four phytohormone treatments and homologous maize PRXs under SA treatment. (A) qRT-PCR of T. aestivum PRXs under SA, JA, IAA and ABA treatments. (B) Homologous maize PRXs under SA treatment. (PDF 912 kb) [file 12864_2019_6006_MOESM11_ESM.pdf]

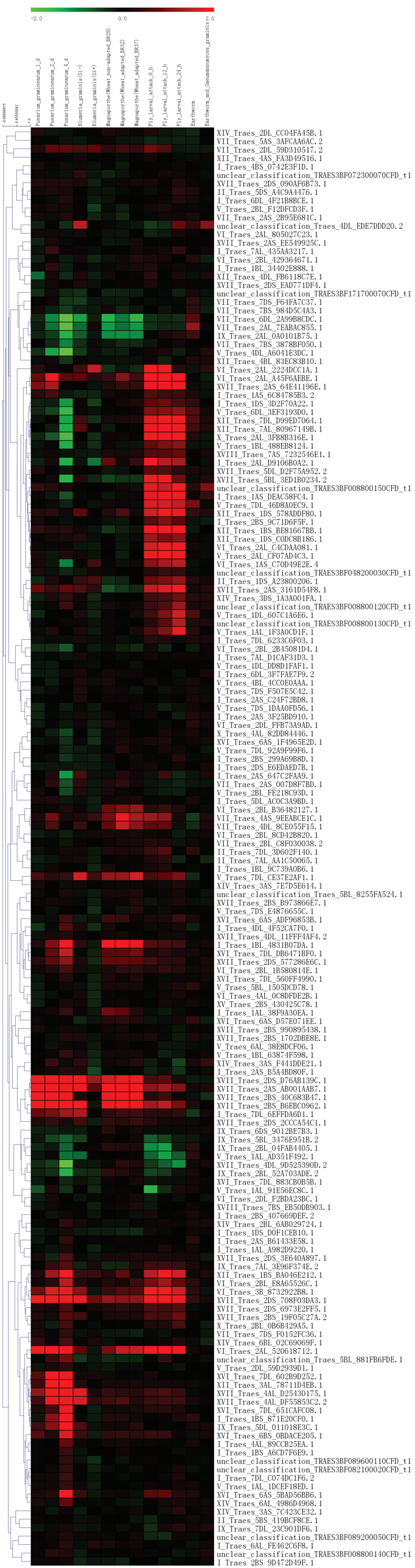

Supplement: Supplementary file 12 — Figure S12. Heatmap of the expression patterns of T. aestivum class III peroxidase genes under biotic stress treatments. The expression patterns of 170 class III peroxidase genes under biotic stress treatments (Fusarium head blight, powdery mildew, blast fungus, Hessian fly larvae and earthworms) are presented. Normalized gene expression values are provided in Additional file 24: Table S12. (PDF 1418 kb) [file 12864_2019_6006_MOESM12_ESM.pdf]
