# Supplementary material for: Genome-wide and evolutionary analysis of the class III peroxidase gene family in wheat and Aegilops tauschii reveals that some members are involved in stress responses
Source: BMC Genomics. 2019 Aug 22;20:666. doi: 10.1186/s12864-019-6006-5 (PMC6704529; doi:10.1186/s12864-019-6006-5)

GSE12508（tissue）

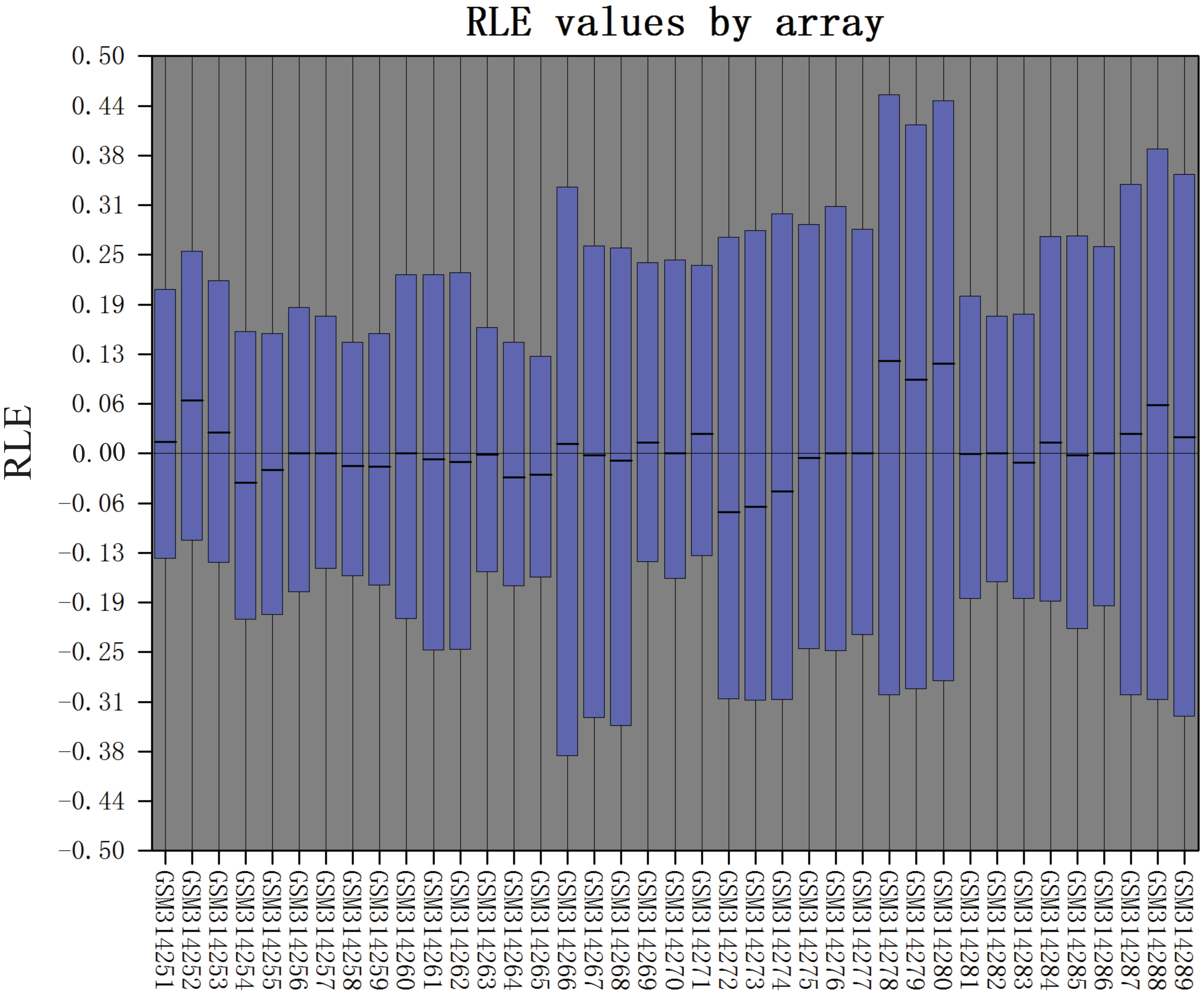

GSE12508（tissue）

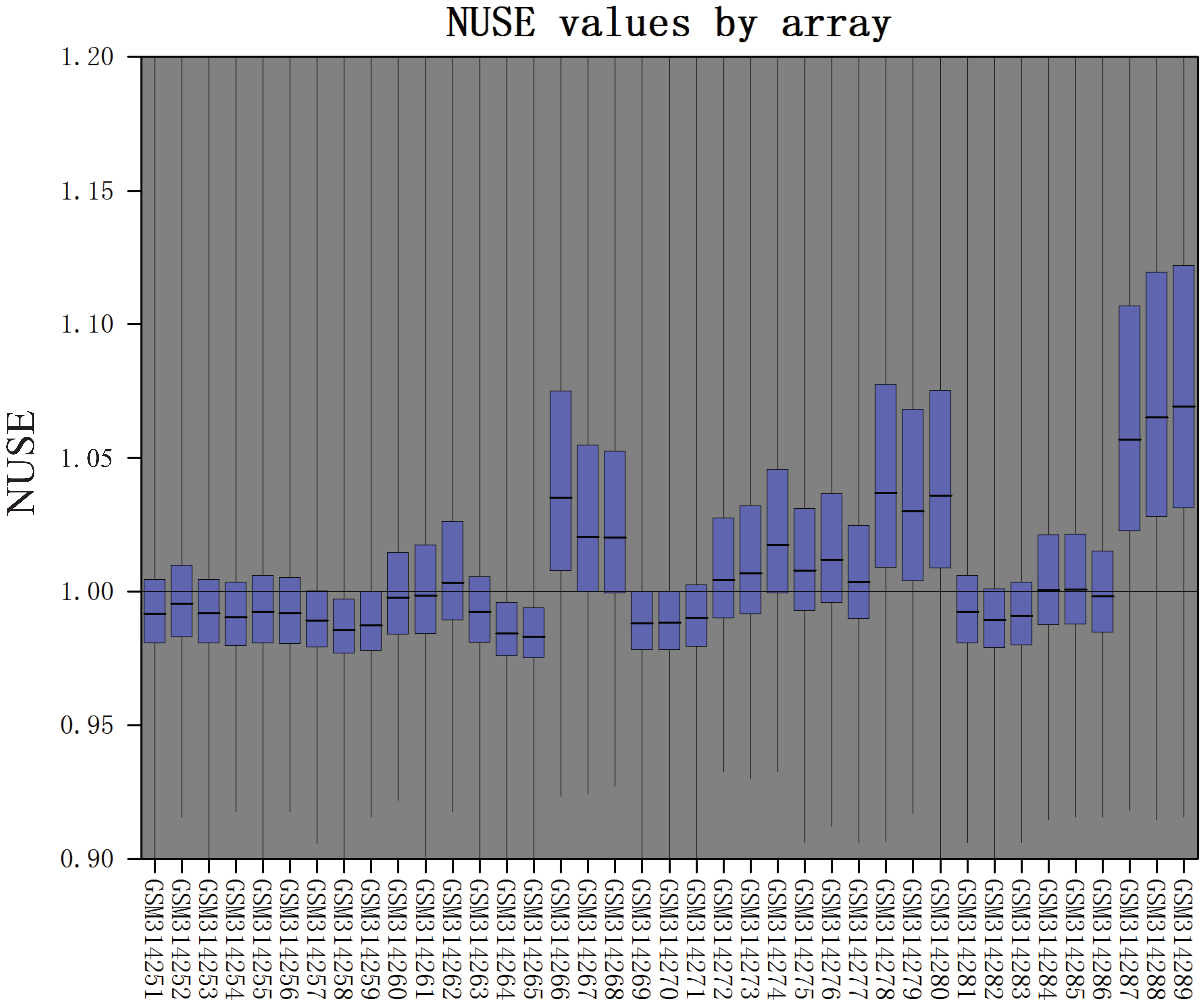

GSE14697 (cold)

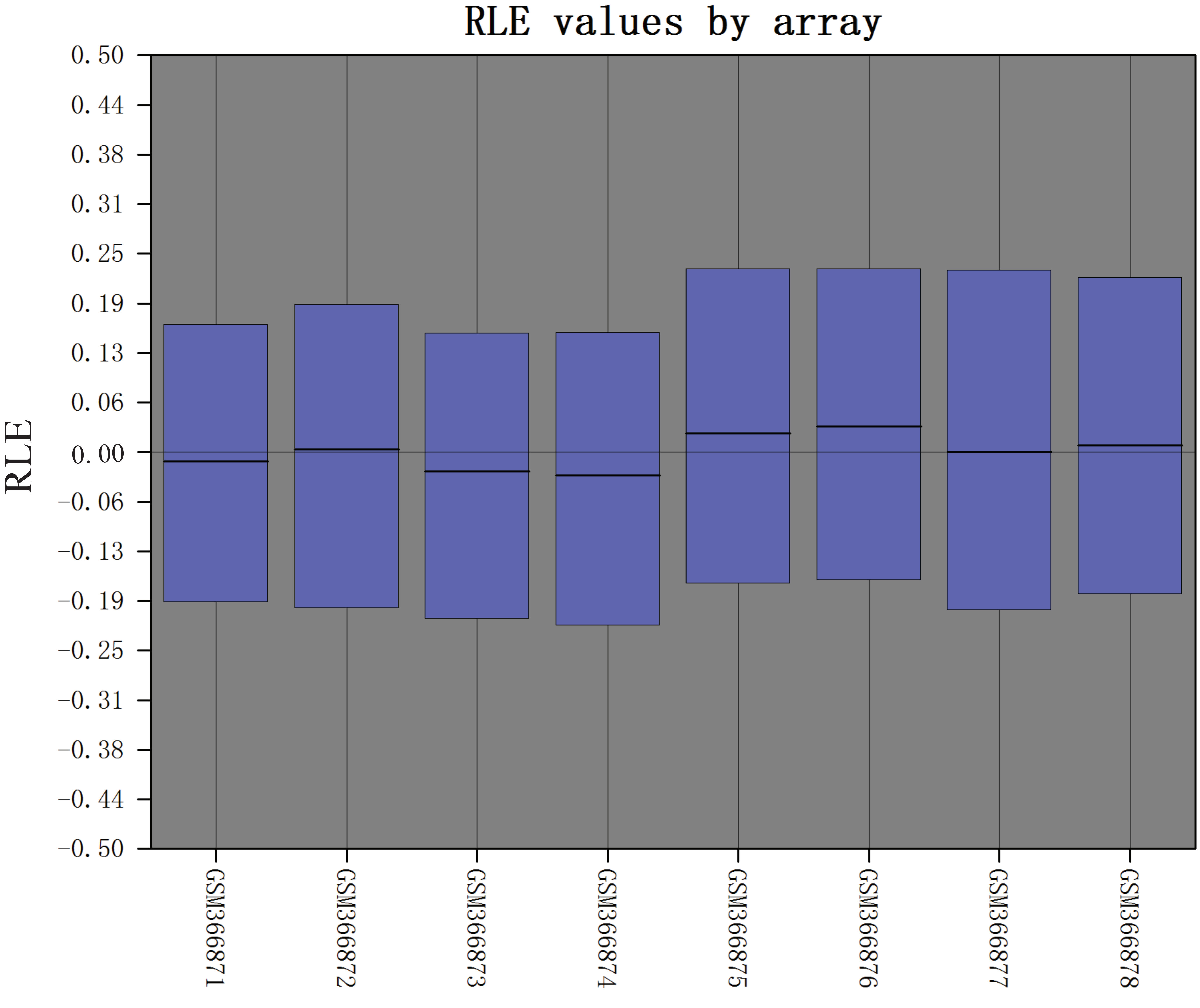

GSE14697（cold）

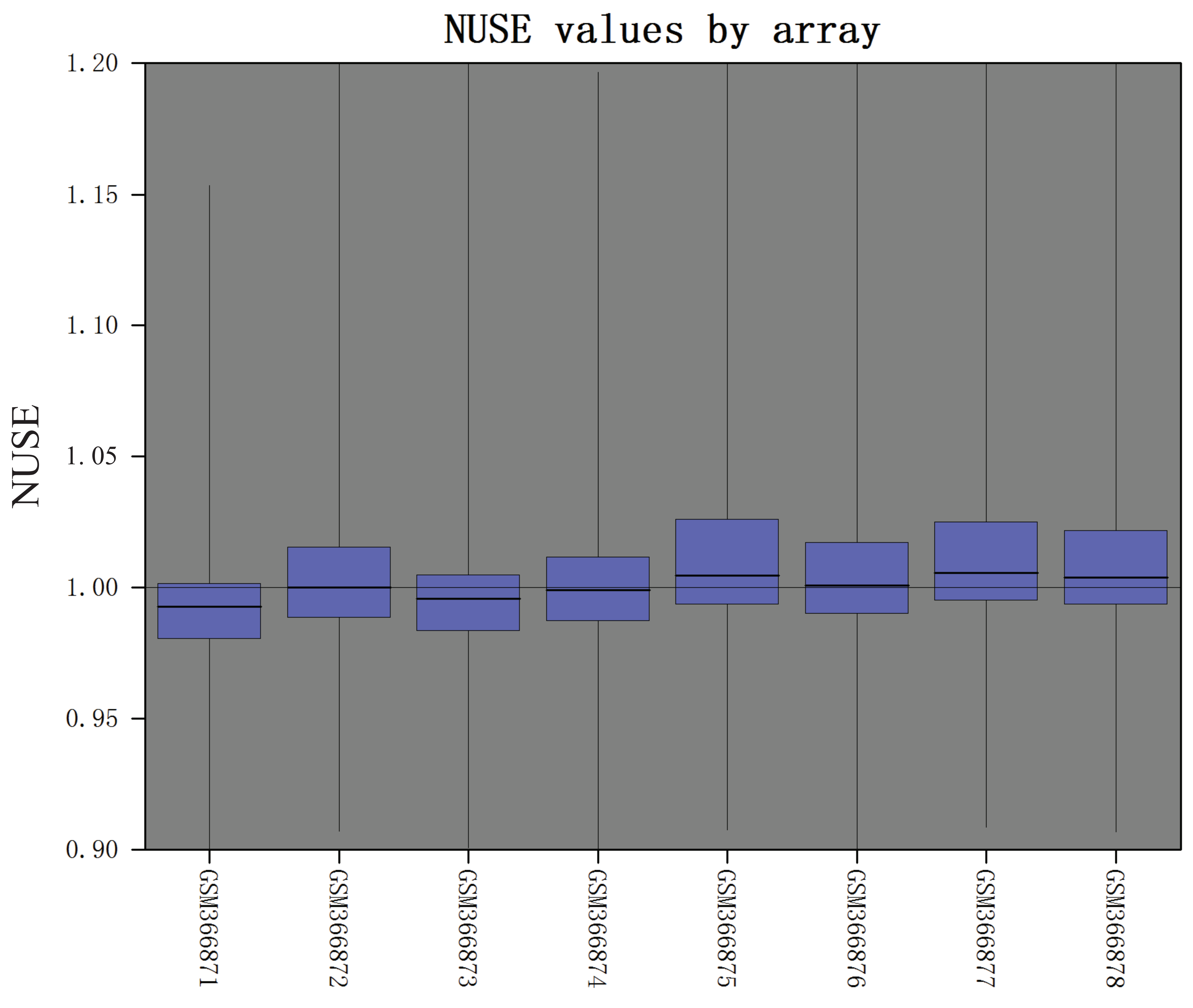

GSE60351（heat）

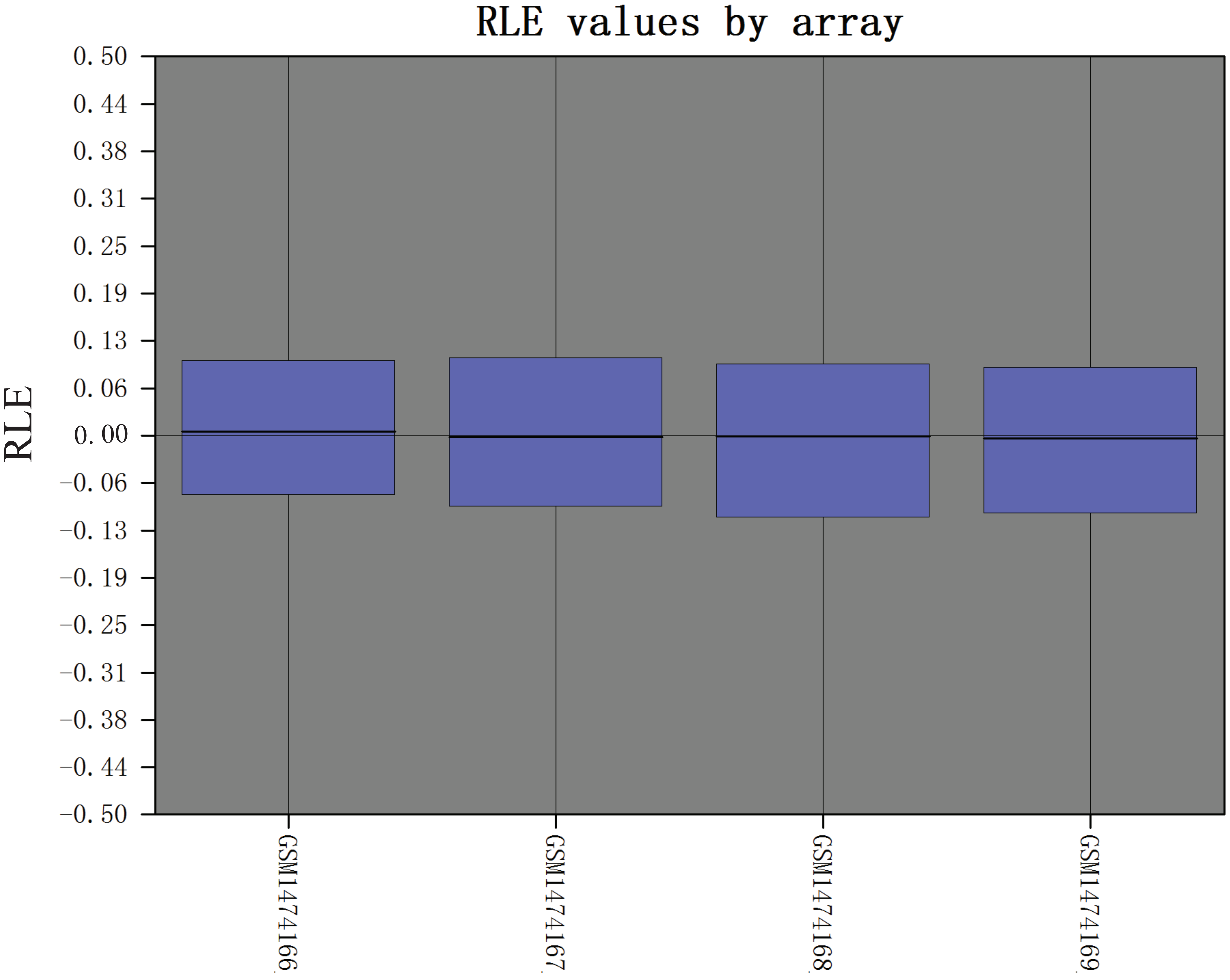

GSE60351（heat）

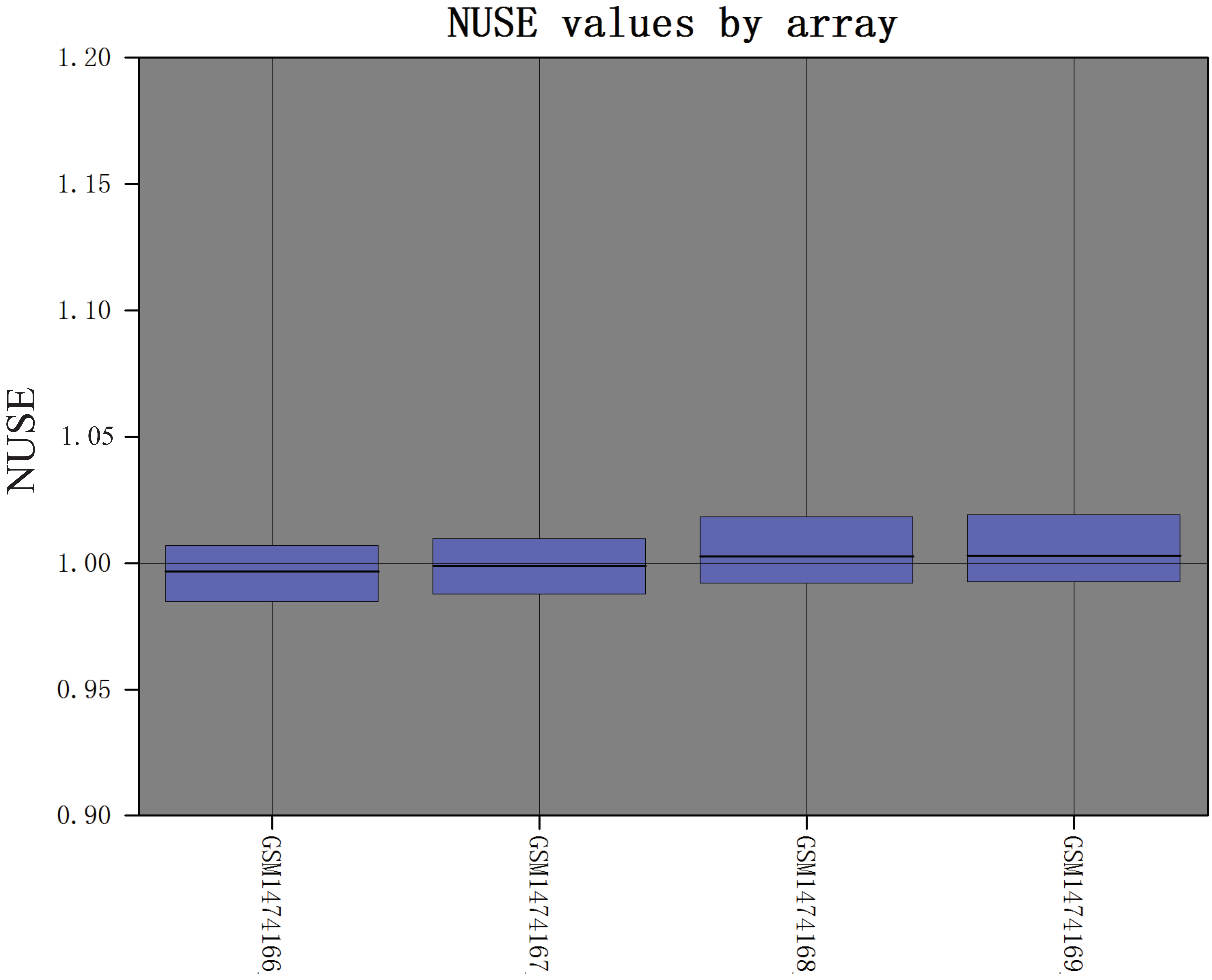

GSE87325（drought）

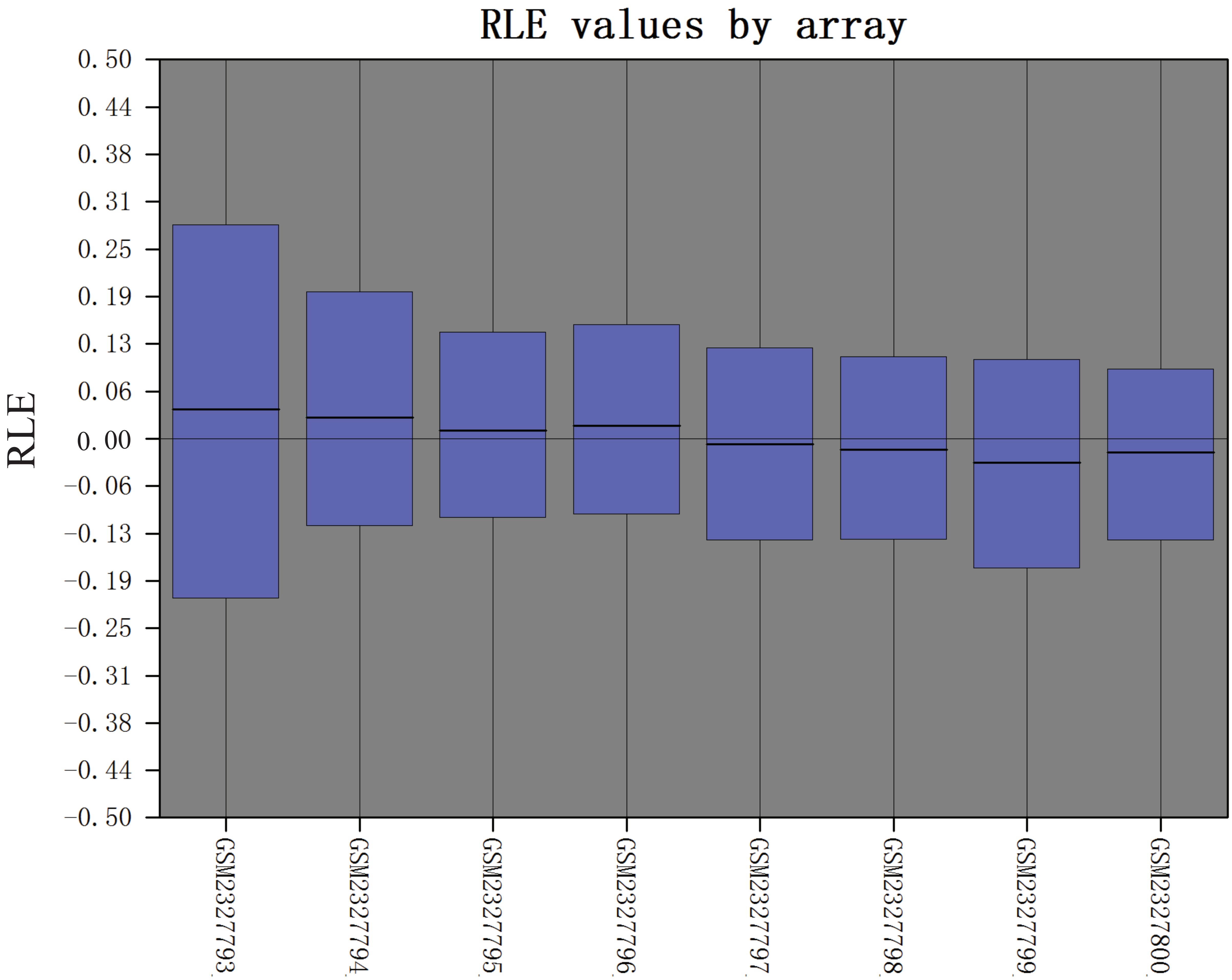

GSE87325（drought）

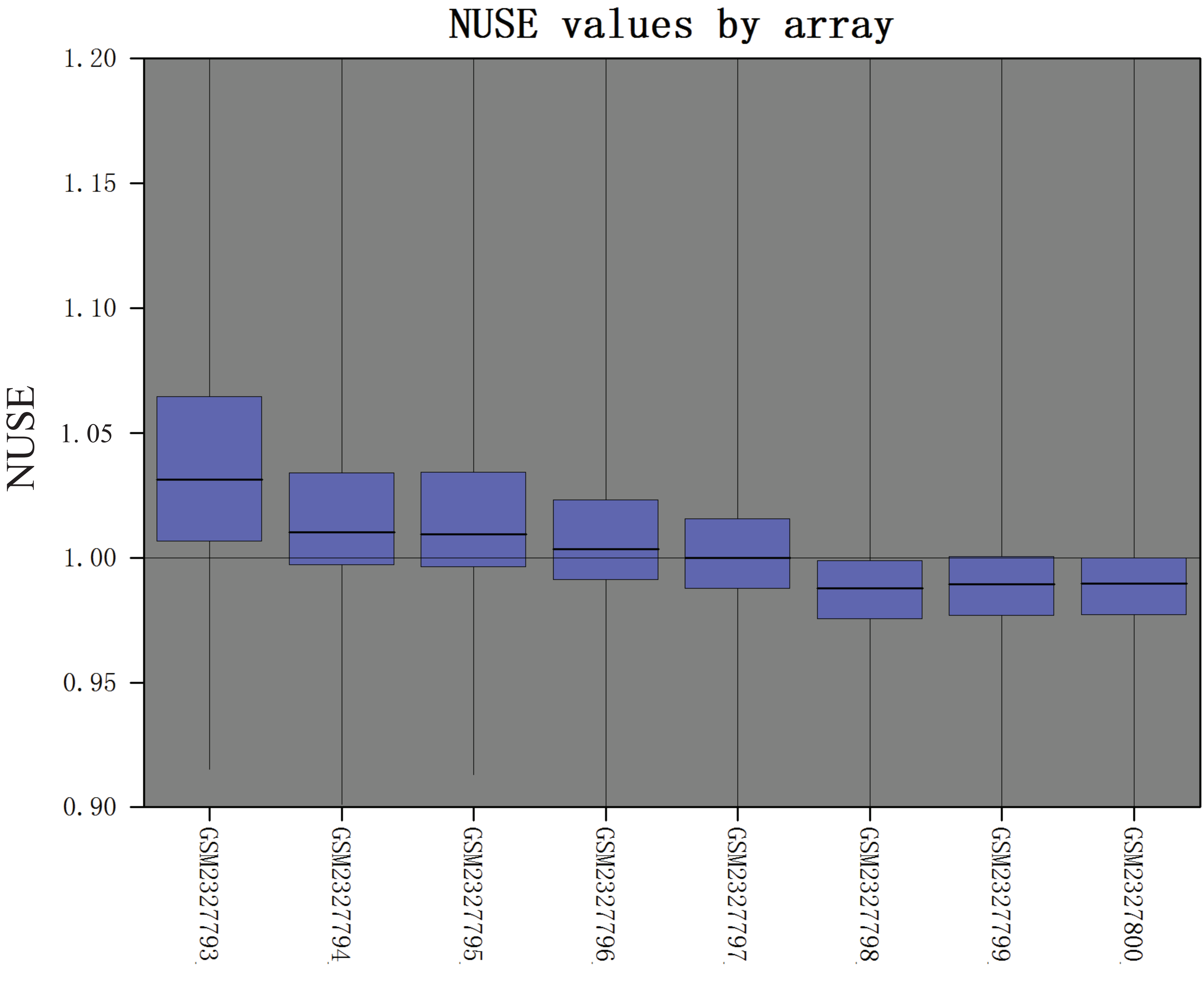

GSE61679(nutrient deficient)

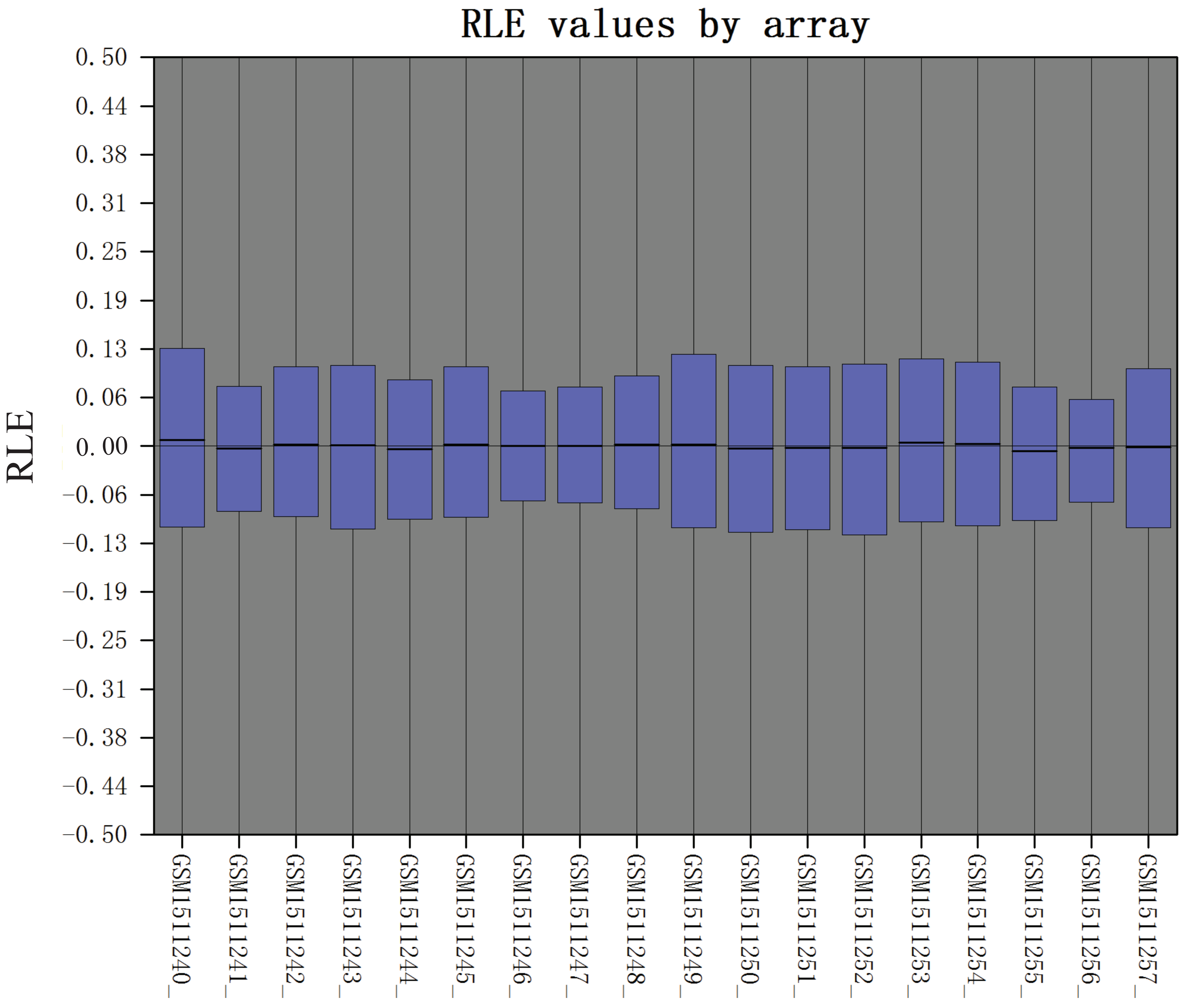

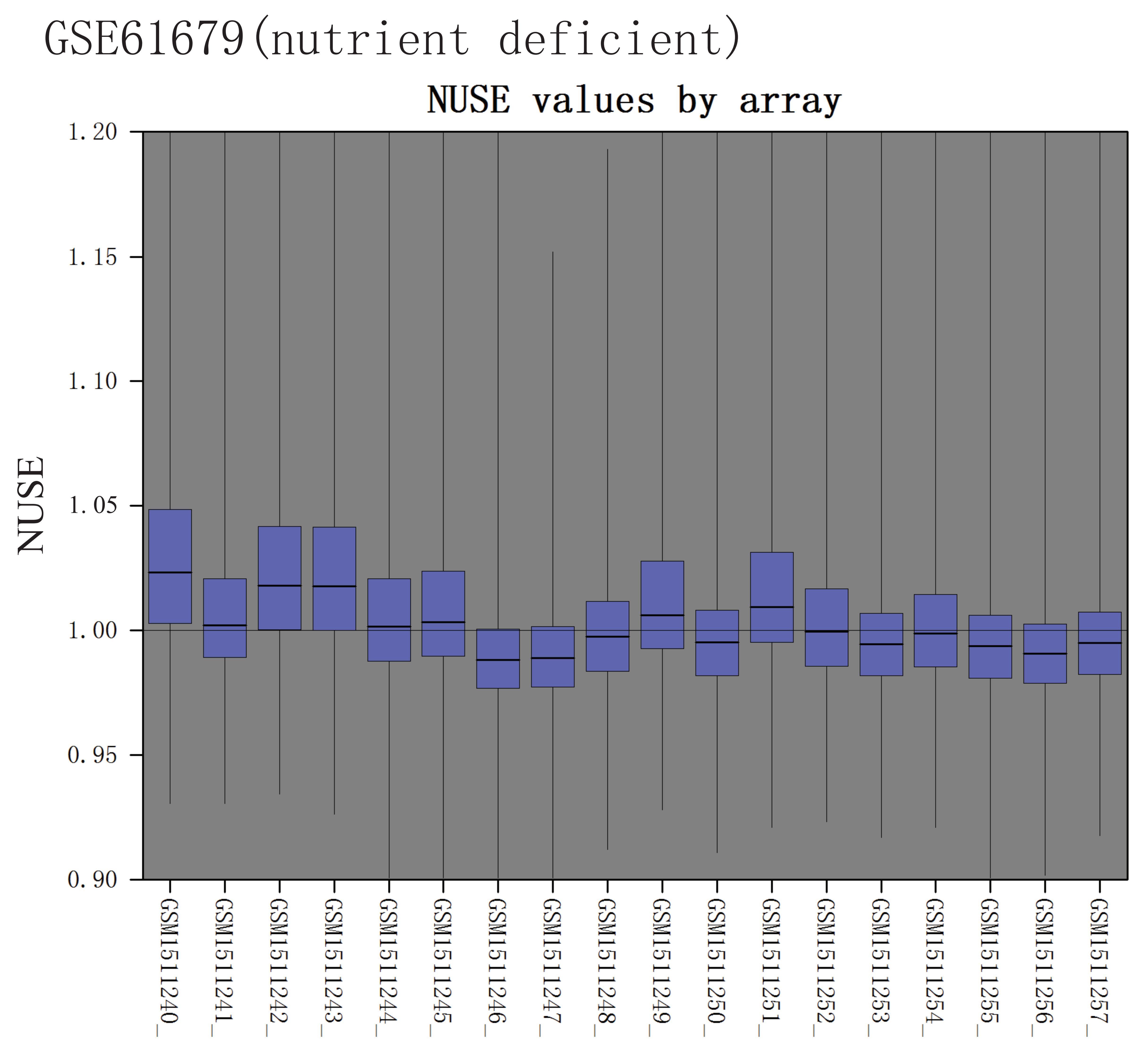
GSE61679(nutrient deficient)
NUSE values by array
NUSE
1.20
1.15
1.10
1.05
1.00
0.95
0.90
GSM1511240
GSM1511241
GSM1511242
GSM1511243
GSM1511244
GSM1511245
GSM1511246
GSM1511247
GSM1511248
GSM1511249
GSM1511250
GSM1511251
GSM1511252
GSM1511253
GSM1511254
GSM1511255
GSM1511256
GSM1511257

GSE103430 (seven phytohormones)

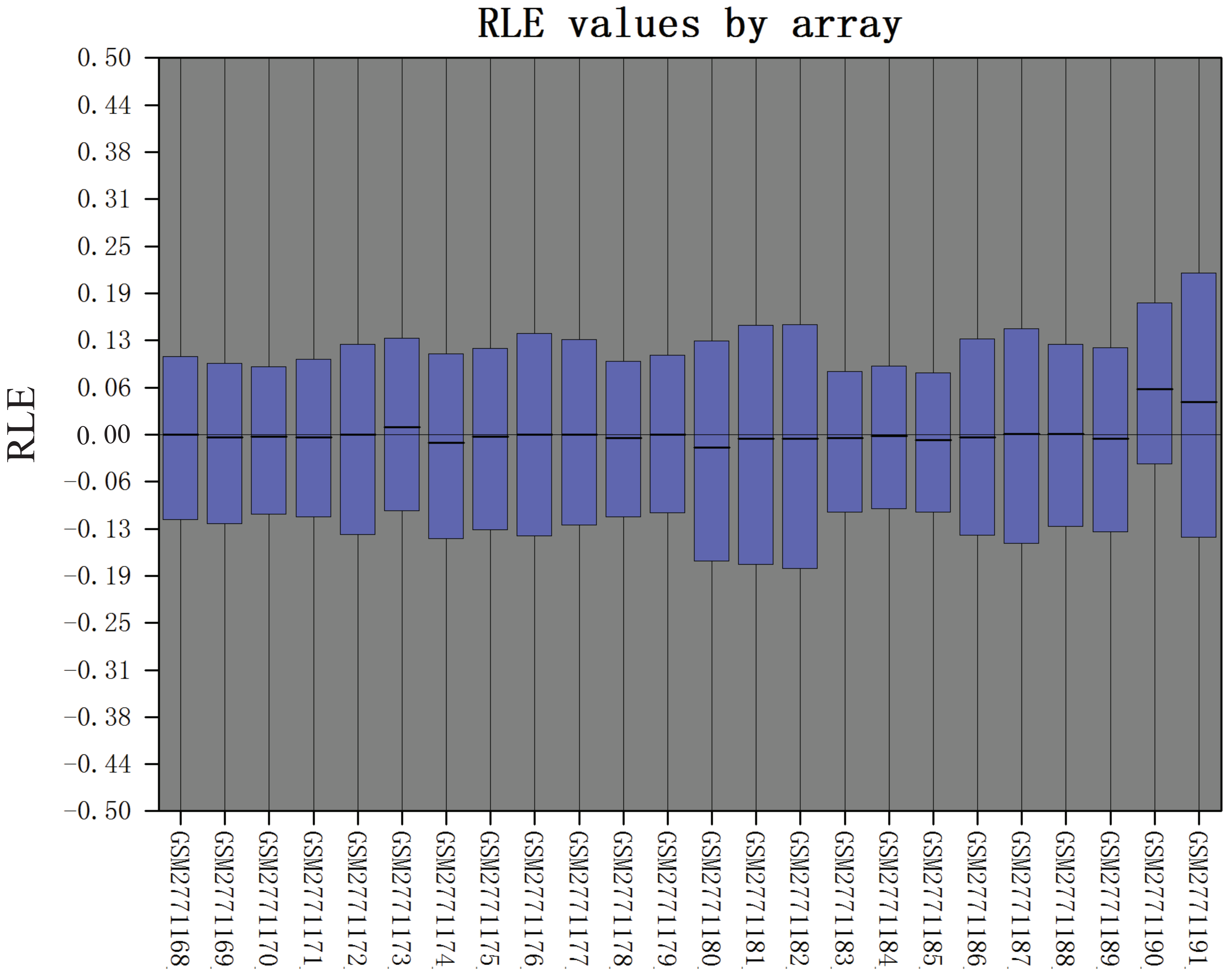

GSE103430 (seven phytohormones)

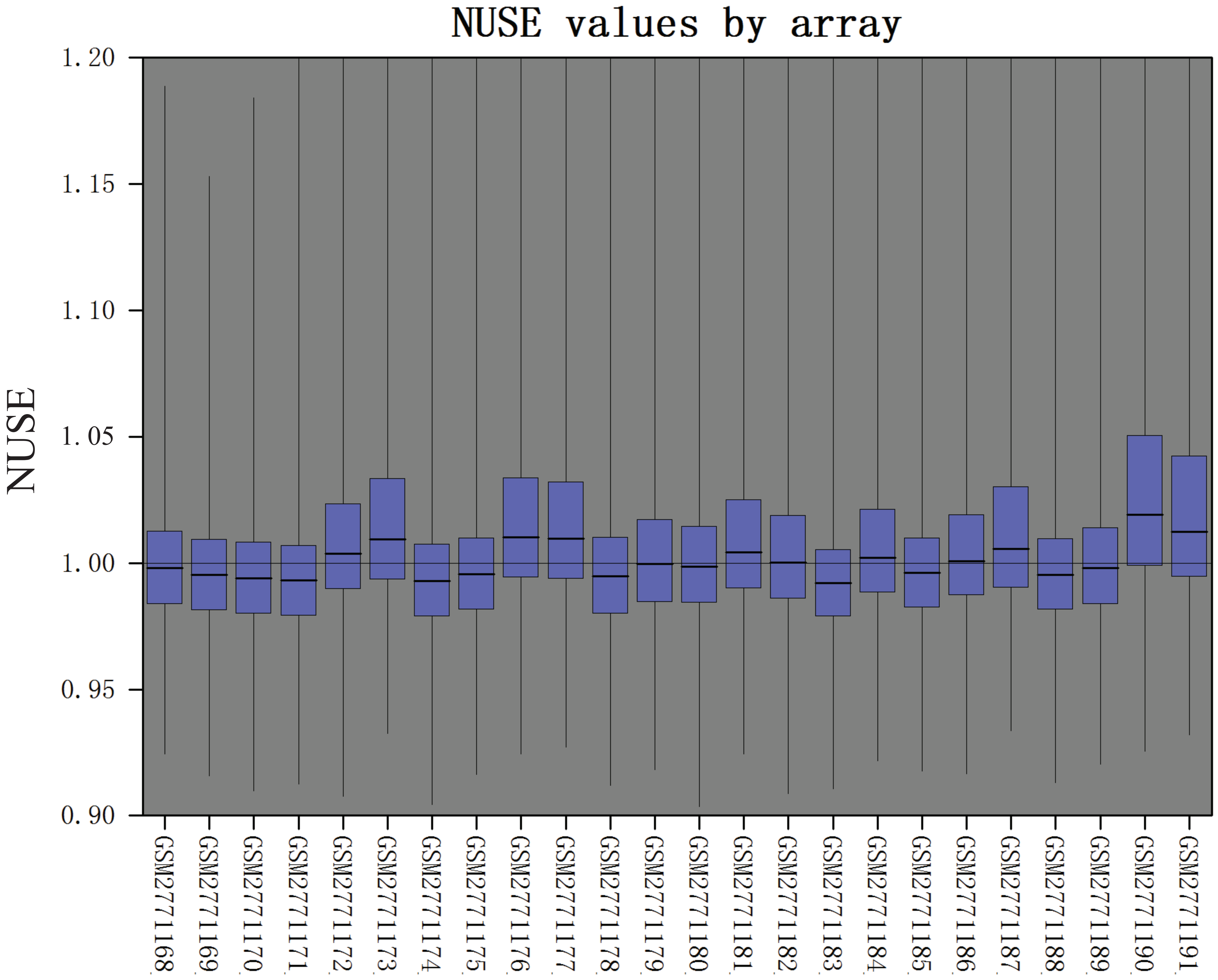

GSE36283(Fusarium Head Blight)

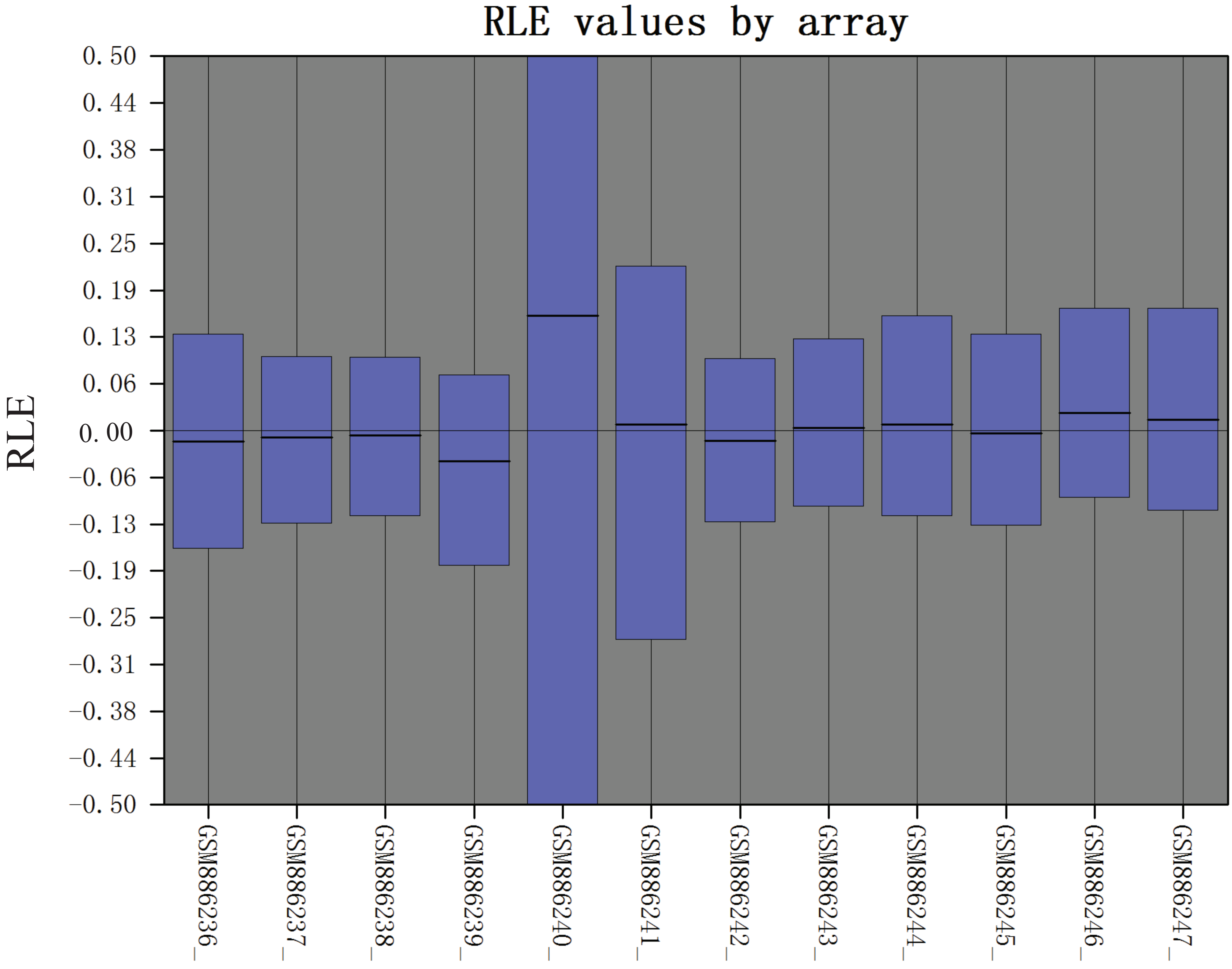

GSE36283(Fusarium Head Blight)

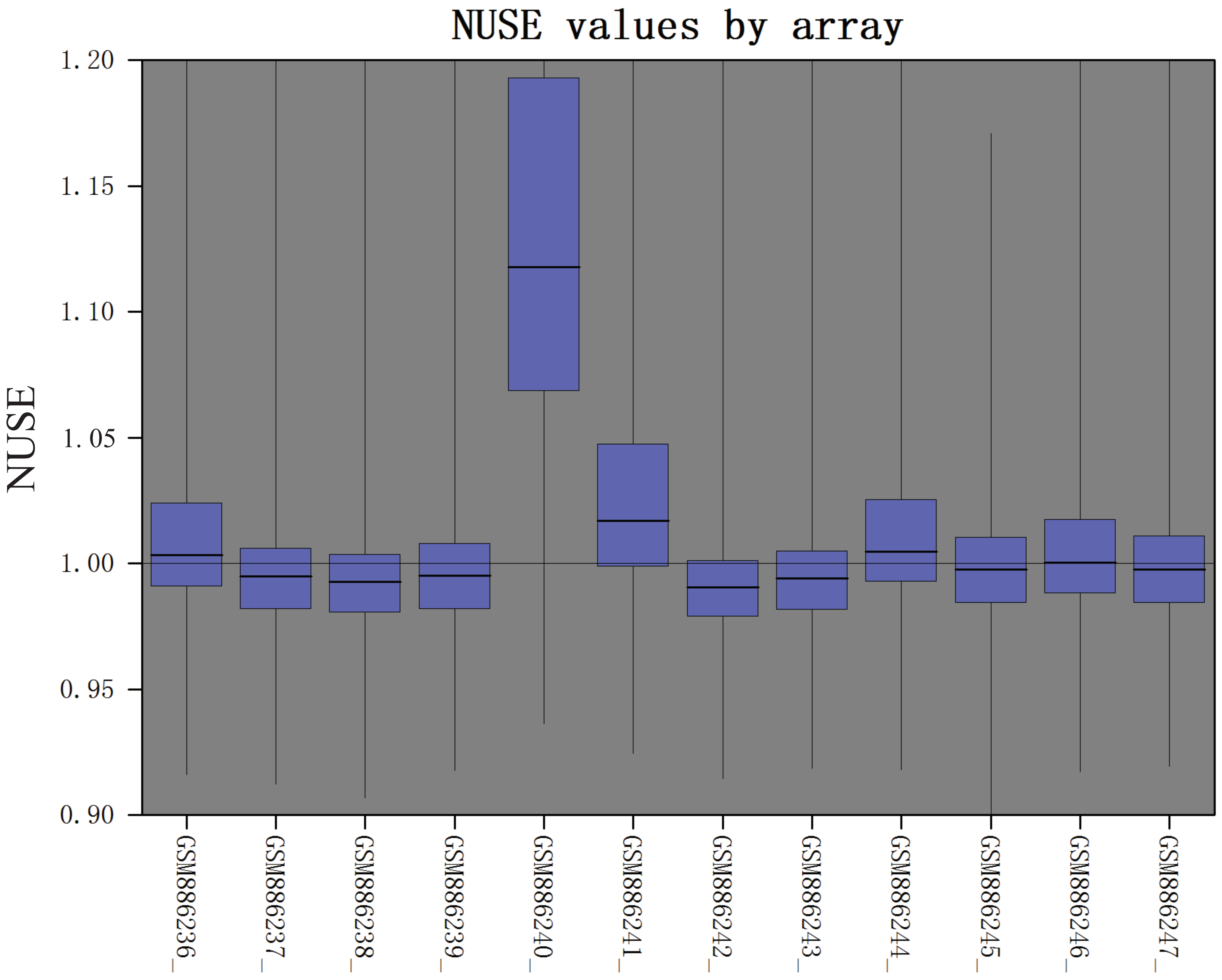

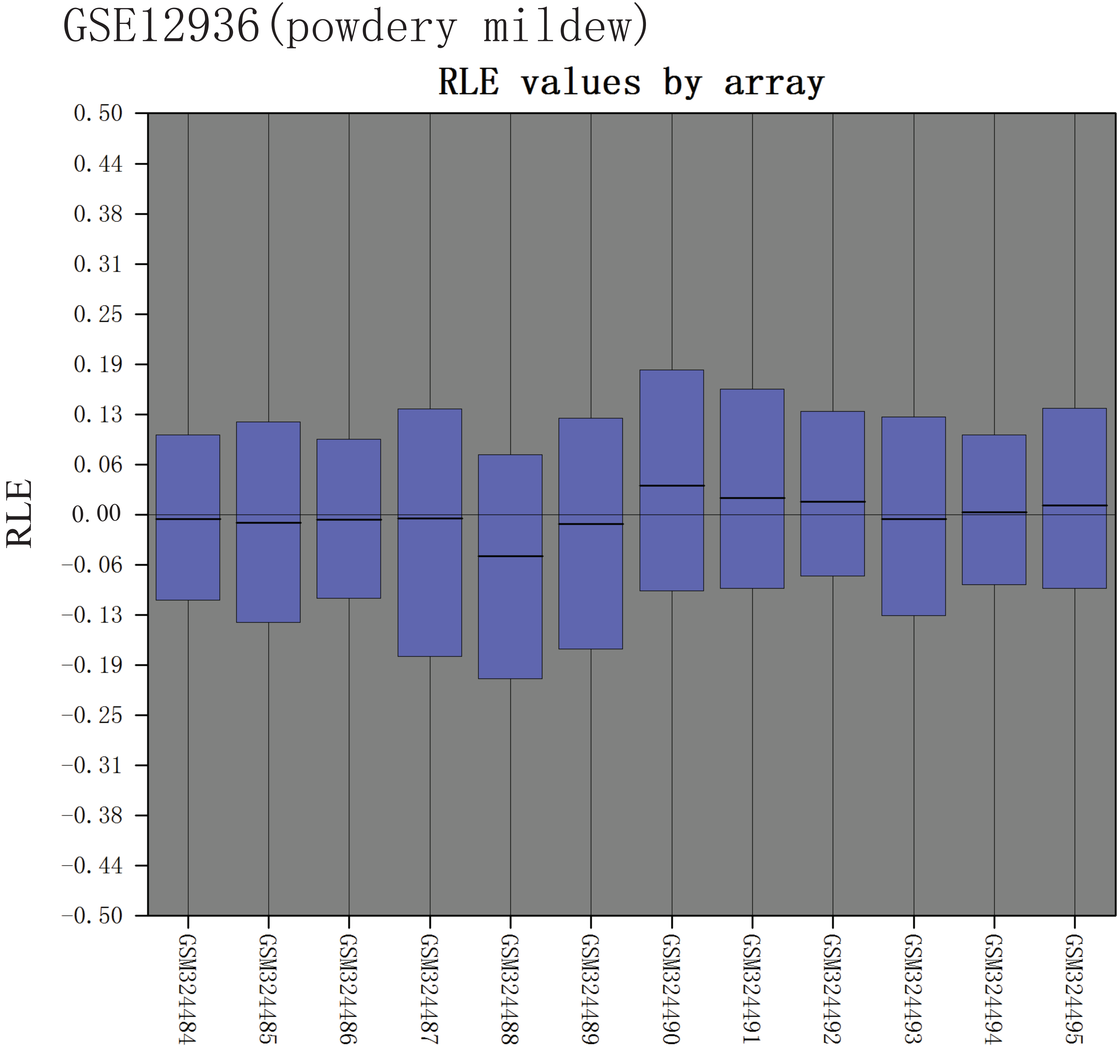
GSE12936(powdery mildew)
RLE values by array
RLE
0.50
0.44
0.38
0.31
0.25
0.19
0.13
0.06
0.00
−0.06
−0.13
−0.19
−0.25
−0.31
−0.38
−0.44
−0.50
GSM324484
GSM324485
GSM324486
GSM324487
GSM324488
GSM324489
GSM324490
GSM324491
GSM324492
GSM324493
GSM324494
GSM324495

GSE12936(powdery mildew)

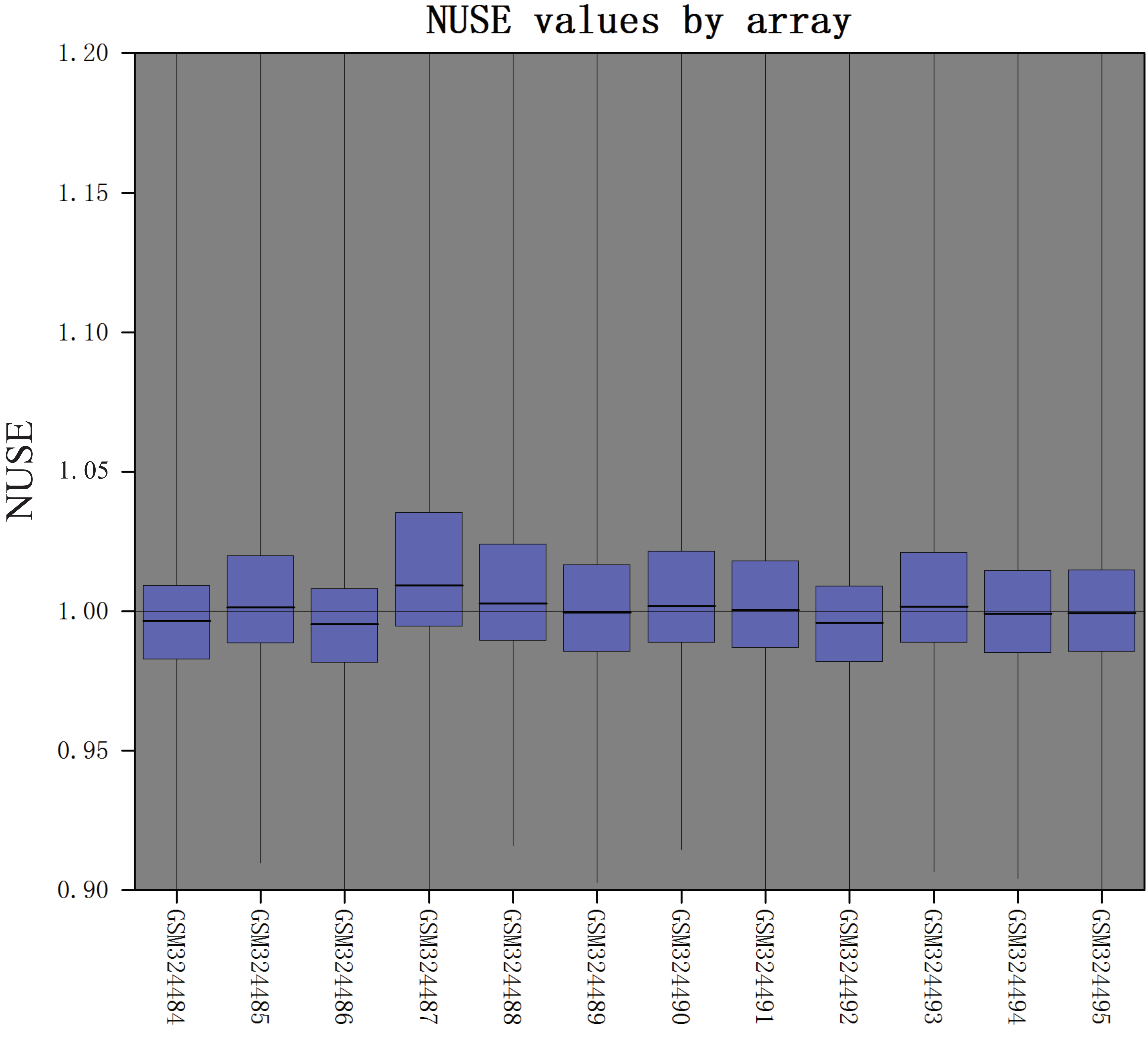

GSE31760(blast fungus)

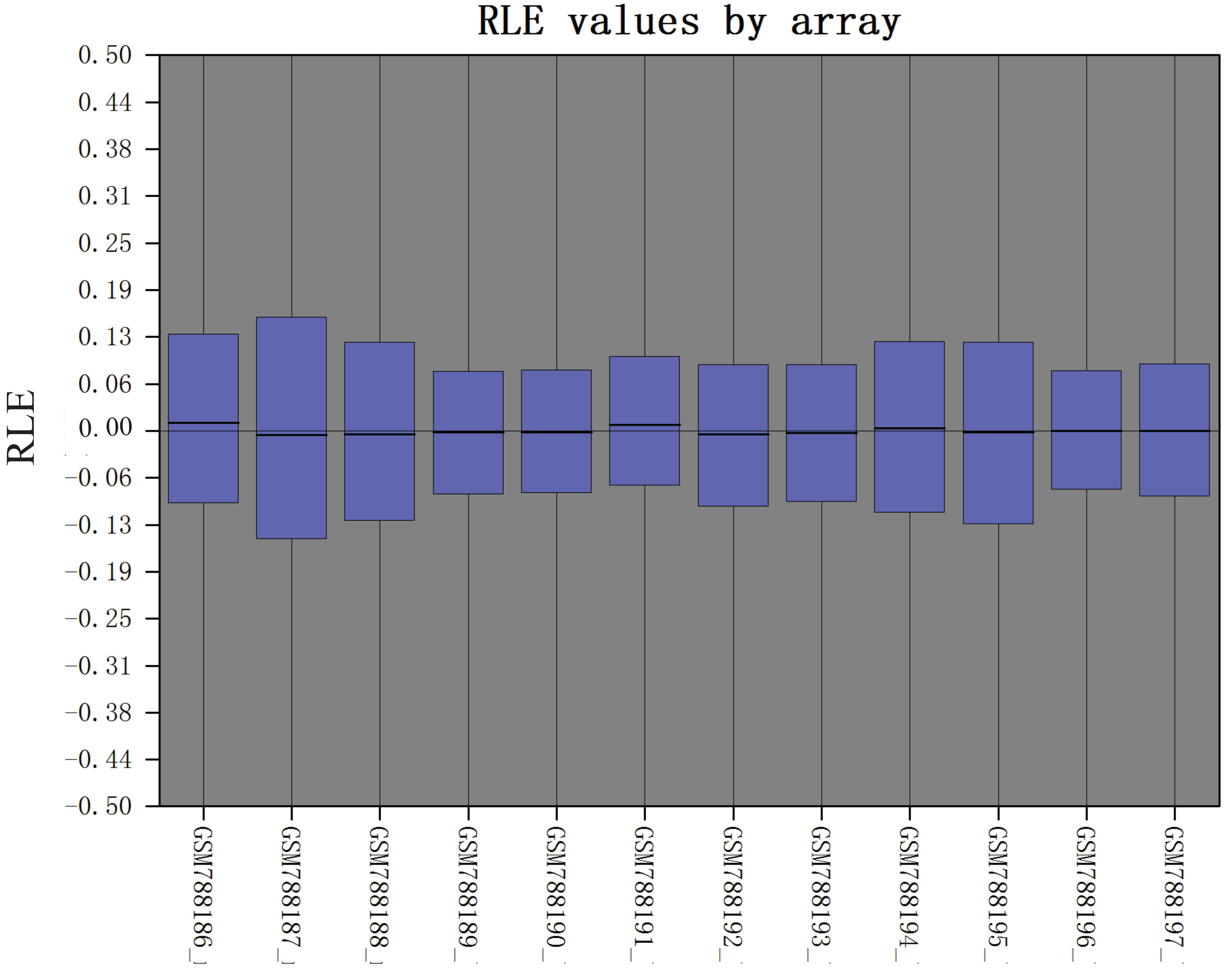

GSE31760(blast fungus)

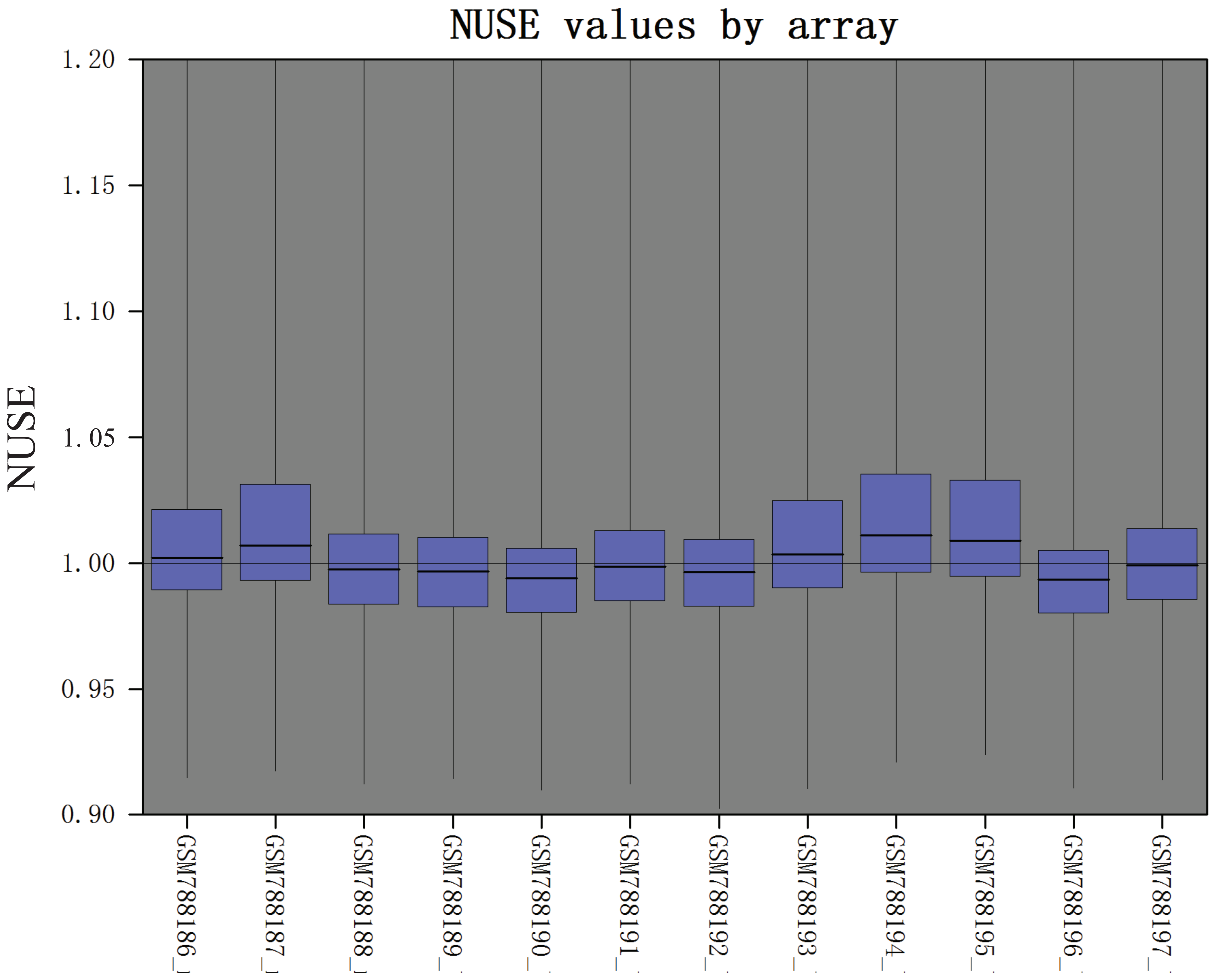

GSE34445（Hessian fly larval）

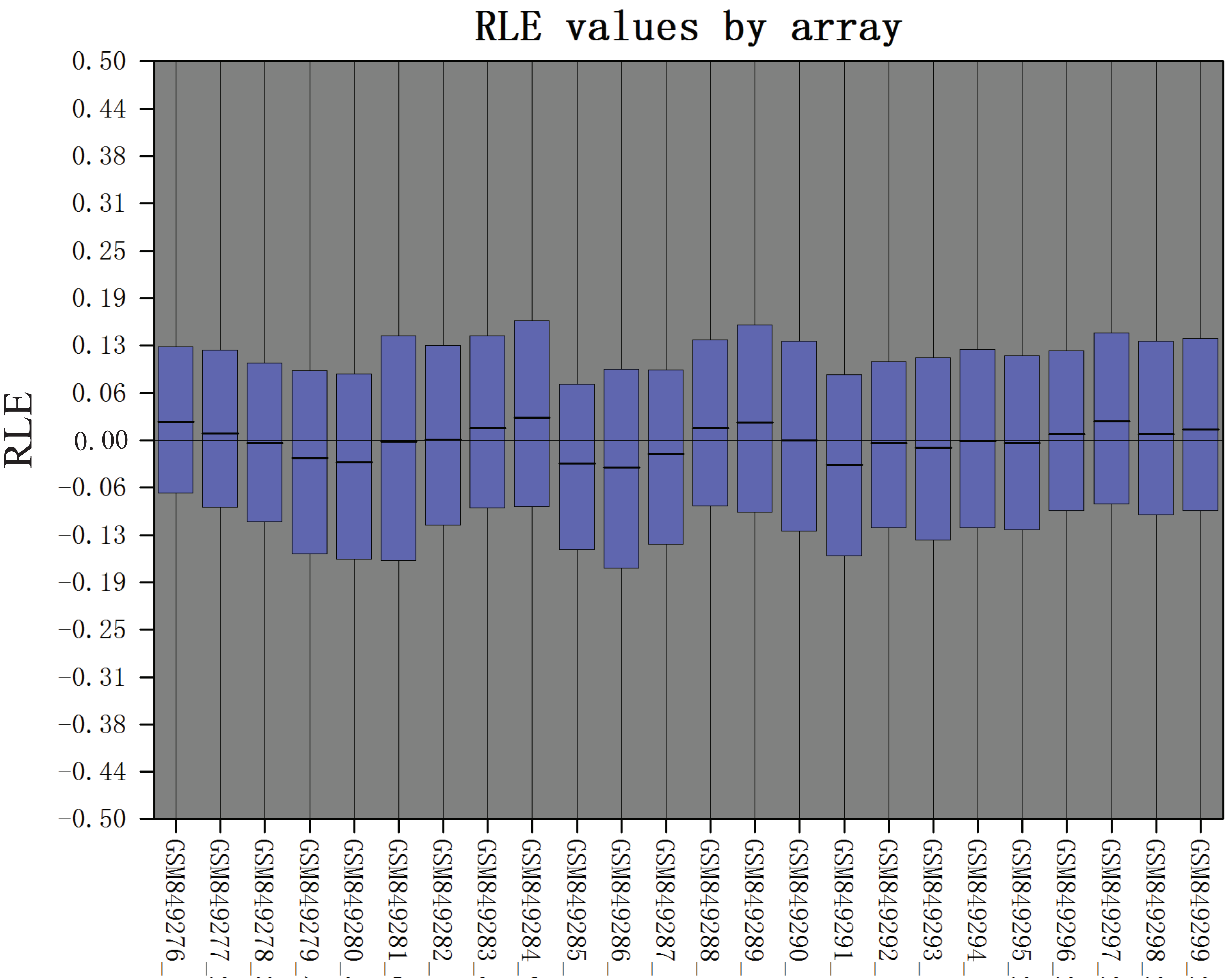

GSE34445 (Hessian fly larval)

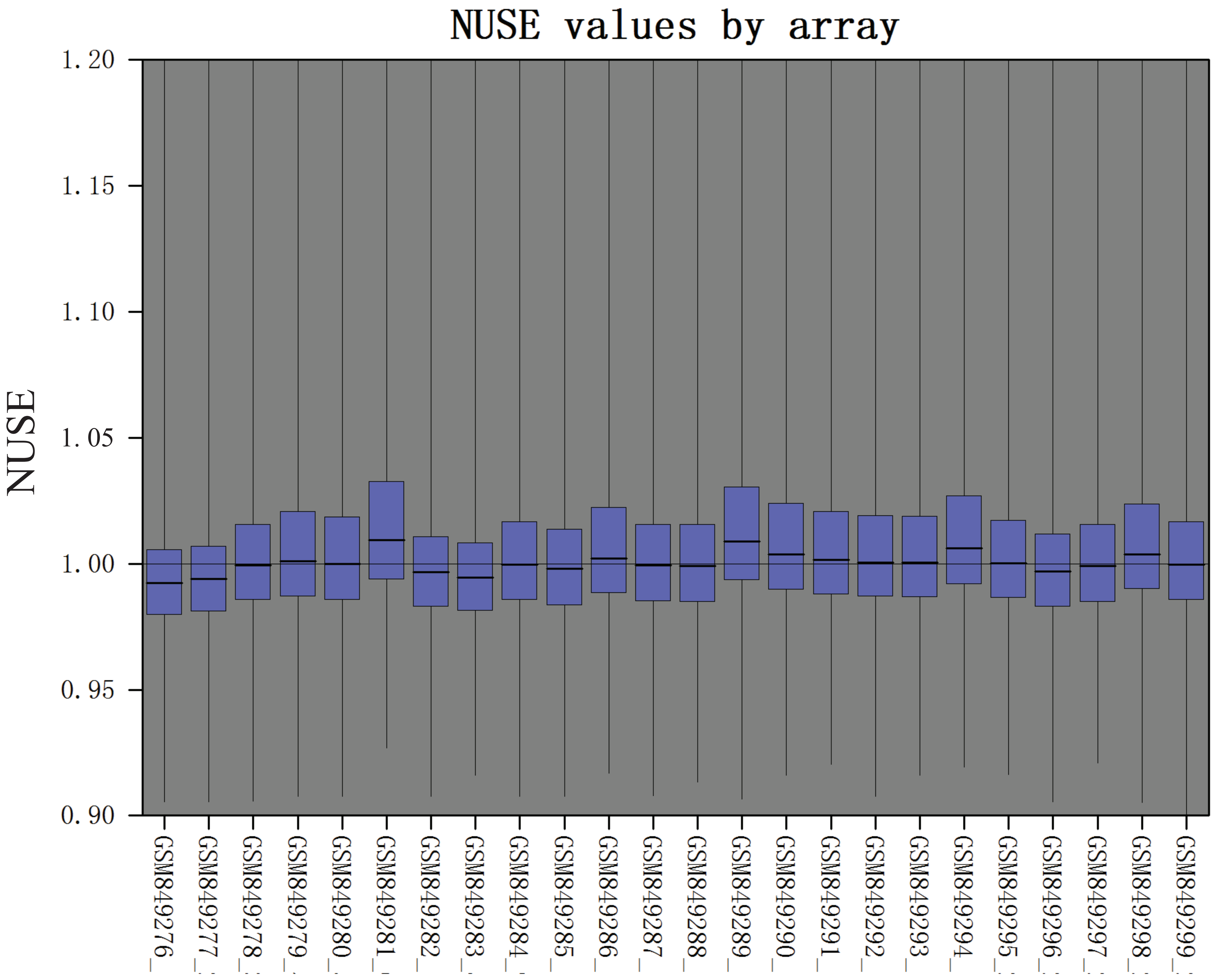

GSE47479（earthworms and *Gaeumannomyces graminis*）

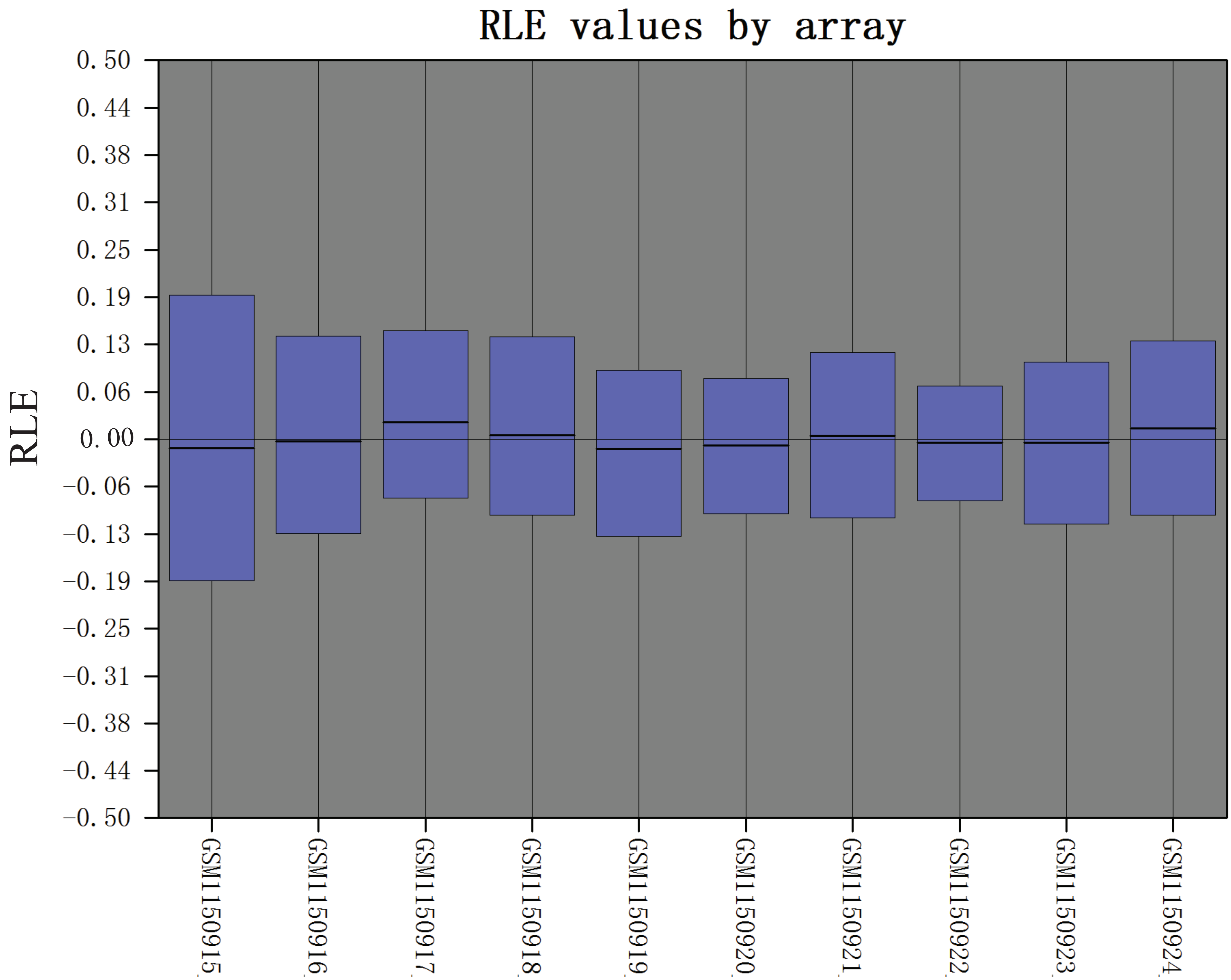

GSE47479（earthworms and *Gaeumannomyces graminis*）

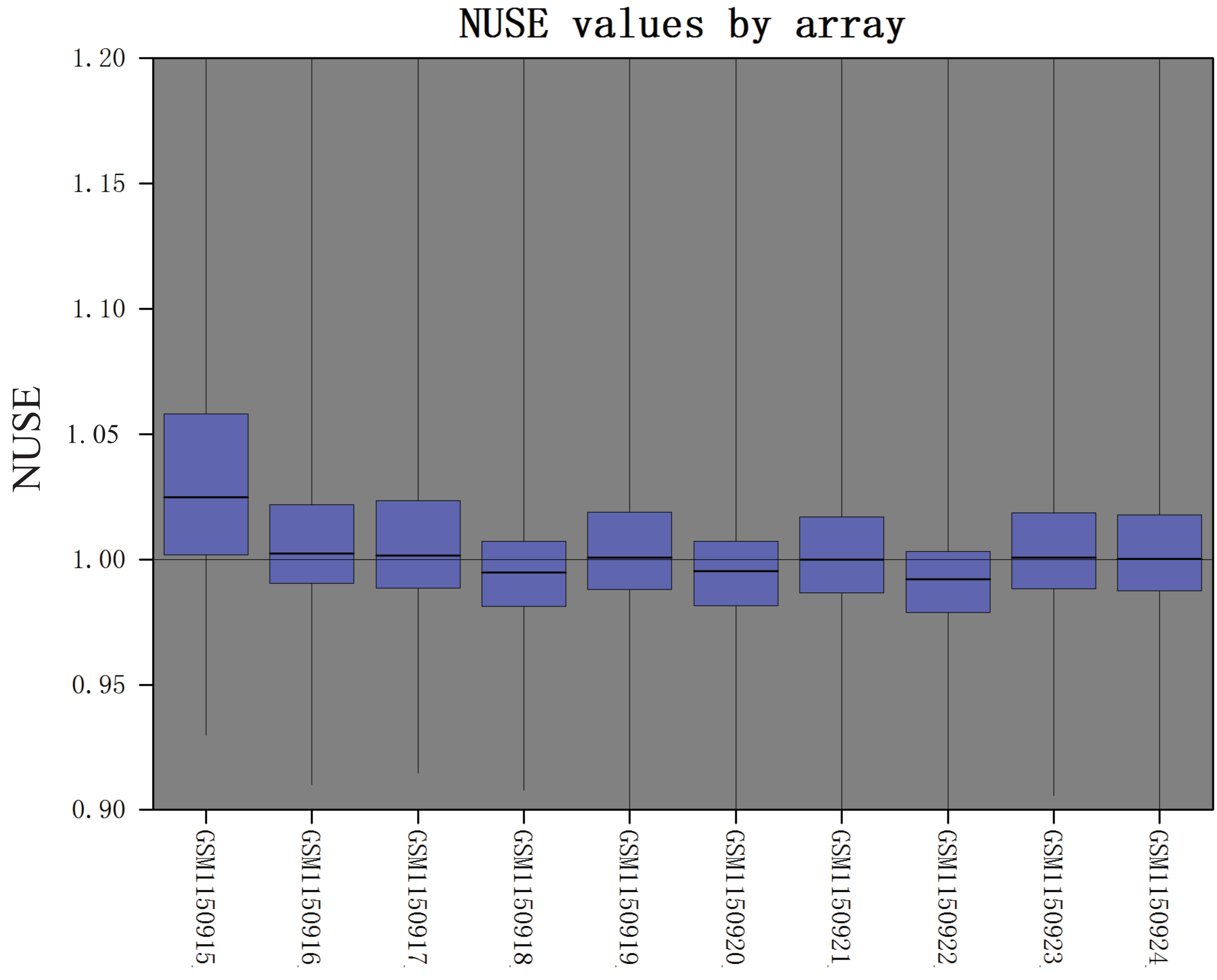

Supplement: Supplementary file 5 — Figure S5. Quality control of GEO microarray datasets. RLE (Relative log expression) and NUSE (Normalized unscaled standard errors) values of each GEO microarray dataset. (PDF 2638 kb) [file 12864_2019_6006_MOESM5_ESM.pdf]
