# Supplementary material for: Genome-wide and evolutionary analysis of the class III peroxidase gene family in wheat and Aegilops tauschii reveals that some members are involved in stress responses
Source: BMC Genomics. 2019 Aug 22;20:666. doi: 10.1186/s12864-019-6006-5 (PMC6704529; doi:10.1186/s12864-019-6006-5)

1A

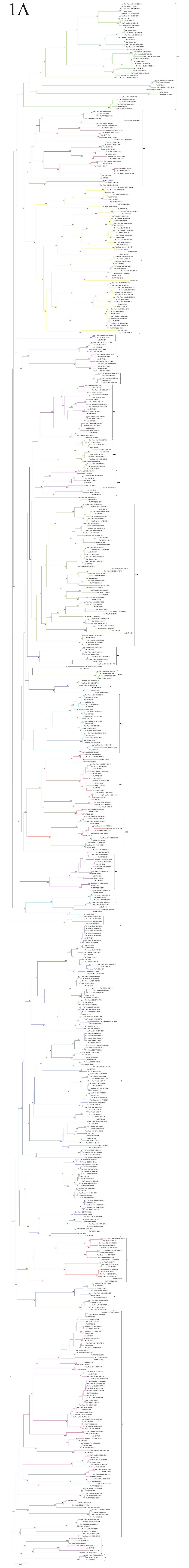

1B

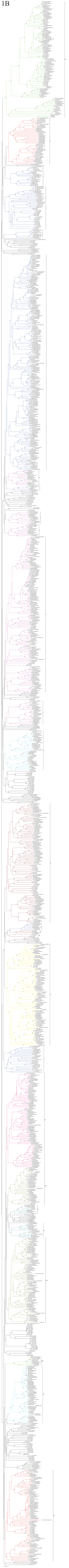

Supplement: Supplementary file 1 — Figure S1. Expanded phylogenetic classification of class III peroxidases using the neighbour-joining method. (a) T. aestivum, T. urartu and Ae. tauschii; (b) All investigated plants. (PDF 376 kb) [file 12864_2019_6006_MOESM1_ESM.pdf]
