# Supplementary material for: Genome-wide and evolutionary analysis of the class III peroxidase gene family in wheat and Aegilops tauschii reveals that some members are involved in stress responses
Source: BMC Genomics. 2019 Aug 22;20:666. doi: 10.1186/s12864-019-6006-5 (PMC6704529; doi:10.1186/s12864-019-6006-5)

upstream of Traes_2DS_D76AB139C.1 (+, 5'->3') and its sequencing data

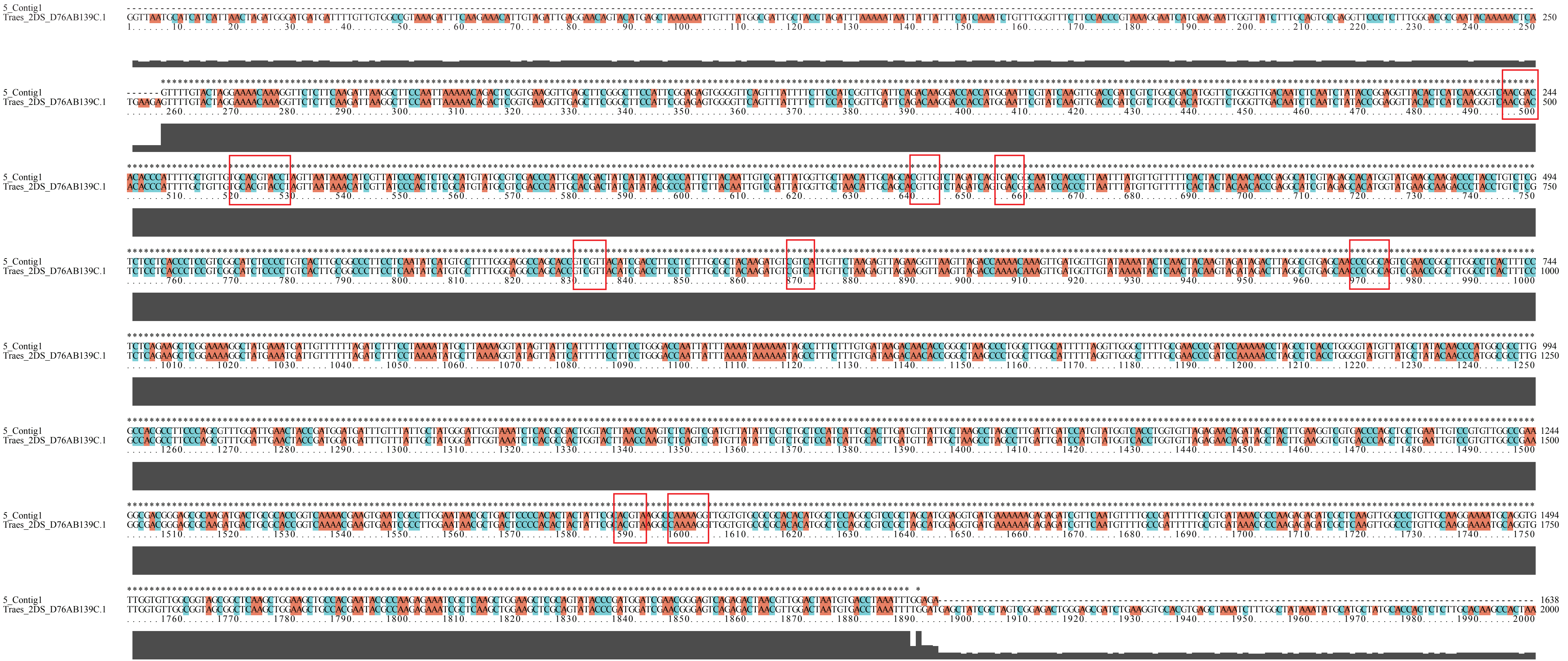

upstream of Traes_2DS_2CCCA54C1.1 (+, 5'->3') and its sequencing data

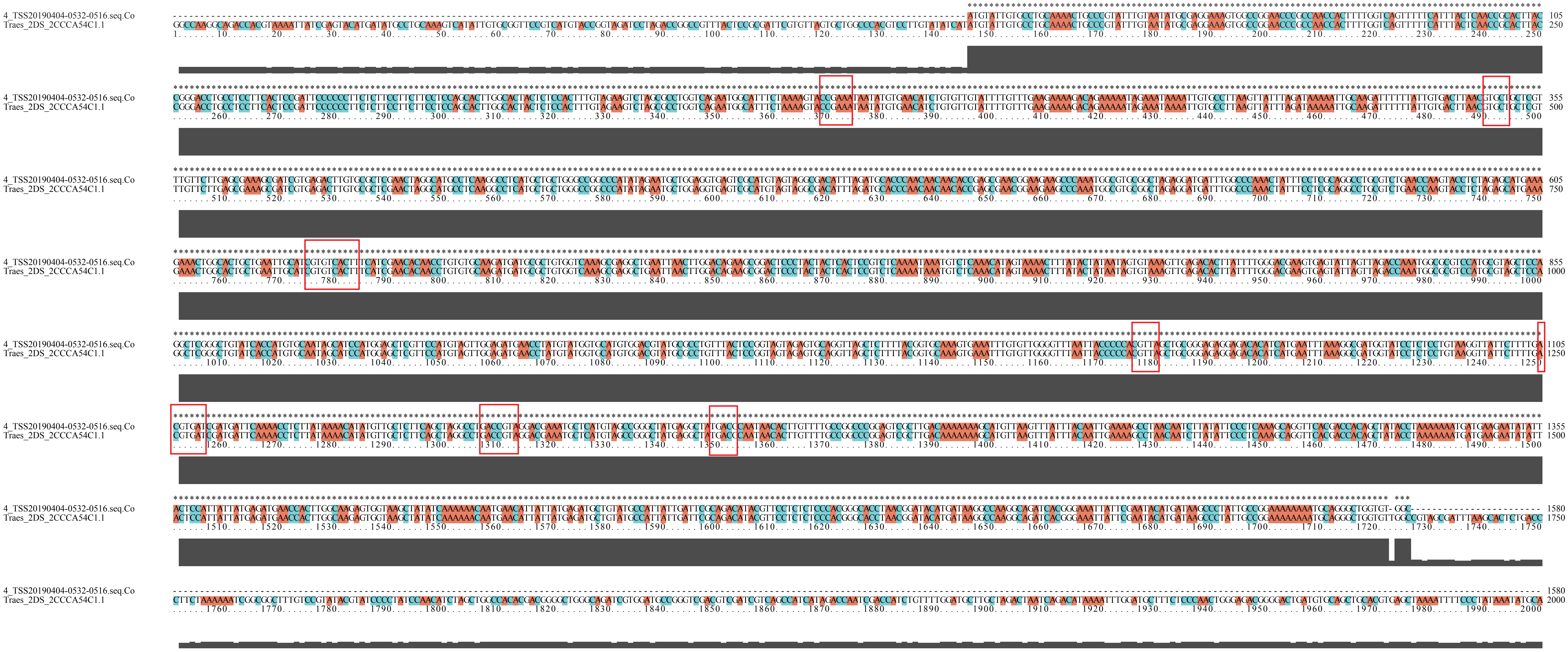

upstream of Traes_2BS_40C683B47.1 (+, 5'->3') and its sequencing data

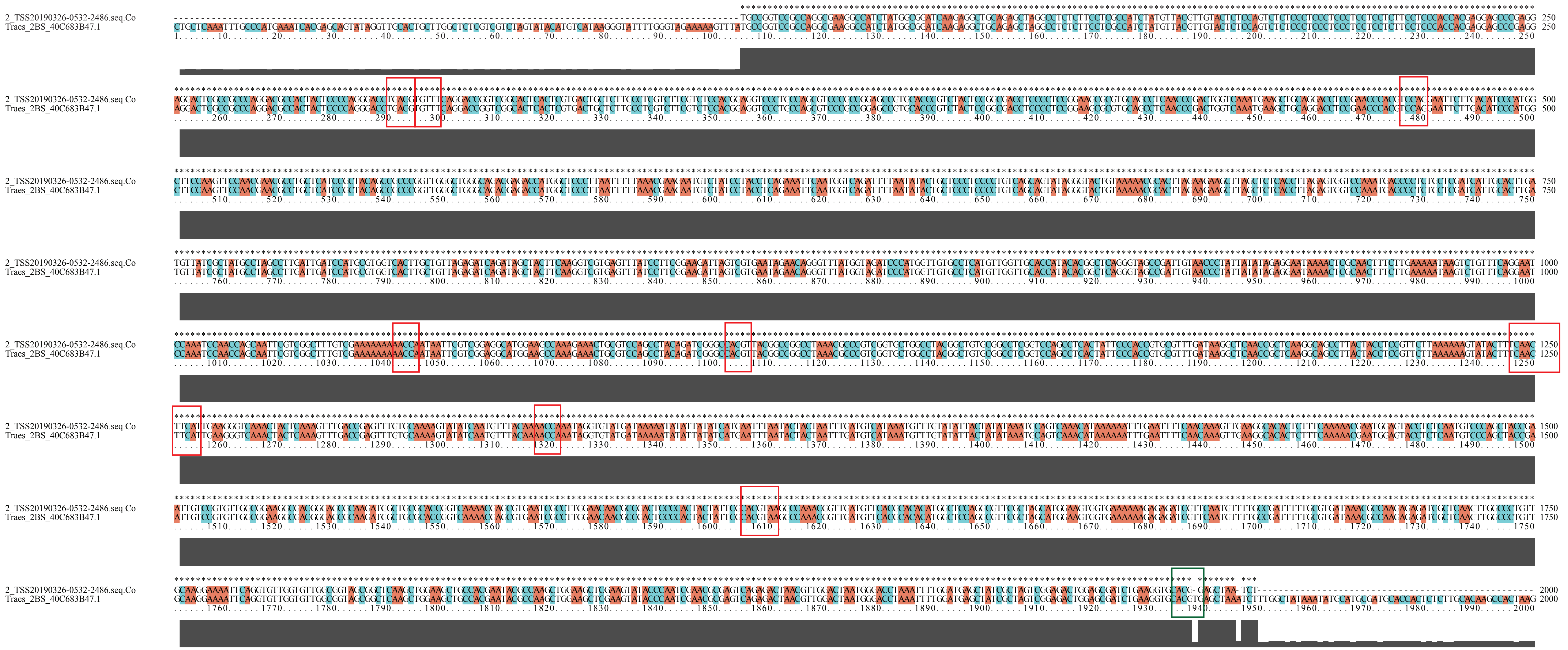

upstream of Traes_6AS_5BAD56BB6.1 (-, 3'->5') and its sequencing data

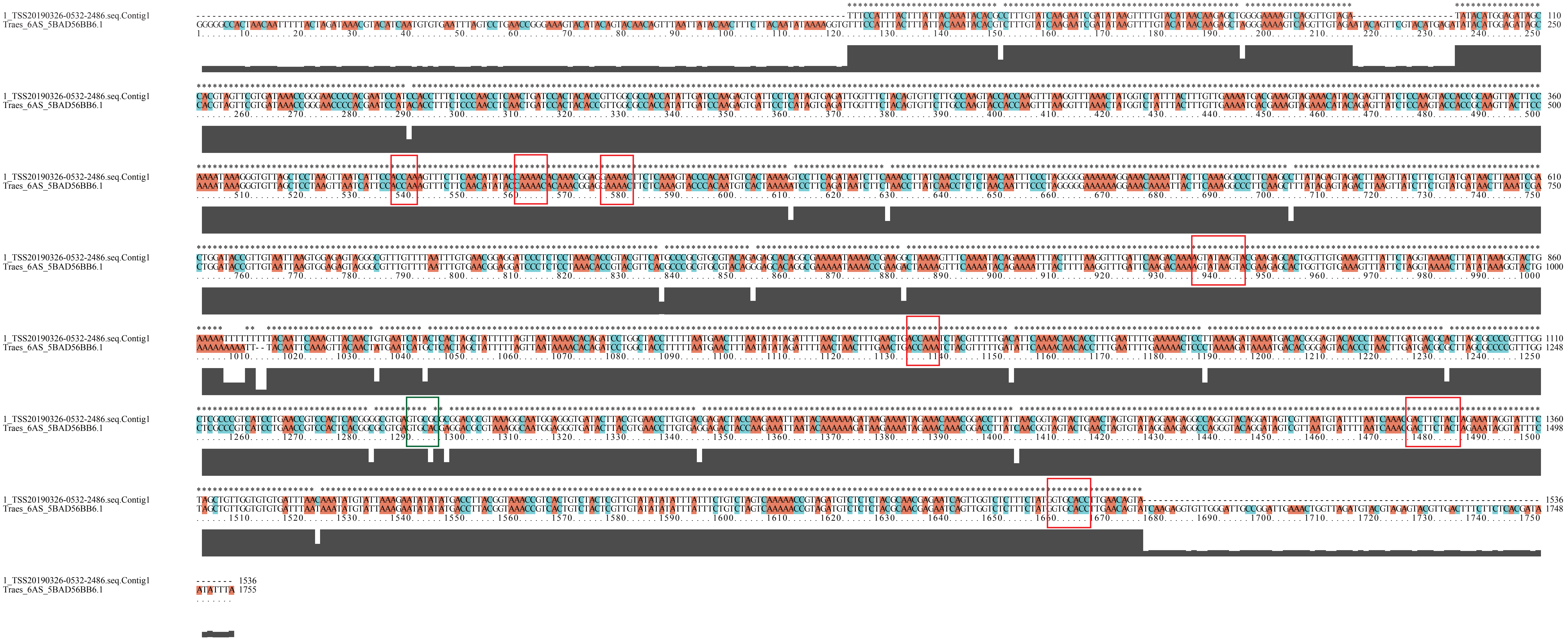

upstream of Traes_2BS_B6EBC0962.1   (-, 3'->5') and its sequencing data

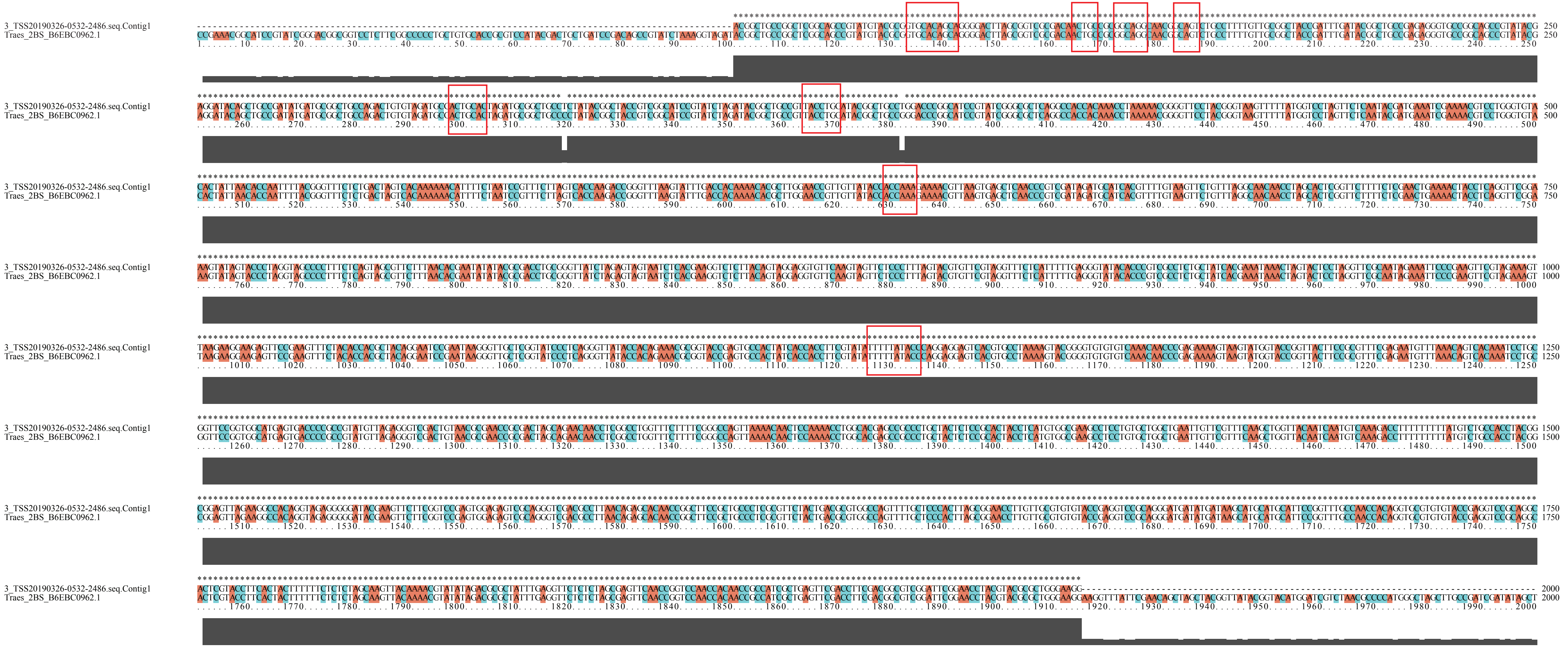

upstream of Traes_1AS_6C84785B3.2 (+, 5'->3') and its sequencing data

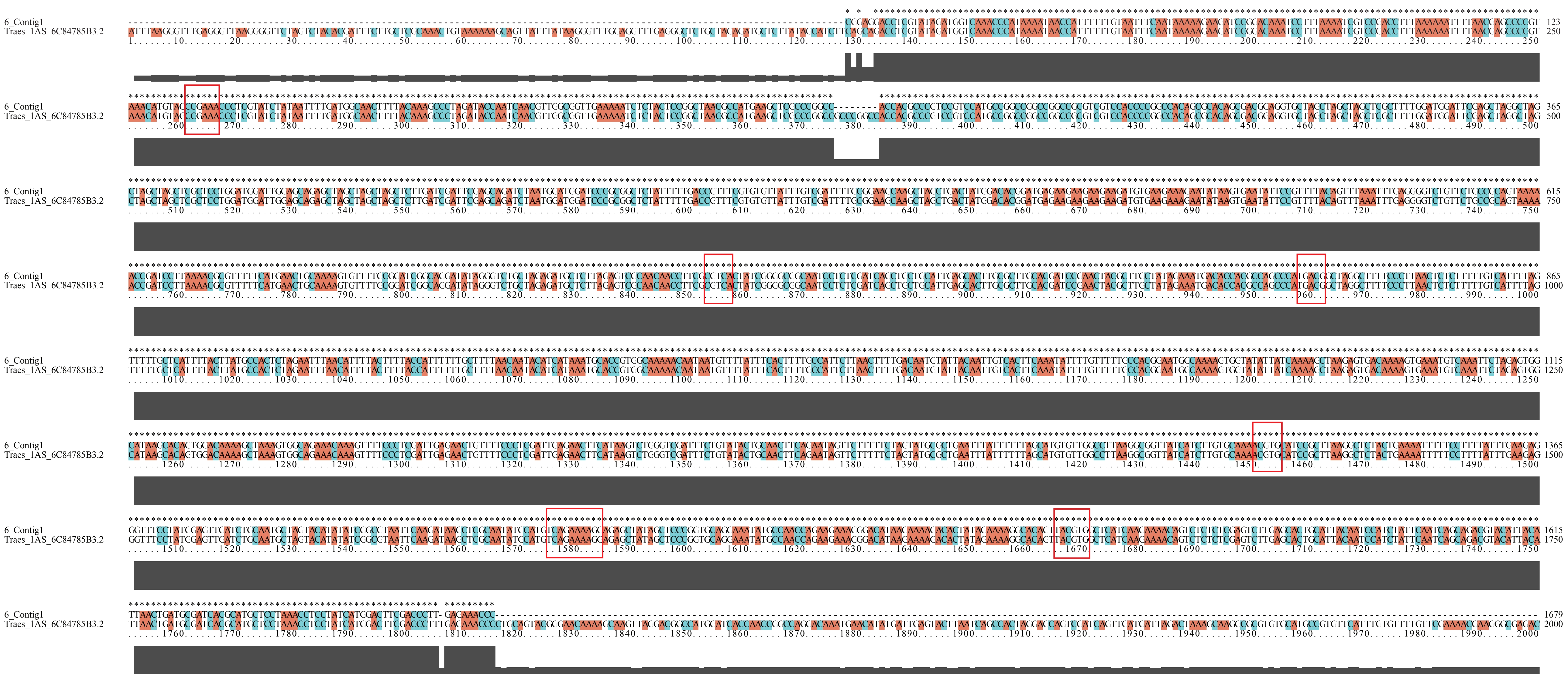

Supplement: Supplementary file 10 — Figure S10. Sequencing data of the predicted c is-elements in upstream. Pair alignments were performed between upstream sequences and sequencing data. Matched cis-acting elements between them are circled by red boxes. Cis-acting elements with tiny differences are circled by green boxes. (PDF 1069 kb) [file 12864_2019_6006_MOESM10_ESM.pdf]
