# Supplementary material for: Genome-wide and evolutionary analysis of the class III peroxidase gene family in wheat and Aegilops tauschii reveals that some members are involved in stress responses
Source: BMC Genomics. 2019 Aug 22;20:666. doi: 10.1186/s12864-019-6006-5 (PMC6704529; doi:10.1186/s12864-019-6006-5)
Supplement: Supplementary file 9 — Figure S9. Upstream sequences and predicted cis-acting elements related to stress and hormone responses in TaePRXs. Various predicted cis-acting elements are circled by different colours. The corresponding information is provided in Additional file 23: Table S11. (PDF 6070 kb) [file 12864_2019_6006_MOESM9_ESM.pdf]

>Traes_2DS_D76AB139C.1

```
+ GTTAATGCAT CATCATTAAC TAGATGGGAT GATGATTTTG TTGTGGCCGT AAAGATTTCA AGAAACATTG
- CAATTACGTA GTAGTAATTG ATCTACCCTA CTACTAAAAC AACACCGGCA TTTCTAAAGT TCTTTGTAAC

+ TAGATTGAGG AACAGTACAT GAGCTAAAAA ATTGTTTATG GCGATTGCTA CCTAGATTTA AAAATAATTA
- ATCTAACTCC TTGTCATGTA CTCGATTTTT TAACAAATAC CGCTAACGAT GGATCTAAAT TTTTATTAAT

+ TTATTTCATC AAATCTGTTT GGGTTTCTTC CACCCGTAAA GGAATCATGA AGAATTGGTT ATCTTTGCAG
- AATAAAGTAG TTTAGACAAA CCCAAAGAAG GTGGGCATTT CCTTAGTACT TCTTAACCAA TAGAAACGTC

+ TGCGAGGTTC CCTCTTTGGG ACGCGAATAC AAAAACTCAT GAAGAGTTTT GTACTAGGAA AACAAAGGTT
- ACGCTCCAAG GGAGAAACCC TGCGCTTATG TTTTTGAGTA CTTCTCAAAA CATGATCCTT TTGTTTCCAA

+ CTCTTCAAGA TTAAGGCTTC CAATTAAAAA CAGACTCGGT GAAGGTTGAG CTTCGGGCTT CCATTCGGAG
- GAGAAGTTCT AATTCCGAAG GTTAATTTTT GTCTGAGCCA CTTCCAACTC GAAGCCCGAA GGTAAGCCTC

+ AGTGGGGTTC AGTTTATTTT CTTCCATCGG TTGATTCAGA CAAGGACCAC CATGGAATTC GTATCAAGTT
- TCACCCCAAG TCAAATAAAA GAAGGTAGCC AACTAAGTCT GTTCCTGGTG GTACCTTAAG CATAGTTCAA

+ GACCGATCGT CTGGCGACAT GGTTCTGGGT TGACAATCTC AATCTATACC GGAGGTTACA CTCATCAAGG
- CTGGCTAGCA GACCGCTGTA CCAAGACCCA ACTGTTAGAG TTAGATATGG CCTCCAATGT GAGTAGTTCC

+ GTCAACGACA CACCCATTTT GCTGTTGTGC ACGTACCTAG TTAATAAACA TCGTTATCCC ACTCTCGCAT
- CAGTTGCTGT GTGGGTAAAA CGACAACACG TGCATGGATC AATTATTTGT AGCAATAGGG TGAGAGCGTA

+ GTATGCGTCG ACCCATTGCA CGACTATCAT ATACGCCCAT TCTTACAATT GTCGATTATG GTTGCTAACA
- CATACGCAGC TGGGTAACGT GCTGATAGTA TATGCGGGTA AGAATGTTAA CAGCTAATAC CAACGATTGT

+ TTGCAGCACG TTGTCTAGAT CAGTGACGGC AATCCACCCT TAATTTATGT TGTTTTTCAC TACTACAACA
- AACGTCGTGC AACAGATCTA GTCACTGCCG TTAGGTGGGA ATTAAATACA ACAAAAAGTG ATGATGTTGT

+ CCGAGGCATC GTAGAGCACA TGGTATGAAG CAAGACCCTA CCTGTCTCGT CTCCTCACCC TCCGTCGGCA
- GGCTCCGTAG CATCTCGTGT ACCATACTTC GTTCTGGGAT GGACAGAGCA GAGGAGTGGG AGGCAGCCGT

+ TCTCCCCTGT CACTTGCGGC CCTTCCTCAA TATCATGTGC TTTTGGGAGG CCAGCACCGT CGTTACATCG
- AGAGGGGACA GTGAACGCCG GGAAGGAGTT ATAGTACACG AAAACCCTCC GGTCGTGGCA GCAATGTAGC

+ ACCTTCCTCT TTGCGCTACA AGATGTCGTC ATTGTTCTAA GAGTTAGAAG GTTAAGTTAG ACCAAAACAA
- TGGAAGGAGA AACGCGATGT TCTACAGCAG TAACAAGATT CTCAATCTTC CAATTCAATC TGGTTTTGTT

+ AGTTGATGGT TGTATAAAAT ACTCAACTAC AAGTAGATAG ACTTAGGCGT GAGCAACCCG GCAGTCGAAC
- TCAACTACCA ACATATTTTA TGAGTTGATG TTCATCTATC TGAATCCGCA CTCGTTGGGC CGTCAGCTTG

+ CGGCTTGGCC TCACTTTCCT CTCAGAAGCT CGGAAAAGGC TATGAAATGA TTGTTTTTTA GATCTTTCCT
- GCCGAACCGG AGTGAAAGGA GAGTCTTCGA GCCTTTTCCG ATACTTTACT AACAAAAAAT CTAGAAAGGA

+ AAAATATGCT TAAAAGGTAT AGTTATTCAT TTTTCCTTCC TGGGACCAAT TATTTAAAAT AAAAATATAGC
- TTTTATACGA ATTTTCCATA TCAATAAGTA AAAAGGAAGG ACCCTGGTTA ATAAATTTTA TTTTTATCG

+ CTTTCTTTGT GATAAGACAA CACCGGGCTA AGCCCTGGCT TGGCATTTTT AGGTTGGGCT TTTGCGAACC
- GAAAGAAACA CTATTCTGTT GTGGCCCGAT TCGGGACCGA ACCGTAAAAA TCCAACCCGA AAACGCTTGG

+ CGATCCAAAA ACCTAGCCTC ACCTGGGGTA TGTTATGCTA TACAACCCAT GGCGCCTTGG CCACGCCTTC
- GCTAGGTTTT TGGATCGGAG TGGACCCCAT ACAATACGAT ATGTTGGGTA CCGCGGAACC GGTGCGGAAG

+ CCAGCGTTTG GATTGAACTA CCGATGGATG ATTTGTTTAT TGCTATGGGA TTGGTAAATC TCACGCGACT
- GGTCGCAAAC CTAACTTGAT GGCTACCTAC TAAACAAATA ACGATACCCT AACCATTTAG AGTGCGCTGA

+ GGTACTTAAC CAAGTCTCAG TCGATGTTAT ATTCGTCTGC TCCATCATTG CACTTGATGT TATTGCTAAG
- CCATGAATTG GTTCAGAGTC AGCTACAATA TAAGCAGACG AGGTAGTAAC GTGAACTACA ATAACGATTC

+ CCTAGCCTTG ATTGATCCAT GTATGGTCAC CTGGTGTTAG AGAACAGATA GCTACTTGAA GGTCGTGACC
- GGATCGGAAC TAACTAGGTA CATACCAGTG GACCACAATC TCTTGTCTAT CGATGAACTT CCAGCACTGG

+ CAGCTGCTGA ATTGTCCGTG TTGGCCGAAG GCGACGGGAG CGCAAGATGA CTGCGCACCG GTCAAAACGA
- GTCGACGACT TAACAGGCAC AACCGGCTTC CGCTGCCCTC GCGTTCTACT GACGCGTGGC CAGTTTTGCT

+ AGTGAATCGC CTTGGAATAA CGCTGACTCC CCACACTACT ATTCGCACGT AAGGCCAAAA GGTTGGTGTG
- TCACTTAGCG GAACCTTATT GCGACTGAGG GGTGTGATGA TAAGCGTGCA TTCCGGTTTT CCAACCACAC

+ CGCGCACACA TGGCTCCAGG CGTCCGCTAG CATGGAGGTG ATGAAAAAAG AGAGATCGTT CAATGTTTTG
- GCGCGTGTGT ACCGAGGTCC GCAGGCGATC GTACCTCCAC TACTTTTTTC TCTCTAGCAA GTTACAAAAC

+ CCGATTTTTG CGTGATAAAC GCCAAGAGAG ATCGCTCAAG TTGGCCCTGT TGCAAGGAAA ATGCAGGTGT
- GGCTAAAAAC GCACTATTTG CGGTTCTCTC TAGCGAGTTC AACCGGGACA ACGTTCCTTT TACGTCCACA

+ TGGTGTTGGC GGTAGCGGCT CAAGCTGGAA GCTGCCACGA ATACGCCAAG AGAAATCGCT CAAGCTGGAA
- ACCACAACCG CCATCGCCGA GTTCGACCTT CGACGGTGCT TATGCGGTTC TCTTTAGCGA GTTCGACCTT

+ GCTCGCAGTA TACCCGATGG ATCGAACGGG AGTCAGAGAC TAACGTTGGA CTAATGTGAC CTAAATTTTG
- CGAGCGTCAT ATGGGCTACC TAGCTTGCCC TCAGTCTCTG ATTGCAACCT GATTACACTG GATTTAAAAC

+ GATGAGCTAT CGCTAGTCGG AGACTGGGAG CGATCTGAAG GTGCACGTGA GCTAAATCTT TGGCTATAAA
- CTACTCGATA GCGATCAGCC TCTGACCCTC GCTAGACTTC CACGTGCACT CGATTTAGAA ACCGATATTT

+ TATGCATGCT ATGCACCACT CTCTTGCACA AGCCACTA
- ATACGTACGA TACGTGGTGA GAGAACGTGT TCGGTGAT
```

Motifs Found

+
+ ABRE

Site Name	Organism	Position	Strand	Matrix score.	sequence	function
ABRE	Hordeum vulgare	518	-	9	CGTACGTGCA	cis-acting element involved in the abscisic acid responsivene
ABRE	Arabidopsis thaliana	1586	-	5	ACGTG	cis-acting element involved in the abscisic acid responsivene
ABRE	Arabidopsis thaliana	637	-	5	ACGTG	cis-acting element involved in the abscisic acid responsivene
ABRE	Arabidopsis thaliana	1935	+	5	ACGTG	cis-acting element involved in the abscisic acid responsivene
ABRE	Arabidopsis thaliana	520	-	5	ACGTG	cis-acting element involved in the abscisic acid responsivene
ABRE	Arabidopsis thaliana	1934	-	6	CACGTG	cis-acting element involved in the abscisic acid responsivene
ABRE	Arabidopsis thaliana	965	+	7	AACCCGG	cis-acting element involved in the abscisic acid responsivene

+ ABRE3a

Site Name	Organism	Position	Strand	Matrix score.	sequence	function
ABRE3a	Zea mays	520	-	6	TACGTG	
ABRE3a	Zea mays	1586	-	6	TACGTG	

+ ABRE4

Site Name	Organism	Position	Strand	Matrix score.	sequence	function
ABRE4	Zea mays	520	+	6	CACGTA	
ABRE4	Zea mays	1586	+	6	CACGTA	

+ AT-rich sequence
+ CAAT-box
+ CAT-box
+ CGTCA-motif

Site Name	Organism	Position	Strand	Matrix score.	sequence	function
CGTCA-motif	Hordeum vulgare	654	-	5	CGTCA	cis-acting regulatory element involved in the MeJA-responsiver
CGTCA-motif	Hordeum vulgare	867	+	5	CGTCA	cis-acting regulatory element involved in the MeJA-responsiver

+ DRE core
+ ERE
+ G-Box
+ G-box
+ GT1-motif
+ MRE
+ MYB
+ MYB-like sequence
+ MYC
+ Myb-binding site
+ P-box

Site Name	Organism	Position	Strand	Matrix score.	sequence	function
P-box	Oryza sativa	1596	-	7	CCTTTTG	gibberellin-responsive element

+ STRE
+ TATA
+ TATA-box
+ TATC-box
+ TC-rich repeats
+ TCT-motif
+ TGA-element

Site Name	Organism	Position	Strand	Matrix score.	sequence	function
TGA-element	Brassica oleracea	494	+	6	AACGAC	auxin-responsive element
TGA-element	Brassica oleracea	829	-	6	AACGAC	auxin-responsive element

+ TGACG-motif

Site Name	Organism	Position	Strand	Matrix score.	sequence	function
TGACG-motif	Hordeum vulgare	654	+	5	TGACG	cis-acting regulatory element involved in the MeJA-responsiver
TGACG-motif	Hordeum vulgare	867	-	5	TGACG	cis-acting regulatory element involved in the MeJA-responsiver

+ Unnamed__1
+ Unnamed__2
+ Unnamed__4
+ W box
+ as-1
+ [illegible]

\>Traes_2DS_2CCCA54C1.1

```
+ GCCAAGGCAG ACCACGTAAA ATTATCGAGT ACATGATATG CCTGCAAAGT CATATTGTGC GGTTCCGTCA
- CGGTTCCGTC TGGTGCATTT TAATAGCTCA TGTACTATAC GGACGTTTCA GTATAACACG CCAAGGCAGT

+ TGTACCGGTA GATCCTAGAC CGGCCGTTTA CTCCGCGATT CGTGTTAGTG CTGGCCCACG TCCTTGTATA
- ACATGGCCAT CTAGGATCTG GCCGGCAAAT GAGGCGCTAA GCACAATCAC GACCGGGTGC AGGAACATAT

+ TCATATGTAT TGTGCCTGCA AAACTGCCCG TATTTGTAAT ATGCGAGGAA AGTGGCCGGA ACCCGCCAAC
- AGTATACATA ACACGGACGT TTTGACGGGC ATAAACATTA TACGCTCCTT TCACCGGCCT TGGGCGGTTG

+ CACTTTTGGT CAGTTTTTCA TTTACTCAAC CGCACTTACC GGGACCTGCC TCCTTCACTC CGATTCCCCC
- GTGAAAACCA GTCAAAAAGT AAATGAGTTG GCGTGAATGG CCCTGGACGG AGGAAGTGAG GCTAAGGGGG

+ CTTCTCTTCC TTCTTCCTCC AGCACTTGGC ACTACTCTCC ACTTTGTAGA AGTCTAGCGC CTGGTCAGAA
- GAAGAGAAGG AAGAAGGAGG TCGTGAACCG TGATGAGAGG TGAAACATCT TCAGATCGCG GACCAGTCTT

+ TGGCATTTCT AAAAGTACCG AAATAATATG TGAACATCTG TGTTGTATTT TGTTTGAAGA AAAGACAGAA
- ACCGTAAAGA TTTTCATGGC TTTATTATAC ACTTGTAGAC ACAACATAAA ACAAACTTCT TTTCTGTCTT

+ AAATAGAAAT AAAATTGTGC CTTAAGTTAT TTAGATAAAA ATTGCAAGAT TTTTTATTGT GACTTAACGT
- TTTATCTTTA TTTTAACACG GAATTCAATA AATCTATTTT TAACGTTCTA AAAAATAACA CTGAATTGCA

+ GCTGCTCGTT TGTTCTTGAG CGAAAGCGAT CGTGAGACTT GTGCGCTCGA ACTAGGCATG CCTCAAGGCC
- CGACGAGCAA ACAAGAACTC GCTTTCGCTA GCACTCTGAA CACGCGAGCT TGATCCGTAC GGAGTTCCGG

+ TCATGCTGCT GGGCCGGCCC ATATAGAATG CTGGAGGTGA GTCGCATGTA GTAGGCGACA TTTAGATGCA
- AGTACGACGA CCCGGCCGGG TATATCTTAC GACCTCCACT CAGCGTACAT CATCCGCTGT AAATCTACGT

+ CCCAACAACA ACACCGAGCG AACGGAAGAA GCCCAAATGG CGTGCGGCTA GAGGATGATT TGGCCCAAAC
- GGGTTGTTGT TGTGGCTCGC TTGCCTTCTT CGGGTTTACC GCACGCCGAT CTCCTACTAA ACCGGGTTTG

+ TATTTCCTCG CAGGCCTGCG TCTGAACCAA GTACCTCTAG AGCATGAAAG AAACTGGCAC TGCTGAATTG
- ATAAAGGAGC GTCCGGACGC AGACTTGGTT CATGGAGATC TCGTACTTTC TTTGACCGTG ACGACTTAAC

+ CATCGTGTCA CTTTCATCGA ACACAACCTG TGTGCAAGAT GATGCGCTGT GGTCAAAGCG AGGCTGAATT
- GTAGCACAGT GAAAGTAGCT TGTGTTGGAC ACACGTTCTA CTACGCGACA CCAGTTTCGC TCCGACTTAA

+ AACTTGGACA GAAGCGGACT CCCTACTACT CACTCCGTCT CAAAATAAAT GTCTCAAACA TAGTAAAACT
- TTGAACCTGT CTTCGCCTGA GGGATGATGA GTGAGGCAGA GTTTTATTTA CAGAGTTTGT ATCATTTTGA

+ TTATACTATA ATAGTGTAAA GTTGAGACAC TTATTTTGGG ACGAAGTGAG TATTAGTTAG ACCAAATGGC
- AATATGATAT TATCACATTT CAACTCTGTG AATAAAACCC TGCTTCACTC ATAATCAATC TGGTTTACCG

+ GCGTCCATGC GTAGCTCCAG GCTCGGGCTG TATCACCATG TGCAATAGCA TCCATGGAGC TCGTTCCATG
- CGCAGGTACG CATCGAGGTC CGAGCCCGAC ATAGTGGTAC ACGTTATCGT AGGTACCTCG AGCAAGGTAC

+ TAGTTGGAGA TGAACCTATG TATGGTGCAT GTGGACGTAT GCGCCTGTTT ACTCCGGTAG TAGAGTGCAG
- ATCAACCTCT ACTTGGATAC ATACCACGTA CACCTGCATA CGCGGACAAA TGAGGCCATC ATCTCACGTC

+ GTTAGCTCTT TTACGGTGCA AAGTGAAATT TGTGTTGGGG TTTAATTACC CCCACGTTAG CTGCGGGAGA
- CAATCGAGAA AATGCCACGT TTCACTTTAA ACACAACCCC AAATTAATGG GGGTGCAATC GACGCCCTCT

+ GGAGACACAT CATGAATTTA AAGGCGATGG TATCCTCTCC TGTAAGGTTA TTCTTTTGAC GTGATCGATG
- CCTCTGTGTA GTACTTAAAT TTCCGCTACC ATAGGAGAGG ACATTCCAAT AAGAAAACTG CACTAGCTAC

+ ATTCAAAACC TCTTATAAAA CATATGTTGC TCTTCAGCTA GGCCTGACCG TAGGACGAAA TGCTCATGTA
- TAAGTTTTGG AGAATATTTT GTATACAACG AGAAGTCGAT CCGGACTGGC ATCCTGCTTT ACGAGTACAT

+ GCCGGGCTAT GAGGCTATGA CGCAATAACA CTTGTTTTGC CGGCCCGGAG TCGCTTGACA AAAAAAGCAT
- CGGCCCGATA CTCCGATACT GCGTTATTGT GAACAAAACG GCCGGGCCTC AGCGAACTGT TTTTTTCGTA

+ GTTAAGTTTA TTTACAATTG AAAAGCCTAA CAATCTTATA TTCCCTCAAA GCAGGTTCAC GACCACAGCT
- CAATTCAAAT AAATGTTAAC TTTTCGGATT GTTAGAATAT AAGGGAGTTT CGTCCAAGTG CTGGTGTCGA

+ ATACCTAAAA AAATGATGAA GAATATATTA CTCCATTATT ATGAGATGAA CCACTTGGCA AGAGTGGTAA
- TATGGATTTT TTTACTACTT CTTATATAAT GAGGTAATAA TACTCTACTT GGTGAACCGT TCTCACCATT

+ GCTATATCAA AAAACAATGA ACATTATTAT GAGATGCTGT ATGCCATTAT TGATTCGCAG ACATACGTTC
- CGATATAGTT TTTTGTTACT TGTAATAATA CTCTACGACA TACGGTAATA ACTAAGCGTC TGTATGCAAG

+ CTCTCTCCCA CGGGCACCTA ACGGATACAT GATAAGGCCA AGGCAGATCA CGGGAAATTA TTCGAATACA
- GAGAGAGGGT GCCCGTGGAT TGCCTATGTA CTATTCCGGT TCCGTCTAGT GCCCTTTAAT AAGCTTATGT

+ TGATAAGCCC TATTGCCGGA AAAAAAATGC AGGGCTGGTG TTGGCCGTAG CGATTTAAGC ACTCTGACCC
- ACTATTCGGG ATAACGGCCT TTTTTTTACG TCCCGACCAC AACCGGCATC GCTAAATTCG TGAGACTGGG

+ TTCTAAAAAA TCGGCGGCTT TGTCCGTATA CGTATCCCCT ATCCAACATC TAGCTGGCCA CACGACGGGG
- AAGATTTTTT AGCCGCCGAA ACAGGCATAT GCATAGGGGA TAGGTTGTAG ATCGACCGGT GTGCTGCCCC

+ CTGGGCAGAT CGTGGATGCC GGGTCGACGT CGATCGTCAG CCATCATAGA CCAATCGACC ATCTGTTTTG
- GACCCGTCTA GCACCTACGG CCCAGCTGCA GCTAGCAGTC GGTAGTATCT GGTTAGCTGG TAGACAAAAC

+ GATGCTTGCT AGACTAATCA GACATAAAAT TTGGATGCTT TCTCCCAACT GGGAGACGGG GACTGATGTG
- CTACGAACGA TCTGATTAGT CTGTATTTTA AACCTACGAA AGAGGGTTGA CCCTCTGCCC CTGACTACAC

+ CAGCTGCACG TGAGCTAAAA TTTTCCCTAT AAATATGC
- GTCGACGTGC ACTCGATTTT AAAAGGGATA TTTATACG
```

Motifs Found

- \+
- \+ AAGAA-motif
- \+ ABRE

Site Name	Organism	Position	Strand	Matrix score.	sequence	function
ABRE	Arabidopsis thaliana	13	-	5	ACGTG	cis-acting element involved in the abscisic acid responsivene
ABRE	Arabidopsis thaliana	1306	-	7	TACGGTC	cis-acting element involved in the abscisic acid responsivene
ABRE	Arabidopsis thaliana	1173	-	5	ACGTG	cis-acting element involved in the abscisic acid responsivene
ABRE	Arabidopsis thaliana	1968	+	5	ACGTG	cis-acting element involved in the abscisic acid responsivene
ABRE	Arabidopsis thaliana	487	+	5	ACGTG	cis-acting element involved in the abscisic acid responsivene
ABRE	Arabidopsis thaliana	1967	-	6	CACGTG	cis-acting element involved in the abscisic acid responsivene
ABRE	Arabidopsis thaliana	1249	+	5	ACGTG	cis-acting element involved in the abscisic acid responsivene
ABRE	Arabidopsis thaliana	127	-	5	ACGTG	cis-acting element involved in the abscisic acid responsivene
ABRE	Hordeum vulgare	770	+	9	GCAACGTGTC	cis-acting element involved in the abscisic acid responsivene

- \+ ABRE3a

Site Name	Organism	Position	Strand	Matrix score.	sequence	function
ABRE3a	Zea mays	13	-	6	TACGTG	

- \+ ABRE4

Site Name	Organism	Position	Strand	Matrix score.	sequence	function
ABRE4	Zea mays	13	+	6	CACGTA	

- \+ AT-rich element
- \+ CAAT-box
- \+ CAT-box
- \+ CGTCA-motif

Site Name	Organism	Position	Strand	Matrix score.	sequence	function
CGTCA-motif	Hordeum vulgare	66	+	5	CGTCA	cis-acting regulatory element involved in the MeJA-responsiven
CGTCA-motif	Hordeum vulgare	1348	-	5	CGTCA	cis-acting regulatory element involved in the MeJA-responsiven
CGTCA-motif	Hordeum vulgare	1247	-	5	CGTCA	cis-acting regulatory element involved in the MeJA-responsiven
CGTCA-motif	Hordeum vulgare	1855	+	5	CGTCA	cis-acting regulatory element involved in the MeJA-responsiven

- \+ G-Box
- \+ G-box
- \+ GATA-motif
- \+ GTGGC-motif
- \+ I-box
- \+ LTR

Site Name	Organism	Position	Strand	Matrix score.	sequence	function
LTR	Hordeum vulgare	368	+	6	CCGAAA	cis-acting element involved in low-temperature responsivene

- \+ MBS

Site Name	Organism	Position	Strand	Matrix score.	sequence	function
MBS	Arabidopsis thaliana	1936	+	6	CAACTG	MYB binding site involved in drought-inducibility

- \+ MSA-like
- \+ MYB
- \+ MYC
- \+ Myb
- \+ O2-site
- \+ STRE
- \+ TATA-box
- \+ TCA-element

Site Name	Organism	Position	Strand	Matrix score.	sequence	function
TCA-element	Nicotiana tabacum	1879	+	9	CCATCTTTTT	cis-acting element involved in salicylic acid responsivenes

- \+ TGACG-motif

Site Name	Organism	Position	Strand	Matrix score.	sequence	function
TGACG-motif	Hordeum vulgare	66	-	5	TGACG	cis-acting regulatory element involved in the MeJA-responsiven
TGACG-motif	Hordeum vulgare	1348	+	5	TGACG	cis-acting regulatory element involved in the MeJA-responsiven
TGACG-motif	Hordeum vulgare	1247	+	5	TGACG	cis-acting regulatory element involved in the MeJA-responsiven
TGACG-motif	Hordeum vulgare	1855	-	5	TGACG	cis-acting regulatory element involved in the MeJA-responsiven

- \+ Unnamed__1
- \+ Unnamed__4
- \+ W box

>Traes_5BL_3ED1B0234.2

```
+ ACTTTGCTTT TGTCTCTATA TCTGAACCCA AGAAAGTCGA AGAAGCCTTC ATGGAACCTG AATGGACTCA
- TGAAACGAAA ACAGAGATAT AGACTTGGGT TCTTTCAGCT TCTTCGGAAG TACCTTGGAC TTACCTGAGT

+ AGCTATGCAA GAAGAGCTTC AATAGTTTGA CCTGAATAAT GTCTGGGAGC TTGTCAAACG TCCTGATCCT
- TCGATACGTT CTTCTCGAAG TTATCAAACT GGACTTATTA CAGACCCTCG AACAGTTTGC AGGACTAGGA

+ TGCAAGCACA ATGGCATTGG CACGAAATGG ATCTATCGCA ACAAGCAAGA TGAACTTGGA CAAGTTGTCA
- ACGTTCGTGT TACCGTAACC GTGCTTTACC TAGATAGCGT TGTTCGTTCT ACTTGAACCT GTTCAACAGT

+ GAAGCAGAGC TCGCCTTGTT GCTCAAGGTT ACACACAAGT GGAAGGAATA GACTTCGCTG AAACCTTTGC
- CTTCGTCTCG AGCGGAACAA CGAGTTCCAA TGTGTGTTCA CCTTCCTTAT CTGAAGCGAC TTTGGAAACG

+ TCCTGTGGCT CGGCTTGAAG CCATTCGCAT ACTACTGGCT TATGCAAACC ATCACAACAT TCTTCTGTAT
- AGGACACCGA GCCGAACTTC GGTAAGCGTA TGATGACCGA ATACGTTTGG TAGTGTTGTA AGAAGACATA

+ CAAATGGACG TGAAGAGTGC CTTTTTCAAT GGCAAAATCG AAGAAGTGTA TGTCACTCAA CAGCCTGGTT
- GTTTACCTGC ACTTCTCACG GAAAAAGTTA CCGTTTTAGC TTCTTCACAT ACAGTGAGTT GTCGGACCAA

+ TTGAAGATCC TAAGCATCCC GATATGGTGT ACAAGCTCAA CAAGGCATTG CATGGCCTCC AACAAGCACC
- AACTTCTAGG ATTCGTAGGG CTATACCACA TGTTCGAGTT GTTCCGTAAC GTACCGGAGG TTGTTCGTGG

+ TCGGTCTTGG TATGACAGAC TGAAGGAATT CCTGAAGAGC AAAGGCTTCA AACCTGGTTC ACTAGATCCC
- AGCCAGAACC ATACTGTCTG ACTTCCTTAA GGACTTCTCG TTTCCGAAGT TTGGACCAAG TGATCTAGGG

+ ATTCTCTTCA CGAATACATA TGATGGTGAA CTATTTATGT GTCAAATATA CGTGGATGAC ATTATCTTTG
- TAAGAGAAGT GCTTATGTAT ACTACCACTT GATAAATACA CAGTTTATAT GCACCTACTG TAATAGAAAC

+ GATGCACTGG TAAGAGATAC AGTGACGAGT TTGGATACAT GATGCAAGAG CAATATCAGA TGTCCATGAT
- CTACGTGACC ATTCTCTATG TCACTGCTCA AACCTATGTA CTACGTTCTC GTTATAGTCT ACAGGTACTA

+ GGAGGAGCTG AAATTCTTCC TTGGTCTTCA AATCCGTCAA CAACGCAATG TCATCTTCAT ATCGCAAGAA
- CCTCCTCGAC TTTAAGAAGG AACCAGAAGT TTAGGCAGTT GTTGCGTTAC AGTAGAAGTA TAGCGTTCTT

+ AAATACCTTA AAGATTGCCT GAAAAAGTTT GGAATGCAAG ACTGCAAAGG GTACATGATG CCAATGCCAA
- TTTATGGAAT TTCTAACGGA CTTTTTCAAA CCTTACGTTC TGACGTTTCC CATGTACTAC GGTTACGGTT

+ CCAAAAGTCA TCTCGGACCT GACACCAATG GTAAAGAGTT CGATCAAAAG GTATACTGCT CCATGATTGG
- GGTTTTCAGT AGAGCCTGGA CTGTGGTTAC CATTTCTCAA GCTAGTTTTC CATATGACGA GGTACTAACC

+ TTATTTGCTC TATTTATGTG CATCTAGGCC TGATATTATG CTTAGCATTT GCATGTGTGC CCAATTCCAA
- AATAAACGAG ATAAATACAC GTAGATCCGG ACTATAATAC GAATCGTAAA CGTACACACG GGTTAAGGTT

+ GCGGCACCAA AGGAGTCACA TCACCTAGCG GTGAAGCGAA TACTTAGATA TTTGGCTCAC ACCCCAACAC
- CGCCGTGGTT TCCTCAGTGT AGTGGATCGC CACTTCGCTT ATGAATCTAT AAACCGAGTG TGGGGTTGTG

+ TAGGATTATG GTATCCAAAN NNNNNNNNNN NNNNNNNNNN NNNNNNNNNN NNNNNNNNNN NNNNNNNNNN
- ATCCTAATAC CATAGGTTTN NNNNNNNNNN NNNNNNNNNN NNNNNNNNNN NNNNNNNNNN NNNNNNNNNN

+ NNNNNNNNNN NNNNNNNNNN NNNNNNNNNN NNNNNNNNNN NNNNNNNNNN GCTGAACGTG TGCTGAACTC
- NNNNNNNNNN NNNNNNNNNN NNNNNNNNNN NNNNNNNNNN NNNNNNNNNN CGACTTGCAC ACGACTTGAG

+ GGAGGTGCCG TACGTTCGAT ACTTGGATCG GTCGGATCGT GAAGACGTAC AACTATATCA ACCGCGTTGT
- CCTCCACGGC ATGCAAGCTA TGAACCTAGC CAGCCTAGCA CTTCTGCATG TTGATATAGT TGGCGCAACA

+ CATAACGCTT CCCCTTACGG TCTACGAGGG TACATGGACA ATACTCTCCC CTCTCGTTGC TATGCATCAC
- GTATTGCGAA GGGGAATGCC AGATGCTCCC ATGTACCTGT TATGAGAGGG GAGAGCAACG ATACGTAGTG

+ CATGATCTTG CGTGTGCGTA GGAATTTTTT TGAAATTACT ACGTTCCCCA ACAATCCCAT CCCATCCACC
- GTACTAGAAC GCACACGCAT CCTTAAAAAA ACTTTAATGA TGCAAGGGGT TGTTAGGGTA GGGTAGGTGG

+ ACGTGCACGG TTGACGGAAG AGCAGGCCTC CAAAACCTCG CCTCTTGAGA TCGTGTATGG GAGAGGTGCG
- TGCACGTGCC AACTGCCTTC TCGTCCGGAG GTTTTGGAGC GGAGAACTCT AGCACATACC CTCTCCACGC

+ ATTAGGTTTT TGAGGAGCGT TCCCGGGTGC CCCTACACCC TCATGAACAG TAAATGAAGT AGACAAACGT
- TAATCCAAAA ACTCCTCGCA AGGGCCCACG GGGATGTGGG AGTACTTGTC ATTTACTTCA TCTGTTTGCA

+ AGATGAAGTA TTGGAAAGGT GAAGGCGATG TTTGTCTACT TCATCTACAT TGTCGTCGCC TTCACCGAGT
- TCTACTTCAT AACCTTTCCA CTTCCGCTAC AAACAGATGA AGTAGATGTA ACAGCAGCGG AAGTGGCTCA

+ ATTGGAAAGG TGTAACAGTT CCCGGGTGCC CCTACACCCT CATGAACAGT AAATTATGTA TTATTTAATT
- TAACCTTTCC ACATTGTCAA GGGCCCACGG GGATGTGGGA GTACTTGTCA TTTAATACAT AATAAATTAA

+ TGATTCATAC TAGTATGTAT AATTCACCAA CTGTCTTCCG TAAAAGAAAC CATTAATCTC AGGTGTTATG
- ACTAAGTATG ATCATACATA TTAAGTGGTT GACAGAAGGC ATTTTCTTTG GTAATTAGAG TCCACAATAC

+ GCATCTCGAT GGAAAAGTAC TTTCCTACTA AGAGCACCTT GTGATGAGAA TATCATCCTT TTTTCCTACT
- CGTAGAGCTA CCTTTTCATG AAAGGATGAT TCTCGTGGAA CACTACTCTT ATAGTAGGAA AAAAGGATGA

+ AACATAAGAG ATGATGAGCA GTCAGCATCA CATGGTAGCT AAGCAGCTTT ATCGTCGCCT GATAGAGAAA
- TTGTATTCTC TACTACTCGT CAGTCGTAGT GTACCATCGA TTCGTCGAAA TAGCAGCGGA CTATCTCTTT

+ AGGCGACGAC GCCCTTTTAC TTAGCCGTGT CGCCCTTTTT TGCTTTCACA GGTTCAAGTC CAAGTGTTCT
- TCCGCTGCTG CGGGAAAATG AATCGGCACA GCGGGAAAAA ACGAAAGTGT CCAAGTTCAG GTTCACAAGA

+ CACTCACCAA AACCTGAAGC AGTGTACGTA CGTGCCCT
- GTGAGTGGTT TTGGACTTCG TCACATGCAT GCACGGGA
```

Motifs Found

+
+ ABRE

Site Name	Organism	Position	Strand	Matrix score.	sequence	function
ABRE	Arabidopsis thaliana	1400	-	6	CACGTG	cis-acting element involved in the abscisic acid responsivene
ABRE	Hordeum vulgare	1983	-	9	CGTACGTGCA	cis-acting element involved in the abscisic acid responsivene
ABRE	Arabidopsis thaliana	610	+	5	ACGTG	cis-acting element involved in the abscisic acid responsivene
ABRE	Hordeum vulgare	1987	+	9	CGTACGTGCA	cis-acting element involved in the abscisic acid responsivene
ABRE	Arabidopsis thaliana	1176	+	5	ACGTG	cis-acting element involved in the abscisic acid responsivene
ABRE	Oryza sativa	1986	-	10	GACACGTACGT	cis-acting element involved in the abscisic acid responsivene
ABRE	Arabidopsis thaliana	1401	+	5	ACGTG	cis-acting element involved in the abscisic acid responsivene
ABRE	Arabidopsis thaliana	1990	+	5	ACGTG	cis-acting element involved in the abscisic acid responsivene
ABRE	Arabidopsis thaliana	358	+	5	ACGTG	cis-acting element involved in the abscisic acid responsivene
ABRE	Arabidopsis thaliana	1276	+	7	TACGGTC	cis-acting element involved in the abscisic acid responsivene

+ ABRE3a

Site Name	Organism	Position	Strand	Matrix score.	sequence	function
ABRE3a	Zea mays	609	+	6	TACGTG	
ABRE3a	Zea mays	1989	+	6	TACGTG	

+ ABRE4

Site Name	Organism	Position	Strand	Matrix score.	sequence	function
ABRE4	Zea mays	609	-	6	CACGTA	
ABRE4	Zea mays	1989	-	6	CACGTA	

+ AC-I
+ ARE

Site Name	Organism	Position	Strand	Matrix score.	sequence	function
ARE	Zea mays	326	+	6	AAACCA	cis-acting regulatory element essential for the anaerobic induc
ARE	Zea mays	1727	+	6	AAACCA	cis-acting regulatory element essential for the anaerobic induc
ARE	Zea mays	416	-	6	AAACCA	cis-acting regulatory element essential for the anaerobic induc

+ Box 4
+ CAAT-box
+ CGTCA-motif

Site Name	Organism	Position	Strand	Matrix score.	sequence	function
CGTCA-motif	Hordeum vulgare	653	-	5	CGTCA	cis-acting regulatory element involved in the MeJA-responsiver
CGTCA-motif	Hordeum vulgare	1412	-	5	CGTCA	cis-acting regulatory element involved in the MeJA-responsiver
CGTCA-motif	Hordeum vulgare	735	+	5	CGTCA	cis-acting regulatory element involved in the MeJA-responsiver

+ G-Box
+ G-box
+ GATA-motif
+ MBS

Site Name	Organism	Position	Strand	Matrix score.	sequence	function
MBS	Arabidopsis thaliana	1708	+	6	CAACTG	MYB binding site involved in drought-inducibility

+ MRE
+ MYB
+ MYB-like sequence
+ MYC
+ Myb
+ Myb-binding site
+ Myc
+ O2-site
+ P-box

Site Name	Organism	Position	Strand	Matrix score.	sequence	function
P-box	Oryza sativa	885	-	7	CCTTTTG	gibberellin-responsive element

+ STRE
+ TATA-box
+ TCA

Site Name	Organism	Position	Strand	Matrix score.	sequence	function
TCA	Pisum sativum	751	+	10	TCATCTTCAT	
TCA	Pisum sativum	1581	+	9	TCATCTTCAT	

+ TCT-motif
+ TGACG-motif

Site Name	Organism	Position	Strand	Matrix score.	sequence	function
TGACG-motif	Hordeum vulgare	653	+	5	TGACG	cis-acting regulatory element involved in the MeJA-responsiver
TGACG-motif	Hordeum vulgare	1412	+	5	TGACG	cis-acting regulatory element involved in the MeJA-responsiver
TGACG-motif	Hordeum vulgare	735	-	5	TGACG	cis-acting regulatory element involved in the MeJA-responsiver

+ Unnamed__1
+ Unnamed__10
+ Unnamed__12
+ Unnamed__14
+ Unnamed__4
+ Unnamed__8
+ W box
+ WUN-motif
+ as-1

>Traes_2BS_40C683B47.1

+ TGCTCAAATT TGCCCATGAA ATCACGAGCA GTATAGGTTG CACTGCTTGG CTCTCGTCGT CTAGTATACA
- ACGAGTTTAA ACGGGTACTT TAGTGCTCGT CATATCCAAC GTGACGAACC GAGAGCAGCA GATCATATGT

+ TGTCATAAGG GTATTTTGGG TAGAAAAAGT TTATGCCGGT CCGCCAGGCG AAGGCCATCT ATGGCGGATC
- ACAGTATTCC CATAAAACCC ATCTTTTTCA AATACGGCCA GGCGGTCCGC TTCCGGTAGA TACCGCCTAG

+ AAGAGGCTGC AGAGCTAGGC CTCTCTTCCT CGCCATCTAT GTTACGTTGT ACTCTCCAGT CTCTCCCTCC
- TTCTCCGACG TCTCGATCCG GAGAGAAGGA GCGGTAGATA CAATGCAACA TGAGAGGTCA GAGAGGGAGG

+ CTCCCTCCTC CTCTTCCTCC CACCACGAGG AGCCCGAGGA GGACTCGCCG CCCAGGACGC CACTACTCCC
- GAGGGAGGAG GAGAAGGAGG GTGGTGCTCC TCGGGCTCCT CCTGAGCGGC GGGTCCTGCG GTGATGAGGG

+ CAGGGACCTG ACGTGTTTCA GGACCGGTCG GCACTCACTC GTGACTGCTC TTGCCTCGTC TTCGTCTCCA
- GTCCCTGGAC TGCACAAAGT CCTGGCCAGC CGTGAGTGAG CACTGACGAG AACGGAGCAG AAGCAGAGGT

+ CGGAGGTCCC TGCCAGCGTC CCGCCGGAGC CGTGCACCCG TCTACTCCGG CGACCTCCCC TCCGGAAGCG
- GCCTCCAGGG ACGGTCGCAG GGCGGCCTCG GCACGTGGGC AGATGAGGCC GCTGGAGGGG AGGCCTTCGC

+ CGTGCAGCCT CAACCCGACT GGTCAAATGA AGCTGCAGGA CCTCCGAACC CACGTCCAGG AATTCTTGAC
- GCACGTCGGA GTTGGGCTGA CCAGTTTACT TCGACGTCCT GGAGGCTTGG GTGCAGGTCC TTAAGAACTG

+ ATCCCATGGC TTCCAAGTTC CAACGAACGC CTGCTCATCC GCTACAGCCG CCCGGTTGGG CTGGGCAGAC
- TAGGGTACCG AAGGTTCAAG GTTGCTTGCG GACGAGTAGG CGATGTCGGC GGGCCAACCC GACCCGTCTG

+ GAGACCATGG CTCCCTTAAT TTTTAAACGA AGAATGTCTA TCCTACCTCA GAAATTCAAT GGTCAGATTT
- CTCTGGTACC GAGGGAATTA AAAATTTGCT TCTTACAGAT AGGATGGAGT CTTTAAGTTA CCAGTCTAAA

+ TAATATACTG CTCCCTCCCC TGTCAGCAGT ATAGGGTACT GTAAAAACGC ACTTAGAAGA AGCTTAGCTC
- ATTATATGAC GAGGGAGGGG ACAGTCGTCA TATCCCATGA CATTTTTGCG TGAATCTTCT TCGAATCGAG

+ TCACCTTAGA GTGGTCCAAA TGACCCCTCT GCTCGATCAT TGCACTTGAT GTTATCGCTA TGCCTAGCCT
- AGTGGAATCT CACCAGGTTT ACTGGGGAGA CGAGCTAGTA ACGTGAACTA CAATAGCGAT ACGGATCGGA

+ TGATTGATCC ATGCGTGGTC ACTTGCTGTT AGAGATCAGA TAGCTACTTC AAGGTCGTGA GTTTATCCTT
- ACTAACTAGG TACGCACCAG TGAACGACAA TCTCTAGTCT ATCGATGAAG TTCCAGCACT CAAATAGGAA

+ CGGAAGATTA GTCGTGAATA GAACAGGGTT TATGGTAGAT CCCATGGTTG TGCCTCATGT TGGTTGCACC
- GCCTTCTAAT CAGCACTTAT CTTGTCCCAA ATACCATCTA GGGTACCAAC ACGGAGTACA ACCAACGTGG

+ ATACACGGCT CAGGGTAGCC GATTGTAACC CTATTATATA GAGGAATAAA ACTCGCAACT TTCTTGAAAA
- TATGTGCCGA GTCCCATCGG CTAACATTGG GATAATATAT CTCCTTATTT TGAGCGTTGA AAGAACTTTT

+ ATAAGTCTGT TTCAGGAATC CAAATCCAAC CAGCAATTCG TCGGCTTTGT CGAAAAAAAA ACCAATAATT
- TATTCAGACA AAGTCCTTAG GTTTAGGTTG GTCGTTAAGC AGCCGAAACA GCTTTTTTTT TGGTTATTAA

+ CGTCGGAGGC ATGGAAGCCA AAGAAACTGC GTCCAGCCTA CAGATCGGGC CACGTTACGG CCGGCCTAAA
- GCAGCCTCCG TACCTTCGGT TTCTTTGACG CAGGTCGGAT GTCTAGCCCG GTGCAATGCC GGCCGGATTT

+ CGCCCGTCGG TGCTGGCCTA CGGCTGTGCG GCCTCGGTCC AGCCTCACTA TTCCCACCGT GCGTTTGATA
- GCGGGCAGCC ACGACCGGAT GCCGACACGC CGGAGCCAGG TCGGAGTGAT AAGGGTGGCA CGCAAACTAT

+ AGGCTCAACC GCTCAAGGCA GCCTTACTAC CTCCGTTCTT AAAAAAGTAT ACTTTCAACT TCATTGAAGG
- TCCGAGTTGG CGAGTTCCGT CGGAATGATG GAGGCAAGAA TTTTTTCATA TGAAAGTTGA AGTAACTTCC

+ GTCAAACTAC TCAAAGTTTG ACCGAGTTTG TGCAAAAGTA TATCAATGTT TACAAAACCA AATAGGTGTA
- CAGTTTGATG AGTTTCAAAC TGGCTCAAAC ACGTTTTCAT ATAGTTACAA ATGTTTTGGT TTATCCACAT

+ TGATAAAAAT ATATTATATC ATGAATTTAA TACTACTAAT TTGATGTCAT AAATGTTTGT ATATTACTAT
- ACTATTTTTA TATAATATAG TACTTAAATT ATGATGATTA AACTACAGTA TTTACAAACA TATAATGATA

+ ATAAATGCAG TCAAACATAA AAAATTTGAA TTTTCAACAA AGTTGAAGGC ACACTCTTTC AAAAACGAAT
- TATTTACGTC AGTTTGTATT TTTTAAACTT AAAAGTTGTT TCAACTTCCG TGTGAGAAAG TTTTTGCTTA

+ GGAGTACCTC TCAATGTCCC AGCTACCGAA TTGTCCGTGT TGGCGGAAGG CGACGGGAGC GCAAGATGGC
- CCTCATGGAG AGTTACAGGG TCGATGGCTT AACAGGCACA ACCGCCTTCC GCTGCCCTCG CGTTCTACCG

+ TGCGCACCGG TCAAAACGAG CGTGAATCGC CTTGGAACAA CGCCGACTCC CCACTACTAT TCGCACGTAA
- ACGCGTGGCC AGTTTTGCTC GCACTTAGCG GAACCTTGTT GCGGCTGAGG GGTGATGATA AGCGTGCATT

+ GGCCAAACGG TTGATGTTCA CGCACACATG GCTCCAGGCG TTCGCTAGCA TGGAAGTGGT GAAAAAAGAG
- CCGGTTTGCC AACTACAAGT GCGTGTGTAC CGAGGTCCGC AAGCGATCGT ACCTTCACCA CTTTTTTCTC

+ AGATCGTTCA ATGTTTTGCC GATTTTTGCG TGATAAACGC CAAGAGAGAT CGCTCAAGTT GGCCCTGTTG
- TCTAGCAAGT TACAAAACGG CTAAAAACGC ACTATTTGCG GTTCTCTCTA GCGAGTTCAA CCGGGACAAC

+ CAAGGAAAAT TCAGGTGTTG GTGTTGGCGG TAGCGGCTCA AGCTGGAAGC TGCCACGAAT ACGCCAAGCT
- GTTCCTTTTA AGTCCACAAC CACAACCGCC ATCGCCGAGT TCGACCTTCG ACGGTGCTTA TGCGGTTCGA

+ GGAAGCTCGA AGTATACCCA ATCGAACGCG AGTCAGAGAC TAACGTTGGA CTAATGGGAC CTAAATTTTG
- CCTTCGAGCT TCATATGGGT TAGCTTGCGC TCAGTCTCTG ATTGCAACCT GATTACCCTG GATTTAAAAC

+ GATGAGCTAT CGCTAGTCGG AGACTGGAGC GATCTGAAGG TGCACGTGAG CTAAATCTTT GGCTATAAAT
- CTACTCGATA GCGATCAGCC TCTGACCTCG CTAGACTTCC ACGTGCACTC GATTTAGAAA CCGATATTTA

+ ATGCATGCGA TGCACCACTC TCTTGCACAA GCCACTAA
- TACGTACGCT ACGTGGTGAG AGAACGTGTT CGGTGATT

Motifs Found

+
+ ABRE

Site Name	Organism	Position	Strand	Matrix score.	sequence	function
ABRE	Arabidopsis thaliana	1101	-	5	ACGTG	cis-acting element involved in the abscisic acid responsivene
ABRE	Arabidopsis thaliana	1933	-	6	CACGTG	cis-acting element involved in the abscisic acid responsivene
ABRE	Arabidopsis thaliana	471	-	5	ACGTG	cis-acting element involved in the abscisic acid responsivene
ABRE	Arabidopsis thaliana	1934	+	5	ACGTG	cis-acting element involved in the abscisic acid responsivene
ABRE	Arabidopsis thaliana	1604	-	5	ACGTG	cis-acting element involved in the abscisic acid responsivene
ABRE	Arabidopsis thaliana	291	+	5	ACGTG	cis-acting element involved in the abscisic acid responsivene

+ ABRE3a

Site Name	Organism	Position	Strand	Matrix score.	sequence	function
ABRE3a	Zea mays	1604	-	6	TACGTG	

+ ABRE4

Site Name	Organism	Position	Strand	Matrix score.	sequence	function
ABRE4	Zea mays	1604	+	6	CACGTA	

+ ARE

Site Name	Organism	Position	Strand	Matrix score.	sequence	function
ARE	Zea mays	1039	+	6	AAACCA	cis-acting regulatory element essential for the anaerobic induc
ARE	Zea mays	1315	+	6	AAACCA	cis-acting regulatory element essential for the anaerobic induc

+ AT~TATA-box
+ CAAT-box
+ CAT-box
+ CGTCA-motif

Site Name	Organism	Position	Strand	Matrix score.	sequence	function
CGTCA-motif	Hordeum vulgare	289	-	5	CGTCA	cis-acting regulatory element involved in the MeJA-responsiver

+ DRE core
+ G-Box
+ G-box
+ MYB
+ MYC
+ Myb-binding site
+ STRE
+ Sp1
+ TATA-box
+ TCA

Site Name	Organism	Position	Strand	Matrix score.	sequence	function
TCA	Pisum sativum	1245	+	9	TCATCTTCAT	

+ TCCC-motif
+ TGACG-motif

Site Name	Organism	Position	Strand	Matrix score.	sequence	function
TGACG-motif	Hordeum vulgare	289	+	5	TGACG	cis-acting regulatory element involved in the MeJA-responsiver

+ Unnamed__1
+ Unnamed__4
+ W box
+ as-1

>Traes_6AS_5BAD56BB6.1

+ CCCCGGTGAT TGTTAAAAAT GATCTATTTG CATGTAGTTA CACACTTAAA TCAGGACTTG GCCCTTTCAT
- GGGGCCACTA ACAATTTTTA CTAGATAAAC GTACATCAAT GTGTGAATTT AGTCCTGAAC CGGGAAAGTA

+ GTATGTCATG TTGTCAAATT AATATGTTGA AAGAATGTTA TATTTTCCAC AAAGGTAAAT GAAATAATGT
- CATACAGTAC AACAGTTTAA TTATACAACT TTCTTACAAT ATAAAAGGTG TTTCCATTTA CTTTATTACA

+ TTATGTGCAG AAACATAGTT CTTAGCTATA TTCAAAACAT GTATTGTTCT CGATCCCTTT TCAGTCCAAC
- AATACACGTC TTTGTATCAA GAATCGATAT AAGTTTTGTA CATAACAAGA GCTAGGGAAA AGTCAGGTTG

+ ATCTTATGTC AAGCATGTAC TCTATATGTA CCTCTATCGG TGCATCAAGC ACTATTTGGC CCTTGGGGTG
- TAGAATACAG TTCGTACATG AGATATACAT GGAGATAGCC ACGTAGTTCG TGATAAACCG GGAACCCCAC

+ CTTAGGTATG TGGAAAGAGG GTTGGAGTTG ACTAGGTGAT GTGGCAACCG CGGTGGTATA ACTAGGTTCT
- GAATCCATAC ACCTTTCTCC CAACCTCAAC TGATCCACTA CACCGTTGGC GCCACCATAT TGATCCAAGA

+ CACTAAGGAG TATCACTCTA ACCAAAGATG TCACAAGAAC GGTTCATGGT GGTTCAAATT CCAAATTTGA
- GTGATTCCTC ATAGTGAGAT TGGTTTCTAC AGTGTTCTTG CCAAGTACCA CCAAGTTTAA GGTTTAAACT

+ TACCAGATAA ATGAAACAAC TTTTACTGCT TTCATCTTTG TATGTCTCAA TAGAGGTTCA TGGTGGCGTT
- ATGGTCTATT TACTTTGTTG AAAATGACGA AAGTAGAAAC ATACAGAGTT ATCTCCAAGT ACCACCGCAA

+ CAATGAAGGT TTTATTTCCC ACAATCGAGG ATTCAATTAG TAAGGTGGTT TCAAAGAAGT TGTATATGGT
- GTTACTTCCA AAATAAAGGG TGTTAGCTCC TAAGTTAATC ATTCCACCAA AGTTTCTTCA ACATATACCA

+ TTTGTGTTTG CCTCCTTTTG AAGAGTTTCA TGGGTGTTAC AGTGATTTTT AGGAAGTCTA TTAGAAGATT
- AAACACAAAC GGAGGAAAAC TTCTCAAAGT ACCCACAATG TCACTAAAAA TCCTTCAGAT AATCTTCTAA

+ GGAATAGTTG GAGAGATTGT TAAAGGGATC CCCCTTTTTT CCTTTGTTTT AATGAAGTTT CCGGGAAGTT
- CCTTATCAAC CTCTCTAACA ATTTCCCTAG GGGGAAAAAA GGAAACAAAA TTACTTCAAA GGCCCTTCAA

+ CGAAATATCT CATCTGAATT CAATAGAAGA CATACTATTG AATTTAGCTG ACCTATGGCA ACATTAATTC
- GCTTTATAGA GTAGACTTAA GTTATCTTCT GTATGATAAC TTAAATCGAC TGGATACCGT TGTAATTAAG

+ ACCTCTCATC CCGCAAACAA AATTAAACAC TTGCCTCCTA GGGAGAGGAT TTGTGGCATG CAAGTGCGGG
- TGGAGAGTAG GGCGTTTGTT TTAATTTGTG AACGGAGGAT CCCTCTCCTA AACACCGTAC GTTCACGCCC

+ CGCACGCATG TCCCTCGTGT CCGCTTTTTA TTTTGGCTTC TGATTTTCAA AGTTTTATGT CTTTTAAATG
- GCGTGCGTAC AGGGAGCACA GGCGAAAAAT AAAACCGAAG ACTAAAAGTT TCAAAATACA GAAAATTTAC

+ AAAATTCCAA ACTAAGTTCT GTTTTCATAT TCATGCTTCT CGTGACCAAC ACTTTCAAAT AAGATCCATT
- TTTTAAGGTT TGATTCAAGA CAAAAGTATA AGTACGAAGA GCACTGGTTG TGAAAGTTTA TTCTAGGTAA

+ TTGAATATAT TTCCATGACT TTTTTTTTAA ATGTTAAGTT TCAATGTTGA TACTTAGTAC GAGTGATCGA
- AACTTATATA AAGGTACTGA AAAAAAAATT TACAATTCAA AGTTACAACT ATGAATCATG CTCACTAGCT

+ TAAAAATCAA TTATTTTGTG TCTAGGACCG ATGGAAAAAT TACTTGAAAT TATATATCTA AAATTGATTG
- ATTTTTAGTT AATAAAACAC AGATCCTGGC TACCTTTTTA ATGAACTTTA ATATATAGAT TTTAACTAAC

+ AAACTTGACT GGTTTAGATG CAAAAACTAT AAGTTTTGTT GTGGAAACTT AAAACTTTTT GAGGGATTTT
- TTTGAACTGA CCAAATCTAC GTTTTTGATA TTCAAAACAA CACCTTTGAA TTTTGAAAAA CTCCCTAAAA

+ CTATTTTACT GTGCCCTCAT GTGGGATTGA ACTACTGCGC GAATCGCGGG GCAAACCGAG CGGGCAGTAG
- GATAAAATGA CACGGGAGTA CACCCTAACT TGATGACGCG CTTAGCGCCC CGTTTGGCTC GCCCGTCATC

+ GACTTGGCAG GTGAGTGCCG CGCACTCACG TGCTCCTGCG CATTTCCGTT ACCTCCCACT ATGAATGCAC
- CTGAACCGTC CACTCACGGC GCGTGAGTGC ACGAGGACGC GTAAAGGCAA TGGAGGGTGA TACTTACGTG

+ TTGGAACACT CCTCTGATGG TTCTTTAATT ATGTTTTTTC TATTCTTTTA TCTTTGTTTG CCTGGAATAG
- AACCTTGTGA GGAGACTACC AAGAAATTAA TACAAAAAAG ATAAGAAAAT AGAAACAAAC GGACCTTATC

+ TTGCCATCAT GACTTGATCA CATATCCTTC TCCGGTCCCA TGTCCTATCA GCAATTACAT AAAATTAGTT
- AACGGTAGTA CTGAACTAGT GTATAGGAAG AGGCCAGGGT ACAGGATAGT CGTTAATGTA TTTTAATCAA

+ TGCTGAAGAT GATCTTTATC CATAAAGATC GACAACCACA CACTAAATTA TTTATACATA ATTTCTTATA
- ACGACTTCTA CTAGAAATAG GTATTTCTAG CTGTTGGTGT GTGATTTAAT AAATATGTAT TAAAGAATAT

+ TATACTGGAA TGCCATTTGG CAGTGACAGA TGAGCAACAT ATATATAAAT AAAGACAGAT CAGTTTTTGG
- ATATGACCTT ACGGTAAACC GTCACTGTCT ACTCGTTGTA TATATATTTA TTTCTGTCTA GTCAAAAACC

+ CATCTACAGA GAGATGCGTT GCTCTTAGTC AACCAGAGAA AGATACCACG TGGAACTTGT CATAGTTCTC
- GTAGATGTCT CTCTACGCAA CGAGAATCAG TTGGTCTCTT TCTATGGTGC ACCTTGAACA GTATCAAGAG

+ CACAACCCTA ACGGCCTAAC TTTGACCAAT CTACATGCAT CTCATGCAAC TGAAAGAAGA GTGCTATTAT
- GTGTTGGGAT TGCCGGATTG AAACTGGTTA GATGTACGTA GAGTACGTTG ACTTTCTTCT CACGATAATA

+ AAAT
- TTTA

Motifs Found

+
+ AAGAA-motif
+ ABRE

Site Name	Organism	Position	Strand	Matrix score.	sequence	function
ABRE	Arabidopsis thaliana	1287	-	6	CACGTG	cis-acting element involved in the abscisic acid responsivene
ABRE	Arabidopsis thaliana	1657	-	6	CACGTG	cis-acting element involved in the abscisic acid responsivene
ABRE	Arabidopsis thaliana	1288	+	5	ACGTG	cis-acting element involved in the abscisic acid responsivene
ABRE	Arabidopsis thaliana	1658	+	5	ACGTG	cis-acting element involved in the abscisic acid responsivene

+ ABRE2

Site Name	Organism	Position	Strand	Matrix score.	sequence	function
ABRE2	Zea mays	1656	-	8	CCACGTGG	

+ ARE

Site Name	Organism	Position	Strand	Matrix score.	sequence	function
ARE	Zea mays	536	-	6	AAACCA	cis-acting regulatory element essential for the anaerobic induc
ARE	Zea mays	1130	-	6	AAACCA	cis-acting regulatory element essential for the anaerobic induc
ARE	Zea mays	557	-	6	AAACCA	cis-acting regulatory element essential for the anaerobic induc

+ AT-rich element
+ AT~TATA-box
+ Box 4
+ CAAT-box
+ G-Box
+ G-box
+ GATA-motif
+ MBS

Site Name	Organism	Position	Strand	Matrix score.	sequence	function
MBS	Arabidopsis thaliana	1727	+	6	CAACTG	MYB binding site involved in drought-inducibility

+ MYB
+ MYB-like sequence
+ MYC
+ Myb
+ O2-site
+ P-box

Site Name	Organism	Position	Strand	Matrix score.	sequence	function
P-box	Oryza sativa	574	+	7	CCTTTTG	gibberellin-responsive element

+ STRE
+ TATA-box
+ TCA

Site Name	Organism	Position	Strand	Matrix score.	sequence	function
TCA	Pisum sativum	935	+	9	TCATCTTCAT	
TCA	Pisum sativum	1473	-	9	TCATCTTCAT	

+ TCCC-motif
+ Unnamed__1
+ Unnamed__2
+ Unnamed__4
+ W box
+ WRE3
+ WUN-motif
+ circadian

>Traes_2BS_B6EBC0962.1

```
+ GCTTTGCCGT AGGCATAGCC CTGCCGCCAG GAGAAGCCGG GGGACGACAC GTGGCGCAGG TATGCTGACG
- CGAAACGGCA TCCGTATCGG GACGGCGGTC CTCTTCGGCC CCCTGCTGTG CACCGCGTCC ATACGACTGC

+ ACTAGGCTGT CGGCATAGAT TTCCATCTAT GCCGACGGCC GAGCCGTCGG CATACATGCG CCACGTGTCG
- TGATCCGACA GCCGTATCTA AAGGTAGATA CGGCTGCCGG CTCGGCAGCC GTATGTACGC GGTGCACAGC

+ TCCCCTGAAT CGCCAGCGCT GTTGACGGCG CCGTCCGTTG CCGTCAGACG GAAAACAACG CCGATGGCTA
- AGGGGACTTA GCGGTCGCGA CAACTGCCGC GGCAGGCAAC GGCAGTCTGC CTTTTGTTGC GGCTACCGAT

+ AACTATGCCG ACGGCTCTCC CACGGCCGTC GGCATATGCT CCTATGTCGA CGGCTATACT ACGCCGACGG
- TTGATACGGC TGCCGAGAGG GTGCCGGCAG CCGTATACGA GGATACAGCT GCCGATATGA TGCGGCTGCC

+ TCTGACACAT CTACGCTGAC GTGATCTACG CCGACGGGGA TATGCCGATG GCAGCCGTAG GCATAGATCT
- AGACTGTGTA GATGCGACTG CACTAGATGC GGCTGCCCCT ATACGGCTAC CGTCGGCATC CGTATCTAGA

+ ATGCCGACGG CAATGGACCT ATGCCGACGG CCCTGGGCCG TAGGCATAGC CCGCGAGTCC GGTGGTGTTT
- TACGGCTGCC GTTACCTGGA TACGGCTGCC GGGACCCGGC ATCCGTATCG GGCGCTCAGG CCACCACAAA

+ GGATTTTTGC CCCAAGGATG CCCATTCAAA AATACCAGGA TCAAGAGTTA TGCTACTTTA GCTTTTGCAG
- CCTAAAAACG GGGTTCCTAC GGGTAAGTTT TTATGGTCCT AGTTCTCAAT ACGATGAAAT CGAAAACGTC

+ GACCCACATG TGATAATTGT GGTTAAAATG CCCAAAGAGA CTGATCAGTG TTTTTTGTAA AAGATTAGGC
- CTGGGTGTAC ACTATTAACA CCAATTTTAC GGGTTTCTCT GACTAGTCAC AAAAAACATT TTCTAATCCG

+ AAAGAATCAG TGGTTCTGGC CCAAATTCAT AAACTGGTGT TTTGTGCGAA CCTTGGCAAC AATATGGTGG
- TTTCTTAGTC ACCAAGACCG GGTTTAAGTA TTTGACCACA AAACACGCTT GGAACCGTTG TTATACCACC

+ TTTCTTTTGC AATTCACTCG AGTTGGGCAG CTATCTACGT AGTGCAAAAC ATTCAAGACA AATCCGTTGT
- AAAGAAAACG TTAAGTGAGC TCAACCCGTC GATAGATGCA TCACGTTTTG TAAGTTCTGT TTAGGCAACA

+ TGGATCGTGA GCCAAGAAAA GAGCTTGACT TTTGATGGAG TCCAAGCCTT TCATATCATG GGATCCATCG
- ACCTAGCACT CGGTTCTTTT CTCGAACTGA AAACTACCTC AGGTTCGGAA AGTATAGTAC CCTAGGTAGC

+ GGGAAAGAGT CATCGCAAGA AATTGTGCTT ATATATGCGC TGGACGCCCA ATAGATCTCA TCATTAGAGT
- CCCTTTCTCA GTAGCGTTCT TTAACACGAA TATATACGCG ACCTGCGGGT TATCTAGAGT AGTAATCTCA

+ GCTTCCAGAG AATGTCATCC TCCACAAGTT CATCAAGAGG GAAATCATGC ACAAGCATCC AAAGAGTAAA
- CGAAGGTCTC TTACAGTAGG AGGTGTTCAA GTAGTTCTCC CTTTAGTACG TGTTCGTAGG TTTCTCATTT

+ AACTCCCATA TGTGGGCAGC GGAGACGATA GTGCTTTATT TGATCATGAG GATCCAAGCG TTATCTTTAA
- TTGAGGGTAT ACACCCGTCG CCTCTGCTAT CACGAAATAA ACTAGTACTC CTAGGTTCGC AATAGAAATT

+ GGGCTTCAAG CATCTTTCAA TTCTTCCTTC TCAAGGCTTC AAAGATGTGG TGCGATGTCC TTAGGCTTAT
- CCCGAAGTTC GTAGAAAGTT AAGAAGGAAG AGTTCCGAAG TTTCTACACC ACGCTACAGG AATCCGAATA

+ TCCCAACGAG CCATAGGGAG TCCCAATATG GTGTCTTTGC GCCATGGCTC ACGGTGATAG TGGTGGAAGC
- AGGGTTGCTC GGTATCCCTC AGGGTTATAC CACAGAAACG CGGTACCGAG TGCCACTATC ACCACCTTCG

+ ATATAAAAAT ATGGGTCCTC CTCAGTGCAC GGATTTTCAT GCCCCACACA CAGTTTGTTG GGCTCTTTTC
- TATATTTTTA TACCCAGGAG GAGTCACGTG CCTAAAAGTA CGGGGTGTGT GTCAAACAAC CCGAGAAAAG

+ ATTCATACCA TGGCCAATGA AGGCGCAAAG CTCTTACAAA TTTGTCAGTG TTTAGGACGC CAAGGCCACC
- TAAGTATGGT ACCGGTTACT TCCGCGTTTC GAGAATGTTT AAACAGTCAC AAATCCTGCG GTTCCGGTGG

+ GTACTCACTG GGGCGGCATA CAATCTCCCA GCTGACATTG CGCTTGGCGC TGATCGTCTT GTTGGAGCCG
- CATGAGTGAC CCCGCCGTAT GTTAGAGGGT CGACTGTAAC GCGAACCGCG ACTAGCAGAA CAACCTCGGC

+ GACCAAAGAA AAGCCCGGTC AATTTTGTTG AGGTTTTGGA CCGTGCTCGG CGGGACGATG AGAGGCGTGA
- CTGGTTTCTT TTCGGGCCAG TTAAAACAAC TCCAAAACCT GGCACGAGCC GCCCTGCTAC TCTCCGCACT

+ TGGAGTACAC CGCTTCGGAG GACACGACCG ACTTAACAAG CAAAGTTCGA CCAATGTTAG TTACAGTTTC
- ACCTCATGTG GCGAAGCCTC CTGTGCTGGC TGAATTGTTC GTTTCAAGCT GGTTACAATC AATGTCAAAG

+ TGGAAAAAAA AATACAGACG GTGGATGCCG CCTCAATCTT CCGGTGTCCA TCTCCCCCTA TGCTTCAAGA
- ACCTTTTTTT TTATGTCTGC CACCTACGGC GGAGTTAGAA GGCCACAGGT AGAGGGGGAT ACGAAGTTCT

+ AGCCAGGCTC ACCTCTCAGC GTCCCAGCTG CGGAATTGTC TCGTGTTGGC CGAAGGCGAC GGGAGCGCAA
- TCGGTCCGAG TGGAGAGTCG CAGGGTCGAC GCCTTAACAG AGCACAACCG GCTTCCGCTG CCCTCGCGTT

+ GATGACTGCG CACCGGTCAA AACGAGGGTG AATCGCCTTG GAACAACGCA CACATGGCTC CAGGCGTCCC
- CTACTGACGC GTGGCCAGTT TTGCTCCCAC TTAGCGGAAC CTTGTTGCGT GTGTACCGAG GTCCGCAGGG

+ TACTATACTA TTCGTACGTA CGTAAGGCCA AACGGTTGGT GTCCACGCAC ACATGGCTCC AGGCGTCCGT
- ATGATATGAT AAGCATGCAT GCATTCCGGT TTGCCAACCA CAGGTGCGTG TGTACCGAGG TCCGCAGGCA

+ GAGCATGGAA GTGATGAAAA AAGAGAGATC GTTCAATGTT TTGCATATAT CTGCGCGATA AACTCCAAGA
- CTCGTACCTT CACTACTTTT TTCTCTCTAG CAAGTTACAA AACGTATATA GACGCGCTAT TTGAGGTTCT

+ GAGATCGCTC AAGTTGGCCA GGTTGGTGTT GGCGGTAGCG ACTCAAGCTG GAAGCTGCCG CAGCCTAAGC
- CTCTAGCGAG TTCAACCGGT CCAACCACAA CCGCCATCGC TGAGTTCGAC CTTCGACGGC GTCGGATTCG

+ CTTGGATGCA TGCGCGACCC TTCCTTCCAA ATAAGCTTGT CGATCGATGC CAATATGCCA TGTACCTAGC
- GAACCTACGT ACGCGCTGGG AAGGAAGGTT TATTCGAACA GCTAGCTACG GTTATACGGT ACATGGATCG

+ AGATTGCGGG GTACCCGATC GAACGGCTAG CTATATCG
- TCTAACGCCC CATGGGCTAG CTTGCCGATC GATATAGC
```

Motifs Found

+
+ A-box
+ ABRE

Site Name	Organism	Position	Strand	Matrix score.	sequence	function
ABRE	Hordeum vulgare	46	-	9	GCAACGTGTC	cis-acting element involved in the abscisic acid responsivene
ABRE	Arabidopsis thaliana	132	+	6	CACGTG	cis-acting element involved in the abscisic acid responsivene
ABRE	Arabidopsis thaliana	49	+	5	ACGTG	cis-acting element involved in the abscisic acid responsivene
ABRE	Arabidopsis thaliana	299	+	5	ACGTG	cis-acting element involved in the abscisic acid responsivene
ABRE	Arabidopsis thaliana	48	+	6	CACGTG	cis-acting element involved in the abscisic acid responsivene
ABRE	Arabidopsis thaliana	133	+	5	ACGTG	cis-acting element involved in the abscisic acid responsivene
ABRE	Hordeum vulgare	130	+	9	GCAACGTGTC	cis-acting element involved in the abscisic acid responsivene

+ ARE

Site Name	Organism	Position	Strand	Matrix score.	sequence	function
ARE	Zea mays	628	-	6	AAACCA	cis-acting regulatory element essential for the anaerobic induc

+ AT~TATA-box
+ AuxRR-core

Site Name	Organism	Position	Strand	Matrix score.	sequence	function
AuxRR-core	Nicotiana tabacum	363	-	7	GGTCCAT	cis-acting regulatory element involved in auxin responsivene

+ CAAT-box
+ CCAAT-box
+ CCGTCC motif

Site Name	Organism	Position	Strand	Matrix score.	sequence	function
CCGTCC motif	Nicotiana tabacum	171	+	6	CCGTCC	

+ CCGTCC-box
+ CGTCA-motif
+ DRE core
+ G-Box
+ G-box
+ GC-motif
+ GT1-motif
+ I-box
+ MSA-like
+ MYB
+ MYB recognition site
+ MYB-like sequence
+ MYC
+ Myb-binding site
+ O2-site
+ STRE
+ Sp1
+ TATA-box
+ TCA-element

Site Name	Organism	Position	Strand	Matrix score.	sequence	function
TCA-element	Nicotiana tabacum	1125	-	9	CCATCTTTTT	cis-acting element involved in salicylic acid responsivenes

+ TCT-motif
+ TGACG-motif

Site Name	Organism	Position	Strand	Matrix score.	sequence	function
TGACG-motif	Hordeum vulgare	66	+	5	TGACG	cis-acting regulatory element involved in the MeJA-responsiver
TGACG-motif	Hordeum vulgare	182	-	5	TGACG	cis-acting regulatory element involved in the MeJA-responsiver
TGACG-motif	Hordeum vulgare	163	+	5	TGACG	cis-acting regulatory element involved in the MeJA-responsiver
TGACG-motif	Hordeum vulgare	297	+	5	TGACG	cis-acting regulatory element involved in the MeJA-responsiver

+ Unnamed__1
+ Unnamed__2
+ Unnamed__4
+ W box

>Traes_1AS_6C84785B3.2

```
+ TTTAAGGGTT TGAGGGTTAA GGGGTTCTAG TCTACACGAT TTCTTGCTCG CAAACTGTAA AAAAGCAGTT
- AAATTCCCAA ACTCCCAATT CCCCAAGATC AGATGTGCTA AAGAACGAGC GTTTGACATT TTTTCGTCAA

+ ATTTATAAGG GTTTGGAGGT TTGAGGGCTC TGCTAGAGAT GCTCTTATAG CATCTTCAGC AGACCTCGTA
- TAAATATTCC CAAACCTCCA AACTCCCGAG ACGATCTCTA CGAGAATATC GTAGAAGTCG TCTGGAGCAT

+ TAGATGGTCA AACCCATAAA ATAACCATTT TTTGTAATTT CAATAAAAAG AAGATCCGGA CAAATCCTTT
- ATCTACCAGT TTGGGTATTT TATTGGTAAA AAACATTAAA GTTATTTTTC TTCTAGGCCT GTTTAGGAAA

+ AAAATCGTCC GACCTTTAAA AAATTTTAAC GAGCCCCGTA AACATGTAGC CGAAACCCTC GTATCTATAA
- TTTTAGCAGG CTGGAAATTT TTTAAAATTG CTCGGGGCAT TTGTACATCG GCTTTGGGAG CATAGATATT

+ TTTTGATGGC AACTTTTACA AAGCCCTAGA TACCAATCAA CGTTGGCGGT TGAAAAATCT CTACTCCGGC
- AAAACTACCG TTGAAAATGT TTCGGGATCT ATGGTTAGTT GCAACCGCCA ACTTTTTAGA GATGAGGCCG

+ TAACGCCATG AAGCTCGCCC GGCCGCCCGG CCACCACGCC CGTCCGTCCA TGCCGGCCGG CCGGCCGCGT
- ATTGCGGTAC TTCGAGCGGG CCGGCGGGCC GGTGGTGCGG GCAGGCAGGT ACGGCCGGCC GGCCGGCGCA

+ CGTCCACCCC GGCCACAGCG CACAGCGACG GAGGTGCTAG CTAGCTAGCT CGCTTTTGGA TGGATTCGAG
- GCAGGTGGGG CCGGTGTCGC GTGTCGCTGC CTCCACGATC GATCGATCGA GCGAAAACCT ACCTAAGCTC

+ CTAGGCTAGC TAGCTAGCTC GCTCCTGGAT GGATTGGAGC AGAGCTAGCT AGCTAGCTCT TGATCGATTC
- GATCCGATCG ATCGATCGAG CGAGGACCTA CCTAACCTCG TCTCGATCGA TCGATCGAGA ACTAGCTAAG

+ GAGCAGATCT AATGGATGGA TCCCGCGGCT CTATTTTTGA CCGTTTCGTG TGTTATTTGT CGATTTTGCG
- CTCGTCTAGA TTACCTACCT AGGGCGCCGA GATAAAAACT GGCAAAGCAC ACAATAAACA GCTAAAACGC

+ GAAGCAAGCT AGCTGACTAT GGACACGGAT GAGAAGAAGA AGAAGATGTG AAGAAAGAAT ATAAGTGAAT
- CTTCGTTCGA TCGACTGATA CCTGTGCCTA CTCTTCTTCT TCTTCTACAC TTCTTTCTTA TATTCACTTA

+ ATTCCGTTTT ACAGTTTAAA TTTGAGGGGT CTGTTCTGCC GCAGTAAAAA CCGATCCTTA AAACGCGTTT
- TAAGGCAAAA TGTCAAATTT AAACTCCCCA GACAAGACGG CGTCATTTTT GGCTAGGAAT TTTGCGCAAA

+ TTCATGAACT GCAAAAGTGT TTTGCGGATC GGCAGGATAT AGGGTCTGCT AGAGATGCTC TTAGAGTCGC
- AAGTACTTGA CGTTTTCACA AAACGCCTAG CCGTCCTATA TCCCAGACGA TCTCTACGAG AATCTCAGCG

+ AACAACCTTC GCGTCACTAT CGGGGCGGCA ATCCTCTCGA TCAGCTGCTG CATTGAGCAC TTGCGCTTGC
- TTGTTGGAAG CGCAGTGATA GCCCCGCCGT TAGGAGAGCT AGTCGACGAC GTAACTCGTG AACGCGAACG

+ ACGATCCGAA CTACGCTTGC TATAGAAATG ACACCACGCC AGCCCATGAC GGCTAGGCTT TTCCCTTAAC
- TGCTAGGCTT GATGCGAACG ATATCTTTAC TGTGGTGCGG TCGGGTACTG CCGATCCGAA AAGGGAATTG

+ TCTCTTTTTG TCATTTTAGT TTTTGCTCAT TTTACTTATG CCACTCTAGA ATTTAACATT TTACTTTTAC
- AGAGAAAAAC AGTAAAATCA AAAACGAGTA AAATGAATAC GGTGAGATCT TAAATTGTAA AATGAAAATG

+ CATTTTTTGC TTTTAACAAT ACATCATAAA TGCACCGTGG CAAAAACAAT AATGTTTTAT TTCACTTTTG
- GTAAAAAACG AAAATTGTTA TGTAGTATTT ACGTGGCACC GTTTTTGTTA TTACAAAATA AAGTGAAAAC

+ CCATTCTTAA CTTTTGACAA TGTATTACAA TTGTCACTTC AAATATTTTG TTTTTGCCAC GGAATGGCAA
- GGTAAGAATT GAAAACTGTT ACATAATGTT AACAGTGAAG TTTATAAAAC AAAAACGGTG CCTTACCGTT

+ AAGTGGTATA TTATCAAAAG CTAAGAGTGA CAAAAGTGAA ATGTCAAATT CTAGAGTGGC ATAAGCACAG
- TTCACCATAT AATAGTTTTC GATTCTCACT GTTTTCACTT TACAGTTTAA GATCTCACCG TATTCGTGTC

+ TGGACAAAAG CTAAAGTGGC AGAAACAAAG TTTTCCCTCG ATTGAGAACT GTTTTCCCTC GATTGAGAAC
- ACCTGTTTTC GATTTCACCG TCTTTGTTTC AAAAGGGAGC TAACTCTTGA CAAAAGGGAG CTAACTCTTG

+ TTCATAAGTC TGGGTCGATT TCTGTATACT GCAACTTCAG AATAGTTCTT TTTCTAGTAT GCGCTGAATT
- AAGTATTCAG ACCCAGCTAA AGACATATGA CGTTGAAGTC TTATCAAGAA AAAGATCATA CGCGACTTAA

+ TATTTTTTAG CATGTGTTGG CCTTAAGGCG GTTATCATCT TGTGCAAAAC GTGCATCCGC TTAAGGCTCT
- ATAAAAAATC GTACACAACC GGAATTCCGC CAATAGTAGA ACACGTTTTG CACGTAGGCG AATTCCGAGA

+ ACTGAAAATT TTTCCTTTTA TTTGAAGAGG GTTTCCTATG GAGTTGATCT GCAATGCTAG TACATATATC
- TGACTTTTAA AAAGGAAAAT AAACTTCTCC CAAAGGATAC CTCAACTAGA CGTTACGATC ATGTATATAG

+ GGCGTAATTC AAGATAAGCT CGCAATATGC ATGTCAGAAA AGGAGAGCTA TAGCTCCCGG TGCAGGAAAT
- CCGCATTAAG TTCTATTCGA GCGTTATACG TACAGTCTTT TCCTCTCGAT ATCGAGGGCC ACGTCCTTTA

+ ATGCCAACCA GAAGAAAGGG ACATAAGAAA AGACACTATA GAAAAGGCAC AGTTACGTGG CTCATCAAGA
- TACGGTTGGT CTTCTTTCCC TGTATTCTTT TCTGTGATAT CTTTTCCGTG TCAATGCACC GAGTAGTTCT

+ AAACAGTCTC TCTCGAGTCT TGAGCACTGC ATTACAATCC ATCTATTCAA TCAGCAGACG TACATTACAT
- TTTGTCAGAG AGAGCTCAGA ACTCGTGACG TAATGTTAGG TAGATAAGTT AGTCGTCTGC ATGTAATGTA

+ TAACTGATGC GATCACGCAT GCTCCTAAAC CTCCTATCAT GGACTTCGAC CCTTTGAGAA ACCCCTGCAG
- ATTGACTACG CTAGTGCGTA CGAGGATTTG GAGGATAGTA CCTGAAGCTG GGAAACTCTT TGGGGACGTC

+ TACGGGAACA AAAGCAAGTT AGGACGGCCA TGGATCACCA ACCGGCCAGG ACAAATGAAC ATATGATTGA
- ATGCCCTTGT TTTCGTTCAA TCCTGCCGGT ACCTAGTGGT TGGCCGGTCC TGTTTACTTG TATACTAACT

+ GTACTTAATC AGCCACTAGG AGCAGTCGAT CAGTTGATGA TTAGACTAAA GCAAGGCGCG TGTGCATGCC
- CATGAATTAG TCGGTGATCC TCGTCAGCTA GTCAACTACT AATCTGATTT CGTTCCGCGC ACACGTACGG

+ GTGTTCATTT GTGTTTTGTT CGAAAACGAA GGGCGAGA
- CACAAGTAAA CACAAAACAA GCTTTTGCTT CCCGCTCT
```

Motifs Found

+
+ A-box
+ AAGAA-motif
+ ABRE

Site Name	Organism	Position	Strand	Matrix score.	sequence	function
ABRE	Arabidopsis thaliana	1449	+	5	ACGTG	cis-acting element involved in the abscisic acid responsivene
ABRE	Arabidopsis thaliana	1665	+	5	ACGTG	cis-acting element involved in the abscisic acid responsivene

+ ABRE3a

Site Name	Organism	Position	Strand	Matrix score.	sequence	function
ABRE3a	Zea mays	1664	+	6	TACGTG	

+ ABRE4

Site Name	Organism	Position	Strand	Matrix score.	sequence	function
ABRE4	Zea mays	1664	-	6	CACGTA	

+ ACE
+ AE-box
+ CAAT-box
+ CAT-box
+ CCGTCC motif
+ CCGTCC-box
+ CGTCA-motif

Site Name	Organism	Position	Strand	Matrix score.	sequence	function
CGTCA-motif	Hordeum vulgare	852	+	5	CGTCA	cis-acting regulatory element involved in the MeJA-responsiver
CGTCA-motif	Hordeum vulgare	957	-	5	CGTCA	cis-acting regulatory element involved in the MeJA-responsiver

+ ERE
+ G-Box
+ G-box
+ GATA-motif
+ GT1-motif
+ Gap-box
+ LTR

Site Name	Organism	Position	Strand	Matrix score.	sequence	function
LTR	Hordeum vulgare	260	+	6	CCGAAA	cis-acting element involved in low-temperature responsivenes

+ MBS

Site Name	Organism	Position	Strand	Matrix score.	sequence	function
MBS	Arabidopsis thaliana	1921	-	6	CAACTG	MYB binding site involved in drought-inducibility

+ MBSI
+ MYB
+ MYB-like sequence
+ MYC
+ Myb
+ STRE
+ Sp1
+ TATA-box
+ TCA-element

Site Name	Organism	Position	Strand	Matrix score.	sequence	function
TCA-element	Brassica oleracea	1574	+	9	TCAGAAGAGG	cis-acting element involved in salicylic acid responsivenes

+ TGACG-motif
+ Unnamed__1
+ Unnamed__2
+ Unnamed__4
+ Unnamed__6
+ W box
+ as-1
